# Supplementary material for: Chemosensory Gene Families in the Oligophagous Pear Pest Cacopsylla chinensis (Hemiptera: Psyllidae)
Source: Insects. 2019 Jun 17;10(6):175. doi: 10.3390/insects10060175 (PMC6628306; doi:10.3390/insects10060175)
Supplement: Supplementary file 1 [file insects-10-00175-s001.zip › Supplementary materials/Table S1.docx]

**Table S1 Amino acid sequences of *C. chinensi* and other insect used in phylogenetic analyses.**

**OBP**

>CchiOBP1

YSFDNPEFNEHLRQSLDEYMYGEDEEPRFKRNAKDANDDEKFVCKQKHFKSCCGNESTMKKLHQKEKDVGKECHKNVTSKFEELKVNTNHPRADPMNFFSCEGMMNMKKKIYCVNECIAKTQKLLNEDGSLNEENLRSYVLGESFKDEWLKELVTHGVDECLKKDFYKISSPKEETTPAYPTDAALECNPTYLQFLSCLYKEVEIGCPEQHQKNSKKCKAIRKNFTENKKRMLDLLKN

>CchiOBP2

SLDAPAETVELIGKKALPADESQRCFLECVYKDLAVVKDNKFDAEVVHTLAKERFPDGGEPLTKASTMIDTCAKEIVADEKESCAMGSAVRACFYKYSDDSHPPL

>CchiOBP3

QISAMDEEGNVDPDVFTGTVPEDYKGYAEGVIKSCKTVGGTTPCEKAYNLNICAQKVDPAKYMLI

>CchiOBP4 1-25

ALPEAQINAFKKQVKTQCLAKTKVDPALIDKVLVGEFADDHKLECFTKCVMEKSLVTTKGRIDWKKIQATSKAMLPPKLAKKVEVVAAVCKDIELQEDLCKHAMMATKCVYEQDQDLFKFMN

>CchiOBP5 1-21

VDIESFTTISPVVKQATTPKLSVAQNLNKAAMECQSKQGVNQAFMGLLSTKNIPTNEKMRCFLLCVYETLGLVQDNKFQATNAKLMATQRFSGEELKKANHLIDTCNKTLSGPIEPNPKEYCLMGRLVRTCFKEEGDKISFFPKA

>CchiOBP6

AGIIENTKFSKAGAEKLAERYYGDNVEDMNKARSIIDNCDSFVSDETEECNLATKLAFCVVEEAQKVGLSSVPGG

>CchiOBP7

SKQLAAQRFKGEELTKANKLVDTCAKEAIIPAGTVEKCALGKSVRSCFVKNGDKINFFPKA

>CchiOBP8

LECVYKSLDIVKDNKFNSDAAKAIIKERFEGDQLIKAEHAVEACGKELANADSSAESCALGKTVRACFAKNADESLPPLLAQ

>CchiOBP9

RHLQNSALSDQADFVSVCNSTWPTETDILSSVISEKKFQSIHNKNFKCFLHCLYVHYNWMDQTGGFLLDNMKDELMETSLDDESAEIILFKCTAIHSTHSCDRAYRFTDCFWKETQMYGDVETNEINKYEHVYVK

>CchiOBP10

MICNIQQIDSYSKGDFDNLAKVCPNVTQDDLEICKNFEVPDSPSGKCFLRCALQTMGMMATTGGIDKDNSIQTMIKSWPNFSAELNTKIFTNCYDQVHPLEKELSGTCELAYKLMKCLNVETRKNGYYKDFLKNR

>CchiOBP11

YPNDVSTAAINKFKTSVDKCKTDLNASPEAVNLIGKKVLPTDESQRCFLECVYKDLDIIKDDKFVPEVSKGFAKARYPEGGEEFTN

>CchiOBP12

YEFEDGESFDCEQMMKNERLVNGIVDCLKSDSPDCGTIMFTKIKELAPEIMQTLCAKCTDAQKTKFKVCTNEFIKIRPDDYEILVKKYDPDNKYRGPLEEYLKS

>AgosOBP2

SDPCNISTCYKSGTTKPPTTVTPTRLPVQSSSTPTSHQQTTYAKDHVHSSTATKSGVNTTATTTSGASVNGTERTTVVKSSSGVAGNVTTPKPTMTDGHVALKQKLNTIAVKCKDELHAPQEIMALVSNTVVPQNEQQRCYLECVYKNLNLIKNNKFSVDDGKAMAKIRFANQPEEHKKAVTIIETCEKEAIIDPKTTEKCAAGRVIRNCFVKNGEKINFFPKA

>AgosOBP3

RFTTEQIDHYGKACNATEDDLVIVKSYKVPTSDTGKCLMKCMISKLGLLNDDGSYNKTGMEAGLKKYWSEWSTDTIESINNKCYEEALLVSKDIIATCNYAYVVMACLNKQLKLDNST

>AgosOBP4

QKQELSGKCRAPDKAPLNLEIIINICQEEIKSALLQEALDILNDGTLEQNTPSYSRSKRDADEDLSNEERRVAGCLLQCVYKKVKAVDETGFPVVDGLMKLYNEGVQDRNYYMATLSAVRHCISIAQQLKQQQPSKSFDDGQTCDLAYEMFECVSEKIEENCGVENKLNNLSQRQV

>AgosOBP5

EAGHQRRGKELLDTEDSDFFRCKQASRKSCCGPENAMKRFGDKDKVAADECYAQVAEKFATVTATTPKQDLFSGEAVKITKKKQFCLHECIGKKNKLLTEDGSLNKTFIADYAMKSVFKEQWQKQIGQKALDKCLEETYIPWPAEETENKCNPVYVQFQHCLWLEYESNCPDNKIKLTKKCEKTRNRYRMQKSPSNQ

>AgosOBP6

VGFERTWILRQKRMTNDNECRALFPSPEKKLPTCCQMPNILPGLDNAWEVCFEKFKQFKDKHATKEYKEMVHENEPPCLFQCVFMQSGLTTSDGKVNEDAVIKKMAEGMDNDEKWKSIWRNTFNKCLNDVKQEDKEQIKVMNTPTGRLMKCFLRDLYMNCPKNVWVENSECSNLKDLVEKCPKLPPPVFQSPPKLI

>AgosOBP7

YLSEEAIKKTQKMLKNVCSKKHSVEEEVFTDIKKGIFPENNNNIKCYFACNFKTMQMVNQKGILDKKMFKDKMTMLAPPNVLAILLPPIEQCIGNDKDTEICQSSYNFIKCAHRVDPKSLEFLPL

>AgosOBP8

ENNQQNSNDRSASIFQSCISETKLSGDALKGFRSMSIPKTQAEKCMMGCLMRKVNVINKGKFSVEEATKVAQKYYGTNESMMKKAKDLIDVCAKKAQSTTEECALAGIVTTCIVEEAQKAGLTGGPGSRSKRTVSPKFRHSIV

>AgosOBP9

ADDADTADKELMSKLITVAFKCFKDADWGTCGEMITTKYDITQAKYKQCTCHMACAGEDLGLINSNGQPEPAKFLEYVKRINNSVIKSQLQHIYDKCQNVKGTEKCDLAEQFAICAFKESPEMKERVTKLIEMLVKMKPKSK

>AgosOBP10

RPQPDEPDDIKKTLYNACSEKFPLTEEIKNNVKNSMVIDDQNFKCFLRCCFDEMSLIDEDGIIDGESLAAMAVDKIKPVAEKIVHDCLPAGKQEKQDGCEAAFKFFSCGMKLNPLTIELLPLQ

>DcitOBP1

MEFLVRGEIKSTSFKFLIFLVLFLSVNNILQTVSYSTKEFDNLAKVCNVSAEDLEICKKYEVPDSESGKCFLRCALQKLGMMNPAGDIDKEASIQTMIKSWSRFSSELNTELFTECYNQVQPVTKELAGTCELAYKLMKCLNVVTRKHGYYNDFLKSDK

>DcitOBP2

YSFDNHELNEHLRQNLEEYMHLADEEPRGKRDAKDEGNGSDDKFVCKHRHFRSCCGDDNLMKKFIEKDKSIGKSCYRNVSAKMDELKTNQQMGDPMDFFSCEGLKNMKKKIYCVNECIGKKQELLNEDGSLNQEKIQAYVTAENFKEDWQKDLVTAGLNECLKKDFYKTSFPKEESTDGAGLNCNPTYLQFLSCLYKEVEIGCPDQYQKNNKKCKAMRENFANNKKRMLDLLKN

>DcitOBP3

EIDESMKAVAKMIHDQCVDDSGVDPAVMDRIFSTKTMEPDEKFKCYLMCGLQQISAMDDDGNVDSEVFIGTMPEEFKSYAMEVVKKCSDVGGTSPCDKAYNMNVCAQKVDPNKYVFI

>DcitOBP4

QTQHRPQPTPAKAAEPRKYSKCEPPTSAPQKLEKIIGQCQDEIKQALLQEALEVIDDVKQQIGNSNNNAYNTPHTRTKRENFSGEERRVAGCLLQCVYRKVKAVDSNNFPSAEGLVRLYSEGVQDRNYFTATYQAVQFCMKLAEQVRQSKPNGVLDGGQTCDLAYDMFNCVSDQIEKFCEVQA

>DcitOBP5

MVPVYLYYSQSVSPQCELNHAPSSTQEIVSLVIMTLGQITLSAVLSMIVAVVSGRYIQNSAEDRADFVTICNSTWPTDTDILSSVISEKKFLSIHNKNFKCFLHCLYVHYDWMDQTGGFHLDNMKEELIKTELDDESLDVILFRCPETHSTHACDRAYRFTDCFWRTTMMYGDMDNNELVKYDIHDQD

>DcitOBP6

ALPEAQMKAFKKQIKNQCVQKTKIDPAMIEGALNGNFPEDHKFECFVKCIMDKSLVTTKGRIDWKKIQTTSKAMLPPKLAKKVEAVAAVCKDIELQEDLCKHAVAATKCVYENDKELFKFMNPE

>DcitOBP7

QTTIAPQKPTTPKLSVAQNLNKAAMECQSRLGVNQAFMSLLSTKNIPTNEKMRCFLLCVYETLGLVKDNKFIGSNARLMAARRFAEPELSKANQLIDVCEKSIVVPSNSTEFCALGRLVRKCFKEEGEKIDFFPRA

>DcitOBP8

DEAENRERVQQIYAKCMSETQAEDKDLEGFRKMQIPDSEKGKCMMACLMKEAGIIENTKFSKEGAERLAQRYYADNEENMTKARSIIDSCDAYVSEETEECSLATKLAFCVVEEAQKVGLSSVPGG

>DcitOBP9

MAQKVNAVATKCKTELNSPPEAVALLGAKSLPTDEKQRCFLECVYKNLGIIKDNKFSPEGSKALAAQRFKGDELTKANKLVETCAKEAVVAAGNTEKCALGKSVRSCFVKNGEKIDFFPKA

>SfurOBP1

DEATSSSSPDADSLITSTTLSPTSNETDAARASIKEQLAKLTESCKTSSQANSDDAKIIETESVPKTEGEKCFLQCVYGGLGIVKHDQFSVEGAKLLAQKRFGSFPEELEKANQLIETCSKEALKKDSKDKCPMGFLIRQCFVKNGQKINFFPKA

>SfurOBP2

GLTPEKLKEIKPLIDTCIKESKVEEETLGKLHNGHEIPSSQSGKCFIACMAEHMKLMKDGKFEPAMTMEFIDKMVQDKAKADEIKKAVDDCFKSVPDGDKCEMAASLATCMKEHHAELAGMN

>SfurOBP3

KIDKAKKEAAIKKCQAETSASDEDVKKVRKEHVVPDSEEGKCFIACGFNHYDMLKDNRINLEGVNAFFEKLYDEQEKRDIAIKAAASCAATESVSGLNDCHVAAKYFACLQRHPDFVKMKDDFDV

>SfurOBP4

EDTTIKIKNQQSPHKQQQVYCQAPPTAPERLERIIEQCQDDIKTALLQEALNVLTDTSPRDLVKKTRSKREVFSGEEKRIAGCLLQCVYRKVKAVDDQGMPTVPGLVRLYSEGVQDRNYYVATVQAVQQCVSASQHFRYYNPQVLKEDGYTCDLAYDMFNCVSDKIEAFCGRTP

>SfurOBP5

YDFSDPYFNEHLQSAMEEIMEEEMLSIGRVQRDADQTQEVADEYFKCKHRNLKTCCGKINLMKNYGDKGKIYGKQCYEEVVSAFKTNSSSTADDDDSMMDMFSCEKVKMIKLKHICVHECIGKKTKILKEDGSLNAEEIKQYAREYMFNEEWSKELGERALDKCLTQSYNSVTKMLDEYEIKCNPTSVQFHHCLWKEIEMTCPESKVDLKAKCVRLRERLRKQQAAGM

>SfurOBP6

VFVDRENGYRLTRQAPPDDECRPPRPGHNDDGVCCDMPPVFRTAHDKFESCLEELSSIFPPPPPPNGHHGPPPPGGPGGHGPPPPPPPGGRRGPPPGFGGPPGHEPPIFACAHECLFNKTGLLENGKLNVEALKKKLEGELGDDEVWKNLLQSIVDKCMESKDPPSNDMCTSGSHELARCVLRDMFMNCPQEKWKESDDCSNMKMKLEKCPELVPPMAMRLSQPPMP

>SfurOBP7

QYGPIALSTAQSAATAGLPPSATNKVDTFNRVRREALEANERVVRESSEEEDEACKPTKAEHEAKMCCDLPLVYRGTPELFKACREELGFPDHKPPPPPPSSDGHGPHGHPQRGMCVAECLFNRTGLLENGKINKEALKKALDEYLKTDGAWKDVATTTLEICYDAQTRGDFKPDNEKFTSGSSEFLKCFTRNLFMDCIPEKWTDSEECKKMKEKIDKCPKMLPPALFNKRPH

>SfurOBP8

VITQIMAADSNNPDMQTVFNNCREEASATEDDIKTFRAQQIPSTTTGKCMLACMFNHSGLMKEGKYNSEGALKLVGQVFAADPVKLGKAKTLINTCSDEVKNENDKCEIASKIADCTVKMTSQVGLS

>SfurOBP9

MQADFAMMQFPMQGTGTPMIQSIAGELKYCMDVNAEQNSDGLEDYLPLLFNEELPTSLGQKCFLTCLFNRFGLLKDGFLDAQTAKTLVETFYKDKHDEKTMANIAINVCRVSAVPDILNPCEIGFSLKSCFVDSNKKGKELRGKN

>SfurOBP10

IPALTEAQIEQVGKAMANMCISSSGVQRSLITKAMTGEIEDDRKLKCFFGCIMEAVQVTKNGKMQPEVLKRRANAMLPKTMREMILPTIDSCSHIENEDKCELAYSIVKCHFSVNGKNPFFFNF

>SfurOBP11

RFSEEEKQLMNQVHTQCVTETGTSEDLVNKATNGDFAEDENLKCYVKCIWSTLTVMDDDGNFDVGVLEVMLPADMKDIVMKAMSACIGAGGGSPCEKAFAVTKCLYKEAPADFFLP

>SfurOBP12

LVAAELEKLKNSCLKKSGATEDTARRLVGINVVTENHVESCFLTCIYKGLKIVSSDNKFQPDTVKKIADDHFLGRNLKVTYQIADSCTKEIKADPADKCSIGASFRNCFSKYGKELGFFPHM

**CSP**

>CchiCSP1

EDDKVTAHNLSKFLKKFQHININTITHNDRLLTSYTSCFLNKGPCSTESRELKRAFPVIMKTSCGSCKSEQKKKVQTLLK

>CchiCSP2

YEFEDGESFDCEQMMKNERLVNGIVDCLKSDSPDCGTIMFTKIKELAPEIMQTLCAKCTDAQKTKFKVCTNEFIKIRPDDYEILVKKYDPDNKYRGPLEEYLKS

>CchiCSP3

ASAPYTTKYDNVNLDEILANDRLVSNYIKCLLDEGTCTPDGAELKSVLPDALKTSCTSCSDKQRSGSKRIFKYLIEKKPEEWAKLEQKYDPEGIYKAKYQAELKALNINAAHEEVASPVVAPVDSISKSETVAKSETSAKVEAEAKPVETKSESENKTPSH

>CchiCSP4

SPAYSTKYDNINIDEILSNDRLFNGYYKCLMDSGTCSPDGAELKKSLPDALKTECGGCSDKQKEGSKKIFKFLIDNKPEQWANLEKKYDPTGTYKAKYSAQLA

>CchiCSP5

SKRLVSNYVNCLTDKGPCSPEGADLKKTIPTVLQTLCEKCTKSQKEKAVTVMKRLKKDYPAEWETLMKKWDPQGEYKKKFMEKFPELKQEE

>CchiCSP6

AKDEIPAYPTRYDAIDVDLILGNDRIIRRFVDCILGRGPCTREGLELKRIIPDAIRTECTKCNPSQKKHVGKVLSYLFHNRKNYWDELLAKFDPDKSLREKYGFT

>CchiCSP7

APQKAKEAESEGGSSILDNFDLDSVLNNSRVLKNYVKCTLGTGPCTAEGREMKRVLPEVLKTACGKCSPSQRDRLRRTLNKLKNDPKLVEDYNKIVQKYDPKKEYVENLEKFLLSGDKKSA

>CchiCSP8

ASVTPKPTSVETKSEDKPVVSTSSSSSSSSSKSAATSAKPVATPTKSAAAPAKSASSVSDAELDKLLLDRRYLSRQLKCALGEGVCDPVGRRLKSFAPLVLRGNCPQCTPTETRQIQKVLSHVQRNYPKEWAKIIQQFTGN

>CchiCSP9

YTNRYDGFDIDSVLKSERMYKSYLNCFLDLGPCNPMSEEGKRLIPEIINTSCAKCSPHQKYLICKVFKLILEQKPDDLQKLLAKYDPQNKH

>CchiCSP10

ANVATTPSAKTQQTSAAPAASDEKNHGGSLLDKLPIMDLLKNPKVRDGYTGCLLEKGPCTPDAAEVKGIMPEALTTGCAKCTPTQKAVFEKLILHYNKKQPEIFKEVTAKYDPKGEFKKNHLAKLKKSVKKD

>CchiCSP11

AYTTKYDNVDLDEILANDRLFSNYYKCLLDQGNCSPDGAELKKILPDAIATECGGCSEKQKEGSKKVFKFLIEKKPEQWKALEAKYDPSGSYKAKYDAQLKAL

>AgosCSP1

MNILTIFCYVTVMCDTQVKPAVSAQRLQSVNQNVTPTNDGRKTIRETSSYPTRYDYIDIEAVMNNERIIKILFNCVMSRGPCTREGLELKRIVPDAIQTECAKCNERQRKQAGKVLAHLLQYKPEYWKMLVQKFDPNNVYLRKYMADNDDDEKLSLQKLSNDTTKKKRNI

>AgosCSP2

AEEKYTTKFDNFDVDKVLNNNRILTSYIKCLLDEGNCTNEGRELKRVLPDALKTDCSKCTDVQKDRSEKVIKFLIKNRSTDFDRLTAKYDPTGEYKKNLEKFEKERASAKPLKA

>AgosCSP4

APQKDAVAASGPAYTTKYDHIDVDQVLASKRLVNSYVQCLLDKKPCTPEGAELRKILPDALKTQCAKCNATQKNAALKVVDRLQKDYDAEWKQLLDKWDPKREHFQKFQQFLAEEKKKGFTKF

>AgosCSP5

SPAGTATAAAVSADDEIKDFPAYMKRFDKLNVEQVLNNDRVLASHLKCFLNEGPCVQQSRDLKRVIPVIANNGCNGCTERQMTTIKKSLNFLRTKKPTEWARLVKIYDPSGTKLNKFLDA

>AgosCSP6

APAKYTTKYDNVNIDEILNNDRLVASYFKCLMETGKCTPEGEEIKRWLPEAIENKCEDCSEKQKLGSEKIIKFLFEKKNDMWKQLEAKYDPQGTYRQRYAEEAKKLNINV

>AgosCSP7

RPEDVKVENKPAVIKSETLAAPLPTNIVKRATDTIQLDSSLPNVSEDVLDKALSDRRFVQRQLKCATGEGPCDPIGRKIKAHAPLVLRGMCVKCSQSEIKQIQRVMSHIQKNYPKEYTKMLKQYQSGF

>AgosCSP8

ADGGIITPQQQQQQTMMFTAPTGYYVSTYDHIDVGRLLRNNKVVSGYVKCFVNEGPCTPDGKLVKAYLLPEIIRTVCGKCTPRQKDMARMVLKHIYTYRQADFEKIMQIYDTDGKRNEILAFMNH

>AgosCSP9

TFTRSTKFDDRTGIDIHLVKRDTDDVNDDENSVESDEGFFYRFTHFFQDSSDKEDDDDDEKKPDFITTFDIFKLLDEEYAMQQFYCVINEDPCDEVGMRLKATIPEEINRNCERCTSTERNNIRRILNYVKKHYPQFWKRVEPIYKKKI

>AgosCSP10

MINTRPRKLVRCIRGVSISVAKGDDAVNAENKDDDSHLVNREEIQRYMSMMEKINIDQMLNNTRLMSNNVKCFLNEGPCTAHLREMKKMVPMLVKDSCSSCTKEQKIMMKKAMDAVKARRPNDYEKLSKFFDPEGKYEKKFLENLNESK

>DcitCSP1

AVTPKPADSKPAIDSDADPAKPAVTSSTSSTSSSSKSAAKSVTPKSAPAIKSEAPKSEAPAKSEPAAKSATKAPAAAPSKATSSVSDDELDKLLQDRRYLSRQLKCALGEGVCDPVGRRLKSFAPLVLRGACPQCTPAETRQIQKVLSHVQRNYPKEWAKIIQQFTGNK

>DcitCSP2

EEDLEKKYADFDIEAVLNSKRLVTNYVNCLTDKGACSPEGKDLKKNIPTVLQTLCEKCTPSQTDKAVMVIRRLKKDYPEEWKILLEKWDPKGEYTRKFQEKFGDTYKV

>DcitCSP3

QKKQAESAPESGILDNFDVDSVLNNGRVLKNYVKCVLNMGPCTAEGREMKRVLPDVLKTACGKCSEQHKERLRKVLLKLKNDPKLKEDYNKIIEKYDPKKEYVANLEKFLLS

>DcitCSP4

MLFTVCYFVSQVISPVVFPTVKMYKVLAVAVCCAVIASCLAKPQTKKYTTKYDNINLEEIIHNDRLLNNYYECLIGTGNCTPDGAELKRLLPDALENECKACSEKQKEGSEVMIKYLIDNKPEMWAELEKKYDSTGSYRKKYAAEAEKRGLKV

>DcitCSP5

MKTSCGSCKSEQKKKVQTLLKAIKSHKPKEWKEMLALYDPSEDYRKQINKLVKPKKQSSD

>DcitCSP6

YTNRYDNFDIDSVLKSDRMYKSYLNCFLDLGPCNPMAEEGKKLIPEIIDSSCAKCSPHQKYLICKVFKIILEQKPD

>DcitCSP7

APAESVYTNKFDNFDVDKVLNNERILTNYIKCLMDEGSCTNEGRELKKTIPDALAGGCSKCTEKQKAATEKVINHVMSKRKPDWERLSKKYDPNGFYETKYKALLEKAKHPAAPAAATVTKEGDAKKDLKESKEPVKVASPAPLAKPTNDKPAKSADPKAAVRAPGPALPAPGPALPKEDALLQAALGKKAAPAVPAVPAPIR

>DcitCSP8

KPAGEKYTTKYDNVDLDQILRNDRLFNNYYNCLLDKGKCSPDGQELKNKLPDAIATECKSCSEKQKEGSKRIFKYLIDNKPEQWAELEKKFDPNGTYKAKYQKELSDLKAGKPVKI

>DcitCSP9

EDVNKYLANPQYVEQQIDCVLDRGNCDPIGRNLKAAIPLALRENCRACSSKQVANARKLGSYISERYPREWRQIQA

>DcitCSP10

SPAYTTKYDNVDLDEIIANDRLFSNYYKCLMDTGACSPDGAELKKVLPDAIATECGGCSDKQKEGAKKIFKFLIEKKPEEWKALEGKYDPSGSYKAKYDAQLKAL

>DcitCSP11

RPQNSIQSSTVKDEIPTYPTRYDSIDVDLILSNDRIMRRYIECILNKGPCTREGLELKRIIPDAIRTECAKCNPSQKKHVGKVLSYLFHNRKNYWDDLLAKFDPDKSLREKYGFT

>DcitCSP12

YEFENEENFDCEVLMKNERLVKGLADCLTSDELECGNVMFSQVKKYAPEILETVCAKCTDQQKATFKKCTNLFMQSHHDDYEAIMKKMDPENKYRGPLEAFLAEP

>SfurCSP1

APEEAQYTTKYDKINLDEILNNDRLFKSYFGCLMGGKCTPDGQTLRDILPDALETACSKCSDTQKAGTEKVFKFMIEKKPSEFADLEKKYDPNGKYRARYEADAEKFGIKV

>SfurCSP2

MVLADTPTTSPKVETKAVESGKSSSKDEIPDQTFDRYINNERYMLMQYECLMGNKPCDHVGRKLKAAVPLVVRGLGCPKCSQREEDQMKRIVSHVQRSYPDKWQKLIKKYGN

>SfurCSP3

DMPQSTYPTKYDDYNPDDILKNDRLFNQYFICLTKKKGCTTAGELLSAIIPDALATSCAKCSAKQKAIGEKVIRFLYFNKPDEFAEMSKIYDPEGKYLEMYIASGGLI

>SfurCSP4

ATKEKDPERKALYRLEYIDIEKVLDNNRMLTNFIRCFLRKGPCSPEARDFRKLLPKLAKTMCSDCSPRQRFIIKKVFKHLMEERPKEWELLMDRFDPQRKYAERLDTFMVDMTTPSTTTTTTSTTPSTPMSSTTQRIIEILRTSTEMSNESSP

>SfurCSP5

QQQQTQKPQQQNVDNIEMSIYDKMFENMDVNSLLKNHRLVDSYLKCFLNEGSCTHIGHEVKMMIPEVIRSRCATCGENQMRALKAGLRLFIVLRPDDWKRFLDVYDPDRTEWPHIKAFMESDD

>SfurCSP6

QSEKSEKPEKYTTKYDYINVDEILSNDRLFNSYYKCLMGGKCTPGGPELRTHLPDALQTNCSKCSEKQKEFSDKVIKHLMDNKPEEFSALVKKYDPEGIYKDAFKPKHNQ

>SfurCSP7

QQKSKDTRYTTRFDSIDVEVILKNERIFKRYMDCLLDKGRCTPEARELKRLLPEALKTECLKCSEVQRRQGAKVMGFIIKNKRPYWDLLLAKYDPQGIFRAKYNYNENNIEGVLKQLEREQQGLYGTYSNTTNTTNTVNSTSTRK

>SfurCSP8

GKLYKDRYTTKFDKIDLDEALNNQRLFESYLKCLMGDKCSPDGYELREALPDALATACAKCSDAQKAGTEKVIRFLIEKRPKEYALLEKEYDPEGIYRDKYKPIAQEKGIKI

>SfurCSP9

DEANKYTSKYDNIDIDKILKNDRVLSQYIKCLMGEGSCTQEGRELKRLLPDAIQSNCSKCSEKQRQASVKVMRHLRQSKERDWNRLLDKYDPQGDKRKNLKLD

**OR**

>CchiOR1

IRGQDELATIEEKELELKAARNDLKTDSDGFLEANVKSSHQKISGLIPNSLPVPAEYRKSSFNDLDEREITFDENYLKIRQVYERKYFKQLVKFHQKLLKNIEQMMKDISELVLLMVLGNYFVSSLALYQLTLQTETTSMTRLFKFLVEYVLVLVQFYTYCKAAESLDDNQSNTRNSIYFSAWYRLCPSVRREMCVLLSRLLKSNHPSFFHGVFVLQNPVLTRLLKVSYQVVNLIRLKSTRV

>CchiOR2

AFSTIGYLFYALAQVFFFCFYGNELIEQSSSVMEAAYSCSWYDGSEDAKTFVQIVSQQCQKSLSITGSKFFTVSLDLFASVLGAVVTYFMVLVQLQ

>CchiOR3

FKYAIIIPTLYAPQFVKFLNPLAFSCLAVNLFISILCLFQLVVSTVDMSPQRYAKFCAELFGVLMVFFMFCESSEMADEVNGWMRQALTQCGWENCSNKTKRQLLMLIRCAQQPNYCRFNNGAVIMSRLAFLNVLKCAYSFVNVFKSTTH

>CchiOR4

VNTIVLSVCLYEVMVFSSETIGQARYTKFVGQCGAVIIQYFFMCNMSEMADDCHTIMRRALINSGWTMSRNSIGRDVCIIYRRVEGGNHLTFNDGLLVLSRVLFVDVVKLSYSFCNFMRLGEQMT

>CchiOR5

MKLKEILRQINPVWLCLSMKNYLNTFLVSSHEKILLRSLLTFAEKIGIMHEQHGGKRIMQSVYVFTVYSILIIFIGCHLYNTITRSVRYLPKCFQAIVEDLYLGVLTYVCNYFRLHYEQLDSLMILLESMYQSNVKVRQICQNKSKLVLGLLGIPVIVGGAINVGEALLPLSPHNLDIQRMVYKTCKHPERRLPLPVRIPFVDETESWAFEPLFLIQIYILFLIVSIFITVFSILPIILIYIQGQYKILSKYIEKIGQDHRDSNGNAIEYTNIEINRFRRVEKGLLNNVKLARLGLRNVIKMSVESKKQYEKTYIKQIIQYQQKLIVMHNKVC

>CchiOR6

MQILDFFETTAQLHREHKAVCRLQNIEGIFFPVALVVTGVLMTLHVIVVPCLPKSSEELETLSKFYNLEYPQNVLAFPVFVPNVDISNPKLFYFFLGLEIYLAFLVSLAIVLTIVVYPLSVLNLKCYFTILRDFTLSVGKTHFNSSGRPIFYTNLLENKKVMRRYLQDGKKIEPPMSEQDKHLSRWFEQHPDLYEAFYLREIILFQKTLIRQRQCLDEYFANKFLLWYPMSMIVLVTSMYGGFTPILATTLARGKMIIEAGATLTYIFMHFYSGELLADCNESLGTGLWLSQWYTCSTRTQRDMIVFLRMNQKLDYTTVMYVLQVGYKSMMSGIKFGYSVINALRMRKNTRF

>CchiOR7

MVSFNQICQLLQYGGRVNLHQHKSNWKSTLQDIYVFFQETLCTLLTLCHLFSAITRAVRYLPEFFQTLCDNIIFWYLNCYCFYYQIRNRQLCRLIELTEDTFTKADQKIVQKYNRRSNFVSIGYFNVIFSYFMFTLILETAMPIPENELDIRRFVYRTAHPERRHAAPVRIPFLDESKSWTYEIIYVLQIYLLAFYSLWATSLSSTVPVTMMHAQGQYEILCKYICMIGKEHRDMLGNRIFYTSIERNEYVTESTPPVRRRNTQNRMNRKLKIYQLYEQNYLGQIVKFHQKLQHVQNEVSLQYLDLDTYLHIFFYDQSNFE

>SfurOR73

TAVLTITIFQVSFLSVNNVITEMQLLLMSLEEFNVNFPIGYNDEKNEGNGKLFDDEEGNFPRSKLKVLFNDICEHHQYIFRKIEILNEGYRYRLFYVNTFTCLQLCFALFCLQKGDLGSKVRYGLLSAAVILTNYVFSENGQRLENEGEKLREAFFNCCWIGKPHWLKSSLVILLSRMTKSPKIQAYGIFTLNKNNLKIVVQAAYSYYNFLGRFKERMNE*

>SfurOR33

PFDSSISPMNEITRFIFCLTILLAVPLVTIRFSLLPVMVLHAVGIFETVSADIAKLKYSCGDNTEEIIIEIIKLHQKALIISKNLVEFFRPIILLKAVFSFWMFAVLMVVITEEPIGSSIFINCAFCMLCSGYELLLDCWSGEQLARKSEKVGVAAYQCQWDRMLNGARRNLVLLTLRAQRPIQLDVRPYLIPMSLKTYVQIMKASFSCYTLLLNIKAEKH*

>SfurOR15

AFLFCEVFIVIMYIGASVPFLVYLVFEVNLQYMILERSILNLELRAEELYKKSVDLHSEIIVKNSLLYEDCVRECLRENIHHHLAIARLYNLYQDLTTTIYGIVIGLSMSVLATLALVLTRTKLFSFETMKFTSLFVIELTMVFGYCFMGTSLTEASSKVSNALYNCSWFNFPANQKKTLLIFQLCTSETIVLKGNGLFEINLQLFVQIVRTTYSIFNIFSSTDY*

>SfurOR124

MPENHSWKVLFYNIFTTIVFILITMMVGGTMMSAIVMVKIFPTLGVLQGYLSGTGTLVMLLLFILIKTRENLNKLVESVNNLYKFDNEDEDRKLYEQYSLIILSFIAVTMGSFFLVFVVITENLVDKHVVETNVFNHENPERVIGFMPWVPFDYTISPYFEISRVCVYAIVSAAYPVVVIRLALMPVLIMHVIGEFESISLRLSKISLNPGEGAEKRIEELIKSHQQILL*

>SfurOR85

IFYNPVTQSNISTDLTQYVILALVQCLYLYLSFNVCMDLYYSSVYSCSNVKTDMELFMLSIREFDEVCEIAIESYGEEMGRERRRILREYVKSLVRQHQIISRKMELLAEGFYAETIYVNAFVCVQICLAVFCFQRTEIMIFKIKYGFVAISTIVLCFVYSEECQVLYEKFEDMANSLFHSSWMTKPPWIQRSILILITRNTRVPIIGIDGIISTNRSHVRKVTQAAYSYFNFLSRVVGK*

>SfurOR123

MFHPLKLMNFFLTVTGLNPPPNPKGKVSYYNLLTIITFSLLSLLVVGTLRSATIMIKIVPTVGTLQIFLSATGFMMMIMMIILITNRQQLLALVESIDNVYKIDNSDEEKKIFRKISLKLLIFLSASIAIALSGFTIVTENFMDDRVVEANTNVFNRENPERVIGFLPWVPYDYTVSPYFEISRVCMYATMIMLYPVAVIRFSLMPVLILHVNGQFESISSRISQISSNPGSEIDESIKEVIVLHQQILL*

>SfurOR11

NICPTLTIHHVGLLSRGLQICQCLFNCTDNYFLMVCDDNLPMVHVPQIYATIHRMCHSSSQLNMRILEKIDEIIASKLTTAAAQTNIESYNSKPSEKNHLKDVFNGIIKDHQSICSAAKLLNGNVNGITMGFMNIFGFQLCIFLMFIVEAKDISLKVRYVFRYYLVLGILFFCTKKCQRIKDKGQQLRDAVYGSSWTDKPRWLRKSLTVMMTMANKDMQISPYGIYVIDMSYMTNIIKASFTYFNVLSALKA*

>SfurOR122

MESTRFLLKFTNFVRYGTPLNSLPFLSAIIAVALLFTNLFMVILYEHNSTERRLKAMAEIVSVTSVFSCVVQRGLRPEHAMKLIELIRGELLAYNCYIISVGKVKLGEIYANYDKKREDMERLYRKMLIVTTGLYFAYIGRNAFNELLEGKNKSDRKWPTPYIYWCPPGYDSFYTFIFLEILHIYVFSFMLAEAFCLMLSTCLATERVLADFQAIYMLIEDLSEQFMEHNIEFNDNYPQPNQKIDYDDKNVMRNLENYMALLIQCHQKLNR*

>SfurOR120

LYFLAISSYHLNLALISLSEFVFYLLDIKTDEKPPQPHSLHAFFTFPPSIVPKFFDKLFKTSLELFFIYYGTIINANVHIMQSLSISNIVGEMKLFHLNFSEIDEYFEEVLKSENYKNASDVIRKQMENKLRLATINIVKHHQNIFRKVQLLNYGTNYCVFCTNCYLCLEMVFMITMFLKGDSIDKIKYGLIFIGFVLFEFIYNEAGQRLEDEGERMEMTLYDSNWLEKPTWFLQTLKIMMIRSNKLPKVGVLNLFTLNRKNKTEVMRGAYGYFNFLNEF

>SfurOR89

MAKNLYIVLILLGSLMITRNILKYLLGMYETPENEATRWPTPYLSYFSQYFDNFEFFIFFYLLQAVVIVLTTFEGVLIFNSFHLLTEKLLTDFEIFYILLEKFAEDFPDVSIGVDCGYMNNSVEYPNKIDMEHPDQEIICNSHSSSIDINLKKDMRRIVALHQDIYRNFRICAYNSSYVLTIANFSVLLYGCTFAYLMLTSNDPKKTVHYAMFFILMNLTSFNAYNNGQRIFNQNDILRRSLTEVPWTDKPLWFKQTLHIMMTRANVDIEMRPYGIYTLNYLSFKELMKTVYSVGSVFRKYDSK*

>SfurOR94

ILLGIFERHKLRLRRMKCFKEISSKFYTEINVMESHSGHFLKLFLSMNFVYSYLPFLNAVYIRVTSEHSTNFKNVAQISYFYYPVSEVTFKHYCFGAMFLYIGLIYASVFSFCILYSSYLALHCVVTAFSQLRTLILEWDESSEKEVEGEVNMEECLLEIVVFHQEICSEARLLNKGMETATMNLLQGCVVQICLSLFNILEGGDKIKFGGFTIFILLMLVGFSSFGQILEDRIARIKLTLWESRWVEKPVSVRKTLFMMMIGASTQIELRPFGLRAGLNMQSFAKVLRTVYSLFNVINHSSSIHNTDFS*

>SfurOR22

MVLLLSEVGRAPEKLQTILGIVKYGVKYSINMKLSEKQKLIIKQANKNDLLMCFCFIGTVTCYAITTGIIPAISGFIKISTGDLDDDILEKEIPMPVAGSWLPFSIKNLSTYTLLAAFQQFAISLHLFIYMGWFAMTSSAMMNISSALKLLACFVDEMDERLGNIQEGKPLDNYIKFMVDYHNAIYKTIKDYDSATAVMMLLLYAVCVVEQCVSFFCIYEVNDRGLQITFLGVLIVNILILGSFTFFGQFIIDEGEKLRLSLNHSNWINKCDSYKRSLLIIMANTQKDVALKPAGLYVLDRHTIMLIANASYSYLSLMRNFKK*

>SfurOR62

NGEVICRNEELFEKNCGVIGENEDLCERTLNPEIPSDNKRLQKEFFHRKSMEIIEEMRSTNLNYTRTGLLLLQGLSMAFLLVPLHGFISYMIGAIEFQELKFAIMVYEPFTGSSSPANFAQFIAVTIVQALYIYLFYMLSSDSFLIESLSNCTLKTEIKLFLNNLEEFDAIFCARKWDDKDGILSARLRKLKIFEKFGRIAAHHQIIFRETRIYQKTMAYLTVYFNLGVCGGMCISLFLVEQGDFFFKIKYAVAACCLGLALALYSQEGQDIEDGFEEMKDALYRISWHNLSVSLRKSLAILLTRHCLPPKVTGSYGIFVINRKNLSMI

>SfurOR86

MININYLYSQYAVTLVHVIEEECSVCERDCEGREGLWEKIDLIHKRNFEVMTFTDKCIRITFIIMFWGYVTKNAIAHILRIPIYTDVQSIPWVTPFLWFTDSEFEASLYFFLIFIVHTLVHVVIHLEAYLTISLACVPVQTLFGDFEILFILLNQFREYFPDNNEDQNCCEHDEKYSSLKQYMAKIVQCHQRISRNHNICSRNSAFLILAAVIVVMSESCNNAFFMLKAQGFKKAVLFAIMFVLVNIALFFVYQNGQKIINQNDILRRHLAELTWLNKPRWLRQSLHIMMTRANVDVQIKPYGIFTLDYNAYKDLMKFFFTVGNVLYTRKLSNQT*

>SfurOR59

MLERELQLKYLQEQQLILSNQLEDSPKKHCAEEAEKENSTERVSSEQSQDEKQDQASQQQTTKEQTFKKLSSSEIRTGPVSSRIQQIIQRIEEAAGTSAKPQKAVQNDFYEPVDTSDGIVHSRTIRDSNLTMFEVIDVFTGAVPGFIAFAMLCCFCWRVDDVRCLKQLFDKRLLSFGTNDDKEIVRENIKRSNMFIFIYMGPVYSNLIFWLIRPLFSNDKSIFNTINNGTEIIYKIVDCHYPFDYTVTPNYYYVCLYEIVGVYQLITLMLVFDTLFGSMILVFCGQLKVIKFNIDKLKMDKLQISQQNGEQRVFNVTMEENLKCIIKNHQKLISPVGQCD

>SfurOR111

MGIKDLVHVTASFSVLIQLSFNPQGAVKLVKVIENGCIVDISKITGRERNWRRIKKCYMMRRKNMEKMFKFIYYLIIVVYVGAVNRNLFSSLLGSTSGSKKTRAWLTPFVYWSPSGFDSFSYFIVIYIFHSILLLFMAFQGFFIEITVYLATEKVLADFETIFLLLDDIAANFSEIELMDETSTDNEHRINFLDSKLRRDMSLIVECHQRFNRNFKDCADFSAYGILVNTLNISIDTVFNIYLMMKAEDLRTSINYGVASFLVNLLGFFRFHTGNKIVNQNDIFRQAIANLPWSDKDQWFKKTIAIMMGRANIDTEIKPYNIFVLDHKTYKNLMKSIFSYGNFLYTTSKPTE*

>SfurOR61

MVLWQIPKEDWLRIKRGLEKSGIRFVIASGLLVSPEQPWLTINLLMMLLAIIFMSFFVYVGGVSMYYARNDLTTFVEIAHAYTFVLAFWVLLTVHFVFKLPVLHELLQTVDGEIFEYKNKCCEEKEAKVKSQNRFYHLLFQTVLDSAVLVAVFILGFLAPAMIKLFEDENRRKVKEINYNFPVPLWFPFRTDNLLGFCFAYFLLMVEILLIGIYLLAAIPFLVFAALEVNTQYRILKNSILNLEPRALEKYNGMCRVSNNQLEILRQDSFFKKCVQECIKENILHHIEILRFFSLYQDLSSTIFGVIIGVSMIILASLSLVLTKTELLSFDTLKFAFFFIVELVVVFGYCLMGTFLTNS

>SfurOR129

MAFFLYVGAVSMYYAKDELMSFVEIAHAYSFMFAISTLLTLHYLFNLRGTLEVIKLIDSGVFEYRTKYGAEGDNKFRKTMEFYYRIFERVFRSAIWVAYSLLVIFAPLLAAVFGNGYGHRVREITYNMPVPLWMPFNMDTSLGFSIGYSLICINIGLIGFYLTASVPFIVFMATEVCIQYGILKRSILALEHRVLERYIGCGGNTESKMAELRDDPLYVRCVHDSLKENILHHIEIVRLFNLFQDVTSMIFGVVIGVSMVNLLSVESLKFSFVYLVELMFVFGYCLMGTFLTHASEEIRTALYNCPWLNLNKSHKRTLRIFQFRTSINIELKGSGLFLINLQLFVQYVAAFAVSSFAATVD

>SfurOR132

MSFFVYVGGVSMYYARNDLTTFVEIAHAYTFVLAFWVLLTVHFVFKLPVLHELLQTVDGEIFEYKNKCCEEKEAKVKSQNRFYHLLFQTVLDSAVLVAVFILGFLAPVMIKLFEDENRRKVKEINYNFPVPLWFPFRTDNLLGFCFAYFLLMVEILLIGIYLLAAIPFLVFAALEVNTQYRILKNSILNLEPRALEKYNGMCRVSNNQLEILRQDSFFKKCVQECIKENILHHIEILRFFSLYQDLSSTIFGVIIGVSMIILASLSLVLTKTELLSFDTLKFAFFFIVELVVVFGYCLMGTFLTNSSLEIASALYNCPWYNLTEKHRRMLKVFQINSSISIELRGNGLFLIDLQLFVQSIPYLIEAHIFLKLD

>SfurOR76

MDEEANIQDYDKLKEVMFEIIDLLKLIQFYPPSQVGLRGTFLYLFMIILVFQPLAAILANWADWDFNSRVTAVDNLSFSIGVWALASDIFLMPEKSKLFFDVIDSKFALHREKATGDAIYREKCELIEEMTNEGHRLVRTTGSVVMYYFASQIVVPIFGLALYFLKSEPAQKLPLLFKFYNPLTLTLEVRTIFEYLLIIFLQLSFVCYAATFATVIHQMQMLSLHHMRVEMRLFHMNVKEIDFYYEMMIGEDGERGNKADENGENERIDKKEEKGERPTEEGLRIMVRKLVKQHQTIFRNVKDLNEGFKFRLFYLNVYICLQICLGIFIFIKGELLMKIKYGMILLSITVVEFLFSENGQKLQDEGEYLRTALYNCN

>SfurOR63

MVVRPFRLYVLDIGCIVWEKSSILTKVQVLKSGVTTQAPATKFFFTVSLIIQVDIILNLFTEWRDPISRLLSMKEFILLWNGILGCMSSKEGRKMLNYVRQHFEKYKVYEKTSSFEERKIFEDRDKKVLKYNRVVSFLLTYTLHITCGLVPYVMILISLLKSYQKGENFKNLPQILHLYFTKAFDKSFTFQLIYQTSIYAWYGLLLYYWTIVGKGVFFSCTCLVTEISLFCHEMEKIDEINDDEGEGKLRAKLRQIVNEHQSICRNIEDFGDISSKPLFGIINLYGSQICIYLAFITLIDDIHIKLKYFTRYFITFAFVYFSASNGQIITDMGRKLRLSLFQCSWIDKPVWFRKTLFFMNIRASKEMVIRPFGLYVCIDFFA*

>SfurOR57

MIFSALVFCPEQPWFTFNIIFISLTLSTMMFFAIIGSVSIYYARNNLVTVVEIAHAYSFFASLFLVLIFQYFKAPKMYQLLQIMDNDVFVYKTKHGREDDEKVRTSMRKYGLIFQTSFHISLVAAITLAQVAPLLNNMLGDGGRVKVKEINYNFVPFWFPFNTYTLTGFSVAFLLITTEICLVLFYVGIGLSFVVFIALEVAAQLRILHDSIVNIEPRAIERYRCWRELGKHNDMLGDDPLFQRCVRECLKENIQHHIEIIRFFKIFQSVISNMLGVSIVDAMSLLACLSLVLTKVEPFSIEAVKFTFFFILELVFAFGFCFMGTSITEASKTIPEAIYRSPWFRLLRGEEKRTLLIFQMNAARNFVLKGSGMFAIDLQLFVQ

>SfurOR134

MRSTRFLRVFTNFLSNDKPFGSLPSLFLIVLATCVISNFVLVVLLETEKVEKRIKALEECFIILSALTCVVRRIKNPKIVMQILDLIEGELLTEVGNDYRPCRSNKLEKIHRHYVKRRRTMENFFEKMSIISIGMLFGCIVRNAFGQTIANDENFKDWPTIFVYWCPPGYDSLAFFLVVEVLFILLFMFTMAEGFSLVLTTCLATERVLADFETIYMLLEDIIKDYAVIQSDSKSNQDDQFSRHMDLKRDMKILIKCHQRLISNFKVCADYTAFGSVMCTLIIYADTIASTYTMLKAYNLKMMVHFAAACIFSNLTILFCYHNGQKLVNQNDYLRQYLMEIPWTDKPQWFKTSLCFMMRRANVDIQLMPYNVYVINHESFKNTDK

>SfurOR31

MLDLIYTILHGEKIDRNQYNSFLKIMKLLLIVSGLRKQSDPEQLNTILIIINVILLVSGCITCLLSCLRFINDLNTFTEALMFFILFARVIGNYYSLGTNLRKINRFLWACETNFDEFEKKNLKEISNLKQISDHSVLTFVVVMVSLFVSMFVTYTSSFRFFPITSPWDNQIGSSVFLSNIALLYQIVVCFISSVVSLTDATMGLGSVNIICGETEILGRTLLATDGNPRLKLCVKQHCFLISLMYNIQSIFSLNIFLIFISCEFMLCLCSFQIYQMHATMTIASLGKNICMLCALLFHEFVYFFYGQQLVNKTADLHHDIYFSKWYKKPAQFKSSTSIIQYRTRKPFVIMAGKIIPVTLASYVACLQAAFSYFNLLKALSHNQE*

>SfurOR18

MLLLNLINYLKSSNTEKYECHINIMKVLFFMLGLWKSPDSREPNYKIVIAIAANLTVSCIFLLFALHHSVVNVDIEGMTEALMFFILFARIILSYFWLVKRISSINRLIEAAERNYDDFEERSIAHKTGLKSECDSWNLLFFKSLIWMFCILQLLYAYATGFYMPHSTEKKYMFPIRSPWKFEESGNLCYYLAISNQFASSALGCALGTLDVTFSVMSINILSAEVDILGEAWKQTTTYNQLRRNVRQHVLLLSLMNDVQSILSFIVLDMFVTCEFMLCLCSFQIYQIYSTTSFVSLMKNICMLAALIVHEFVYFFFGQRLVSKTMDLHRSVYFCEWYNETKSFKSSMNIVHIRMNKPFTILAGKIVPVTLASFITCLKAAFSYFNL

>SfurOR108

MPDKKSTRECVQIVLNTVSLVGFNHHKTKTSTRGKLLFIAVILIEINLILTMIKNWTDFEKRILAIEDLNVNTLALFMIGELIFFNQNISKYLGMIEKDCVRGWGEYDKYNEERNERENFVVINCRKALVIIVFNNAVFQLLLTFYFHLVQKAEVVDLPIPIELYFKDSISTFTIYTIVFLLECIMYSYYAFLAFCEYAMGFVVFEDILFEMRIFCVMVEEFEERNRKLRQSSEELRGFLRKLVVHHQKIMKKVSIGQKIFRSQVMYVCQGFCIQLCIGAYLIENEQMIVMKCKHILVLAALTVVLFLFANGAQNLLDMNNELTSALYNLQWHEKHHWFQKDLKTLMMQVCIEPTIKFFGVVTVDRSSVSSVFKGAYSYLNILHSLAEFK*

>SfurOR116

MENTKFMKNLVEWQRYDKHNSIPFLLAIVLTVITISGMVSAAVLVENEDMKIAASKDVVMLSLVFIIIFKNCVNPENVMAMLNLLEEECSVNVGDFEGREELWKKIKIAYERNIEGMILIDKYIRITFVIMFSAFIIKNAVAYLLRISIYSDSQSLPWVTPFLWFTESEFKASAYFFSIYFLQVLVLGIGHIHGYLVISSVTLSIEKSLADFNSIYIFLENFDIYFPVASIENREQHWLVENENMYSLKKSMAKIVQCHQRICRNHKICARKSAFILTCTVCATMTDSCNNAFFVLKAQGIQKAVTFVIMFILLNLVIFFMFQNGQKIMNQNDVLRRHLAEIPWVDKPRWFRQTVHTMMTRANVDIEMKPYGIYVLNYLSFKDMMKLFFSVGNV

>SfurOR98

MKKRRDQESSAASEKKYRIPNIPILTSDLSPTPAYGFFYLVVLIMQVDLTINIYSRWHTVISRLLTLKEFLLSWCGLLGYLASNDSKNMLQNVHNHYEKYKNGENDNFTNEHMEILKERDKQILKYNKVIIFMLKYILHIMCGLVPYVFILISIYKCYKKGENYGKIPEIIHSYFPKAFKNSMLFQLVYHTSIYVWYSLLLYNWSIITRSMLFNFICLDTEIKLLSLTLKEIGEWRGEGRRKLEERLRKIVQQHQSICRGLMDLEKNSSKVVFGFVNLYGSQICIYLIFITLIDDLHTKVRYVSRYLITFTFVYFTCDSGQTITDLGRKLRVAFYDCSWVDKPYWFRRTIFFMITRASREMVVRPFRLYVLDIGCIVWIVKGTYTYLNFAKKLVD*

>SfurOR69

MTTTSSFLVRFNDLMRYKRNKPHKSLPLITFIIIFLYVQLDIIVTLAIEPRDLEQTVVTLTDSVMISILFCHSVLRKLKTHNVDNLMQLIRSEFPFNSNDLLVDDRLEIEQFYRKEKKYGTTMTKNLARIFFVYNLLVINRNIFNYLTGLTHEDYNIWPTPYISRSPPFLKSPLFFLYIYFLHALLISMFMLDVLFVIMLVFLSTARIVFDFETLHILLRQLSKNYVEIDEKSIQAMKNLRRDMARIIELHQTLIKNFKVCVEGSGLMISVINLVIMIYCCTIAYLTLTTNNMKKRVNFGMSFFLVSSSLFSIYHNGQRIYNQNVILRENLEKLPWIDKPKWFKQSLHIMMTKANDDLQMKPFGIYTLSYMAFTDWIKFVFSTGNLFYSKRTSQA*

>SfurOR130

MTKSSEIHELDWNLIYDGLEKTNLSYIPVSGLIIYSSGKYRTLSLFIISVMIFLLCFICFILSITVFKSLDDFPVFVETAHAFSLCMLVLKTLIVHHLRINSYLSLLTTFSTNIFNYESEFARQKTVEMRVKVNKHIKLFQNMIVFICLSAGFLFLVSPIIQAKYMNKDRDEIPTLNYNYPIPLWFPFDTSNVWSFRFAFGYCLVVCTMAVLIILIVTFVPSIVLFFMEMYAQLEILNHSIINLDKRAALLFNSSNNIFGNSSSRMEMDTSSLIENGDYQRCLLYCMKKNVQHHWAIKRSELTSRNGLQIEAESLKTKIISLLKVEKFRDTFQHFSGIFLGDVLVGSGLMIVTLSLTLTKSDTALSPDNIKLLFLLVCELLNLFGYCFCGTLVKDK

>SfurOR25

MVRAGKLSTEEESLLEIFSDKRISRKMTVLFFALTWSVQINLIFCLVYEWANLLNRILVMKEILLSLFAINVYFIRNDMLVLENKLIMRYIKNMNQHNTSKERDRLITEQSQFMKWFGGVASVRVFKFIFIFGACLPMLQVSIALARAYFTGKLEKVPSVIFVYVPQVFRTIPGYLIIHLLCFTWSSFITAICAVIYKVYLTSMKCVETEMDLLCQSMFEIGPMLANDYELNTKEEQRSEKKELRHLFSVIIQHHQHILRSIKLLSKHLKLMIITLLNVYGLQTCLYIILTLKMEQVGLRIKNASLYMMSMLIIYTCTKTGQELKDKGDKLRMALYDCSWIDKPVWFKRSLLVMMSRAIQATEFRPFGLYSFDMNCMANVLKATYTYFNAANEFLK*

>SfurOR93

MDDIIKVRIMNSTGTGFLKTYFTLMRLDKRSLNNSSPFLLFLALLAVTLAGVFEGFLQESDGDKMSATVSTIMVLTIMISSKIRHCVSPQQFEELMRLIEEDCFVTRSDFSERLGTHWEKVHQLHRGTVENMEFVHHYLRLVTSIGTTGFLSKNLVPYLLNMPLFVMDERSDPWLTPFLVHRPSEFEPLTFFTYVFTLQSLIMILVCVEGYFLGLVICLSTERMLGDFEIFYQLVELFCQDYSAELCVEKQAGEPNQFHQMKSDLTRIVKLHQRITRNHRTFSDNSGYLLSTAIFAIITDSSMSAYFMLKSTEVRKAAIFGITFLMINMFLFLVYRNGQRISNQNELVRKSLAALPWIDKPRWLRQNVHIMMTKSNVDIEMKPFGIYVLNYMSFKDLM

>SfurOR45

MENDIEIKLKSKLKKISEICKNYIGFQRDGISFILFYGAAINIFAFCALSGVKAVQLFSSGNDETLISNECLNAVVVFFSLLSAEIKLILYFKNRRKAYKLYSVICKTLPLNSNDMEREGEETIHIAVMILIWSSAAATLFALLNSNVLSFNRGETILLFQIYLPKSIERNAFTFYAVYGLQLLMCTHVVAINGSTLASFAHASNVLCVQNKVLGIAICNLTLDDGGRITDVKLTFGHRRQEKMREYVEFHLHLINIVSQLESVFGGLIFWEFVGSQCLSCLLTYLSIVEANKGRIFSSIRTIFMIPALLITSFIYFFLGSKISEASEELRKLLYSSYWYDQPLWFQKCLLIMMKRMQKVLTFNSVHIIPVTLNSFLRALQAGFSYFNFLNAMTGRYK*

>SfurOR42

MRGFSNPEDCFRWVCGFLGRPYPAGKIHFRFYIIVILYIMNFLQLLLGMRPRWNNFQARYQSIEDLIVLAFMTIIVCEMGWSFNKIVVMVEIAKSNLYPASQPLSDRQKSIIEKMNDEILGIIKTFAFIVAGFSLINGFIPFLHGIFFVSRKDINNVSQEQIPMPVECWIPYNIEDVITYIIFVFPQVIMFAMCSFVGFIWFTPFIISLFHLMKEIDILCHSLNDMDYWLEEKTGSDYDFHLQKYFKGVIEHHQNICRTVQQLNEALSVFLFLFNCVCCAQICVSLYSCFETSDSVSQMKYILLLGPVFSCFYFFCWSGQELINRNDQLEKVIANCSWMNKPKWFRSSLRIMILRSSKPLQIKPFGLYTLNFNNIMMVCRAAYSFFNFMHKVQMKSVY*

>SfurOR78

MDNTVFLRKFIELFRCDEPRKSVPLLLHALLVFHNIHNTVKAIAVEDDIVTAFTAIKDLSGHTLMLTFSINHGLNSIKTLNLLELIESGTIVKEEEFHGRMNKWERIKIIDDNKIIEMRSKYKYLSLVMALFYVGFVTKNAVNYILIDIPISNHPSNSYPTPFLSHIPLEQQSLISLLYMFTLHSMTTLLECLQALMIISSTCLSTESILSHYETIYTLIEEIENDFSDAEIFNDDAGNMEILKEDMGRIVRCHQVINRNCKICLENASFGILIITVTITLDSCANAFFMLKSEDIGKALVFCIMFMLINSCLLFVFHNGQRIFDQNDKLRKYLNNIPWTNKPIWFRKSVNLMVTRANKDNMMNPYGLFILNYQSFKDLMKTVFSMGNVFYSIKESRF*

>SfurOR23

MESSNKPANTASRKYTTLTLPPKIVSGIYLILTILLQLNLMLSVHSGWNDFIQRLITIKDMILTCFCYMSFARVIGRDTTLLCEYVEEHISSVNIGKNRIEGQNLILLNRERKLETTHKIVKRYYYLLVGICGLLPYVQSLKVLIRADYLERKPKVAFLLNVHYPEEYRSFFMDLIVQTFLLVWYCVFIYYWISDLKAMSLSFQCITTEMELLRNSIDEMDTIIENHHLAINKQKNMDSDLFNKIILHSYLSGIVKSHQNICSRMNFFNINLKNLVVFLMNVYALQLCLFLIFVIELPDIFSKVRYLLRYSIILSLVFFVSVDGEKVTSEGEKLRRALWMCSWTDKPNWLKMSLLTMMTRATVDLKIQPFTLYSLDLTCFTQIVKGTYSYFNIYKSLKK*

>SfurOR107

MDSMKVEDLSSLPLSEFGNKLPELLSTLYLLFTCCMQIDLIVAMNIQWDDFILRILSIKEFNTTLFAMVGYLSMNFEVAHLDRMIYLHFRRLNGENMKGNRIIESRDTKKKDFERKASRIFVAIFLFGSILPYVHGVILAVKNGTENLNFEKLPYIVYTHYPENTRTVTIYLTILTTQCVWYGLMTLQWYFLYKSFFVSSLCLMTEMELLIKALNNLDCGSAAIKEKNRDEYMDLNRIENAKNQSLGERMDRIIKDHDIMCRGLETLNTGIGFMYVGFYNVFALQMCLYLLFVIEMNEMVARIKYTATGLIVFSLSISGSKIGQDVADQGERLRLAIYQSAWIDKPVWLQKYILLMLTRASCNMELKPLFGLFILNMSSVTKVVKGTYTYFNLVTSLRV*

>SfurOR8

MDQTITGASLVSCFGEPRIVGFKGALFFVLTVLISVDLGFCLVYEWHDFIKRLLTLKEIALVIFCTGGYFAHLNMASVENEYIFKYLKYVDEPSHSHSPKKLELINEMNVFIKWLSAFSTRAFKFLYIFAAVIPLTHILIVLTKAHFSHKKLDEVNIPSVIHFYVPMKLRTASGYFLTNVAASTWYAFCLYIWSIVFKLFVIGIGCLCTEMELIFDDVNEIDHIPPAAETNPSKPIEGTMHVNEILLKRKFNSIIEHHQDITKSMKLMNRNFQTTIVLFLNVYCLQLCLYLVFIMKLEDIVQRIKFLALYIFALYLQYQYAACSQRIKDRGETFGETLYECGWVDKPQWMKKMLLIMKCMADQPMVMKPYGWYVVDRVFMANIFKATYTYLNVVNEFLK*

>SfurOR131

MREAMQLRGHKEGEKSSFLSKFYIFSTVVYILNTILDIFNLEAGQFEEKIFRFKNINMLLCGLAYPLVDSDTFRLLFSIEQQGSFETSLRSSDAIKVLQQKLDKELNQINKLTYFVIVFIMGTLSIAPLMAAILKTYPKLLTGSWDTINVTKFSLPISFWYPQEYANIYTYILLYTLQIFYIYLFSGYMYCVLTSGFMALKITIYNLKYLCVTVEEGRNRLEEVNKNFGNGELSYEDRSHLERMLSLRVYLVNIIKLHDMICRSAGNLNRDLERMYTVFNNTICFQICVCLYSSVKMEDFVLKVEHILLVIPLAYILFLYCSNAQELLNEGENFRMTLWGSFFVDTPKWYKSSLLIMMIRTTKELEIKPFGFYVLNLKTFSVVMKAAYSYFNMLNSLKKRI*

>SfurOR9

MEDRVSSESVISIFGNKRVTGLEAFIFLSLSLLILLNLIFCLIEEWSDFFARLLTLKEIMLILFSAIGGYVVQFHMRDVEENYILKYLRNTKNFETTEEKRKIIDGTNNYIVRHNVVTLRAFKFLFISGGCLPLLQITIKTLKQYLTGIEAEKMAFVIHLYLPEGYKTPLIYLLTQVAAFILYGLLVYMWGVNYKVFLIGLKCISTEVCLLVESLKELESFEGKSAPAELAENEEQMLAVSGFEERKLREHLGRTIEQHQDVLQSLKLVNDSSKLYIFLFINVYSIQLGLYIIFVLKLKELAQKMKYVFTYMVILFIQMQWAIYAQDIQDNGEKLRMALYNCSWLDKPQWMKKSLVLMLMKANKPLELRPFGLYALNKSCMANMMKATYTYVNVVNELLSRK*

>SfurOR90

MEKTQFIRKYTDFVRYDKLWNSLPCLLIMLIGILGALNSLIVVITDQQSFEKRVLGVKDLLTTFLLILNCVERSFKPHKNIKLVELIENDFLVNHHEFIQRGKKWETFDKYYNETKMKMQKTSQNLLIVFSIMYVGYMNRNIFNKLLRDSIGLERKSMDLPVTYVYWSPDASGSFNSFGFFAYIYIFQCLMILIFALETYCIQISVSLSSERLLADFGTIYILLDDLAKDFPEITGTSHQHDNDNNKNLEKDINRIVRCHQSLIRNFKEAETNAGLVIIVISLVVFCDTCIVFYLILEADHLLTLITYAISNTLINLAILFPYHTGQRIANQNDILRQCLANVPWIDKPKWFKKTLHIMMMRANIDIVMQPFGVHYLDHMSFKNIIKAAYSYGNFLYSTNSK*

>SfurOR128

MRKFLMQRGHFERKSMRVLAKFYLMLTVVLIGNTLQDIYYLQADQFEEKVFRFKNINMLICGLAYPLLDKSTYRLISYIEAHGKSDATGNNNSSKSRLQLQNKMNKDLNRVNKLTYTVVVSIMGTLSVAPLLAAIFKIYLQISSKTPVDFRSMSLPISFWYPEEYASFFTYFVLYLLQMFYFYLFAGYVYCDLTSAFMALKITIYDLRFLCLSVKEWDEDNGGLINKEEVLKYTEYRNESLEEEMVTRYRSDIVNVIRLHDKICRRASSMNKDFEMIYTIYNNTICFQICVCLYSSVKMDDLVLKIQNILLIIPLAGILFLYCFYAQEMLNEGERFRSTLWESSFVDKPRWYRSSMLIIMTRNAKELEMKPFGFYVLNLRTFSMVMKAAYSYFNMLNSLKKRI*

>SfurOR80

MVDEKMSSQTKVDKISSSLKTARNLRLPVWYRWHAQAWKFLTVVLQLDLIYNMHQMWNHFVLRLLTIKELNSTFFGIMGYIGMYDSADLFTYVESHVARMDERHRAGDTKCSTTKQRILDDRDTQLKRSAFAVSFLFGTLFFLCALVPYAQTFQKIVQAYLHDVTLGKDVSMIVYTYYPESFQTIPLYTLFHNLLFFWYSSIVYLWAMDLRAVLMAFECLNAECELLVASIEEDLASSAGEQLRRNDLELRRRLRLIVIDHQEVCRNTRILDKYSRNILMGFMNVYGVQLCLILIFIMEIEEAGPRLRYLFRYCMVLSLMYVVSSQGQSVTDRGFAVRTALANSPWIDKPKWFKETLLIMLTRTTRELLIKPYGLYTINMNCIANIVKGSYTYMNLVNNFRKKN*

>SfurOR82

MIWMRSTSLMRQIIHLLRFDRPYNPLPFLLVTICHLLTLSNILIVFLNKAEHLETYVIAAKDFTLITSIFSVTVKRFLNAPESVRITNIIEEEHVIDCYQPDYNERKWRRVKKHYNKQKKNMQLSGSFVIGLCFFTFIGIICRFPVLYSLDPDLKKYRGKPTAFISYIPGDYSQYTDFYIYIFVLHCLIILFQVVEGYLVHIALLQCIQKLLTDFEVIYILLDELANEYADFDCVTDKQLPDSNAKLRKDLRLLVQCHQNLNWTLNYCVKVFGYGIFVYSSMIALDMCLNAYFILQAKNVKIIVNQVLSCITYSIIMLLCYYISQCISNQNEIFRMNLAKVPWIGKPRWFKQTLIIMKIRANADSEIKPLGLYVLNLSSYKSIMKGAFSFANIFYTTKFSQNNS*

>SfurOR127

MGRGYNCFLALIHNWPDFFKRILIMRDMNVLLSMYTMYANFALRPETILNLIKIIDSDFILLNFHNQTEKQTVIHEKKTRLSNLEKNILNGNIFCITGMTVMSLITSGYIYFLTDQPVVTDLQLPYPFYFPKGYENIRVYLLAYAIDVVITVFLVFATSYCYFSTTIMAIERVGIDFELLKLSMEKLNEEFSRMKQEEEEEEEEEEEEGMNKRGKRRERNTIERCLILILNYHQNLLRKSKELQKNSQFVLLVAYQLTCFFDVGNFYCFYKTNDLKLRVLLVIITVLANLLLFYTCDSGQRMIDATNDLKLRVLLVIITVLANLLLFYTCDSGQRMIDASEQARETMYFVWQNKPKNIVKDLFTMMIRANRDVHVQPFGLYTLQHKTFANLSAMSSKSEPVFEKPL

>SfurOR133

MMSSNNNKKYDKLKKALNDIIGNLRLLQFYPPDEIAIRGRLLYITILLLLIQPAIALLENWTKWNFHSRVSTLDNLTYSLGLFCLSSDLFFMPQKTKILLDVINSDFSIYSEEIRSYKREIYIKKCKLIEDMTQEGYNMIKIIANILQYFFAAHVTIPIYGICFYLIKSEDVEKLPLLIKMYSPLTLSLEMHSIQEYLMMSVLQLTYMYVSLIFVIILLHMQILSIFHIRVEMKLFHMSMELINKYCSEMTPIVDGGLQYVPESEMKFMMRELARHHQTIFKKVEEVNSGFKFRLFYFNAYFCLQFCLGIFIFLKGQALLKLKYGIILVTIITVAFLFSEDGQKFEDEGEEIRSILYNCNWQNKPKWFTSTLQILMARNNKLPKIALLHVFTLNRTNLTVIVREHY

>SfurOR79

MDATNFLRRYIDFMRFDKPQKSLPFILFTLMEILIEINTFTAILTESEYSVKIFLALQDLFIRFVLFCNMIKRNLNRREDFKKLLHVIEDDFIVSRDEFDGTEKLWNTLENYNIKKRKFMEMIFRNLAAVFAVTYIMCLSRNATNIFIGFTQHSENKSINSLTPFFSYCPPEFDSLVFILYLCFLDALLMGAYIMEMFITFSLVCLSTERVLADFDTLHFLFNDLSMYAELNSNHQVNKSISHTKYAMNLRKNMGCIVRCHQRLIRNFQMCSEVSDLLVSIAVLLVMLYSCTVAYMALMVDDLQKRLMFVIFFIVVNMTAFLSFHNGQRIFNQNDILRQTLVELPWTDKPQWFKQTLLMMMIRANLDIEMKPYGIFTLNYMAFTDVMKTTFSIGNLLYSKKQLTGS*

>SfurOR100

MSNAISKFLSYLQSTGLHSTDKFILKGKLSFFIVSIAVLEIIVATLFCWKQWDINVRVTALENLNMAIGILEIAIECQLMPEKIESQLTLLSSGVYLYDLTLEGIKLSIFKEKENFVRGLEAKIISLTEIIETSMHWFFIGYFMSAFCGIIVYLFNQDLNALTLPIVFFNPFSHSPSVSFMDFNQYCIVLSLELWYFYVSYKLTLSMGGFLFLSTDNVIREIKLFQMNLHELNLNLDAIEAEYKYFPQNYERFEKLKPVFRKICEHHQRIFRKVKMLNKGIDFIVICYNPYICFQLCLAIFCIMKVDFQFKIKYGFAFITVLVIAFKYCELGQELENEGEKLRLSLFNSSWAGKPSWFGRSLLIMMIQSHRIPKIEAFRILTLNRINLRLIVQTVYSYLNLLTRVSS*

>SfurOR5

MNIDEQHTPSHLELKESVWKRTQFTLFPKITVTEVIIAVIVTYFATQTALLLILHWDAYDINSRPEVFANLSYNLFVFLVTMEVFFINGRLRIFMQIIDSEFKISGELVEKKRMKIDELEETSLKNSKALSKVLLFFSIGYSFNCIHGILMCVYKLRPIDELPITFSFLLPANFTKTKESGYFYVCIALLQVWYLYFGYIIVSVLYTSRLFAYNSIFTEIELFLITLEELNHFRFENEEESNFESRVIEQSDVVRLKEIIRTLGKHHQVIFKKISMFEEGGRYHIFYVNASICALLCLAIFCTQRVTAHNLKVRYALLALSMIAGCMIYSENGQRLMNRGEDSRKAIYECSWIDKPIWVQKSLLIMMMRNTKDFDINFYGIFKSNRSNVSSLLQASYSYFSFLNNTN*

>SfurOR92

MECTRALRKYIDFIRYDKPSNSIALLFIITVGLLEFLNNCMVIFTEHKNKEKFMMGIKDLFMISAAFSIILETSFNSQKAHKLLDLIEKECLVSRYRFAGIGEKFRKIEDSYNKKKEDMEKMYNNIFYVVVVVYLGAINRNLLSCLFEESSELGITRDWPTPFVYWCPSGYNTFTFFLFMYSFQSLMLFMCTISGGSVEIIVYLASEKILADFETIVLLLDRMETDFPDFDFEGNSKSLQKQQMKSLLKMNEELKRDMITIVQCHQALNRNFKDCAEIPAYGILMNTFIISLDTIFNVYLMLKAHDLITSVNYAAASFFINLIGFLRFHTGHGIVNQNDVFRQSLVNLPWTNKPQWFKQMLIIMMGRANIDTEIKPYNIFVLNHKTFKNIMTSVFTYANFFYTRHSMS*

>SfurOR95

MADSTTDWFWKSATSEVVTRIFELMGRKLPKDNDQDVGNISFRFVLLLLINTTGITNSIILTYRFWTTDYKFDFILNSVVIYFQFCAIIHLIYFPYHYIFLFKVLDRKLYPTDKNFPLNEEQIELIRETNRREKKVHIMFLLFVLAWSLKSLLTMAKGVHELYSKEFDAIEEYEILMVYITIPIQKIFSNRIPAILVYMILSIIQVLIFIFTLEMSANFCFLVTCVVKKLEAEIWILRQYIRSIDKVISKDNNSQFYEISLKRYLNHCIKYHQAILHSIRNVNSAISFMMFPFNNVIAVIVGMKLYFLLESTGSLYNITDVGSAFFTFAIICFMGQGLTDESERLFHDFAMCNWIEKPHWFKKSVTICMTCSIRLVGLKTALYTINLTNVVKVLQATYSYANLMWKLK*

>SfurOR35

MYQPFNILKILMSFMCIHPPKFCTTSELKLSMYNGLTGLAVAIEFLSYIFYFPSTFFMYKSGSELTPFQAFIALYGISMMMQIFMMITTRSELMDIIKSIHDKYAADGAHAEVENEQSRKRSLWLFSAITVLQVPTFLGGIINSEYIGNNEDSIKANIHFGRKNPERLIAYLPWLPYDYTISPYFEVTKTLMYIALTMGCISMMVRYSLLPMLTFYINGQFDMIAKRFESLKATDEKPGSNGRKLIEEEILNEVIRSHQQALGLSKRVIVYFRPLIVTKTIFYFGILADLLYAVTEQTAGTAQQINCCFCLMSCGYELLLDCWCGEYIARKSQLVGLAAYKSEWHNMSFNVRKSLAIVINRAQKPEQLNGRTGFIPMSLETYIQIFKASYSYYTVLQNLNKADQQVQI*

>SfurOR91

MDSLIKLTKFMKNLIKLLQFDKLHNSIPFLLEMITASIVMCGMLMAAKLVKDEDIKIAALKDILVVSFVLLHMIEISVHPKKTMAMLDLLEEECSVNEGDFKGREELWNKIDKAYKRNIEGMILLHKLIGITLFLCFVGFLNRNAIPYFFRIPIYTDVQFSSVITPFLWFSGLEIDFSPYFFSAYLSQALLLGLGHLKGYLLISAVSLSIEKILADFNTIYIFLQAFGKYFPDDSINNSDENSWRKTENMNSLRNSMAKVVQCHQRICRNYKICAEKSAYMITCLLLGVMCDSCSNAFFILKAQNNVQKAVSFVFMFIFTNLVLFFLYQNGQKIMHQNDVLRIHLAELPWTDKPRWFRQTVHIMMTRANVDFEMKPYGIYYLNYNSFKDLMKFFFSVGNVLARKMANQS*

>SfurOR74

MEDTNFLRKYINLFKYDKPYNSIPFYALLTLAGVSLSNICLSTKTEETVEISLEALKDAFSAFVLYLCIVEHSLNPNRALQLVGLIEDEFLVEKKDFVGKGMEKWEKFDKLHKKKKQDLRNKFRYITIAQYFICFGYIFKNFLNYLLGIRVYDRYRWPTPFLYPIPESHFQSTIFLIYVCFLHALTLTTVCSEGYVMLTTVCVSTERVLGDFETLYLLLDNFSKDFSDSNEGKCIEGLFVAQETGNEFENREDTLKKDMRRIVKCHQNINRNLKICANNSAFVVSAAIAVIVADSCSNAFFMLKTTDAKKAIISTSMFIIENFTMFFLYQNGQRIYNQNQVLRKYLTELPWTDKPQWFRKTLHLMMTRANVDAEMRPYGIFILNYVSFKDVMKLTFSVGNVLYSMKVSN*

>SfurOR71

MQDEQRSLEKVVSTIVSSLELLNFQPPSRTATLWARILHSMAIYIVCQSVAVIFFGWNIWDFGTRIAALESFNYLFGLYTTSADLFHLSNRNQQIIIMLQSRYYSPINCYSEKGCKKRSEMIKTMDKTSVEKAALLHKIMRSIVTGYILFPAVSIVLYFLNLLKLEDLRLPMITFIPKIYPISLTFSSINNYLLSFCYNLFCLLYSYYINLSAFKLLVLSVDNVLNEMKLFRLNFEELDLVLDEEEERGGVEKDERLRRLMKHVVRHHQMIFRKVGILNNGLKYRLFIFNAFNCVQTCLSIFSFMKGEFTFQKKLGVAIFAFCVNCYCLYISNMGQKLEDEGERIRNTLIYGSKSMRKPNWMNKMLIILMIRSNNLPKLDLFNVFTLNRDNFKVIVQGAYSYFNILKNV*

>SfurOR21

MKKIIEITDVVETILRYLKRPGLYPPSKSGVKGKITYGIVVYVVLEILFVMALCWREWDFNARVTALENLNLAVGILNLATEPKLLHDRTAYQVRLMSSKPMLYNLSSSSKQLISIYEEKEAYAREMENQSILAKIIGTSVRCFFVGYTLSSLFGVVVYLIDSDFNRLSLPMIFYNPFTPSPAISFSTFTQYLLILPVELWYIHLSFTLAFVLNESSFLAVDNVVREMKLFQMNLHELNKNADAIEKQANATEDYNRLKVIVRQISIHHQLIYKKVEMLNKGFDFQVMYNNTFICFHLCLAIFCMVKVDLLYKIKYGVSIFAITIISFMYSENGQRLENEGENLRLALYSCSWIGKPAWFCRSLLILVTQNNRTPKLETFKIFTLNRNNLKVVMQAAYSYFSVLIRFSR*

>AgosOR9

MSIAVMVHYMAEMFFKKAICSDDDLHRREAMRMVFFTYGELAITLFFAVSTYLSIVHSTEDLSVHLYGVLCLIIQLLVFAFLSFRSYHRSHFRDMYQRSRGMEISENSNRKIAAVIKHHLIMPNVVHIGDPFTFPFMDVLPIETTSVAVYVCKYVVYALPVYLTQIEVCFLNVTYIVIFRLLEKQVEEAMVNKDEHKLKIAIKHHQELLKFFKEMKTVYEKPIFLIIVSCGLYIGLTHDRILNALFQHQLLYSQNKSFKQLILIMMTRATIPLEFKAGSIFTVNMNLLVRILKFAYTVFNVLITSINHQLIKTAV

>AgosOR33

MSSFEVSNVAINIKLYKLLRFYHLFDPNNIFGYHFYRFTGIFITVFIQLFVLFGLLGCFMEMEDTINDIEQFIFIFVNLSNFLSVMKLCVFTYKAKNTWDLFDVTCIHFLKSEKCCKYRNEILEKVRNKSIKLTNFIFYRKLKLYYSIIWYVILTYVVLSSCSIITLTYSFIMVITCISSTKSLPVLSIIKIIAPFSIVSFQIFLHCYFFGLINFKKASVSYGMYSCNWTSMDLKFKKLLLLSMQMNDADKLMIKATPTRIINLELFVKVDNNLLLSEAIYK

>AgosOR10

MEHIVDMFLKKTGCNDDRSYDTMCQVFFIYFELAITLFFAVSSCLSIVNSTEGLSVRLYGLLCFLIESHIFFFIAVRLYYLPQFRDMYQRTLKMGIPENFRQRIAMVIKHHFIISNVFVSIFMLYTISMDWVQMGDPFTFPFIDVLPIKTTNLTIYVCKYILYTLPVYIAHFETCFLNVTFMYSTGVMKRYFQILDGQVEEAMVNKDEQQLKIAIKHHQEVLRFFNDMKTAYEKPILMTIEFCGLYVGLTSYFSILVIQGYIHQIILGLCIVSSIASLITIIIYCIYASNMYDLHDGILSALFEHRSVYSRDNSLKRLISIMMTRATIPLEFKAASIFTIHLNLLIKILKCVYTVFNVLLTSISRKLKETAV

>AgosOR39

MNSENIFNGRPVALNLSTYKQLGYYQLLDPKGPYLYGCHFYRTILKIFLLVIEDSDANNGKSNSFELIIILTNCTLSSLKIYTLISNTKIIWDLFDLTCIDFLKCSRHSELITENFVTRCKKSTEITKWIARSFLIGLILWVMGPFIANEEYTAPNASHRYQNIINIKFPVTVKTYNNYYLVFYLMEVAVGFCIVYGSILVDAFLMSFCWIISAQYQSVTKAFATFGHKNELSSPEEIYKDFKSIIIDHQNVYLKMKSFYAVVRPITLIHVFAYSCSLIMYAYVIVTIFHSKESFIIAEIMKIVMTVSNVTIEVFIFCYLFELIDNKKEDVNFGLYSCNWTGMDIKFKRLLLMSMKMNNANRLKLKATPDVTINRPFFANV

>AgosOR6

MDVREENKHVFNIWLAKRVGLYQMFDPGTARYRGKNVYHIALTFIVLYLGVIATMMNVSGVYYWKDNMPISIDYFWKAETWLFVFFKMWIVVYRSTDIWDCLSITRYGFTSFGYRNTRTLDRWRERSVRFTTAVTVIYLTSLVFYIAGSLAFREDVILVKNHDGSVGYYHQNVMNFYFVVSDSTYNAHYNTFFFVEAATAVLLTMLFLIFDILLITMCFATCCQMQLVGCEFESFGHDKPLGDDPRRSPIGEHEFSDYTDERKNVFKERVSMYYDELKTIVLDHQAKYENLLSLFELAMLLQIFVSSITLIILWFIFIMSFSNDDRFIVSDIIVKKMIFLIPSLSYQIYMECYLFGLLHNQKDSVIFALYSSNWTEMCMRCKKLILLTMEMNNANHIKLKFTRTKIVNLEMFFKTMSDCYTITSVLINHIKTKNK

>AgosOR20

MAHSSSTTVVDVSLFKTIGLHQLLCPVNRGGYSVRSRRALMAHSSSTTVVDVSLFKTIGLHQLLCPVNRGGYSVRSRRALMTALGLSFALHAFQVPWLYCALNDLQRFAYMAAVIIYGMMCAFKGYVLVTNSDRLWWVLDAAGYGYTGCGGRDPSALRRCRVTLSALLRSFVALSYATLIVWIVLPFFVDEYTPITNLDGTVTRYRTTIHNMQFPVPLSVYNSRPVWTLIYVTEVSVCIVNVFIWSIFDCYLVTMCFVLNAQFRTMSTGYVTLGRRRVKPLPQDTPVKGVRIKFNDVKSNHYDDLIGHIEDNRKLIKAFDVFFDVVRPVVLVQIGNGSYSVISLIFLTSLMYLMGVPVLSAPFLKFICGVISLTLELFIFCYGFNHIETAKSNINFGLYSSNWTEMDLKFKKTLLLAMKMNSSHKRVMKVSPKSSVGLEMFARVMNMSYSIVSVLLNSRS

>AgosOR22

IGMYQLLYPAECGLNDGGHGYRTAVLAAMGLVLGLQSMQVCRLYLARHDIQMFANMGMLVVYGFMCLLKGHTTATNASRICVTLDAARYAFTGCGGRDPSVMRRCRATLSTILRTFVYDDMPTVWAVVYVVESIIFTVNVFLWTSFDCYLVTMCFVFDMCFVFDALFRTMSAGYEKLG

>AgosOR21

MYQLLRPVECGLDVGRCRSAALAVVFMTLGLQSMQVARLYLARHDFQMFANMGVLVVNGLMCLLKGYMVVANADRMCSTLDATRYAFTGCGGRDPSVLRRCRATLSTILRTFVALSFGVWAVVYVVESTILTVNVFCWTSFDCMDQWYTKHKLRLFRTMSTGYETLGRSRSGDVKLSARQQFDTVAAGAAISKTSITDDNLDDLKSHIIDNKNIIEQYDAFFDVVRPMVLIQIADGSYSIITLIFLTSLVYLKGYSIVSAPILKFVCGLASLTIELYIYCYGFNHIEDGKSTVNFGLYSSNWTEMGF

>AgosOR32

MATFNEHTAAIGLKVLKQFGFYQMFESNTKKIFGWNVYQFSYIILLMINQCLIVFGNSGFLFELDDTINNINLLLIIFSNSFNYLTVYKVIILILNKSKIQQVLDVTDLKFLKSKQCRDNKVKLYFSMMKPIVLMHVSINAGLIMMLSTSFCMVLLSTESFTQAFVNLFKIGIGIVYLSLQLFLYCHLFDNIHLNIQSVNLGIYSCNWTNMDLKFKKLLLLTMQMNKANEIMMKASMKKIINLQLFASVLTTSYNIVSVMVKTIGK

>AgosOR5

MPRIDAINVFLQMTGCTDSKRMLYLTYFEFLITLYYFIASYVSIIYYEQSVSIQLFTLLCMLIESYILLNITFRIYHKNQFREMDQYSKQLGIPDDYQSKINIITMYHLIASNMFVIFPVYFNTMKIVFTKPILQSMSFNAIYFGLTTTLVIQAIRGYINQTIVSICIASGIAAIINITIYTFYGSVLLDLHDEILRVLFDNSFFYVNKSFKRSILIMMLSYTIIKMILSSEAIKPHKMS

>AgosOR2

PGDTTQQQPESFVLTPFQKFCIRWSVFFDSTSDRLSRIETVLRSVQLSTIMITSVLTMTSVLIADNKKALESFTYFVICVFLLAIITFAIRTKRFNRAMLLMVVDEFPGYERPMPDDLKRKISAIRKSYGEFTMKVMVSYLTLVLFEIPATAMVPLTAARLTDVKLGSQSTQMVVLWFPGDTTQFLIVMIVKFIITGIMCSFSFFVSQMISEFQILSAYVEHAVEIVEYDLSTGKTTDQKLLDHTTDQKLLDHVKSCVMLHHRLIDFKDQLNESYGYIILLELMFSTLYFCLSAFNMIFVGNRFVIAKGLLTLSNYLAELFIFCMYGSMVEEAHMGLLRASYSAAWYSQPVRFRRSLMMVMSRTQTPLQLTIGKVFIANLPLFLSVLKVSYSGVNALRAANAK

>AgosOR25

MATTTKMVYKNEDNLMINTRLMKITGLYQLLDSRTSKIFGQNMLKCMSLFQLSIMFITVVIFLANTYYFSDDINAVMQYSILFVCDVLSILKLYVTIVKSDTIWNCIQMTSIDDLSYKYHNRSILRNGQLKSKSYSILIMFMWMNLIILWALAPLFVTNYFLEVEVKKKIYRYRFNIMNFVFPATDQFYNDNFVIYYCIEFACLIIWCHCTMNFDVLLLSTNITFKYQLKTIANSFSAFNITHYIKNNFTKNIKHYKESELIFDFKSIIYDQQRVIENMRNIYRIFQPVVLTQLAFESIIIILLSCIIMMNYFNGISLLSAMNLRLFAAVLTFTFHIYVICYLFDNVNQDRKSVV

>AgosOR30

MVTTTKMTTKNEDNLMINTGLMKITGLYHVLDSRSLKIFGHNVFKCLSVVQMSNLILLTIIFPANIYYFSNDINVVMQYLMLLTSDMISILKLYVTIVNSDTIWNCIQMTSIDNLSYKYHDRRMLRDGQLKSKSYSILIMFMWMNLMLSWGLAPLFVTNYFLETQVENKIYRYRFNILSFVFPVTDQFYNDNFMIYYYIEFVYLILWCHCTMNFDILLLSMNITFKYQLKTIANSFSTFNITHYVKNNLTKNVKHRKESELMSDFKSMIYDQQRIIENMRNIYRIFQPVSTYSTSFRVYNNNSSVLYHNDELF

>AgosOR29

MATRTKMATKNEDNLMINTRLMKLTGLYHLLDSRSSKSFGHNVLKYLSLVEMSILFTMMVIFTANLYYFSDDINAVMQYSMLFACGVISIHKLYVTLVKSDTLWNGIQMTSIEDLSYKYHDRSILSKGQLKSKTYSILIMFSWINLIISWSLAPLFVTNYFFETRVENKIYRYRFNIMNFAYPATDQFYNDNYMIYYYTEFVFLMLWCHCTMNFDILFLSMNITFKYQESELMFDFKSMIYDQQRVIENMRNIYRIFQPVVLTQLASDGTFFFIQNYFNGISLISAMNLRLFAAVLTVTFHIYIICYIFDDVNQQVSYIKDSINFALYSSDWTQSNPQHKHLLLHAMRMNNAENLRLQVTRKRIVNFKMFTDVHSINFALYSHDWTQSNAQHKLLLLHAMRMNNAENLRVHVTQNRIVNFKMFTGIMRKAYSILSVLGKMCAKKT

>AgosOR26

MNNAENLRLQVTRKRIVNFKMFTDVRTIFLFFFILVPTTDIIFGHNVFKCLSVVQMSSLILLTVIFLSNIYYFLDDINAVMHYSTLLTSDMISIFKLYVTIVNSDTIWNCIQMTSIDDLNSDTIWNCIQMTSIDDLSYKYHDRRILINGQSKSKSYSILIMFMWMNLIISWSLAPLFVTNFFLELEVENEIYRYRLSIMNFVFPVTDQFYNDNYVIYYCIEFTGLILWCHCTMNFDILLLSMNITLKYQLKTIANSFIIYLYNYVLYAFNNLTKNVEHHKKSELLMFDFKSIIYDQQRVIENMRNIYRIFQPVVLTQLASESIIIILLSCILMMWHFFFFIQNYFNGISLISVMNLRLFLAVLTFTFHIYVICYLFDDVNQQKDSINFALYSHDWTQSNAQHKLLLLHAMRMNNAENLRVHVTQNRIVNFKMFTGVRIIFFFYFNAYLQICLKRYNFIFYI

>AgosOR38

SLLEPNEVSINLKLFKFIRFFHIFDPNIRKICNINVYHLAFHIINCVIGCIVIYGLLGYFTEMEDVFNIINQIQLMFCILIYYSSLLKIITFLYKANNIWDLLLVSKINFLTSTQCKTHIDILHKYRNKSIKITNMISTIGVVTTLEWIMYPLLLQLLQKEDANKSNQRFENIFNFRFPVTINYYNNNYAIFYFMESFITMYMLYIYVAVDVFFISACYIMIAHYEMIKRAYENINIELISENNNKNKNYCNDCIDDLVSIMMDQQKHFAKLKLFYSTYKFIILSTVIINSGSIIILTYASVVIFMSSETISILSVIKLISAFGYVFIVLFFLCYLIDRINNKMESVHFGMYSCNWTAMNLRSKKMLLLSMQLNNANKLMIKITPKKIINLQFYNSVILKFIYIYFFII

>AgosOR41

MTFDKSILESNEVSINLKLFKFIRFFHLFDPNIKKICNLNVYHLAWYIINCVIGCIVIYGLLGYFTEKEDILNIVNQIQLMFCGLLYYSSLLKIIIFLYKANSIWDLLRVSHMNFLTSSQCKTHIGILHTHRNQSIKITNFISGFGIVIALEWIMFPLLLQLLQKEDVNKLNQRFENIFNFPFPVTINYYNNNYVIFYIMESFIAMYMLYIYVVVDVFFISVCYVMIAHYEMIKRAYENVNTELVSENNNKNKNYCNDCIYDLVSIMKDQQKHFALLKLFYSTYKFIILSTVTINSGSIIILTYASVVIFTSSESIPILSVMKLISAFGYMFVVLFFLCYLMERINNKIESVHLGMYSCNWTAMNLRSKKMLLISMQLNNANKLMIKITPKKIINLQLYNSVIITCYNVLSAMLNTRSE

>AgosOR42

MLNLSDNGDCIVSSVLAKCTGLYYIINPKSIKLGGHNVFHIAIMVMITFTSVCLLLCPIGLYYWVNDVTQFIIQLIVLGNFSFGCFKAFTIVHYSDDIRRCLDVTRFDFLLRLPRHGS

>AgosOR28

MDNFNGKNILINFKLCKQFQFYQMICSSRMKIFGWNIHQLVLYKQRDKNSKFVNYFLIFSFVIILKWFIFPIVINQILYFENSNVRAQNIINLCFGFVLIFYLLEITVTSLTVYILIMMDTLIIFLCSAIIYHQEVLIYAFKNIGTVLILIKIGSKVIYLTARLFIYCYMFDSINIKRELVNYSIYFCIWTKMDFKFKKLLFLTMQMNDANRMIMSMSFNMVLVLLKITNSENHKSQLT

>AgosOR7

MDGFNEQNILIDFNLFKELQFYQIFYSSGIKIFGLNIHQLFYISYALVALCIESYGISTLFSTNCKFLSYIDYFIIFYVVNQMYLSFWKLFKCLNDRNRLLDLFKIAQLNFLTSEECTKYSKVLYKHRDKNSKFVNYFLIFSFVVILQWFMFPIVINQILYFENSNVRAQNIINLCFGVATLTYNKFVLIFYLLEITVTSLTVYILIMMDTLIIFLCSAIIYHQEVLIYAFKNIGYEDNPKISKIIFFYSKYKKLIRINVYFFRKIKLFYSIMKSTILLTVGIDSFYLIFFTYLFILICLTPGSDTLLPLIKISMSVVYITGRLFIYCYLFDSIYIQRESVNFNIYSCNWTKMNLKFKKLLLLTMQMNDANRMGIKASPKKIINLQLFAGIMSMSFNMVPVLLKITNSENNKP

>AgosOR8

KSLDGTFTSFFYIFYALVGLCIGCYGIFTSFSTNCKFLSNIDYFLIFYITTQTYLSYWKLFKCLKDRNRFLDLFKIGQLNFLTTKECAKYNKVLYQHHDKNLKFANYSFIFSFLIIMVFIFPLVINEIIHFENSNVRAENIVNFCFGVSTSTFNEYYLIFYLLEITVTSLTVYILIMMDILIIFLCSAIIYHQEVLIYAFKNIGYEDNLTISKTMKIKLFYSIMKSTILLTVGIDSFYLIFFTYLFILVFLTLIKIGSTVLYITARLFIYCYLFDSINKKRELVNYSIYCCNWTKMDLKFKKLLLLTLQMNDANRMGIKASPKKIINLQLFAGVIIKF

>AgosOR34

MDGFNEQNILINFKLFKQLQFYQIFNSYGMKIIGWNIYHLFYIIYALVGQFIGCYGMFTSFSINCKYLSDTDYFIFVYTAIQMYLSIWKIFKCLKDSKKFLDLFKIAQLNFLTSEECTEYSKVLYKQRDKNLKFARYFLIFSFTVSIQWFIFPLVINQIINFENSDVRAQNIINFCFGVSTSTFNEYYLIFYLIEIIVASLTMYILIIIDALIISLYSAIIYQQDVLIYAFKNIGYEDKPTICKIIFLVYIFKMNVYFFRKIKLFYSLMKSTILLSVAIDSLYLIFLMYFFILVCFLFKLIQL

>AgosOR24

MELQNEHSITNLQFMKITGSYQLLMPSHGLTFFNINIYKIAFIVQILFLTIAAIMGVFSIYSCRNNVNQIIHYIIVIFAIYFAIYKYYFIIKNSKIIWDCMHMMSTNFLSYNDHTKEIFKIARTRSFNVDIDFPYLSAILIHSSYLKIKHEHGIYKYRSNALGLVFPVTDTFYNKYFIVFYTTESIFLLFWGQMMWVFDILMISICISIEYQLKTIADSYSLLGLKDKHLTRELSNSKYIRFFVFIQLAAESFQIILHACMILKLYFDGSMSPTIFLKLLFPEITYLCHLFLTCYLFSIVNEQESMNFALYSSNWTDMNIKFKKLLLFTMRVNNAENLKMKISINRIVNMEMFADVMHITYSIVSVMMKSYSK

>AgosOR15

MTPDNTLKYIINLKLMKLTGLYQLLNPDNPKSFGCNIFKLGGTLAVVYLILVIIMCNLSIYYSLNDFTEVVKYIMLIIAALFASTKMCFVILYSNELWKFISFTSIDYLSYKGHKKYMHNKARKLSKSISNIFTLAWIAVISVWILSPIIIKDNFMNVKSKDDTYNQYRYNMLNLIFPVSAQFYNNNFKVFYFFESIALIVYGYSMMVFDCLVISMCITITYHKPLDERSNDLNNLILIIQDHQKCIKNLNKGLSLTSPESIKLILSALTNIIHLFSTCYIFSIINTHKDSINFALYDCNWTNKNIMFKKLLLLSMKINNSEKLKLKASSQIIVNLQLFTNVIHTTYKIISVLVNQYR

>AgosOR23

MNPNDENYIINLKLMKITGFYQLINPHTSKYLGFNVYKVGAGLEVMFGIISMLLLFLSSYYYLDNTNELMSHFMLIVAIFFSTFKISWVSKKSEMIWNNLDMTSINFLSYTGHKQEILQTARAKSISTTIIFVILWSSVTVAWSISPFFIKDVYLNVKFNDEIRRFRYNSLNYVYPITEESYNENFLYFYVVEMLQVIFWGHGTVAYDTFVISICISIAFQLKTIAVSYTSLNDIKGDIKNLKHNDLEAILNLKLVIQDQQKMFKKIKEIYKIFQPVTFVQLAAQSMLIILQAYMIFIFLLLSVPIIKLIVTVAPNIIHLFITCYLYSNINDQVLNLIIRHIIIIMTIIYPQNILIILSIILFQKDSMNFALYSSDWTAMSIKYKNMLLFAMRMNDAEKLKLKISLRKIVNLEMFASVKPKIKFYEKNNN

>AgosOR11

ISIYYSQCDFTAVVRYIMFIYATFFVIIKISFLIIKSDILWNFISFTSINFLSYSGHQKYFLMNARIISLIISNVFAILWVAFIALWIFSPIVINDSYLNIKSKNSTYMQYRYNTLNLLFPVSTQFYNDNFTIFYLFETIILIIYGYSMIVFDCLIISFCLTIAFQLRTIASSYSTLGYNHTNNQIKSFINNIIHIVNTINSNNGISLTSTESIKLLSAEIVNTGHLFSACYLFSLIDIYNDTINFALYNCNWTEMNINFKKLLLFTMQMNNANNFKLNISTNIIVNLKLFTNVIHFTYKIISILKSVVN

>AgosOR4

MTLIENKNYHRKFYTILMTVAFFLNTSQYDCIPKFLMHFYIFDWMMFVTLAAGYIFFYEKPGMSLGMELIQYMIVGILYTLIFLVFILKNEAIMSNYNFIQTKFIHWSNKKSLHPNAVYKKNIETVKSLAIPLATLSLSIAFGPLVSTINDIGKLPLDNRAHFVLFWPKIVDTNKISMYGIIYAVQVLFTVILYISSLSFNLGFMVFLNELTNQFEILLDGINDAFKYKRDKQFQPLFIDCIRHHQIIIKFLDDLKSYFKWVILIEIIVVQVVLAILIYNLTKVNASIGYKVKVAGSLLFNLLPICFHCHVGEVILSLHTRLSNHIYNMVWYDMPNKNKQLLVIMFQRTQRDLTLSSALFSNERASRSLISKVIKQVYTILNVLLKT

>AgosOR3

MKNTAVNEMKFYQILIAIAFSVNTGHPYNYIFQYIIYLGIFNYILSLIIAPLYIIWEGQTMSMIMELILYIICGILHSSHLIVIALNKQSIVATYSFIQTHFFNWSVKRGMDPNGAYKKNIKKIKYFVTIWSFLGCTIMFSPLLSTIADLGELPLDHRSHWNTHWPIFFSINNLYTYGFIYLLQTILAVFLITATGAMKCGFIVLLSELNYQFEHLLHGLEHAFNHRMEQKFKIIFFDCVRHHQYVDNFKSYFKWITLINIFMLQGIISTSLYCIIKIDAPIGYKMKQCGIIITHIAEFLFHCYLGEIISRMHNHLEEKVYNMTWYDMPNPHKKFLMIMLQRTQKDLVPNAAIFSSHSLSRSLMTKFVKQIYTLLNVLLKT

>AgosOR40

GKLNKKEIQKEFVSIILDCQTVVFTLEKIFSQLKTIYGIARPIILIYMVGDSIGMITMPFLIVMFYTQDKSIFNSNVIAFSWTLLVVGIQLYMYCSLLQNVNERRENINFGLYSCDWTRLDIEIKKLILLAMRMNNSNNLKINVTFTKFIDLPMFASIIRSSYSVTSVLINSNIHKINK

>AgosOR35

MKRSRFYHIFNPNGSKIFNYNAYRLLLIILLVLVNGIVVYSSLGFFVKMEDTLSYIDSSVIIFVMINIFLCNWRFSVFLYNAKIIYDVFNVSRFDFFKSKHCCKNINVLCGHRDRTIKITNYFFVFSSTVMSQWVLFPLVLIAFTAPEDENIRQQNIMNLRYPVSTHTYNQYYYLFYLMEVIVAIFTMYSMIIPDILLMSWCWAIIAQQEILIQTFKYFGHEDSSQTVHYEDFKSILVDQIQLNLKIKSFYSVVRPVVLTYVAIISTCFIIVTYVLIVVCLSKESNSVLNIIKLGSSALYMCLDLFLYCYLFDSMNIKLESVNTSIYSCNWTKMDVKFKKLLFLTMQMNNANNLMIKASPKKIVNQQLFANIISMSYNIVSVMLKTTS

>AgosOR45

MKIQDNDKQIFNLALAKFIGFYQVVDTEKVTFLGRHNVRYKIFVFLIVYECLIAAILLLNCLYYSENNPTEFIRYTGFVVNMFYASYKMYIVLKRTKDIWDCLSITQFDFTSYDHRDRRIILDLWRNRSIWFTNTFVVFSLIMLIFYTACPLAFNDTFIVMKSRDGLSSNYRLNVVNLYLFVPEEVYNAYFNIFHIIETFGICIFVLFIVVFDTIVMTSCLALSCQLHMNSAAFESVGHTSVVDSPNNNIDNKMKLPYENINGIAMYNNLKTIIIDHQNVLKKYDEFLSIFKPIMLLEIFVLSYSIIILWLIFLTNFIVGEFTDSKGVASMQTCFAIPFCIMQMFMSCYVFDILHNKHNNNMTIDYRLGSNK

>AgosOR43

MDSKHEKQYIFNMKLAKIMGLYQILTPDSTSIFGYNIYHIVIVFFGSFMFIISMLFPIGLLYLRNDMIALMYYMGCISNFILSSYKMGNILYYSKDIWKCIDVTNFYFISYKHYDRNLFKNWQTRSIRITYIYIVIALFAFFCWIFSPCVMNKSIITIRNIDGSYSRYRMNIFNIYLIASHETYNTNFYIFYIIEIIVSVCYVYFTIIFDVLMLLICFAISYQLETISNTIKSLGYNLSIQDNIGTKCVIFTGTSNSIQLKEKCDIIYNDLITIITDHQHLNDFYNMFRLVTLTQIFIASSSHSIDEGDNADSILSFKLFIVLPLINFQLFMTCSLFGTINEKKDSIIFALYSSNWTDMDLKSKTIILFGLTMNNANQLKMKFTNTKIVNLEMFSHTMRFCYSIFSMLVNYNNKKKNN

>AgosOR12

MDSKALKILRLTGLYQILDPNTAKIDDCNIYHIVIVFFASFTLVVSMLFPMGFIYLRNDIIALMFYVGCISNFMLCCFKVLNILYYSKDIWKFIDVTKYYLTLYKHYNTNVLKNWQTRSTRIMYLYIIIMFIGFCFWFFSPHILNESTVTIRNIDGSYGKYRLNIFNLYLIVSDETYNMYFYVFYVIEIIMQICFLYFTIVCDIIMILISFEIISRMEIICIACGSLNYNIICSKEGSNPIKSEKKFDVIYDDLKIIITDHKTVIRKLKEYYTIFRPVTIIQIFITSSSHIIIWFVVAMNFGEGDKGNSIITIKLFAVHPLLSFQLYMMCYLFGSMNEKKDSIIFALYSSNWTEMDIKCKKMILLAMEMNNANHLKMKFTNTKIVNKEMFTQVNITSHIYKVNYKMNVFYYL

>AgosORco

MGYKKDGLIKDLWPNIRLIQLSGLFISEYYDDYSGLAVLLRKIYSWITTIIIYSQFIFIVIFMVTKSNDSDQLAAGVVTTLFFTHSMIKFMYFSTGTKSFYRTLSCWNNTSPHPLFTESHSRFHAKSLSRMRQLLIIVSIVTIFTTISWTTITFFGESVWKVPDPETFNQTMYIPVPRLMLHSWYPWDSSHGLGYIVAFALQFYWIFITLSHSNLLELLFSSFLVHACEQLQHLKEILNPLIELSATLDSAVHNPAEIFRANSAKNQPINGVDYNGSYVNEITEYGTKGETELNRKGPNNLTSNQEVLVRSAIKYWVERHKHVVKYENLIRDCYGSALLFHMLVSTVILTILAYQATKINGVNVFAFSTIGYLMYSFAQIFMFCIHGNELIEEVTVMEAAYGCQWYDGSEEAKTFVQIVCQQCQKPLIVSGAKFFNVSLDLFASVLGAVVTYFMVLVQLK

>AgosOR17

MATASPSEEESTIVDNRLFKAICLHQILNPTHGSNRYIRSQEPFSKHQSAIMATASPSEEESTIVDNRLFKAICLHQILNPTHGSNRYYRIAILACIWMSIVVQITQLVGLYYAVNDLQRFAFTTTVVVNSFLSLAKAYVLMANVDRLRDGLEAARYEFTSCGSRDQRTVRRARAALSTLVRTFTVFSYVTCFFWMLNPLSAIGEFLPLTNADGTVSHYRVTIYNYWLPVSATVYNTTTVWALTYAIEMTVCFFNVNTWLLFDSYVLTMCFTFKAHFRTLSASYATIGHLDTFRSLTPHASGFLNWKKGLNKTQNKFKFKHVLINIL

>AgosOR31

KANSVIIILKYCTNIATELYNLHNYISISAFWSFNGFFCPIIINSFIVDKNDNRRLENVINRRYPITVNTYNQYYVLFYIIETIIAIKSLYLILMIDILLLSIGWAITVQYEVLAVAFKNLGHDVNFQKGETTLFKKIYKKVKLYFSIVKPIVLMHVAISSGLFIMLSNSFIMIILSKESFTILIVNLFKIGVGIFYICLQLFLYCHLFDNINLKREFVNLGVYSCNWTKMNLIFKKLLLLTMQMNNANQITMKATTNKIVNLQLFSNVNIKFFVLNKSYNIISVMVKAISK

>AgosOR44

MMLLNGLYYSKNNITESILYTGFVINMFYASYKMYIVLTRSKDIWDCLSITQYDFTLYDHRDRRIILDLWRNRSIWLTSTFMIFSFVMVTFYVTCPLAFNNTFIVMKNRDGSTSTYRMNVLNLYLFIPEEAYNNTYFNVFYIIEASGTYVLVLFVIIFDTIVMTLCLALSCQLHMNFAAFESVGHTSVVILKTNYFCLDNNDNIDNKIKLPNEYINGIAMYNNLKTIITDHQNVLKKYDEFLSIFKPIMLLEIFVLSYAIIVLWIIFLTSFIVGEFTESMGVTSMQTGFAIPFCVIQLFMSCFVFDILHNKKDSMTFSLYSCNWTELFDMKCKKLVFLTMGMNDAHHQKLQYTRTRIINLEMFYQVCFTLSS

>AgosOR37

MKWLQDHEVAINLALFKRYQFYHIFNPNSSKILNYDSYKFTNVLFIVVVTSYNIFSAMCFFTDTVDTIDSIDLLLMIFIYSIIIISLLKICVLLFNADQIWDLFNLTRFDFLSSRRCRKNVGILYKYRDRSITITNLYQNYSTIVFIIWMIVPLVLNTFAMTEGPNQRYHNIFNMQYPVSASIYNHYYYIFYLMEIAMGIFVLNYSMIIDNFLISLCWAIIAQYEVITTAFENIGNDCKLENLQKEKKNNSFEAYEDLKSIIMDQKKIYTLICSSAESFSIFNILKISTAFFVFVIQLYLYCYLFDVLNDKKESVNLGIYSCDWTRMDLKLKKILLIAMKFNNANQLKIKATPNKIVNLQLFSSVMTTTFNIVMVMLKTINEKN

>AgosOR13

MDSKHEKQYIFNMKLAKIMGLYQILTPDSTSIFGYNIYHIVIVFFGSFMFIISMLFPIGLLYLRNDMIALMYYMGCISNFILSSYKMGNILYYSKDIWKCIDVTNFYFISYKHYDRNLFKNWQTRSIRITYIYIVIALFAFFCWIFSPCVMNKSIITIRNIDGSYSRYRMNIFNIYLIASHETYNTNFYIFYIIEIIVSVCYVYFTIIFDVLMLLICFAISYQLETISNTIKSLGYNLSIQDNIGTKCVIFTGTSNSIQLKEKCDIIYNDLITIITDHQHVIKKLNDFYNMFRLVTLTQIFIASSSHVFIWSIGAMSIDEGDNADSILSFKLFIVLPLINFQLFMTCSLFGTINEK

>AgosOR36

MDSFNGQNILINFKLFKQLQFYQIFHSSGLKIFGWSIHQLFYIVFGLVGLCIQCYGLSTSFFNNCKNISEIDSFLIVFASTYIYLSLWKLFKCLKDRKIFLDLFKIAQLDFLTTKECTKYSKLLYKQREKNLKFANNFLIFSFVICLQWFIFPLVINEIINFENLNIRAQNIMNLCFGVSTQTYNKYYFYILLTRNNTNIDISFHYNIYGHINNFNLFSYNLSTRFTNLCVQKYWI

>AgosOR27

ISFTSINFLSYNGHQKYFLLNARIISLIISNVFAILWVAFIALWIFSPIVINDSYLNIKSKNSTYMQYRYNTLNLLFPVSTQFYNDNFTIFYLFETIILIIYGYSMIVFDCLIISFCLTIAFQLRTIASSYSTLGYNHTNNQIKKNQLINEKPMDLSNLIVIIQDHQKLTKKIHDIFDEMRPIILFQLLSESILMSLIPLVLFLNSNNGISLTSTESIKLLSAEIVNTGHLFSACYLFSLIDIYNDTINFALYNCNWTEMNINFKKLLLFTMQMNNANNFKLNISTNIIVNLKLFTNVIHFTYKIISILKSVVN

>AgosOR16

MDSFNEQNILINFNLFKQLQFYIFYSDGTKIFGWNIHQLYIVFGLVGLCIQCIGLSTSFFNNCKNISKIDYFLMVYASSQMYQSYWKIFKCLKDRNRLLDLFKLAQLDFLKSEECAKYSKVLYKHHDKNLKFSNYFLIISFVVIIQWFVFPLVINEILNFENSNVRAQNIINFCFGVSTQTYNKYIPIFYLLETTGAALTVYFLTMIDTLIISVCSAIIYQQNVLIYAFKNIGYKIKLFYSIMKSTMLLNIVIDSFFSMIFTYLFILVC

>AgosOR14

TKIFGWNIHQLFYIVFGLVGLCIQCIGLSTSFFNNCKNISKIDYFLMVYASSQMYQSYWKIFKCLKDRNRLLDLFKLAQLDFLKSEECAKYSKVLYKHHDKNLKFSNYFLIISFVVIIQWFVFPLVINEILNFENSNVRAQNIINFCFGVSTQTYNKYIPIFYLLETTGAALTVYFLTMIDTLIISVCSAIIYQQNVLIYAFKNIGYENKSTINYYKTFISILQDQLRLNLKIKLFYSIMKSTMLLNIVIDSFFSMIFTYLFILV

>AgosOR18

NNNTLNCYDELVNHLQDNQRIIKKYDEFFEVIKPVILFQIIGGSYTVITLTFLTSLVSVLVFPRKIFHIYINTFLNVYLFLFLLRRILWGFQLYPYLAVNFGLYSSNWTAMDLKFKKTLLLAMNMNSAHRRVMKVIPRSIINLELFAKV

>AgosOR19

MGVLVVNGLMCLLKGYMVVANADRMCSTLDATRYAFTGCGGRDPSELRRCRATLSTILRTFVALSFGTLVVWVIIPGKYELFFRIVRPVVLLQIANGSYSIITLIFLTSIAYLNGDSIVSPAIFKLVCALISLTIELYIYCYGFNHIEDGRSTVNFGLYSSNWTDKDLQFKKTLLLAMTINSAHKLKMKVSPNSIVNLEMFTRVMNMSYTIVSTLLS

>DcitOR1

MQKVNKHGLVGDLWPKIRLMQLTGMFILEFHEDSSPGTQLLRVAYCWIVTALLIAQYACVITFTVGLNYNNDLLAGGVVTTLFFTHGMIKYIYVGLKNKSFYRVLSSWNNANSHPVFAESNARYRAKSLSRMKRVLTIICVWTSATVVAWVTITMCGDSMFKVPDPEDKNKTMWVKVARLPVHSWYPWDCLNNSTAHIFTFIFQVYWVFSLVAHSQLCDGMFCSLVLFSCDQLKHLKEILKPLIDLSISQHGIPSSLDLFVNKSASSNKKLIANEDFEYQNMYQSQHDFSNYTQSKDSFPVDSQKEELTRSSIKYWVERHKHIVRYTEMVGDCYGISLLLHMLVSTVALTLLAYQATKIEGVNVYAFSTIGYLVYALAQVFFFCFYGNELIEQSSSVMEAAYSCSWYDGSEDAKTFVQIVSQQCQKSLSITGSKFFTVSLDLFASVLGAVVTYFMVLVQLQ

>DcitOR2

MKIPFIDETEHWNYTVCFVYQVYIMLIWLMMSSVTLTLVPIMSTYLIGQYTILSKYVEQLGAHHYGSNGQEIFYINIETNEYYEYYNK

>DcitOR3

MYFLICSFFTVYLAGLLVVLGTVCFCNFPLFVMNVELQIRVLSRYVRMIGKIHQNPQGDVIRYTNIINDEYVLQRRHEISRSYFHDMDKIVSSRDLEKYQHGYMNYEELFTRQIFKFHQKLLLLREEYIGWLHMSLVGRALFSMALLALAVYQVFSRDIYSEKTVFKYCLELTCVVSCYFYFCHISERWQGCNDHVRRAMEDSCWYQCSSRVQKNVIMMHVRTYRSVDYVEACGIFRASYEYYVSSLKLSYSFLNFVKLQERSSMKAP

>DcitOR4

ERLEECSHLLKNSITQCDWHRCSSKTRKGLCILLRRAQRPNYPSFSWGLVVLQREYTLKAARVGYSFMNFMRLKSG

>DcitOR5

MAYPHSMRKVLRILNYTGIMNHLVLYEARWKQVLHRVFCVICQVIGILSLISHILSTTTRSRRFLPEFLQRMLEDTCFISYSFEYLNCKYRMDEFLEIVTRMENTFSSVDKTIVKNCHRTEINIYLSFSSLLGLALLGSIIETYLPISNEELAILERVYRRKYPENRLETNFWVPFDDSDPKYYHILFYFIIFLILRILVMSLCVAFLPVLITQITGQYKILNLYIRNIGADLDDNGLRKYFTNIERGEFIYADVSKKSKNIPHNNSPQETIKSNVVKLKRQVLYEKLYVRQIIRFHQRIIHFEERVRVYFLYVGYLVVLGGETPKYEIIS

>DcitOR6

MPLPSTSQEGLSANVPSESRELQWEAYERWYIRQVILFHQKLLGFQEKMLSLIRNVFLIIVTCISITFSLSMFEIFVCHNLLIKVTVIKLAMMFISLLVHLYFVCYCSEQLNDSNLMLRDSLYNSLWYNCCP

>DcitOR7

LLSRKIAVEHCSLDNMYYLFNPQYKMFLKDLYESIFAPFIVIWVLAFYQMAYMKHLPFYNRFKIVFEFLYTFLAVFQFAIKSESLNDCYVRLAGALYQSRWYNCSPEVRKTLCILLRNFQDSRHFFLLRGIVKVAFTFLLRMVKVAFSFINF

>DcitOR8

MLALQLERSTSASHLHDIIQKHQHLLHFRDKLAAVLANTCLLKAFIYNLTMTLSLYQLSSVKTVNQRAFQLIFQYVVILIMFYSFTTSSEVIDEGNDILYGAIRSYKWYSARHFDRRAAKTFQLLTRMCRKP

>DcitOR9

MKFRLVDRTKAHIKSKIEAIFHANQNYPCLIFTTYLAQVFRFQSIASTKIRFLSLFNFACFQCVTLPLFLQLIFVSTKNVIVLSMQLFESIYCVSVCIEYGLQFISHKRNVSLLQSFHRIFIKNHKYVKRILTIEYVASTIITIILVILGSMWFLESTISTMTEEELQLTNRQNPDRKFKTEFFLPFDYSLSPYYELVWVVVVYYGVILQTILFETITTMPFITLHIKGQLDILAYYLRSIGKLFRLQNNIEKILFPSDLEKVFCS

>DcitOR10

MHEHITNGLYFFSHIEIITTVIVGLDIVSDVSENVLVVDEIRVENQASLRITMLLT

>DcitOR11

MTSIRIYCDFLDKMGLSNFSRSKEKSHYIFMLFQYSGIIFFTVCHGWNTFTRATRYLPEFFQSLYEDIAINFVVFQILCFNQQSEKTMELAIYMENSFCTINNKAIQKSRIKSKKAAILIFLLITLIMSFSALQSIIPPSSEEIELLANIYRTKHPERIFMSTVRIPFIDETESWYYEAIMLFEVLAELVGIALVCVVCTFIINIIYFMEAQYTILCQHIENLGKKLRDSQGQRFFYTNFKNNDIEYLSTIQNKKMFASNKRKPSLKQRLMNEKIIEQHYEKLYLRQIIYFHQKLMLLQQKVSRYMLTIL

>DcitOR12

MEYFIICNCSEIMDDCNGLMRSALMNCGWDKCSTSLRRDLCFLWRRVQRSNHLRFYNGAVILSRLFFLQVVKVAY

>DcitOR13

MIRFMETSFSQFDSRVILKYKKRADLMFNVYLTMAMAIMCGALLEPFFSKTAAEFRIRRLVYQTDHPEKRHPFNVRIPGVDETQPGVFEAIYLFEIIY

>DcitOR14

MALTSVCYIPMFANYIEGQYIILGKYIEKIGHIHRDKKGHRIFYTNIIKKKVMYVGADNELRHRFKFKSMQEIEDIYQQNYCRQIIQFHQMIIRFQEQLFKLYRPYMMMKVVMNNVMFALCMYQVSTNPTDTSHTRFYKLVTELVGVVVQFYYFCYCSERLDDCNAQLRRSFSKVAWYSCSPHIRRSLIMLLRRVRGSNHMKLLLNFVVIGNGYFLTVVKFSYTFVNYMRLKSK

>DcitOR15

MPLTTISLQVHYDLLNDYTKIMGTIHYNPSGQRVFHMNVRRGVCVEVESLFRGKRRKELNSRVYQEYEYYFMKQLIQYHQELRLMRRKIDSYLKVTWILRTGLMGPLLCTCMYALLTPGLLSTVIYNKFILESIGFLMASLFVFLAGELLASFNLTWRSALYNSPWYKCSNKTKKVIPLLLLLNQTHDYHSLNGLIPCSHEYMVRMIKMAYSVFNLLRVRRLNT

>DcitOR16

MAISTVPIFCVHIAGQLRTLSKLAESTGPREGDTRNLALIKHHIGIIRFFKEFETIFRPVFMIKIVLRLIFITLEIVQLSLLKSITDPRFTRTLTQMVIGFTDFLFFCASSEVVNIGEETLFRSVYNSRWYEASPRIRQRVSMMLEYLKRPLKYSFYSTFLIIDMSTFIGGMRLAYSVFTLLHSFIE

>DcitOR17

MPPVNRAQTILEVLALLVGYMVMFHCAETLAACNDTLRTTAWCSQWYQCSNHTKRILGFFLRMNQKLKHIVIFKILLFGYTFMI

>DcitOR18

MSFSFGFRLLYKTLEIIGFVNRVQLRYSWLNKLKTLRHMMFHIFTAAGMISHFISTTCVARRYMPEFYQRLFEDITSCVYYFDCMFYLLNYDRIHALIKFMDTSFCYANDKVVTTCVRRMRLLFIVVLIRNTITMIAVVAETQKPVSQEEIDVLAELYHDKYPERRVPVRYWLPFIDESESWYFEAILTFAFYQFTMVAVMANVCICSIPMIVLVINAQYTILAEYVEKIGHEHRDEEGRRIRYTNIITNEIVYVEEEGRNQGSRRYEEKPAADIVKDNEIIYVRELSEQNEPRKQIQVKGDMAEEEYQQDYFRQVLQFHQMMMDFQGELLDMYHPFLTAKVLLYNLTTALCIYQVTSNPTSFSESRKFKVICELIGVAQGFYLICFCSERLDDCHGKLRQAVARADWGRSSPKIRKALQVLLTMAQTPNHMEILGGVLVISNAYYQGMVQFAYSFVNFMKLKMSA

>DcitOR19

MFRDQLTGFCNNIAFLVIICNNICLSLCLFQLTNNPGDLPPVRLLKFVGQFLNIVVQSFLICNSSELLDDYNTMLRRAFNESCWYACAPRTRRNIVFFLRSIQEPGHVRFYQGTVVLSRKYFLKIVQVSYSIVNAMRLRSNLS

>DcitOR20

MNSIMANIKQNHNYLNLNILLLKMALIWPLETKIFRTAHKVFQYSTAFILFSVSVGQFMTILKTNDLRLISSTVDVFTLTASAMYKQLFVAGNIVSMRYIVDVIHKDFYEMPLVENKAKRPKTFRMKHHLRYAGLFSKIHVTSAVILAVIWCFGPLLVKPQMSLNPARNPGENLTIPAFGNETSYNGPSSFGSPHGSDLLEYPLFVQTNVLLFGNDTESNSGRKNNIENRNEPKSDTEKSSGQSGDISAYDDKNDIGKAKTQNGQSGYTANDKNAIGKPQTNNEQSGDIANENDSHKAQSGNFDGIRTQTSQTTSPTPQRSDRLRKGSKGEVDSKHSVQYDSLNQRRILPMNVWTAGISYHNSPWYEIMFGIQFWAMLMSCVIYLAIDAFYFSIIYICCGQLQLIHISMENMFPPEGDGSDGTKGRDGSDGKGRDRRVRLINEIDNARLCKIARLHAHVLRLVRFLEQTFTSTIMVDFFHAVTSLSFALFHFQTAQGLVEFTKMYVFLIVCILHQFLNNYFGEIMKYWQSQLSYSTYETPWYFRDRQFKKSIQIITARTRVPIMLNGLKMYVLCLASFIEFMKRIFSYYTVLREISK

>DcitOR21

LKSLESSRSKDMVCPHYIHVLCKFLEIIGYLNEFRFKRNWLNLLKKIYFIWFHCIHTTALISQLISTCTRSIRYVPEFLSRLLESFALYLFYLECLFILFQFKQLQGLMKFIENSFSTADECVLKRCNQRARKMVTTFLIL

>DcitOR22

MVYPHSVGTILKILEWVGFTNHSRCQVKWKNILKTVYFVSFHFLCIISLISHLLSTTTRSIRYLPEFFQRLMEDITFHLFYFDNLLYIFRYKELQSIVNFMETSFCTTNRRVVQHCYRRGNLIMIVFGLVHSMVISGCILETYFPVSEKELELLRYVYQRKYPERRLQTNFWFPFIDDSESWYYEVMFYTEFYLVFIVTVLTVTSICLIPMLITYAEGQYTMLSHFVEKIGQPHHDEQGRRIMYTNLIKNEYVYVASCYERGKLVGTSSWSDKEKVEHLYEKSYCRQIVRFHQMLIQFQEKVSVSLQILVDTLSRKRSSCELEEF

>DcitOR23

LPILTLHIKGQLDIISSYLRTIGNDSITLVDIKRGSLIYLHCDNEEAPSLRAACTKHLLTQIIRHHQYVIRIIAQFSRIHQVVFGLRIFLCTTLLLGVLCQYAFTNNTHILFVITNAVTVLQLNFFFNSG

>DcitOR24

MAYKHSIYLLFKLLRLTGFQEFYYKFQSEWKNKLMTVLFWTNNILGVFTLGSHIINTMTASTRYMPEFFQRLLEDVAFFEVYLEANLIRTNLKDLMQLLHHMETLFISQEL

>DcitOR25

MQRMFDKSYPDIQAKCQTAERRIVIYMSGFVFAGALYCYVKRMIFGMTENERKLSANSHNKANPEKQLPFLLWLPVDDTVSPMYYVTLAYNVLFTVVSAVMTYHLITYLPIICVHMQGSCETIARSLTQLGNLRTDHTTRDDLDRNGASKSRRNEIPLDLVHGTQMGPRRERINQSEHDSQIDHALNITSVNSASKLRRYSDPALSSLIAIIIQHQQFLLIRNQLQAAIQRSLLLKNFAYNIILTVSLYQLNSVDFADKDRFLQMIIQYLLMIAMSFSFTQSSELLDRGNDAIHEALTCGAWYNMRARVARLLIPMIRVSGRPKRLMYFAGTVDISYRSFVAVLKFSYSFFALLKNVI

>DcitOR26

MAYDHGVYPLLKLILVLGYQFEITTFKAKWKNNLIEVVYWVHQIIGVASLISHVISTTTRSIRYMPEFFQRLFEDAAFNLLFIEAHFIRANFQDLHELIQHMETSFSTQETEIVEKTYAKAKFIFYVCVTSAIGCLSGSILETFFTVSQEELDLLSHIYDRKYPNNRLQTNFWVPDIDDSEPPYYQIIFVTELYLIYLIILLTILSVSLIPMLVTHIAGQYEILCNRIQSLGHSSIMWNKRRPIDDLQDRESLRSIMQAHKNLISAQSKLQNLYSAGMFIKVVFNNIMFALCLYQLTLSNTNTVSKFRVYKLTLEFLTVAVTYYYLCHCSEILDDCNSKLCDAIWNSRWYHCSRHVQKDLIMFLRRVQRPNHLKCLHGAIILSHVYFLGVVKVSYSFVNCMRFKSRL

>DcitOR27

MVTSLSVYPITILSQFDSQKQLLLVAEAMLTLGYYYYNCLLSESLEWSNCLVRQSIYQNKWYEMCPESQRMMLMFLQMTQKPHHIKTFGGIVALGNVHYLKMLKATYSFIRFINLIKR

>DcitOR28

LTACNYFYICIMSEVMESSNNGLRLAIYNSHWYNLCRPARQHVVMFLQRSQTPSHIRTFGGTVVLGYTHFTRV

>DcitOR29

MAIGGVAVATLLVALIPLTQAETDIRRQLYNMKYPDRLLPSEVKIPFVDESELPNYVFIFVFFAYIVLVSLILAAITINMIPFVTIHLRGQYSMLCNFVEKLGAPHTDLLGNEIRYTDIQSVSKV

>DcitOR30

LCGCGCGRQMERSPTSARFLYTIFQYLGVFPLQNPKPDWRSGLHRVYMWLQASLCAGLVVTHFLNTVLRAVHYWPEFLQRLLEDVILDAIFLIMVGYRMKMDEFNKILKFTEDSFSPAYPHILLQTRRKVQLKFCMFGVLVGCVFVGFLLELWAPLDEAELAMRRRLYQTARPERRLVFDIWIPGVDETESWTYEILIILQLYCGFIAYLTSVIAIMVIPTLVIYSQGQQRILCKHIQLLGTPHYDSDGNRIFYSNFEKNDYVIVLYAMERMRLTRRGWSQVYRKKQTEYERQYFRQIVRFHQKLLDFQEQIVKLTDILAPSIVLMNLITFSLCFHQLLTNPRHLSPPRLFKYLSEFTSIVVQYYTFCNDGDTVDDFHYHLRNALWSSGWTASSTATRRGIVIILTRLQRTTNPR

>DcitOR31

MYNMETLLRIVQVVHTQFEEYPIRGIHKFTMSSHIGPCSLYSSLYVLAGVISSFVWNFAPLFFSPMCPITDPTAPLTIKNRKVMPINIWLPINIAESPAYEIIFALESYTFSMSTILYLSIDAFFFYLIHVISGQLKLIDASFKSLFILDERLKHEHATRLREMSKHPDAGSLTVSKSV

>DcitOR32

MWVGIIFGVLLQFASTFIYSIHDLSEFLQRLLECLSDITILSTIFYYNVLMKEVIELLDLQEKEFKVSNIKIHKKYILQEQKFLAIAFLFSICVSTSFLIETILPKSERTLWLMTSLYHRKHPDRVLPFNMWTPSFIDASDMEYFVILYLLEVCLLVYFTAGVFEMAVMHIIFPISLKGQYTMLGEFVELMGVEHRDSVGDLIFYSDVATGEYLTETQMLHFVMKHKTDQPKAQIKQQRATALKKIPYSSFYTRQIVTRYRTLNQIMNKYTSLQETMYNVAIVPFLVIWVLSFYQMAYMKHLPMFNCIKIVCEFLCVVLGAFNFIYQTDYLNDSHEIIARAIYQSHWYNCTPQVRKIICMFLRSAQQPKNISLLGGVIMLTRQQFLKLINGAFSFLNFMKMTGRV

>DcitOR33

MSLLRIVQRPNHLKFSGGLIILSRVFFLNVVKMAYSFVNFMRMKSTRHGKDHHVYRVHSD

>DcitOR34

MKAGIRFYEALITFVGIAFQFYFFCESAEIFNTCSEDIRRSIINCNWHKCTNQTRRQLILIFRRAQKNNYMAFYSGAI

>DcitOR35

MEVKLKYWRTLIYPRFYSTQVVRRYQVLNQVMNKYIQLQETVMNITVLPFLMVWFLSFYQIACMKHLPKSNCIKIVVEFFSVFLGAYFIISQVDKLNTCNEIIAQAIYQSGWYNSTPQERKKICVFLKNAQQPREMTALGGIFKGNRQQYLVCIKAGFSFINFLKMTGKL

>DcitOR36

LFEIIYGFIFALVACVIMPIIPMIVIHLQGQFELLCHHVRKIGTSHEWEDYQIYYTDIETDQFCMALYSERMTREKDSLLNC

>DcitOR37

MIPKKCSQDEIRWRESVKAKRETLEMEQFKRIVKLHQSLLQLQDKVTTCLSSAVWLTVIQSNLSFSLCLYQMS

>DcitOR38

MLVGTMSLLVNTLFYSIRDVAELCERSIETVLVLVYILESVYLKLNLTKFLRIADFFEAVCIYHTKNQVVVAYKQRENYLVKYMSIAMILVYIGVTFIAPFLPRTQSQLLTMQRIYKWKNPHNTLPIYMYIPFIDITEMRYYVPLYIMQMCLGLLILVAGYIALILMPLTTISLQVH

>DcitOR39

MTQSNRREKYKIYEKYFVRGIIIRHQKLISCKQRYQDLMSSLFDNLM

>DcitOR40

LLSFIFSMTRSSSIQNTILTHNSMFSDFIGHPDILSINIKTGHITRHNEITRYDNRLIIQQAKSFLLQIVQQHQHILTFVKTFERNNQIFFAFRMLLTITLLLGVLCQYVFTNSQRAYIFFNLIIVMQFNFFLCTCSEL

>DcitOR41

MLAQILTISGEGFSHVDHSGYTSVPYRPLPLINVWIPLLDLSQPLCFYIAYVSLLYCMVVFYFMVSNLLTMYPIYLIYQHGQYRVLNKYLSYIGHRGTRRYIVDSGRLKYRRIQRCYVDLWRNQYKVVDTEGQSKEWQDRLDYSNVRQAVQFHQKLISFQHKFNRSMGVICSPIVITWFVSTIISLFELTRLQSHHDLNKLIRVCSEFLMTMTCYFYACFASETLAQCEAKLHLTMYTCDWVHLLPRTRRAVQLFLFRTQRDGAYMTYLNGSVVMCYRQYGNVMKMAYSFVNFMNLIFNTEAKQR

>DcitOR42

MKLQQKVQILFAPIVLPLVLCNNAAFSICLYQLTDRNTNLSNARLFKFLIEFLTVALQYFFLNNCSEMMDDCNTMVCRSIAFSHWQHCTSDTKRGLMSLLRVVQRPNHLKFYGGAIILSRVFFLHAVKMAYSFVNFMRMKSRQSGQPA

>DcitOR43

MERHTIALQSSIICQQKRQKSEKDRDQFIIKSKGSS

>DcitOR44

MSTVHMYDTIDTRPHHLDDTLSSSCETPVCSLLCRVIVHVCSGKSTCPQSTCMTRL

>DcitOR45

LMASVLETYFPVSQEELDLLAYIYDRKYPDNRLQTTFWIPGIVITNSILK

>DcitOR46

METDISDRRSHVTTMDTDSTDPQTLIQFLTILLEGIGAVNFHRKNKPWKNTLQEVYCYCQHLIIVVGFLLMHIINTSSRSVRYLPEFIQCLYEDVAAITVYLHIFLLKKKYKQLADLIGTMADSFSNADPVIVLKYERRIRTIFRTCFCTLSILLSLMAVEASFPLPDNELVIRRKVYRTNSERKLPCNAKIPFVDETEYWTYRGLFAFQVYMLFLYIFISTVSFALLPIMTTKLVGQYTILCQYIDKLGAQHFDSQGREIFYTDLETNKYAYVYWRTIGETRPKVLYNKWVGHMQCQKAGDSKQDDMNLTKREEAIDVESLGHLKSRRALFSKLESHRIGSSETSDRANTEEACDLESVGQSRAHRPFSKLNNQYQKDYLKQIIKFHQKLLIVQRKVTNFFEQIAFFQTSANFVAIALCMYQLLSLRSLSALRLSKFMSEILFYLGQCYYWCHCSELLDDCNIAICRSIRNSEWINCDAGTKKDIQMLLRWAQRPNLLRFNQGVILLGREHFLKIVKIAYSFLNSIRAFNDKLN

**IR**

>CchiIR1

FSFFFLDNSGWQKFNDTEKKTLEHAVFEDWELVPKVEDGLRNVSKGHLLWSYAFLGSKATLEHIVKNNFENRG

>CchiIR2

MAFIWDSSRLDFEAAQDCELVTAGELFGRSGYAIGLQKGSPWADAVTLAILDFHESGFMERLDNTWILQGNVQQCEQYEKTPNTLGLKNMAGVFILVAAGIVGGIGLIIIEMVYKKHQIKKQKRLELARHAADKWRNAIEKRKKARSRADVQRRLKANGINDTVLPISLSVEELPRSLDLQSPIHSMGLEPIPIEMSPLRTPIRC

>CchiIR3

TLFLLTFISLTLSILLSLSHRYGFETQYDRVSIQDVIMSVLGAICQQGLDKVPNSASGRLTVLVTSITCLFLVTSYAANIVALIQTPSSVIRTVTDLAESPFTVKLHEFQHNRAFLRDVK

>CchiIR4

MDHCNRNDKTNKESKANALDVDNIGGVFVVLLCGLAVAIMVAIVEFCYNSKKASRAECSSPNPQAQSLCLEMTDELCFAIKCRGSRQKPALRRKCSNCHSIREARAIAQPRESICNIPPPPAPLYRNVMQHYLNNSPEEFNLDTT

>AgamIR100a

MRWIWAAVVVVVLAAANLSASDPSTTLSFESLSSQQQQQESDQSDALIATIFATIDHLDIVNGAAgamAAET

QLLQHRTVRWFDGEPATLCGAPPASEHSECFAPPERPTDRHQWDGALAgamAgamLILYGSVAKLDRYACLFEP

AgamTYLLVDEPSRSPLEQAHLRRLLGTLWTTRGAYRVYVRARGQLYGYDPFRRPAgamAAEYGALVQLEDGRP

LPTVPLTDFGGYPLRIEVFRSVYSNPDERASASKADQITYSGPDVMARDVFCRALNVTVIPVPADRDLFG

DRLPNGSFTGALGRLVRREVDIVFTGFFIKDYLARDVEFTAgamLYSDAVCCLVRKASRIPEALLPLYIFPG

DIWALLGALGLACASVWALLRWCVRHVKPPAgamGLWSRRHRLAVLFNLPRSLRAAgamPVRRTVQLYVDSFIL

LVSAPYQRFTRSGVERLFLTGLLLVSLIFVSLYTSGLAAVFVNPLYYPDIGTLQQLDESGMAIPVKYRGF

LDDVFAVNYSRLMDSLRARMQHLPAKESMLARVARLGNIATVTRKTTLALDNAIYLTTRQLHMVPECPRT

YNLAYVMPHRSAFGEQFNKVLLRLVGGGLVDHWIEQARYGWTLRDWRVARRMMESSFKVLTVLDmelQFAFY

VLAIGLAVSVAVIGAELVHFHRAHRGDGHGVNVLLRK

>AgamIR100h

MPEAVVPCCLLLLLLTLPLSAgamSLPAgamGVDLIARTPYSLDAgamSTVPVRERLIALERRLVQQQQPCTRLLI

VFVQLSDGRPPHLAgamDTLSLAIDAFAPGHRTPRLVLRGDDEAAADALSSAATLADRDSYCVVLILLLPDL

GNSLPGTLLQTTARLFPLALKLLLLGSAAPTDEELSAARTLLAALWQTERALRALTIWYRPATGLLIGAY

DPFEPPAgamRFYTLAgamPNRTDLPTDLAAFGRRMNLRGSAITVYGFEAAMAYRRQDIDmelLPRTFPARRPGST

HASRDALLDALFGADVEAVRELARRMNFTPVVHHMRANFGFKMANGTFTGVLGRLVARDAWLSMNVYFLK

DYETRDLQFGAAVYQDSLCVFVQAAgamMLPDWLLIFRCFTPTLWIGVWSTVAVVSLCYLALTLLIAIYRPA

WEHGRQHTGNVSPDQPQQDLTGLVGQIVGALLTAPTNGFHRTTTHQKLFVAFGLIWGLTITGAFQGSLVD

VYTTPTSMKNLDTLAELDASGLSITVTAPALIVDVFGTERPGSTLGNLKRRLVIETATNRSAAYGVLGGR

TAgamLIRNQDFVRLSTKYLGRDGTPRLHRMRQCPRSYTLAYLYPRGSPLYRAANDHTLHFLQHGLYAKWQR

EAAHIVRANRALKVQRYAgamQAgamRTAgamQAVTLRLDHLLLPFMVLGAgamFALGVVCFAWECRALLTAAVAVRN

>AgamIR100i

MSPTADNKLNIFNGIPIRCTVFPRNPTLLPWDSLPASFREVHYVQQSVRASNGSGGLDGMLLGNLAVALN

FTAETMDASDGQEYGYRLKNHTFVGSLGDLLQYRTDVSFNVRFMKYYDTHGIEFLHPIYSDQLCILSPKS

LEIPQWLAIFLCFHPYVWTSFVVVGFAgamGYCWYLLKRWTLRKVSRYRQHLLKGDRAQYTVLSIEMWLVLL

GASSTYLPVRMIERTLLVAFLIANVIISGTFQGTLTTAFSTKSYYKDLNTLAALDKSELPIATSSRSLLD

IFGNDSLSPLYQSLKGKLQILNESARHRAAFQRDVCCIERHSDVHLIINTEYIRPNGQPMLHVVDECPRV

YSLAYIVRKGWPFAPLFNAAIYRFVESGLCMKWYEDTETALILQKRIRQLREQEEEPALRKLTMIDmelQTS

FYIMGLGMLLSFSVFIMETFVGRGLKCSQL

>AgamIR101

MKQACSCVLLVLLWQRVAAVLDPLRHNDCHSRAVSMETRILDDYLADQHTVTVIESCTEIPIMSPYCMHN

PVPLNAFTLHRFQAFVHQSLEHTTQELEYRTNECFVGSGHMDDLLTELLPYLSEYNPRAKVMLITQEMAD

QELAELFHAAWYRYRLLQLIVLNHRANDTIESCLFNPFRKALSPELRYLPTGKSDLHCRLLTDGQQLDAY

NRELNLFIDDRVYNLNGYPLNIAMHVSNGSTSAYDCILGTVSFTDIDQEILSIMQKQMNFSLILHKNELE

LSIGYIHQNGTPVGTLGLIEQNAIDLAANSRIIHNYDTRNLLYLHYISTEKLVFITPRNYFRNRDKTQVF

INPFSVAYMLTNVLLSFGVPMIIFLLEYAACRLDVPREPSSHTFGTKVLNLVGIIYNVSVKLPRADRKRW

IIVGLLVYNIVSYPIWQGVTIRYLHPSNQQVNNINSLEELIETELELKVSHYHEHIVRHEGPHFQSPVYS

ALSGRLSTRNTSSLRDSIEQIIMHGNSALLIAEEYVPLVLAgamNYQWIPGKPDGIWPIQKPIYEFYKSMAV

PKTSPFAgamTFNAIVLHCVEAgamMNDRFKHQLETAVQLMQIRRVREHPSVPDYIVFNMEHLLPLFIFYFAML

LLSGGIFLIELAVHAFQASRARSQQRAPLRVPVEEYVPFEFVH

>AgamIR133

MTGPPRSTGPLAAAQSAHLETLVNITKGIHKIETPSAKYELCLVTHATDPYWSSVVVGYLSHFSSYPRVF

LNAVQRNVQLHRCSVYLIVAPLIEGKQLYGLLQNVSAgamRNWNPAAHYIVAINDRPTLTSLVNVFRAFPPL

GIRNGAVVRYRRGIIDTLVCDYNTSRVYVVTGTDAKHRSWLTHDRNRNLDRLPVRIKTSMEFPFLIVSQK

GIAgamVYRWFFVAFAKHINALALFDRQPNTTRYEIALKIHDTYRDTLKPVAFGSFSGDCVVLPEVSKRGLF

YFLLLPFTRPVWLMCGLLATGSFLLNWLQAQRFPNALLLTLLFGDQAQRYTLAERRLLTVGSVLLFTLTE

AYWGKLHALFMVSLNEPHLRTVEQFLRTSVPLEVVHRDAASYYHELHRHRQLIVPETDAQHMDNVQNGRC

ALLMPCSNARLLLHLLLNVNPDVYRVPYYPLEQPVRQHLDGFSVFRYSPIADQLVQFVGTAQQAgamLAHYW

RTIYVHQLEGIARRRILVQETLGWGELASLLYLLFAgamYAIALVAFVLELWSDRFRRVAPNA

>AgamIR134

MALQAQSDDTLSQSVLITLRHVTTRAEFLERPSAgamQELCISTSATHPHWNDVLQAYLHEFPLYPRVLTSI

EIMSDVKLPKCSLYLLFEPNASARQLFLLLKAISSNSNWNTPAHVILLTGWIERPDQFKTLFKVFYTMGM

HNVLHLVANREGNDSAIVACIERGFIQSFRPGSPHYRQLLADRTSNLHQLPLVIEKKVVYPYLMYTRDTV

SGVYRQFFDAFAAHINVRVTFDTTDNQILLGIKAPNALSVPIAADGAFTGNCLVVPEKPKAgamLIHYLLFP

FTAPLWYLCCCLAgamATIVLNCRCPARFGHSILLTILFGDQDTRNSYGRTERRLIFFAVLVMFFLSEAYTA

RLLSEFIRSLNEPHLTTIRQFAASGITLELPSVQRDRATLLTEELRHNLLVSDKRTYLANVRRGQHALLL

DCGNAQHLVHEILNESEDEFPVRYYILQEFVGWRINGWSVSKFSPARVQLTQFAgamRIAQAgamLWNCWHGLY

VKRIHSLRSRSTRYRKTLTMRDLISLHYLLLAgamHGVSAgamVFVMEIVTHFALIACGRLRKSLIKAKSQLQR

MRH

>AgamIR135

MVPPPFDWALLLVRFGLLAADSSPSADAALASLAYLTAHPGTALERPAAgamQELCIAPPPDNDPYWNDVLE

RYLHHFPLLPRVLTRTKLGDNIPLHRCSLYLLFEPASHARQVFLRIQFLSTNGNWNPSARFAVLINARSH

TSEIFNQFENFDLMGVRHGLVLMSVPEHNRTFTMLANCRQRTLDYVMDVSDVGELLANHSSLGLQQSEPI

VVARRAEFPFFIHDRSRIEGVYCKFFTAFARKYKARVVFGDRNVQIVMTIHNRETLTQPYAIGSFTGNCV

IVPERPKAgamLVHFMLSPFSSPVWYLCCCFLAAIFLLNWRWSHRFQHNILLTVLFGDQAADERYSLPERRL

IFLAIVLMFFFSEAYSAKLLSTFIESLNQPRIRTLRALGASDVPIGVLHYEDVEGYEQLHRNLEVLDEPE

YYRNMRAgamRNAFLLQCGNAEYMLHKEMRDTRVFRVPYYILGEMVGWRMNGFSVSKFSSIAgamELVEFIGRI

GQAgamLWEYWREQYVQKLRNVARKELSQRETLLMGDLISLQYVLTMGYGSAAIVFGVEVMMGCVRRCKRTR

RTVVPLRAVGGH

>AgamIR136

MSSPNSTFVQMFANERSTTAALLISVLVRIINKTDRFGSNAVALFNFNTHCMDHVPDQLLKQISTITLLN

YDSSPLIKDTFERPNFFLHIHGYKVAANEQVIWPFALDLWALSGGLAAFYVWYLLVLAPHLRQHRRDmelYD

LVNTPLHIFRIVLLFLLTEYYTALLTSSLGLSQVPFYPTTIQEFVKTPTPLLVLRRDLISILQENKDFAR

KMIFYENIEQYQHGQYALTQLCDmelFMYTIGSITKYLGKEMSYRHYHLIDEPFTTTVTMVPFGKLNPRLKR

FQMYVNRLNEAgamIWTYLVKKWNLKASGTQVVYEPDDVDALFLSLEHFVPRTETKAIHYAPILVHAFGISK

PNGPSEESPIQSEVLNILEYFKLIEKTKNIIVLLDAYGVKPTELDLLAKTYHHFGAIDIIYVLLKMKEPI

VIRLNDmelSTEFVKLSTFARIEQLFPDRLANMSGRPYKVACLENPPLSFRSPATNRTIGIDVEFIDmelIAKH

QHTVADYRYTAQPIELFEPWHSTEIDFATYRIILTEQAYKFALLFLPNQNLWCLAVPKTYNRILHQQILW

PYTTDmelWLLIGTIVICFLLIDLRQDGCQLQSLNTPIVIHAVTSFQWMKNKLYILKDVLDTLQHFGLYGKS

KKLIVLIDmelNRVTMAELKVLKESYQHAgamAIDILYVLEHSNFNQLNIMIPTRHNRTTLVHRSAESSIEQLF

PDRLSNQSGQPYKVACIENRPLTYRDASSGRIIGIDVDFIDIIAKHQRTVAQFKHTADPIKQFKSWYDVE

FDmelATYRVPDGGLAYPFAPLYFPNQFRWCLAVPKTYDRVIHDQVIWPYQPPLWAMILSLAAFLIVYRLFL

RQPIQHQYPDVFPAIDTPLQLLRMLLLFLMTEYYTAKLTAILGLSEVPIYPRTLAEFSSSPIPLLASHRS

GYQYLIDNPQVHAKTIEWNFSAQYDPTGMALLQLCDLFPFTIGDTTQIMGKRLSHHHYHLIDEPISTSIC

ISPFRKTSPRLVRFQQYVTRLNEAgamIWDQLVSKWMLKDGRVSVAHGADKRTSFRSSILELFHFVPVYVIG

GYLYTSALIIFILEHLVYRLQQRF

>AgamIR139

MSSRKIVLVSLILTFYSVHTLPKRQAgamIVDYLAHMAAALQMQHFGVFNCWLLQFSNGTNLSPLLTSIVQR

LSNEHISLVQADHSGRHIPTHQEPNMVIMLWGNEQQMLDQFYINQWLWEIPSDCRTIVLFELDASGASSL

PWQIGEYFESLMLYYVACIAINKNALFSFHYQPLRIVSHSCFPDLSELFFDRLQTIQNKQFAAgamYMKDHY

TAMYCGQMYGEDTALFLLFVDRLGLTLQLQEVLCDGFESLVSCVARYNGIHFLLNRLYFTQYNKHVISAS

AMEQFTIATPKGRLLTVWEILLKPFHHSAWGLILGILVTLQLINQLKPTLFSNNLLALALFGFEKHQLRL

TKRVEKATAFALIVLFFQLKCAYEAKLVSYIAEPPRLPDAASIEDLRERNIIVHSRKINIMEDDKLNGIV

EFYDGVHFKFDGLTLLENRVALVLEKLFADSVEGHGMLYTILQENVYETLPFYALGAKSLLRRRFQTFQQ

HVFEAgamMQQHLRQKQASCLIWYIVKSKKIPGAFDGSSAVIRFDHLKPLMLFFLGQWVLEVMVFMIEMLVE

RYKRSRRV

>AgamIR141

MVHRSAPHHINSTYVAQSIPMFRNASTRIRTYRIGSGRHKVDLHFQLITTNRFNQRLQGWYSYSLPLFHE

HLTMYIHYTHLSYHEQSSQILSIVLLPFALLGLLLCLSLLLALRTGNSYRSDRNQSRPVSFIDTLIWMVG

VLAQQGSIIRPNSSSSMVIILVALYMSAIIYCSYLTKIASLLSVDASVNLDLQTVLSAgamQYQIGFVGNNT

ADETVIQKRYDPVMNEIMRRMVQNSSLYSLSHEHALHRVLTTKYVLIGNVGSVRKAMQTLDEQQNCNITE

IELTGIEQMIALQMPSFYAYRKIIHYE

>AgamIR142

MLLKRGLLNYCIIHTNTHGIEPSLLKYDTIANHFQSHDLNTSIELLFPPFLRTLEGHKLNALLSDNYPYA

YLLSSVWEGVDAYILTLARESFHFEVQLINMAINRQNALHRMDLIRQRLENGTIDIHMPRAHVSRSYLNV

DVAAAYEWEALVLVVPKSDQLNLINIMLQPFTIEVWTIVLAYLLVRQMIKLFSFFKRRCNRFTLKKTLTC

RWSFGSFGSLTTVGVELVSFLLIEAYLAKITEFLLYCRFRSDPQTLDEFFRSTIPVLVPEYMDPLVEALG

PTVAANFHAKLIRPDEYAKRAASCCARIHTLPRAEYVVRMGKYFDATLGRKQLYILPEQLTIIPMSYLVG

RNFAFKNSFELFLLSVHESGLIGRYVTAHRKDmelAMMERNFFTKGWLTLADLLPLFVLVGAgamWCCSFAAFL

SEVFGVRFNRYWARKRVRPFVAE

>AgamIR21a

RINNEQPYVLYTHELYIDGLGANRPQVLTSWIGNKFSRNNVNLFPRKLRKGFSGHRFTVKAAHQPPFMIK

RLSTDGVGNVNIRWEGLEMRLLRVMAQYLNFTYDIIEPGRTELGPGDAVVEEIKRGQGDmelGLAgamIYVTIE

RNLATEMSVSHSTDCAAFLTLMSSALPRYRAILGPFQWPVWVAVILIYLLAIFPLAFSDKMTLRHLLGNW

SEIENMFWYVFGTFTNSLTFQGENSWSNTRKTSTRMLIGIYWVFTIIITACYTGSIIAFITLPVEPERID

GIEQLSRGFFRVGTLDRGGWERWFENKRYGLHVSRECFALYGVSMVFPPNSVHRDPINNAILYMQEAgamLI

GKLNRDVTWETMKTKDGRRKEASVGEVLRSTAPSERGLTLADTEGMFLLMLFGYVVALGVLISEWVGGCT

NKCREVLKERAERLKAAAAEIAAAATAgamSDNGSLPVSSPTSTNRNS

>AgamIR25a

LLPVFVNEVDNNLANVAVEVALNYVKKNPQLGLSVDmelMYVEGNRTDSKDLLQALCSKYGQSLSENRPPHL

LLDTTLTGVSSETVKSFSLALGIPTVSASFGQEGDLRQWRDLTPTKRGYLLQVMPPADmelIPQVIRSIIIY

MNITNAAILYDNTFVMDHKYKALLQNIPTRHVITTIADDRDRASQIEKLRNLDINNFFILGSLASIKQVL

ESAKNEYFERNFAWHVITQEQKDLTCNVENATIMFLRPMSDSSSKDRLGSIRTTYNLKQEPQITGFFYFD

LTLRALIAIKNILQSGSWPSNMKYITCEDYDGTNTPNHTIDLKTAFIEVTEPTTFGPFEIPKGGKMQFNG

NTYMKFDmelDINAVSIRSGASVNTRSLGTWEASLNAPINVANEAEIKNLTADVVYRVYTVVQAPFIMRDPT

APKGFKGYCIDLLNKIAEIVEFDYEIREVEDGKFGNMNENGEWNGIVRKLIDKQADIGLGSMSVMAERET

VIDFTVPYYDLVGISIMMQLPSTPSSLFKFLTVLETNVWLCILAAYFFTSFLMWIFDRYSPYSYQNNREK

YKNDDEKREFNIKECLWFCMTSLTPQGGGEAPKNLSGRLVAATWWLFGFIIIASYTANLAAFLTVSRLDT

PVESLDDLSKQYKILYAPLNGSSAMTYFQRMADIEAKFYEIWKEMSLNDSLTAVERSKLAVWDYPVSDKY

TKMWQAMLEAgamLPNSLEEAVQRIRNSTSASGFAFLGDATDIRYQVLTNCDLQMVGEEFSRKPYAIAVQQG

SPLKDQFNNAILMLLNRRELEKLKEQWWKNDDVQNKCEKPDDQSDGISIQNIGGVFIVIFVGIGMACITL

LFEFWYYKYRNNSKVIDVAESTDQQHGGTIVKNVRPAgamKLMKQDSLKDSTKGHNYQNLRTRTLMPNLSKF

QPRF

>AgamIR31a

VLDFACPGAPNLLEEVSKHRYMNNTVSWLLLSRNTTADLLPRVLWNTAgamIQMNSDLVTAIETTDHRGVFN

LFDIYSKGRHLCKDIFQTLLGTWSADGGFRLTPNYSPYKIRQNFNFLQLRGVTVIDRENVTSGKVDQLLG

EPGLTKGIVAFVKYHYALLVVLRDFHNFTIKFRPTRGWAgamRLRSGYRLGLLGVVQRHETDVAATGIIMRL

SRQPELDSIHYSWAFETGFIYKITPDIGSKSEGNGFVAPFSLPVWVALLLSLALSVLVLQYLARLSADER

NTRATMAYVLDVMACVAQQGVPNVSRLVPTRVAVIVLLVANLVLYNYYTSTVVSGLLSSQMIGPETIAQV

IDSPLLLSLTDTGYHRILLREQTLPYSTRMHERKALPPRSPNDLPLFTDVEHAVPYLRRGGHVLHCELTE

VYPAIANQFTANEICELRTVEGLYRYDIRVMAFVLPKHSMYSELFKITLMRAQETGIVKRIYRIHKIAKP

ICQGSATVYSVELTEVSLAFIILGGCKRRQKTR

>AgamIR40a

MGVGSNSKYILALVLLRVALVWGAFPTQRNLIALYERSNQSGMIRGISEMVNLLAPKSLVILVQNETKID

RLDKLTVMIHHHNIPTCVYYDLEAYFSLIEENLKKSLEITSLIFCHPEDmelLQDITDRRLAHRLSLFIFYW

GAAQLPPTLNPNLLMEPFRVAIITNPRRNIFRIFYNQAKPNNRGDmelLSVNWFDGNDmelTFKRVPLLPSPTE

VYKNFEGRIFTIPVIHKPPWHFIVYGNGSASVGDNQNSSSSDAAgamGFELELDENVTVESDDTYFTVKGGR

DHNLMQLIAERMNFTFQYVEPPEKIQGIALGSEDNASFSGALGMLQRREVELYLGDVAVTWERMKAVEFS

FFTLADSAAFVTHAPRKLNEALALVRPFQITVWPPVIITILISGPILYIIISTPYRWRSAQTVHARNARW

RPTRSRLRKPAFYNLRYIEEMSYTRFRAERTSLINNHHHSRGQDYPSLDRCIWYTINVYLRQSANIPFDG

HLARFFSILLWLCATYVLGDVYSAQLTSQLARPARESPINTLGRLENRMNREGYQLLVERQSAFHAALVN

STGVLQRLYRLTRQRSVNDSFLVKSVEEGIRVLQADPKYAVFGGRETLYFNTKRYGANRFQLSEKLYTRY

SAVAVQIGCPFLDSLNEVIMRLFEAgamIVEKITIAEYEQMFGRQKGGVSHAEETVRTVKSTNSECDTDGTG

SGKRKTDSNDKLQPMNLRMLQGAFLVLACGHLLGGKCYTMAI

>AgamIR41a

MNYSVELSELTQGELFHFELNRLLKWIFLQHLVSFFCTCIVVRTGSSGHWTAATEQFPHPVMVVALSRND

ELDVLLDAIENGCQTFIVAQSTAIEFLDAFRYVHDRATVRYPHKRVIILTDDADVQWRRTVFEHGAFRDV

MDWLLVHPLPDGKRVDLLTTGYGYDEWIPLASHDTSVPGVLHLTVELFPNKRTNLWGRYVRLAIFNYEPY

TLWSAVDDSNDGNAFYQHNRTLFIDGTEARLFVEFCAKLNCSLEISLDEAgamEWGQIFDNRTGDGIIGAVV

ERRADIGVGALYSWYHESLYLALSKPISRTGVTCIAPKPLPLSSWMTALLPFSTEMWLAVLGTIAVSTVC

EMVVSFVTQKFNPNKVHRVDACESIMAIISIFILQSVLIRTTRNPYCSQMILIGSILFVGLMIGNAYSGG

LASVMTVPRFEKSIDTVQDLADRNLRWGSTHDAWIFSIQLATQPTIVKLLESFVTYPKDVLHEHAKQRNL

AYSIERLPYGHYAVGDYITDVVSSNFEIMLEDIYWENCVAMATKTWPLMDELDELTLRIFQSGIQRYWEL

EVVTKFADNKVQHAISTSRHFGNPGPIPLQPSHLVGAFFLLAVGLGLGTVCLLLELLWHRLTATNDGAgamM

ARRSETP

>AgamIR41b

DGTEILMVLELCRRHNCTLEIELVANSEWGQVYPNGSADGLIGSLIDRRSDVAVAAIYRWYNWYQYMTMS

AYTGRSGVSALVPRPRLLPYWQTPFLSFPPSLWLMVAVSFCVGTVAVFLTEHARHHIRPLSGSTSHNNRL

IDSIFFMVSLYVEQSVPLPNSLLAgamSMLLTFLLFGGFMIGNSYAgamALACVMTIPRYEKSIDTRADFAASG

MKWSGPTVAWMNSLLMAEQPELVTIRDRYEVHGGDTLARYSHTRRDmelGYVHERLQYGSYALESFINLNAT

RLLQPLKEDVFWEQIVTACSKTWPLMGYYDDLILRVQQNGILRYWELGSVIRNMGLEIQRNLANARVQDS

DHEPVKLRMAHFLGVFFILFVGLSLASVIFVAELVVYRANS

>AgamIR41c

MVNFLPLDNFNTSLQFLLTHLLRLYYSQHYTVCFIRSFHDNIPFTTGEPPLPLVHIVLEDEVLTAAAPPN

GTAWATKKEEFCQKLLQAVDTNCGGFVVTESTLFPFLEHFYEVHKRAKLRPSPKHLIAMTTSATFDRSRL

LQYNQTLEALVNLLLIVRSSSDKGNHHRKDAFELHTTQLLPNYPLGVRIELVQIGKIVLKQDANGVVEFE

PKHDTEIDFFPDKVRDmelKGRRVRVSTLEYVPCSVYKKVPIGQGNAKCADDAAHEFWLDGFELILTMEFCH

RHNCTVELINQTDWGEVFENGTTTAILHDLVEQRADVGLGALLAWHAWFQLTTITQMIGISMISALVPKP

RLLPFWQTPFLSFPPSLWLVVGITFTAgamTLTVFMVSVARLRILPPNVDDRQRQSRSQHLLDALFFMFSLY

VEQSARLRKDLLAAAILLAALLFGGFMIGNSYAgamALASTLTLPRFEASIDTVDDFLARGMRWTGGSAVWI

YTLYLATTPAMIRMRESFVVIPDSAERERLPFTDPRMGYVIEGMMHGSYGLGTPLALNASLLLQPLKQPI

YLDHTNGFCSKVWPLRDAYNAFILEVHQCGVLHCLELTSLIRHYGLTVQRNIVTARSQDNGHEPVALSID

HFLGVFFIYFFGISLACIVFVVELVLHRWNARF

>AgamIR41n

MENQNPTHSLLHQSIIASDDIVGLNSLVNYLVLTYFAHFFGICLIIARNDSFHHSAPLPTIVLVIQDNDD

FEKALGVAVDmelGCQSFIVTEHAAgamAFFDAFLPVHEAALQRSMEKRVLMLLESSDSPFLSTISQLLLYTLD

ISDGNPVTISPRVIDVIDTRNDSFPNTKSVNHFPDKFANMNKRRLRLGTVPYLPNAFIEDKPLGEGNARY

ILPAKPNVSAMISGTELWLVVLFCEIANCTTEIMIASEWGNVLDNGTKFGLLGAPAKREVDITLAgamLYTW

YSSFQHLAFSAVHSRSGCTCIVAKPRIIANWRTPFLSFTGSLWGAVLAAFVAgamAFAVLVMSRSRQRILQL

GEATRYTFSDSVLIMIGFFMEQGVPMPNELVASCLLFATLMFAgamFMIGSSYNGGLASTMIVPQYEKSINT

VHDLAETRTTWVGVTVNWLFSIQLAYQPDmelLTLLTTFREWEEGEISRRAHERTVAIIVERMEYGHVAHPQ

MELDAMKGRKMMAEDIYWESVVGMCSKTWPARARFDRLVLDLKAFGILAHWELIGVARYLSFKSQQILRY

SRETGGDEFTPLRMANITGALLILLAgamLSLSLVVFVVELAWYWCGPRIKRCVADCLIKCSMRFARKRARP

TGREV

>AgamIR60a

MLLALLPSSVNGIVYVRIRNERGPTAARAgamIQCLELLSHTYFNMRETTQIENIVIFYLQNLSSPAREILL

GYLQLHHTGYDIHEDDDDDEDGEPESQFQLKLMSDSVSSDDLRRAQFMDHKQIDYYVIVIDSFEGLQSAL

DRISATAAFNPRGKFVVLYNNPNDRDSNARLANRALGHLFVGHHSVNVLFAFAIDATSYHVYTGDPYHGA

TDCGQMKALKVATVVNGSFASRALSTAMINIPKVPPELESCTFLLCTRVAPPFIDLDCSRGLELQIMDLL

RESMKFKVNVSCSTMDRGELEPDGSWSDLLGLMRADECDIIAgamGFSPDFDVHDAFGSTTLYLQDYYTWFV

PAAgamPDARWKLLVYIFEPTTWEAFAgamVLFVSALVWRLVAHYLPELAAHRELSMCFLNTWSVFLCISANNR

PECNALRLLFMGLTLYALNVTTIYTSILITMLTNPPLAYQMDTIEEILASGVPIGGRLDSEDWFINDFAD

DRLVSEQYNATSEFQPSLDNLQAVVEGKRSLLMSRLYVRNTKYNGLVHGLSRDVLVTQIEMIMEKGFPLL

PKFNRILSNLIDmelGIMQKLWNDFLYNVTILDRIRAHRALSEADIIAASPEVVLTLDHLQSAFALYGIGMC

LCVVVFLLEVLSKTRW

>AgamIR64a

NPGFISGGKLKIEPLGCYSPAEGLQIPKRESTVVRRRNMDGLRLKIMTVVTQKPHQPFELYLTTPQNTHL

DSVHRYNYGLMGMLKEFYNFTFVNRRTKSWGYLRNGKFDGMIGALSRREVDLGGSPMFFRQERHRVVSYT

TRTFVERPCFIFRHPPRNDAVKNPFLLPFEIIVWYLMVGCGSILVTVFWFLATVMLLLLLLLVVAMVHSR

HAIAITRPSQQTYRRRIDSIRETETLAPNAIIKRKRYPKAFYSSSEALAVLCIPIKLVSVLRNHTTTVRR

SDAPGGAPAFFLVLFSFHLTGLSEIPHLSSGRCTSFFILLFGYLMYQYYSASIVGTLLMEQPKSIKTLRN

LIDSRLTLGIEDIPYSRDYFVVSDCFISMDPAKIVLQKHTRTNQKNRLPHTPNYQRTTDADSLELHRTRI

EYYEPKTGRNESNFLAAADGLQLVREGGYAFHVAISAAYRIIRLTFSEREICELSEIDmelFPVWSQWMVAI

VQKNSPYRDVITYGLRRLNEAgamLMQRQRHVWQEAKPKCVRQIAPTDLIVGLDAVVSAFVLLCGGICLSVC

VLFVEIL

>AgamIR68a

AFVLILGVKHCANKQLNSATKGNDRKSSQSIHHEEYSTELHLEMLLLELAAKMDYGHCYVVLFDEVYESV

LNAAFFRQIHRAARYIVKIEQDEDTFNPRPSLKCILESTRKAgamCGGYILLMANGIQMALYELSTAPFPMQ

IKGVFFSKILNFWQGGKFRLANSTFFDDKTKDLRRQEMRVVVLEHTPAVFKSATTSNYYGLEIELLKAIS

KAMHFQMVFYETSDADKERWGRLGGNGTLTGIIKEMQEGKADFALADLHHTEYNLGFMDLSVPYNTECLT

FLTPEALSDNSWKTLILPFNGEMWAgamVLLSLFAVGFVFYAFSNTLMLKWLRHKKPKTNMSKSSAYDRNKL

KKLRMIPFKRQPEPWHDPLPANDmelFDTFSDCIIYTYSMLLLVSLPRIPEKWPLRMLTGWYWVYCVLVVVA

YRASFTAILANPIPRVTIDTLQDLAESSVRCGAWGEQNRLFFQMAQDQYSQTIGAKLEHAPNQNEAVEKV

SEGLYAYYENIYSLRQLRSTRKSEKARQTLHIMQECAVHMPISIGLGKNSPLKHQVDLYVRALIEGGLTR

KWLSDAIEQFQSNVEIPPQEAIIDLKKMYAgamIVALCFGYVIALFAFVVEKIYWRYYIENNPAFDK

>AgamIR75d

MPYTNVVLAFLNVSDSWELFELYKPYRKARLSVVNIASNYLPAVDRYGSQLIVRRKLVDRRRNLQGFAMP

CGTATTSPEYFTGMDDRNDVHDLFTKANFPFIRELMYDLNFTLNMVQLDKVGYKQNGTFSGVMGKFQNRS

IELGCLGTLMRTERLEVADFMIVTLIIKSSIIFRQPPLSIVANIFELPFSVGVWACCFALMAIYWMTMIA

IRQLTSGERFGAIESLVYIIGTMCQRGCDIVPQFNGTRLLMFSLQLTSFFILTSYSASIVALLQSPSRAI

ASVGDLVRSPLKAgamVMDTSYGRVYYQETQDPDVQELYRKKIKPHGEKAFLEPNEGIGRVKQEMYAYEGEL

NAAYKLIKETFAPEEVCKLQELEAIKLPPFGIPIVKGSKYRELIRQRLMWQREVGIIKRFNLIWIHQKPQ

CENLNAgamFSSVGVVEMRYLYLFLAVGFLAALATLLAERSWWSGMAKKLKSRTAIIRRKNEA

>AgamIR75g

MKWQQLKVVYVFHCFHQQDVLIHQLHSAMKNHPASMSFLNVASFSGSKFEASLQSLHVGVGLMADLNCDG

LAgamVLKALSIGAWSTSAgamLTMWDKRSVYERRQNLLGMQLTGIAKATDDEHLLALVQKQLTQADTLYYDmelA

EGLELILRGRVAFLCDAHHAYQMMQTHFTDEQRCALQEVVLISKKSTHLALAKDNPLRELFRVTVHRIAgam

NGVMQYERSRCYADKPRCAENEVKMPEVNLDQVSSVMVLLLGAIVGSIAVLLLELTHSRVWPRRHRVVPS

RGINRKL

>Agamf.1

MFYERYKTAITLNLECSGMESVLSHLSDNAYFNDTFHWLMFGGRNFEQVTCLLSAQNINYDASIMLVFDR

GDRADVPRIFEVYDVRGTVKRRGGRVSFDLLGTVSSLNQLPKRRARSQDLEGIELWTALTTISKHQPRPL

IEYLNTVKRQTSYTATIHSYQLVKLLEMKLNFKLIVILTEDWRFDLIGKNSSRGVVGQIQTKQVDFATTP

FAITPERIAIFEYTIEIAHGTFYTVFRHPKSLNNSNIFMLPFTNIVWLAISLIFGAVALLIALLIVCIHR

TGRSRSTLDWLVEQSLLGTLGMVCQQGIHHRIIRWSSNRVLVLVAMCSSMILFQFYCSFIVGYQLITPPK

TINTLEKLVDSEIQMTVENLSYQHDFFRRTNNPTALKLYETKILPNRYGGFVNLSFGMQLVRQGGYAFHC

ETSYGNALIIETFTEREICELQQVQLYPQRPVHLPLIKGSPLRELFKVNLQLIKESGLLAYHHARYYTPR

PKCNKQSSTHTEQIHLADVRFAFVLLAAgamMAASAALLGSELVFLHLRTWWHEQQIRAIPAgamFRWLN

>AgamIR75k

MDKSRYLQFLVQSLIVLKVLTVSNGTPQATAVNPLSPQTQTDQRIAAVRDLLVHLDRAHQLLVLTCWSPS

VRYALWQSVRESTATSHRGTASVRFAPIDQRHLPWHDPNQHQIVIVLDLSCPGTDRLLESARQLLYYRVR

WIVFRSSIEESGGSVRGWSNCSEYSVLDRLPLLVSNELFYFCTETSTGQHLVRQQYRLAARSASSSVPIY

ETFGTWNAALGVRVVVDSKVRPPVTSIRRQNFHKFQLRASLVILHNETLNHLDDLHDKHIDTLSRVNYLL

TKSVAHALNATVKFSIVDTWGYRDRETDRYNGMIGELQRDLADLGGTSMFFTQDRIKSVDFLAMTASTRA

SFIFRAPKLSFTSNVFVLPFDQYVWYCTVSFIVLSGVLLLVMLRTEQRYTAgamGRGGRGSAIGSNSPAAVT

GLSDTLLNVFGTTCQQGSFIEPQTAPSRCLILLCLVVLMFLYASYSANIVALIQAPSTKIQTLEDLLASR

LKTGAEDTVYNQYYFRTETEPSRKALYERKMRNKDGTENFLPLAQGVELIRQGHYAFHVERGVCYKLISE

TFQEEEKCGLQEVEYLKVIEPYYAIQKNSSFREPVRINLFKLREFGIQGREHTLLYTKKPRCIGGSSFIP

VSIVDVWPALVTLGWGYLLTVAVLIAELLWFRLRSRILPTAgamYFQ

>AgamIR75l

MIDLRTHKVTSVRRKDLHGHYLRASMVITNPDTLNHLTDYKDKHIDTITKVNYILTNCLVAYLGAEVNYT

RVATWGYYNTTTGMWDGMIGELVHNTADLGASPLFFTTDRIAVIEYLAMTSETRSKFIFRSPKLSYTENV

FVLPFDDKVWICVIAVIIVSSVLLLVTLWAEWRITNGDLDmelPPAPPDSSTMAASLRDTLLMMYGASCQQG

SAVLPRSCSARTITMLTFTVLMFLYASYSANIVALLQSPSTKIQTLEDLLASRLKFGVHDTVFNRHYFTH

ATEPTRRALYEQKIRRPYGPDAFIALEQGIDRIRHGLYAFHVEQGVGYKVISETYQEDEKCGLQEIQYLQ

VIDPYYAIQKNSSYKEMVKIGLFRLNEHGIQYRENDKLYTKKPTCSGGGGKFVPVSLVDVEPAVWIILWG

SGLASGFFLTERLYFRFLRRKVRQLVRRWQHN

>AgamIR76b

MEKFNFTYELVMPEQNIVGSSNDmelAgamSVLQLLTNGTADmelAVSFLPILADARQHIRYSTGLDEGEWIMIMV

RPMESASGSGLLAPFNRDVWILILVSLLAVGPIIYGLLILRHRLTKDKEQIIYTLPHCVWFVYGALMKQG

STLSPTGDSTRILFASWWIFITILTSFYTANLTAFLTLSKFTLPINNAEDVRRKEKQFVTIRGGAIEYAI

KNRDEALNALSVLVDKRLVDFTTNVNDSDTLADKVAKQNYVFVRDRPAIDHMIYADYLVRRKINPKNERV

HCPYATATTPFLKRNRAFGYPPNTEWNRIFDPELLKMVEGGIVKYKLHDRLPKAEICPQNLGGTERQLKN

RDLVMTYFVMVTGFVTSIVKLATERISYLGKQFTTGDSPPPPYAEVFSRHQQQQQLGVLGDSERTGKLFD

DGSGGLFGAgamAgamGANRQMINGRDYMVVREKNGLGSRLIPMRAPSAAIFHYTYAN

>AgamIR7i

MKVLLMVSLCCIATSLCAMVPVSDHHRNKYHHLTVPIAHHFKDTNIPVVFWLDSPVYVTNQSTQLDALHA

IVLAHSDWMVAVFRSSLRCMRCRTRLQNVFIAATVRSLQTFLASLEYDCFYPSGRYIFIVTEQLAREATD

VRDVFEIVWKNRIVHVVLIVSRSNDTARFRAYGYEPYAYGKCGKVRVKLIDRYTDAgamWRRLAgamGWFNCGL

PNFNQCPLKVATFESKPFVMVRTVGNVTRYSGLEVKIFNHIAAKLNVSIVYTPPPNNTRWGVLLPHNSTG

QMGMLQRNEADVGFGSVGRSIERDLYLRSSVPSIVSQLSMTIPPRLPYTALEKLFLPLRPSAWLLVAAgamY

TTILCLYVVLFRGKHRPRRERIPGLYYTFWTILMGGPGREVHRHSTRLYVISLVLNALIVRNLYQSALFQ

RLKSNDLMAANLHTYQDINKAgamLSYYMFRATVRFYADNPEVNGSIRTIANENIDWDEVMYNISQHRLKGV

IPLSLESIAYYVKHRGQQQKGAMVYVSEHTAISYYVAFHFPRRTALQQPFDRLLHRLQAgamGFILHWRAEY

RNNPNGATNYEQQDGVVPTPLQLQQVAgamGFYLWALGLLAATVAFVGEFAVSKVNRASKHI

>AgamIR7n

MRLSWTCTGQLLVLLLSVLRFSAAAANLANNKTLAKLTGQLQSASFTHTELLIGATAEILLQHYTTLTSK

QLYVRWEDGASGPLVDEILRRTATHLSVLVERDPPVTPADGLDQSHHPQLRLLNLLLVTDRAEFDHVVAgam

LTDELYDYSGLYTVLLVGASSKRQHLETAgamIILRTLWALYIVNVVVLIGDGRQQQADGDDDDDDVRLYTY

FPYGEDYCERALPVVWNVYERGVGFVHREHSPFPPKLDNFYHCPLAVATFPVYPFIIPSAMMADLWPGHP

ERTEQEEEEDLKGIEAMVLRTLRQRLNFRLQLMNVDPPDWGTAgamPRDQATGASAYVSSTEEPEKPCPSAS

QRTAVQTPNLTKQIRHLRANLTIGYWATTLHRNRYMANSFAYYTSQLVLAVPPGAPYTSLELLRCPLAKP

IWTFLLCSLAAgamLAVIGLLRWAgamSPTARHFVLGHNAPNRAWPAMIAVLLGAgamLSRAPRGSFARALLLCWL

AgamTLVLRSAYTGSMIRFLQSDRNHTVPANLPALLDAgamYQPYMYRNYSFVFDAYPHIGRRVQLVTAHQFRT

AIVRRLQRPGARLCVLLPLETVTFLNRNLTRTGQLLRIARERVTVAKLAIYTQRTSALLGPLNKLLERFV

ASGLLHRWAAQYHQLRFLANPYQYRGRQQLVLADmelDGPFEILLVGLGLGAgamTFLIELVVGRWEGGNAKNA

KAASNSRTKNRTAVCLSSCVTRSIMTNDASTGNQRAgamEHYYYTVHEH

>AgamIR7s

MWWRRRVQLLIAPLWFHCCCWAQPPLPTQPLVNLADIVGVVLHTHFRTPFATTLVTVRSATQRGAWLQQD

LLERLLVRHGQGQLVVQLEGVPSPVQLLHRHPPWSRTLLLAESYDALRTVFGQLTPDRFDFTGRYLIALS

EAPATLATVDRIFHELWLRQIVNVVVALRPLDDDHSSAAAAAgamPVQLWTYFPFSPGLCRIPKPHLLFTWP

NDTLLYGVDFYPRKSDQFHGCPLRVGSFETRPFTILAgamGDGANPPAVGGFEGDLLHSLSARLNFREDVRV

PPRAQQWGEAAFENSTGLMRMLYTEEVDFGLSCLGVSVERSAMLKAgamKVHFTTDLVVVVPPGKPYTAFEK

LFQPFQPPVWLAVGFCTGVGLGVLATLRLLPLDGRQRATVRRYVAgamDARLQAPALNLVRVLLSSPLPFTP

TGTFPRTLLAQWMLVSLLLSLLYQGSLFQYLQRASTHPPMRTLAEIDRAgamALYHIASSARRFFLPYPQRL

SRVRYFPPVPDSIAAWLRWMGSHPGEPHVAMCTRDHVAYHNAQHGRTDGRQLLIARESMALYAITILYPK

RSMLTASFDQHIERIGSSGLLKYWSARYGDYHFGESRASSSSTTDQRKTATAgamPRPVSVEQLAgamALQLLL

GMLLAATLLFLVELGWARHTRRWRHNC

>AgamIR7t

MGSAIIIAIMHLLVLSVPEVAAHLLNAKPSGNPATVTQQASLLTPIVQTHYRLPEGFVAVRVQNGGHSNP

SHQQQDLIDGLMRTGHDWLAVSFDDLPAAERRPAYYGVFLVADYRSLCTLLDGMTPGAYQFDGLYTIVIE

QRRPKLHDVMERLWSYRLLNVVVIVSEKRANEDEERYVAYTYDPYREHRCGSVEPYAVGQYANGTWTELV

RWYAKRTDNFNGCPLVIGTIHIIPCSIIERDGPGGSTTHKGIEVSQVDDLSRRFNFTPEYRISNGSTRWG

FARAVNSTGLMGWIQRGEVDFGLGSIGISLSRVQHLRPGIASRFGQLAMAIPPKRPDSSVEKLIKPFSRQ

TWLCVVLGLAgamISTLAWALFGIGWRLVADRLRHPCYTVWVLTMGGPCGALRMDSTRLFVVSLVLNMLVVR

TLYHAAMFERLQASASLASELDTLEQINRAgamKTYIMHKTITLFFNDNPLVGPRRIRRTLHDNENWEELLY

QLSQPGSDFVVTLPLDCIKYYVQQYGNRGLVYVGKHNGITYNTAFFYPQTTALQAPFSARVLAYHSAgamLV

DQWARAFEDGRYWSNAKADPEPASLAWSHLSGAFYLCGTMHLLAVCVFVAELGWARKWRKASEQPTPRQY

HPAH

>AgamIR7u

MKLFAALLALAMVAQHCYCSAgamKLIPLVDAVSATEHLQAPLKQHFKYPTFPVNFRAESSENGTTTTTREL

FEMNELMRENSGWLIGTVSGRLPIVTNPYASFYNVFFADGYDAMGKILQTLNYTDYDPTGRCLLVINAAY

ETDHMVQLVAILWQLRIVNVALIVQEAATDTDSYRAYSYDPYREGKCELLEPLLLDQFVAgamRWQSLHRWY

RNKMENFHGCSLSVGTFAAKPYSMVRREGNATIRYGMEVSIVENVARWFNFTIDYRSPAgamTVKWGIIRAA

NSTGMMGMIQRNEVAFGFGCMGYNEYRNRYLTVGLPSFITQLSMAAPPARPFTWLEKLFAPFTLEAWLCI

ALCYAgamYLLLTVLVFDSRLVTTVEHFRNPAYNVWVMLMGGPSRPVRQTSIRLFLAgamFVLNALVIRTMYHS

AMFERLQATTTLGSDLNTFQQINAAHMLYYMYITTSFYYKDNPLVHDRIRILWDETKDWDEVMYNISHYR

LNGVFVIPLDAIEYYVKNVGQRGLVYVSSHTSINYNPGLVYPKASPLTEPFSAIIGRYQAAgamLVPIWREQ

FRDTRYWNNAKQHPEPISLQWSHLSGGFYLWACLLALSSLVLVGERIMSRRK

>AgamIR7w

MLLFCLMLWCTFADGCTVQRREEEMPPEFLQSESTNTRFLAPILRAHYRALALEVHFRSWNGNDTQRQLT

PWQGELIDDALCRNADWMIVSFADLLAPAVQRRSAHYSVLLMLDYDALCSWLQHGLDSGAYHFDGLYTIV

IEQLADRGQLHAVMRELWNREIINVVVVVVLASGEADNRGELVAYSYDPYGEGQCGNAAPYEIGRYANNN

GTWDRLAgamWFPNRLTNLHGCSLTVGTVEVSPFSMTRTVDNRTVQYGLEVHIVDTLAARLNFTFRYVRPTD

GVKWGILYAANSTGLVGLLQRRGADFGFGSLGFSLNRHTYLRMGVPNHMTQMIMGIPPKRPYTSFEKLFQ

PFAADAWLCIALGYAAFALVALALVTVNRRLAREPALQHPLYQLWVLLMGGAVGWLRLDSTRLFLIGFVL

NALVIRTLFQAgamLFQRLQSSASLASDLNTLEAINRAgamLFYNMFRASLQFYRDNPSIPASRIKLVPNDQRD

WDDLFYELSQDRLGGVMVSPLDCIAYYVKRRGKNGVVYVGKDTGFMYNLGFHYPKSTVLQRPFDGWILRM

HAAgamLVHHWSEEYRDNRYWTNAKEDPEPASLRWNQISGGFYLCSALMLLATVVFLGEIVYFRLRTRRLLQ

RCCGRKTRTRRKKSV

>AgamIR7x

MAPLPVWWTSRLMCCWLLWSTTAARAQIPPQPDTDQQLLPHIVTHLLQRYLRHPFQPVQFFLAANTAPHL

LAQRDLLSQVLRHTSGQAAVTFGSSTNNPVGLRAAVQQQRSRAILFGEDFVAFERLVANFSTTLNDYSGR

YLCVLTGASGSVHQLETKHLPRLFDALWTRHIVHVNVLIAAANGTVRAYTYQPYTPERCGKPAVKLAAVF

APGSSVDAQPHLYPRLTTFWNCTLQVGSFEAKPYTLLRPKVDGYTELGGFEGDLLHLLASRLQFRVNVTE

SPHQVQWGVIGAPGNSTGTMQLVQDELVDLVIACMALDVTRGLYLKAgamWAHYTSRILFAVPQGRPYTAFE

KLFRPFGTAIWAALAgamTLLGVAgamVVGGLSCGRRARSWRRFVYGPAVRMPLLGALYLLWGGSVVAVPGRNF

ARSLLALWLGFTFVVRTLYQGSLYLYLQRSATFPPLATIEQVHRSTLHYHMVNIAMRFFVDRPEIKPRVR

FIPPGLDTLGEQVAgamMAARYTDRVVVCPQDmelVAYNNRASRGRQPAAAPIQVTRESITLFPLTIYYPKKSC

LTQPFDRLVRHVVESGLVSFWVRSYGDYDFEANRREPAgamGGEPRKLTLAHLVGAYQLLAAAHLLAFVIFL

LELVSLRLAALRRVLEFCMD

>AgamIR7y

MNRWTILLLAAgamLRECRPEDSNGGMKRLVVRETVGNHFSEVIVNALTRYYVQNHSSTQVMRLSTVSDGTY

DLQSDIMDEVMQRTSRSIAYEFRSALGPSRRPRIFNIAFIDGYGAFEQLFRSLDPAENDFAgamYYLIVLTA

YGRRIAPDTLARIFALLWTMNVINVNIVSADLEDQSRWQSVLMHTYYPYRAgamGNCERIVPHLLHRFRKGQ

RLDRSVELFPSKLSNLHGCPVTVGTFHLPPYMLLTPAgamRYGGLEGDLLRALSRKLNFTVRLVVPDDGELW

GRTPSPSVVVADANRTRGGASSASGCVRLVLTERVNLTLGRFAIRGDRNLVMKSSRSYYTVRMVLAVPAgam

REYTPFEKLFRPFSRSVWVLVTLYLLAgamMVVIGTVQLLHRPAVRDFVYGRGVSTPVLNLLSVLFGGAVVQ

LPMRNFARTLLFLWMYYCLVVRTCYQGSLYEYLQERKNFSPLQTVDALVRNDYRFYSLHGTSLYLEQMPQ

ILSRITWLPDDDASVDRLLTELATRQALTSALFIDLERVAYHNRFYVRGGLVYIANVVLVRLPIGIYYTK

KSCLANRFDHELDRLRTSGYVSYRLGHYLNYDAFKGPADYQPLPTPLTTDQLVGCYETLFGLLLVATGLL

LLELASRWFVSLRQLLTFLQAE

>AgamIR8a

MFEGVSVLLDTTWIDSRYLASAAEEFGIPYFHIDLSVQTYVKLLESFLLARGGNDVVYILPNYQDADATI

YLLITDSYLRAIVFGELDGDTVDRIKDLRPYPSFYAVIAgamTMEMNTILQKAIDGGLVRKPEKWNLLFTDL

QTDKYTLGDDFPQMNRLVLDSNTCCILLQQRFPCICPDPFDPMDAHLQNVLERIVQWFIKDPIPLVRASN

CTPESQQQELLEHAIAAQTKLMNEIAAMAEFWITLESGMLRPNLNLSIISQPANQNTTSAAAQPPPATTI

GNVAKGQIVLVKNESLKPSKRFFRVGTTESIPWAYRKRDPNGNILRNSTTGEPIWEGYCIDFLHQLSVVM

NFDYDLVSPRNGTFGLRDADGKWDGLVGDLVVGEIDFAIASLKMTAEREEVVDFVAPYFEQTGILIAMRK

PVRETSLFKFMTVLRLEVWLSILLAIVATAVMLWLLDKFSPYSAKNNKDAYPYECRKFTLKESFWFALTS

FTPQGGGEAPKALSGRTLVAAYWLFVVLMLATFTANLAAFLTVERMQTPVQSLEQLSRQSRIKYTVVKDS

DTHDYFRNMKNAEDVLYQMWRNLTLSSGNDQAQYRVWDYPIKEQYINILSAIESANPVATAAEGFRRVNE

RLDADFAFIHDSAEIRYEISRNCNFTEVGEVFAEQPYGIAVQQGSHLQDELSYFILELQKERYFESLTAK

FWNNSARSQCPNTDDSEGITLESLGGVFIATLVGLALAMITLLGEVIYYRRKETSRNFIKVAPFGNADKP

MARDGKRIALKQKSIAAVKHLLDLDTPAKALRGATTTVKINTKKEKPIPKEITIGNKF

>AgamIR93a

MVLRLVGLWSILLLLLLLLVLRPDPAVGDDFPSLLSTNASMGKLNITPLLSIILDREYLGADYERTLDET

KNVVEKLIREHLKNGGLIVKYYSWTSINLKRDFSAVLSVSSCKNTWDIYQEAVRERLVMLSITDPDCPRL

PTNNAIMIPRSDGSGSNAFDEVSQIILDmelKSSRAINWHTATLLYDQVYDAEISRCILSLLEDREGIKPLT

LTEFKINAPTHSWEKRKEIRRTLLGIPTAYTGRNFIAIVNIATLTLLMEISKDLKLVNPFAQWLYLIPNT

EKANSNFTTRSTLINEGDNVAFVYNSGSKAQNCTVSVLCYIESYLLHFIRSLSKLIREEQVVFGQISDEE

WEIIRPSKQERKTKFLQMIKAAITSKDECNKCSQWKIQSAETWGYVYRTDFLTDGADLQERRKYTMLDIG

YWSPQDGFMLTDALFPHTQYGFRGVQLIFYSYHNPPWQFVAYNDSGSPVISSGVVYDILNELSRKLNFTY

TMVISQPAEINGSLVEGNTSSVYDLKTISSDIPQEIFSTLVNNKILLAAVGATVNEKQKKFVSFTDPISI

QTYSFVISRPRPYYEVHNKPTDTGLGKVNNCFWYIYGALLQQGLYLPYADSGRIIIGTWWLVVLVIVTTY

CGNLVAFLTFPKIDIPVNRVMQLLRNDRGMTWSIRRGTFLEEMLMVQVISSPIIYDSTEPKYMQLYKGSQ

IIGELTDELVERIEAgamQHVHIDWRNNLRYLMKRQFLRTDRCDFALSTDEFLDEQIALVMPKDSPYLELVN

EEIKRMHQFGFIQRWVAQYLPAKDKCSGTGRVMDVQNHTVNSSDmelAgamSYWILLLGFVSGLFVFVCEFAVA

WYRKHRAARAATVAYRD

>DmelIR100a

MATTLQLIMLALVGGTLGQANNTDHKQVLTSIVKQLEGGLELHLRTSEDGGNDLVQFLMQEKSSIIISAK

QEEVPSRAKIMRHHFFIFDGVHQMQEIRTSLFNTDGFYILALENNTIEDDVLLMEFAADVWLQHGHSRIY

YVQLSKKSVLLFNPFLQRLVVVQDSKTYSRIYKDLEGYHLRIYIFDSVYSSVIGDGENKVLSVTGADAKL

AKTVARQLNFTADFVWPDDEFFGGRLANGEYSGGVGRAHRGEVDIIFAgamFFIKDYLTTHIQFSAAVYMDE

LCLYVKKAQRIPQSILPLFAVHMDVWLCFLLVGLLGALVWLILRAVNLILGIEGVPDGSRATRISYFGAA

RRIFVDTWVIWVRVNVGRFPPFHSERIFVASLCLVSVIFGALLESSLATVYIRPLYYRDVNTLRELDESG

QPIYIKHPAFKDDLFYGHNSEVYRRLDAKMMLVAEGEERLIEMVSKRGGFAgamVTRSASLQLSDIRYVMTK

KVHKIPECPKNYHIAYVLPRPSPYLEEVNRIVLRLVAgamGIVGLWTGEAKERAKWSIQRFPEYLAELDVGR

WKVLTLSDVQLAFYALTIGCLLSAIVCMAEILLGRQRRLHSPK

>DmelIR10a

MAVLGTVFLLFMLDLKTLNLTRLNGLLVEPTRDLPQLELWLRAgamSDHQDAENPYVQWFLLRTEIPLSIVT

YQENRYWMDDPFGRRNLVLVMSLDQLLTNRGAAAPIQKASTFFYILADQDKDLSADEQLRLEGSCRQLWT

QHKVYNRFFLTRDGVWIYDPFKRRDSAFGRLVRYYGSETLDKLLFRDmelAgamYPLRIQMFRSVYTRPEFDKE

TGLLTRVTGVDFLVAQMLRERLNFTMLLQQPEKKYFGERSANGSYNGAIGSIIKDGLDICLTGFFVKDYL

VQQYMDFTVAVYDDELCIYVPKASRIPQSILPIFAVGYDIWLGFVLTAFACALIWLTLRVINLKLRIVSL

GNQHIVGQALGIMVDTWVVWVRLNLSHLPASYAERMFIGTLCLVSVIFGAIFESSLATVYIHPLYYKDIN

TMQELDESGLKVVYKYSSMADDLFFSETSPLFASLNKKLSWNRDLRADVIDEVARFRNKAgamVSRYTSLIL

ESSHFTLLRKIWVVPECPKYYTISYVMPRDSPWEDAVNALLLRFLNAgamLIVKWIQDEKSWVDIKMRSNIL

EADAESELVRVLTIGDLQLAFYVVIGGNLLAFLGFLAEHFRWKLQKKGV

>DmelIR11a

MRFAILWLFSGCLLPGIQVGIWVVVRAQPTGRDVLLSRLGNQQNELNTRRLANASSYLTRNYIANRINTL

VVREICVECPYELSERQRQLVDQILASLAPELSVLLHKGTAEETTWEYTLFVVNDHTAFTGQVFIFPDEL

LEREFFCIVVVSEIQSRQFVRQTVGSIVKSNLQMHFVNVVVVAQLEDGTVGTYSYKLFKANCTPGITVRQ

INHFDRITGKPQQSMPDLYPVRNGHLGDCPFNVGAAHMPPHLIYKRHKDPPPASNVSIPAEDLAgamIDWDL

LQLLAKALKFRIQLYMPQEPSQIFGEGNVSGCFRQLADGTVSIAIGGLSGSDKRRSLFSKSTVYHQSNFV

MVVRRDRYLGRLGPLILPFRGKLWGVIIVILLLAVLSTCWLRSRLGLSHPIEDLLTVIVGNPIPDHRLPG

KGFLRYLLASWMLLTLVLRCAYQARLFDVLRLSRHRPLPKDLSGLIKDNYTMVANGYHDFYPLELTCRQP

LDFSARFERVQRAAPDERLTTIALISNLAYWNHKHPNISRLTFVRQPIYMYHLVIYFPRRFFLRPAIDRK

IKQLLSAgamVMAHIERRYMQYENKRKVASNDPVLLRRITKSIMNGAYRIHGLVIVLATGMFILELLAgamRSN

GRLRRWMEWVHQ

>DmelIR20a

MLASLNRSTGLSAELLDLYGLVVHFLLSGEHTTLVYFNPAgamLDCSWGVLWQRNLTAHPQIVWQRNYSYPD

LYYQFNAKLLVLACLPMDSRAAIQLEILANSLSHLRTVVRLLIEVAgamPDQVTLARQYLSFCLRRSMLHVE

LYFRDYHHSLILYSFRAFPSFELVMRWISVGQGVKLFLHKLDDLRGHRLRVIPDLSPPNTFFYRDARGDN

QVTGYLWDFLATFAgamRLNAgamLEVVRPSWRAgamSASDSSYMLEYSAKGLIDVGLTTTLITKWNLWAIHQYTY

PLLVSSWCTMLPVEKPLATPDLFGRIVCPTLAMTLLLIILVTWLVFRQLRCLTRLKNSRPARIVPHLLTL

LLLTTCSAQLLSLLIFPPYHVRIASFEDLLRGDQKILGMRNEFYNFDGAFRARYAgamVFYLIDDPNELYDL

RNHFNTTWAYTMPYIKWLVIKTQQRHFSKPLFRWSKDLCFFDFMPTSVIVAPDSIYWESIKDFTFRIHQA

GLMKHWIRKSFYDmelIKAgamKMSIKDYSDLETLKPLNIGDLEIVWRVCGAAIAVASAIFIMELLYFYINVFF

NSL

>DmelIR21a

MSYYWVALVLFTAQAFSIEGDRSASYQEKCISRRLINHYQLNKEIFGVGMCDGNNENEFRQKRRIVPTFQ

GNPRPRGELLASKFHVNSYNFEQTNSLVGLVNKIAQEYLNKCPPVIYYDSFVEKSDGLILENLFKTIPIT

FYHGEINADYEAKNKRFTSHIDCNCKSYILFLSDPLMTRKILGPQTESRVVLVSRSTQWRLRDFLSSELS

SNIVNLLVIGESLMADPMRERPYVLYTHKLYADGLGSNTPVVLTSWIKGALSRPHINLFPSKFQFGFAgamH

RFQISAANQPPFIFRIRTLDSSGMGQLRWDGVEFRLLTMISKRLNFSIDITETPTRSNTRGVVDTIQEQI

IERTVDIGMSGIYITQERLMDSAMSVGHSPDCAAFITLASKALPKYRAIMGPFQWPVWVALICVYLGGIF

PIVFTDRLTLSHLMGNWGEVENMFWYVFGMFTNAFSFTGKYSWSNTRKNSTRLLIGAYWLFTIIITSCYT

GSIIAFVTLPAFPDTVDSVLDLLGLFFRVGTLNNGGWETWFQNSTHIPTSRLYKKMEFVGSVDEGIGNVT

QSFFWNYAFLGSKAQLEYLVQSNFSDENISRRSALHLSEECFALFQIGFLFPRESVYKIKIDSMILLAQQ

SGLIAKINNEVSWVMQRSSSGRLLQASSSNSLREIIQEERQLTTADTEGMFLLMALGYFLGATALVSEIV

GGITNKCRQIIKRSRKSAASSWSSASSGSMLRTNAEQLSHDKRKANRREAAEVAQKMSFGMRELNLTRAT

LREIYGSYGAPETDHGQLDIVHTEFPNSSAKLNNIEDEESREALESLQRLDEFMDQMDNDGNPSSHTFRI

DN

>DmelIR25a

MGSRLDWGVADVALWAIADQIDYHQVFINEVDNEPAAKAVEVVLTYLKKNIRYGLSVQLDSIEANKSDAK

VLLEAICNKYATSIEKKQTPHLILDTTKSGIASETVKSFTQALGLPTISASYGQQGDLRQWRDLDEAKQK

YLLQVMPPADIIPEAIRSIVIHMNITNAAILYDDSFVMDHKYKSLLQNIQTRHVITAIAKDGKREREEQI

EKLRNLDINNFFILGTLQSIRMVLESVKPAYFERNFAWHAITQNEGEISSQRDNATIMFMKPMAYTQYRD

RLGLLRTTYNLNEEPQLSSAFYFDLALRSFLTIKEMLQSGAWPKDmelEYLNCDDFQGGNTPQRNLDLRDYF

TKITEPTSYGTFDLVTQSTQPFNGHSFMKFEMDINVLQIRGGSSVNSKSIGKWISGLNSELIVKDEEQMK

NLTADTVYRIFTVVQAPFIMRDETAPKGYKGYCIDLINEIAAIVHFDYTIQEVEDGKFGNMDENGQWNGI

VKKLMDKQADIGLGSMSVMAEREIVIDFTVPYYDLVGITIMMQRPSSPSSLFKFLTVLETNVWLCILAAY

FFTSFLMWIFDRWSPYSYQNNREKYKDDEEKREFNLKECLWFCMTSLTPQGGGEAPKNLSGRLVAATWWL

FGFIIIASYTANLAAFLTVSRLDTPVESLDDLAKQYKILYAPLNGSSAMTYFERMSNIEQMFYEIWKDLS

LNDSLTAVERSKLAVWDYPVSDKYTKMWQAMQEAKLPATLDEAVARVRNSTAATGFAFLGDATDIRYLQL

TNCDLQVVGEEFSRKPYAIAVQQGSHLKDQFNNAILTLLNKRQLEKLKEKWWKNDEALAKCDKPEDQSDG

ISIQNIGGVFIVIFVGIGMACITLVFEYWWYRYRKNPRIIDVAEANAERSNAADHPGKLVDGVILGHSGE

KFEKSKAALRPRFNQYPATFKPRF

>DmelIR31a

MNLLISMFILILAAgamEGEIIPSMEESVVTNFVKSLVKTKQAIVFSCLFKDFKEISLALMRINQFVSVVNL

NQSYSLTSILTRENYARTSVMVNARCSGSSELLFEASENRYFNKTYQWFLWGVDLEVQSLFPLNLNYVGP

NAQITYVNETADGYAYWDIHSKGRHLKSNLEINLIATLINDTLNIARDIFHLQSIDFRGQFNGLTLRGAS

VIDKEDIISNEQIESILSRPTKDAgamVAAFIKYHYELLGLLRERFNFTVNFRNSRGWAgamRLGNTTFRLGLL

GIVMRNEADIAASGAFNRINRFAEFDTIHQSWKFETAFLYRYTSDLDTHGKSGNFLSPFSDRVWLFCLLT

LGAFSIIWVLFEIIDYKILRIRVNSQKLEHLNQKSSVICIKTTCIERILQTFGACCQQGLDPNPVDRSVR

FLVMTLFLFSLVMYNYYTSSVVGGLLSSSDQGPSTVDEITASPLKISFEDIGYYKVLFRESQNRSITRLI

EKKLSSSRSLNELPIFSHIEDAVPYLKAgamGFAFHCEVVDAYPVISEYFDANEICDLREVSGLMEVEILNW

ILHKNSQYTEIFKTAMCNAQEKGFVERILRRRQIKKPACQSLYTVYPVSLSGVLPGFVILICKSINKFS

>DmelIR40a

MHKFLALGLLPYLLGLLNSTRLTFIGNDESDTAIALTQIVRGLQQSSLAILALPSLALSDGVCQKERNVY

LDDFLQRLHRSNYKSVVFSQTELFFQHIEENLQGANECISLILDEPNQLLNSLHDRHLGHRLSLFIFYWG

ARWPPSSRVIRFREPLRVVVVTRPRKKAFRIYYNQARPCSDSQLQLVNWYDGDNLGLQRIPLLPTALSVY

ANFKGRTFRVPVFHSPPWFWVTYCNNSFEEDEEFNSLDSIEKRKVRVTGGRDHRLLMLLSKHMNFRFKYI

EAPGRTQGSMRSEDGKDSNDSFTGGIGLLQSGQADFFLGDVGLSWERRKAIEFSFFTLADSGAFATHAPR

RLNEALAIMRPFKQDIWPHLILTIIFSGPIFYGIIALPYIWRRRWANSDVEHLGELYIHMTYLKEITPRL

LKLKPRTVLSAHQMPHQLFQKCIWFTLRLFLKQSCNELHNGYRAKFLTIVYWIAATYVLADVYSAQLTSQ

FARPAREPPINTLQRLQAAMIHDGYRLYVEKESSSLEMLENGTELFRQLYALMRQQVINDPQGFFIDSVE

AgamIKLIAEGGEDKAVLGGRETLFFNVQQYGSNNFQLSQKLYTRYSAVAVQIGCPFLGSLNNVLMQLFESG

ILDKMTAAEYAKQYQEVEATRIYKGSVQAKNSEAYSRTESYDSTVISPLNLRMLQGAFIALGVGSLAAgamV

ILLLEIVFIKLDQARLWMLCSRLQWIRYDRKV

>DmelIR41a

MFIDLSWSLVLSAIVGKYLNESTICIFWNDKFEFQLLHKSDYISFVGINIKSFDDNGGHYIIDTGLKKKE

LQNKHLFLDELVIKIIISIEVTHCETFVVFDKDIDRFVNAFNKASVYSIWRSLHNKFVFAHIANESPESR

NHFFEDQPNILFVVRDHSSASSFDIKTNKFVGRKAENPSQMILVDRYLASEQRFQFGKSLFADKLNNLQG

REVIIAgamFDYPPYTVIKHNMSTNAQDmelGVSGESDFKNVYIDGTETRIVLNFCEQFNCTIQIDSSAANDWG

KVYPNMSGDGALGMLINRKADICIGAMYSWYEDYTYLDLSMYLVRSGITCLVPAPLRLTSWYLPLEPFKE

TLWAAILLCLCAEATGLVLAYKSEQALYVLPGYREGWWTCTSFGVCTTFKLFISQSGNSKAYSLTVRVLL

FACFLNDLIITSIYGGGLASILTIPSMDEAADTVTRLRFHRLQWAANSEAWVSAIRASDEALVKDILYNF

HIYSDDELLRLAQDQHMRIGFTVERLPFGHFAIGNYLGPQAIDQLVIMKDDIYFQYTVAFVPRLWPLLDK

LNTLIYSWHSSGFDKYWEYRVVADNLNLKIQQQVQETMTGTKDIGPVPLGMSNFAgamFIIVWILGSAIATL

TFLLELSLTYILKQSNLK

>DmelIR47a

MRQIKLLVWLLVVGVVSSTEQLQFLKNFLEAVHKERSISTILLIQRKVHKNDFLHGLYPIFWPIICLDET

KRVELVNNFNKDFLALVYMESEADTLLLSALAADLNHIRDARIMIWLQMSPSENFLDRIVFQASKQKFLN

LVVIENTLKTRRFYPFPQPKVQVIDKPFEEKEIYPALWRNFMGKNAIAVPDLVPPRSFNSFDPKTGHRRE

SGSIYNVFKAFTQRYNITMLLKWPLIRNTTQEEIIGKSVRGEIDLPITGQLISFRHPNGSRSQPLLGMTA

LSIAVPCGPELPMFDRFFLFYGLATPITITGYYVLLNTIEIILGTLSDRIKRHPRRKKILNLVLNLRVFS

CILSLPTPQGNRLRSVKGQLTMVMSITGLILSCIVAAQTSTILTMKPQYRHIKNFQELSDSNITVVCNHL

NYLTIKQQMDPKFMAKFMQNIWIVNSIEQMKMIFDLNTSYAYQTFSYKKDPFTLLQMHTTRKAFCRTPGL

DLVSGLAYTAVLEKNSIYALALQDYTLKAFSAgamLVYYWAEESIRDLISTVGRTQFEKLPIVIGYQSLKLQ

DYNVCWKILLIGGALAFCVFIVEVVVGLINRRI

>DmelIR48b

MILQQSSNLLKLLLLLAISSVRTQGLNDIIIELNQRLLISNNFLYCNQSDKLNEYEIKYLQHMPPISLMI

FTSIESMNFTQVEYNLGADNKLFLIMGNEEPPYDFLHALNLHFQFAEYIIVIDEPVDLKKSTKWLDFVNH

LWQQGYVQLLIYTSYDEKLYHKIIFPETVIEETLVEQYISIRGSFNNLYGYPVRVAAYNNAPRSMLYVNR

WGKHIFAgamFYMRFLRAFIDARNGSFVPVLTPSNSPGNCTLNLVNETVDVCADALAANPAAFSLTHGFRIA

SANVLVTHAKPLHSYRYLTAPFQWSVWACLVIYVLLVVNFLSFIGWLRSGKWEFSKYLLEVFSSLLFSGF

YLKEIRGRERYILFGVLFIAgamFVYSTEYLGLLKSMLISEVFEKQIDTFEALVESNITLMVDPYDKILFAK

YNMPEILSPIMELVSFETLLKHRNRFDQDYAYILFSDRMALYDYAQQFLKHPKLLRIPIDFSFLYTGIPM

RKRWFLKHHLGRAWYWAFESGLTRKLALDADFEAVRVGYLSFLITEHVEAQPLNVDYFVMPAIALAIGYI

LALLSFVIEMTAWRIREFLGCRKATMTSTGCSEGGHVDVD

>DmelIR48c

MSLLRIILIIIFLRIVSSIPDTIISHLSAELQIKIQIYFGLGNDLYDFSRLDGNYQKIIISHNISEEFKT

YHDEPVLIIIRLERDLNLNLATLDVLRSYLTDRQYNDILLIDNDEENLNSYVDIRKAYWNAgamFSQVLIYN

SQQRTWSIKPYPYLQIRPTSLKEYIENRNTRNLMGYPLRVLVTNDPPHCFVDKDELPGSPNRYKGSIVTM

LKIFADQLNATFQANPFREFRRYSTADCVQMVSDDEIDACGSIFIRTYTYATSQPVRLNRVVIMAPFGNP

IEKFYYFFRPFDLYVWIGTGIIVVYIAVMGSLLHRWHFKEWNVGQYLLLAVQTLLNRELSLPQSSSGSKF

MLLLLLFAIGFILSNLYVALLSMMLTTKLYQRPIENLADLKAANVNILLQTHNIRPNSVYGSSEELRERF

LLVEESQHLEKRNGLDPSYAYVDSEDRMDFYLYQQKFLRRRRMKKLSNPVGYTWAVQVIKQNWVLEKHYN

DHVQRFFETGLQNKLVDDVHELAVKAgamFLHFFPTQTQTIEPLRLEDIVMAAMVLGGGHALAVICFLVELF

A

>DmelIR51b

MCKVLTLLVVILLLALTNAAYNVTLLKSVLSLISTREPWINTPIFVGHNTQGGDLNDLIIWLHQTMGVTS

LTMNLFLQPEHIRPLGHFKITRYNGIALFFCHDKHDIMWLTLDRNLRKLRRIRLIIILRNQRSGSQGAIK

SIFNALWQYQFLNVLVLQRDQLYSYTPYPAMRFFKLDIHTEPLFPHAARNFHGYVVSTPAENDIPRVFHV

HDPLTKSRKVLGYAYRTFVEYLDHYNASLRLTNPDENLDPTTSVNMNHIVQLIIDGQLEISLHPYVFTPP

TATKSYPLLIYPNCLIVPMRNEIPRHMYLLRPFQLYSWYILLFAVFYITGILYCISPKLNKSSWPQRLGL

NFLDAISKILFISPPITIYRPTWRHLIIFLQLSVLGFMSTSWYNIELDSFFTTIVVGEQVNSMDQLVHQQ

QRVLVKEYEINTFLRHVEPRLVEKVSRLLVPVNASEQVSALLSFNRSFAYPFTEERWQFFAMQQQYAFKP

IFRFSSACLGSPHIGYPMRVDSHLETSLNHFILKIQDTGLLNHWVVSDFNDAMRAgamYVRFVDNVLGYQSI

DVDTLRLGWCVLGIGWILSALVFSCEYWHLYPWRFIA

>DmelIR52a

MALGWSVIILGFIGQLSAQILNYTQSRDLELLEGSLFRVLSRLNLEEEYNTLLIYGKECVFHSLLRKLEI

SAVTVPSGSTDYDWSFSTAILILSCGYDAENEENSYTLMKLQRTRRLIYLEDNSEPESVCMRYSLKEQHN

IAMVKSDFDQSDTFYSCRLFQTPNYVEGHFFKDQPIYIENFQNMRGATIRTVADSLVPRTILYRDEKSGE

TKMMGYLGHMINTYAQKLNAKLHFIDTSKLGAKKPSVLDIMNWVNEDIVDIGTALASSLQFKNMDSVWYP

YLLTGYCLMVPVPAKMPYNLVYSMIVDPLVLSIIFVMLCLFSVLIIYTQHLSWKNLTLANILLNDKSLRG

LLGQSFPFPPNPSKHLKLIIFVLCFASVMITTMYEAYLQSYFTQPPSEPYIRSFRDIGNSSLKMAISRLE

VNVLTSLNNSHFREISEDHLLIFDDLSEYLVLRDSFNTSFIFPVSVDRWNGYEEQQKLFAEPAFYLATNL

CFNQFMLFSPPLRRYLPHRHLFEDHMMRQHEFGLVTFWKSQSFIEMVRLGLASMEDLSRKRNEEVSLLLD

DISWILKLYLGAMFISSFCFILEILRCGERCKRLWRCRW

>DmelIR52b

MTWLVILLCFLGYMAAHIADISVQNQSLMDNELINLLLKLRNEEFYDTLLVYGKDCEFHSVIKNVDVAVV

LVSDSMNFEWNFSSLTLILSCGPDIDNGGPNSTSIKLQRNRRLVLLKEDFQPSNICNIYTQKEQYNIALV

RENFTKSKSIYTCRYFQDPNVDEVNLSGTKPIFIEQFQNMKGKAIRIVPDLLPPRVMLYQDANDGELKMI

GYVANLITNFAQKVNATLQLDFLKPSTSITEISRMAKDDELDmelGITLEASLNTSNLETSSYPYLLTSYCL

MVQVPAKFPYNLVYALIVDPLVLGIIFVLFLLLSVLLIYSQKMSWQDLSVANILLNDKSLRGLLGQSFPF

PLNASKKLRLIFTILCFASIMLTTMYEAYLQSFFTNPPSEPEICSFQDVGSYNRRIAMSALEVNGLIKTN

NSHFREIRMDDLEIFDNMPECYELRDAFNLSYNYVVTGDRWRSYAEQQTLFKEPVFYFARDLCFSRLIFL

SVPLRRHLPYRHLFDEHMMQQHEFGFVNYWMSHSFFDmelVRLGLTSLKDLSRPLAYTPSLLMDDISWIMKI

YLAAIVLCVFCFLLEIGVDKWKRWMKFRNLQILNTC

>DmelIR52c

MVWLIIILFCLGNSSSQILDVTNNSHLDFDYRLFGLLQRLQVEKSYDTLLVYGEDCAIPSLFERLQVPAV

LVSSGSTNFDWNFSSLTLILSCNFQDEREENYRTLMKLQTSRRLILLKGHIKPESVCDFYSKKEQHNVAM

VKENFYQLEVVYSCRLFQDQNYEKLNLFDGKSIYKDQFRNMHGAPIRTLSDKEPPRTIPYIDSKTGEEKF

KGYVGMLISQFVKKVNATMQIREDLIKDDEEVSFVDITNFTSNDILDIGICEARTLEMSNYDAISYPYLM

SSYCFMAPLPDSLPFSDVYMAIVAPSILIMFLIIFCICSVLIIYIQERSYRSLTIRSVLMNDICLRGFLA

QPFPFPRQYNRKLKLIFMLVCFSSLISTTMYTAYLQAFLWGPPIEPRLTSFDDVKKSRYTMAINIYEREF

LEALNVSLEDVEIYDYGKFSKLRSTFNTNYLFPVTALQWFTINEEQKLFKYKIFYYCDAFCLNQFDILSI

PLRRHLPYRDIFEEHMLLQKEFGLTKYWIDQSYRDmelIRANLTTFKDFSPLLENDYIEVHNLYWVFTMYFV

GMGMGLCFFILEILRPLRYWRNCKIKCEYCYAFLKNFAK

>DmelIR52d

MVRIIIILLCLGYTKARILDATNTNHTDLEERLLSLLLRLQQEQFFNTLLIYGEDCAFSSLSRRLQVPTI

LVSSGSTSFEWNYSSLALILTCEFKAEREENYQTLKKLQMNRRLILLNGNIKPDSVCDFYSKKDQYNIAM

VNNNFHQVGIIYACRLFQERNYEKVYLSEGNPIYVDQFRNMQGALLKSITFNLIPGSMAYRDPKTGQEKH

IGYVANLLNNFVEKVNATLDmelQVKLHKAgamKKTSFYNITKWASEDLVDIGMSYAAYFEMTNFDTISYPYLM

TSTCFMVPLPDmelMPNSEIYMGIVDPPVLVVLIAIFCIFSVMLNYIKQRSWRSLSLVNVLLNDICLRGFLA

QPFPFPRQSNRKLKLISMLVCFFSVITTTMYTSYLQSFMWGPPIDPKMCSFADLENSRYKLAIRRYDIEM

LRPFNVSMDHVVVFDESSQLEYLRDSFDDNYMYPMSALSWSAFKEQQKLFAFPLFYYSEKLCLKPISFFS

FPIRRHLPYRDLFEEHMLQQNEFGLSTYWIDRSFSDmelVRLKLATMNDFSPPRLEDYIEVSDLSWVFGMYF

TGLGISCCCFGLELLGLPSWTRRLRLTNWLRVRN

>DmelIR54a

MWTVITGIVLWAPVLVAgamSAVDFIFRAAAEHSLSVIMIRIDYCPYNWAKDIFENQTIPVVVLSDSETFIN

IRMFSRPLHVACLPGHELQKDLALLENFTSSLMDFPSQKKIVYISNNFSDPTRMDYIFETCYHRRIWNIV

GLLASDEHRYFYRYHLYPSFRTEYRSLESSTIFDKDFPNMHGHPLTVMPDQWLPRSVLYVDRRTGKQILA

GSVGRFFHVLSWKLNATLQLSKKVTTGRFLNATALKELSESFSVDVPASLTIMERVEQLASTSYPMEVTH

VCLMVPVARRIPIKDIYFILSSASNMFLAIVIVSSYGLALNLLRNMTHRDVRLVDFVLNDKALRGILGQS

FNLPLSRSFSTRLIFLMLGIVGLNVSSIFGAgamLDTLMAHPPRQFQARSFAgamLRRTKIPLVTTEEDFPTWM

KLRVPMLVVNVSEYNHLRNGRNTSNAYFASRLYWNLFSEQQKRFTRELFIYSTDDCLWSLALLSFQWPQN

SLFTEPVSQLILEVNANGLYDFWVGMHYYDmelTAAgamLSGLEDPSLQLKEREHPTSLRIVDFQWMWQAYGTF

MVIAILVFLLEVSWHRITSLFVSLVY

>DmelIR56a

MGSRFFIRNLILFGLLASSNMQIPFGELEKKFELDVDFLLGVTELVGHIQGLYSITVYADCIDIHPSIQQ

RIMDKFMVPVNTIGSNLSRPNYHKLDNSRIRIVLFTGLNDTILVNLNKTDVPYSDNFYMLAYASAIKNKC

IELDFIEEVFTLLWKMSIQNAILLIRGEFMMEMWSYLYMGKIHKIKLTKPNSYLESLRKYNYRFSLEVIN

DPPAIFWYNSSEQADVTGGGNLSVSGPLGLIIINFLRHLNVTIDIVPIPGKQTSQYELFQQPDNLRAENG

VNMVGSALLKYSPMVTQSRMCLLVSNRRMIPFSRFLDRLVSPGVHKLTFVSSIGIFVIKYFSHRPRSFVD

AIFCTIRFFFAIPLPSIILNRLPVVDRFIEVFIIIFVQILLSSNISITTSALTTGFWEPPIINVETMRAS

GLHILTEDPTILQAFKENILPSSLADLVILVDEDTYFHHVTTLNNSYVYVVQAHNWQIFRLYQQQMTNEP

FEIASEELCSKWRILGIPLNPKSPLRFMFKDYFYRILESGLREQWVHSGFKKFCEFNNLKKLPVDSVDSW

QPLSIEFYSNVIRAYIIGLVIATLAFVAELLHNGYRRKNVKKT

>DmelIR56b

MLLDTDLASGVIRSPYSFDIPHAFIFNETQFVVPKFCGPYMEIVKHFAEVYHYQLFLDSLESLPKKSVVE

QDIISGKYNLSLHGVIIRPEETSDFFNATQHSYPLELMTNCVMVPLAPELPKWMYMVWPLGKYIWTCLFL

GTFYVALLLRYVHWREPGNATRSYTRNVLHAMALLMFSANMNMSVKLKHASIRVIIFYTLLYIFGFILTN

YHLSHMTAFDmelKPVFLRPIDTWSDLIHSRLRIVIHDSLLEELRWLPVYQALLASPSRSYAYVVTQDAWLF

FNRQQKVLIQPYFHLSKVCFGGLFNALPMASNASFADSLNKFILNVWQAgamLWNYWEELAFRYAEQAgamYAK

VFLDTYPVEPLNLEFFTTAWIVLSAgamIPISSLAFCLELFIHRRKQRRPQYERFECYDY

>DmelIR56c

MQHLLNLLAPFGRMNVFQEIVWFVSPHQRLDQLDEFIMRIDEAFGKSATQTVVNNNTEMRMIYSSARRNH

MSFVFTTGAEDPIMKVFSKVLLGRHFYVSMVIYVDKVGDmelHPIYDLLTFAYNQQFFNSMVHFESMEGVNQ

LFGVSKFPVMSFENRTDFLKYMGKIWKQVQNARSDVGGFGFTTPLRQDLPHLFQSQGHYDGSTYRIIETF

VRFINGSFKELIMPPDSLGGQVINMKDALQLIRERKMEFCAHAYALFMSDEELEKSYPLLVVQWCLMVPL

YNSVSTYFYPLQPFDWNVWFFALGALLALVLLELMWLRMFGGWSGYRGAVLNSFCYIINVPIEGQLQQPC

LLRFLLLATVFFHGFFLSAYYTSNLGSILTVNLFHAQINTMNDIVSAQLPVMIIDYEMEFLLNLNKELPQ

EFLELLRPVDSAVFSEHQTSFNSSFAYFVTEDHWEFLDEQQKHLKQRLFKLSSICFGSYHLAFPLQMDSS

LWRDIEYFTFRIHSSGLLNFYARSSFGSALHAgamLVQRMPDTQEYTSAgamLQHLAIAFILLLVMSFLAgamIVF

VLETLSR

>DmelIR56d

MDNRAAELILRERNIFPTNGSDNITLLNNMFVLEMFYRITQLYHFKNFIFYISERLDLNNKDSQEFFHNF

WTYFPMAPNLIITREHHLGIPMMQFISTPSLVMVFTTGKDDPIMELASHNQQGIHWLKTIFVLFPSLQSR

DFETNPESLAQFTAEIKDVYDWVWRKQFINTFLITIKDNVFILDPYPTPSIVNKTGVWQAEEFFHKYAKN

MKGYLVRTPILYDmelPRVFKSDRPTNRYEKNFIHGTSGNLFLGFLEFVNATLMDTSANVTADYLNMTNLLD

LVSQGVYETLIHSFTEITTKFVVSYSYPIGINDCCIMVPYRNQSPADQYMHEALQENVWVLISLFTLYIT

VAIYLCSPLRPRDLSAAFLQSICTLTYSVPTFIIRTPTLRMRYLYILLAIWGIVTSNLYISRMTSYFTTA

PPVRQINTVQDVVEANLRIKMLAIEYERMAKSPLQYPESYLNQVDLVDKHMLDLHRDPFNTSFGYTVSSD

RWRFLNLQQLHLRKPIFRLTEICEGPFYHVFPLHKDSHMRSVMTEYIMIAQQAgamLMNHWERETFWEAVHL

HRIHVHLFDDEPMALSLDFFSSLLRTWTLGLILAgamLAFAAEMKWHEHVTFKRRPVIRITRKPRSFLRRFM

KL

>DmelIR60a

MWCNNPGLIIIIFLGQILNLCQGIVNLSNETANTVIFMLPEKDLGPDVWKAgamVGCLDSFAQIFFFRNPKE

RFTRAYNLMLVHAFHLSSPADQIQEGFSKLINEAVTNPGPPDREELFQMRVASDYNITNGTEDKGELILA

DNYVIVVDSVDRLKELMKKKIVEMRSWNPGARFLVLFHNATCRNRPLGVASNIFKDLMEMFYVHRVALLY

ANSTMNYNLLVNDYYSNVNCRILNVQSVGQCHDGKLYPNNAVVKASMQDYVSGFSPRNCTFFACSSISAP

FVEADCILGLEMRILGFMKNRLKFDVNQTCSLESRGEMDGPANWTGLLGKVQNNECDFVFGGYYPDNEVA

DHFWGSDTYLQDAHTWYIKMADRRPAWQALVGIFEAYTWIGFILILIISWLFWFTLVMILPEPKYYQQLS

LTAINALAVTISIAVQERPICETTRLFFMALTLYGLNVVATYTSKMIATFQDPGYLHQLDELTEVVAAgamI

PFGGHEESRDWFENDDDmelWIFNGYNISPEFIPQSKNLEAVKWGQRCILSNRMYTMQSPLADVIYAFPNNV

FSSPVQMIMKAgamFPFLFEMNSIIRLMRDVGIFQKIDADFRYNNTYLNRINKMRPQFPETAIVLTTEHLKG

PFFILVVGSCWAALTFIGELIIHRWRTQLVSTSEQQDRRSDKRRRRRRRRKPEKDNRWQRQVQVAPVVRF

TPVKRRKVFQGQTSQK

>DmelIR60b

MRRSLYLIIAIGLVDVHCVSLRYILNALENELQYRAILLVESASEIESCWEQKYIQGAVPILNFNANQSL

YLKDALNTNILALVCLNENVESTMQALYENLEDmelRDTPTILFVLSDSKVQDVFLECLRRKMLNVLAFKGL

DRGFVYSFRAFPTFRVIERNVMDILQYFEQQLEDLGGHTLTTLPDNIIPRTVVYKSPDGSRQLAgamYLYPF

LRNYVSTINATLKVCWHLVPEDGMIQLGEVVRLSEIHDVDFPLGMHGIEHGSTSQNVPLEVSSWFLMLPM

EPSLSRAQFFIMLGFEKVTPVLLLLTILLSTAHRIEMGLRPSWRCYVLGDRVLQGTLGQAFFLPRRLSVK

LMLVYSLILLNGFTFSNYSITSLETWLVHPPSGHPIHSWEQMRTLNLKVLIVPSELDSMTKALGKQFTES

NSDLFELSKSGNFQDKRLAMDQSYAYPVTCTLWPLLEHAQIRLPKPEFRRSREMVLIPLLIMAMPLPKNS

MFHKSLNRYRALTHQSGLYEFWFKRSFNELVALRKIHYKVNGDHQIYRDFEWQDFSYVWLGFVGGTIASI

LVLLAEIGYHRWQLNQN

>DmelIR60d

MRLAIYVAFLSSIGNRSGFLSSLLMSLGKELHYKTILLVGGSSTCWSLEPFETGVPILNLRGENNAYPQD

TFNSQMLALACLQTESEDAVKLLYRSLKDmelRDTPTLLFASSEEHIHDTLFLGCFRENMLNVLALTASSKE

FIYSYQAFPTFRVIKRKLVEIHRYFEPQLKDLGGHIVSALPGNIMPRTMCYRNAEGERQLAgamYLNTFIRN

YVESINGTLRISWGLVPEDDmelRHLTISRLSKIQHVDFPLGIIPLYNKTDKQHVYMEISSWFLMLPMETSV

PRAHLFVKLGLERLLPIIVVVGAVLGNAHRIEVGLGPSWRCYYLADKVLRGALAQPIVLPRRLSPKLMLI

YSLLLLSGFFLSNYYMASLTTWLVHPPASDRILEWDQLRYLHLKVLTIPEEFKYMSLILGTDFMTAYGSI

FQLTNSTDFQRRRISMDPSYAYPVTTSLWPFLELSQVRLRRPLFRRSYDmelVLQPFQVMSLPLPRNSIFHK

SLLRYAALTRETGLYYYWFRRSYYELVALGKISYKEEEGNPYCDLKWNDFRIVWLAFLGGTIISCLALLL

EVAHYRWHLGNSSL

>DmelIR60e

MVIKMISFLLVSVLLCLVGASDSESMQVQVLQDLNLALQTELNVFIDFECCATSEILHKLDSPRILLSSN

SREARDLRIRGNFTESTLIIVSVMDSDLNPLVASLLPRLLDELHELHIVFLSNEEPGFPKQDLYTYCFKE

GFVNVILMSGKGLYSYLPYPSIQPISLSNVSEYFDRARIIRNFQGFPVRILRSTLAPRDFEYSNEQGGLV

RAgamYLFTAVKELTYRYNATIESVPIPDLPEYDVYLAVAEMLHTKKIDIVCYFKDFSLEVAYTAPLSIIRE

YFMAPHARPISSYLYYSKPFGWTLWAVVISTVLYGTVMLHLAARGARVEIGKCLLYSLSHILYNCHQKIR

VAgamWRDVAIHGILTIGGFILTNVYLATLSSILTSGLYDEEYNTLEDLARAPYPSLHDEYYRSQMKAKTFL

PERLRRNSLSLNATLLKAYRDGLNQSYIYILYEDRLELILMQQYLLKTPRFNMIRQAVGFTLESYCVSNS

LPYLAMTSEFMRRLQEHGISIKMKADTFRELIHQGIYTLMRDDEPPAKAFDLDYYFFAFVLWTVGLISSL

LVFFAELVSGHL

>DmelIR62a

MYLQFLFALFLSRYQIVATENFDRAFELALFLDRIGRVHRLHAITIVNSLGSVDPSYLDDLHRGLMCNSS

NHFYMLPQMTATDKDSSHVHFSSLQDEETIYLVFARDSKDAVIYLQAERARGRRYTRTMFLLRKQESQKD

IKYFFELLWKLQFRSALVVVAARNFYQMDPYPTVRVIRMRRLSSYDPHHVFPPANRKNFRGYRMRLPVQQ

DVPNTFWYKNRRTKAWELAgamLGGILINQLMMHLNVTMDLFRFEVNGSSLLNMAALTDLIVKGKVELSPHL

YDTLQSNTSVDYSYPTQVAPRCFMIPLDNEISRSLYVFLPFSLTMWLCLLFVLLVVHFVYVRRLIPDGHF

WAILGVPGAgamQVRYGNRKPVRRFSTFLILFGIFILGQTYSTKLTSSLTVTLIRRPDNSLEELFLLPYRIL

VLPTDVYAIVDSLGHAEQFSTKFSCTDAENFSQKRISMHPEYIYPISTIRWRFFDmelQQRFLRKKRFYFSK

ICHGSFPYQYQLRVDSHLKDALHRFLLHVQQAgamLHDLWLDTCYRKAHRMGYLKDFSTLAELEEKLRLRPL

ALNLLVPAFSLFLCGMLGSGIAFLVEIRHSFGCRQKPPSINRNPGD

>DmelIR64a

MHWWLLVFLPLSCQGLPEHELLELELDYGLAEPQRTSLLQSSLILQFSQDYKHIPRITYFTCQKPHLQTP

NQIPNAAEHRDAFAAKNFQLIKSLYESELFVRIVLLDVLAQSPTSGRPNRPGNGPTGGFSQTPSQAQSNS

EWLEGVLRMEALRQIAVVDLACGAVSRRFLELASAKMLYSEKFHWLLIEDFAWHGRTQTAEGSGKRDDGE

MEEEEPPGQQIQATDDEDLPSIESFLGGMNLYMNTELTLAKRMSEAAHYTLFDVWNPGLNYGGHVNLTEI

GSFTPTEGIQLHTWFRTTSTVRRRMDmelQHARVRCMVVVTNKNMTGTLMYYLTHTMSGHIDTMNRFNFNLL

MAVRDmelFNWTFVLSRTTSWGYVKNGRFDGMIGALIRNETDIGGAPIFYWLERHKWIDVAgamRSWSSRPCFI

FRHPRSTQKDRIVFLQPFTNDVWILIVGCGVLTVFILWFLTTIEWKLVPHDGSALIKPKGGAPPRHHYQQ

QQQQEQVEAPVRPITAVSVVVSKEKVEEKQEEYEDSTPIDAgamTLWQRCYQKLNKYIKDRKAKQKKAPERV

GLFLESVLFFVGIICQQGLGFSTSFVSGRCIVITSLLFSFCIYQFYSASIVGTLLMEKPKTIKTLSDLVH

SSLKVGMEDILYNRDYFLHTKDPVSMELYAKKITSVPTTKENEADEDEPVDPNPVSTDPAKSYRDIVHSH

ETGAHAKDNAASNWLDPETGLLRHLGFAFHVDVAAAYKIIAETFSEQDICDLTEVSMFPPQKTVSIMQKN

SPMRKVISYGLRRVTETGILTYHFNVWHSRKPPCVKKIETSDLHVDmelDTVSSALLILLFSYAITLMILGT

EILYSKWHNRIQLKWVGAT

>DmelIR67b

MELLYLNTLQSLSLLEGNRLVQTVQELNNIYQTELNVFLEFGNGADILESAQGTFVPTLWIKNPQNQKVM

KGNFTSCTLTILYLEDEHLDRGLYYLANWLWEYHHLEVLIFFNGGSYDKLIQIFSRCFNEGFVNVLVMLP

GSDELYTFMPYQDLKILNLKSIKEFYSLSRKKMDLNGYNITSGLVIAgamAPRWFSFRDRQNRLILTGYMLR

MIVDFTNHFNGSVRLMNVLTVNDGLELLANRTIDFFPFLIRPLKSFSMSNILYLENCGLIVPTSRPLPNW

VYLLRPYAFDTWIAWLIMLIYCSLALRILSKGQISISAAFLKVLRLVMYLSGSRDmelGTRPTTRRLFLFVI

LTTSGFILTNLYVAQLSSNSAAgamLYEKQINTWEDLDKSDSIWPLIDVDIKTMEKLIPDRTKLLKKIVPTL

EADVDTYRRNLNTSCIHSGFFDRIDFALYQQKFLRFPIFRKFPHLLYQQPLQISAAFGRPYLQLFNWFVR

KIFESGIYLKMKDDAYRHGIQSGLLNLAFRDRHLEVKSNDVEYYYLIAgamLWFGGLTLATVCFLLELLIGY

AKIKVTISCKMNIM

>DmelIR67c

MFCWLIFLNIILLSDRSESWSAREVIHQFNHDQQLQLNIYLDCNDVELQIGQEVSNLFVNSTADKMKILG

RFSSHSLIIACFKDSTRNRTLNGVKELLWGLQYLPILFVVDSNMDFYFQQALRHGFIHVLALNFMNGSLY

TYKPYPKVEVHQIKDmelQKFYKLTKLRNLQGQAVRTTVETMTPRCFRYRNRHGQLVYAgamYMYRMVKEFIST

YNGTEEHVFGNVDTVPYKEGLAALKNGEIDmelMPRIIHALEWYYFYRSHILYNIKTYIMVPWAEPLPKSLY

FIQPFRGTVWITIMVSFVYASIVIWWIRYRQQGNSSLTQSFMDVLQLLFQLPLSKIWHFNMGTHQVVSFI

VLFVFGFMLTNLYTAQLSSYLTTGLFKSQINTFDDLFREKRTLLVESFDAEVLHNMTKEKIIQKEFESII

LITSIEEVFKHRKSLNTSYAYEAYEDRIAFELSQQRYLRVPIFKILKEVYDQRPVFVALRHGLPYVELFN

NYLRRIFESGIWIKLQEDSFLEGIASGEISFRKSKSREIKIFDKDFYFFAYILLGMGWCVSTIALFLELW

SFKYSVTNVLHEG

>DmelIR68a

MRCLWILIVAFISLAMATSIPIPIANPAPLSGYEMQLKILLQKILWVANVKRCFAVITDDLHYPIYDRIF

FESVGRRVIPFFVMRTNESDDLQRPSRQVELFVKAIKSSDCELNVITILNGWQVQRFLGYIYDNRSLNMQ

KKFVLLHDLRLFESDmelIHLWSVFIDAIFLKRQLDNKYTISTIAFPGILSGVLVMKNIANWELGKGLNGRI

LFADKTSNLFGTSLPVAISEHVPMVLWANATKSFQGVEVEIMNALGKALNFKPVYYKPNQTENMDWTELD

GGASVAYGSGNPDGYAQNGTHIDSMLVDEVAAHSARFAIGDLHLFQVYLKLVELSAPHNFECLTFLTPES

STDNSWQTFILPFSAgamMWVGVLLSLFVVGTVFYAISFLNAIINGNVSSEFFRCLRPNRNVPMDPKIYRRI

SFRIAISRYRSSKGDRMPRDLFDGYTNCILLTYSMLLYVALPRMPRNWPLRVLTGWYWIYCILLVATYRA

SFTAILANPAARVTIDTLEDLLRSHIPPSTGATENRQFFLEANDEVARKVGEKMEVFGYSDDLTSRIAKG

QCAYYDNEFYLRYLRVADESGSALHIMKECVLYMPVVLAMEKNSALKPRVDASIQHLAEGGLIAKWLKDA

IEHLPAEALAQQEALMNIQKFWSSFVALLIGYVISMLTLLAERWHFKHIVMKHPMYDVYNPSLYYNFKRI

YPQH

>DmelIR68b

MKFLVGLLLQWYLPGIYALAEIACRIAVEQNVQVTYLYRCASCPASFDADYSALELDLYRCVGSRLPVIT

RNMEAHELEPFRRTDSLSIFQIPAAEKGDSLVRRILDmelLNPHQRRKHMHKYLFVWPNAgamRHQLLRLFRGS

WAKKLLYGLAITGRENGTFDFDPFAWGGLQVIQRLDGEVPYARKVKDLRGYPLRFSMFTDPLMAMPRSPV

ETAgamYQAVDGVAARVVGEMLNASVTYVFPEDNESYGRCLPNGNYTGVVSDIVGGHTHFAPNSRFVLDCIW

PAVEVLYPYTRRNLHLVVPASAIQPEYLIFVRVFRRTVWYLLLVTLLVVVLVFWVMQRLQRRIPRRGVIQ

FQATWYEILEMFGKTHVGEPAgamRLSSFSSMRTFLMGWILFSYVLSTIYFAKLESGFVRPSYEEQVDRVDD

LVHLDVHIYAVTTMYDAVRSALTEHQYGLLENRSRQLPLGIATSYYQPVVRRRDRRAAFIMRDFHARDFL

AITYDSQAERPAYHIAREYLRSMICTYILPRGSPFLHRLESLYSGFLEHGFFEHWRQMDLITRVGASPDA

EEFLEDLGDQTDTDSGSNELAIRNKKVVLTLDILQGAFYLWSVGIGISCLGFAVEHAHWFWRRQTLRNAV

EARTS

>DmelIR75a

MQLVQLANFVLDNLVQSRIGFIVLFHCWQSDESLKFAQQFMKPIHPILVYHQFVQMRGVLNWSHLELSYM

GHTQPTLAIYVDIKCDQTQDLLEEASREQIYNQHYHWLLVGNQSKLEFYDLFGLFNISIDADVSYVKEQI

QDNNDSVAYAVHDVYNNGKIIGGQLNVTGSHEMSCDPFVCRRTRHLSSLQKRSKYGNREQLTDVVLRVAT

VVTQRPLTLSDDELIRFLSQENDTHIDSLARFGFHLTLILRDLLHCKMKFIFSDSWSKSDVVGGSVGAVV

DQTADLTATPSLATEGRLKYLSAIIETGFFRSVCIFRTPHNAgamLRGDVFLQPFSPLVWYLFGGVLSLIGV

LLWITFYMECKRMQKRWRLDYLPSLLSTFLISFGAACIQSSSLIPRSAgamGRLIYFALFLISFIMYNYYTS

VVVSSLLSSPVKSKIKTMRQLAESSLTVGLEPLPFTKSYLNYSRLPEIHLFIKRKIESQTQNPELWLPAE

QGVLRVRDNPGYVYVFETSSGYAYVERYFTAQEICDLNEVLFRPEQLFYTHLHRNSTYKELFRLRFLRIL

ETGVYRKQRSYWVHMKLHCVAQNFVITVGMEYVAPLLLMLICADILVVVILLVELAWKRFFTRHLTFHP

>DmelIR75b

MLQLHNLILHNLIHMAKLSHVLILHCSLSHLALLAQSKNIFTQFQPLHSDIQLNDDFLNHNILKLGVFLD

INCDKSGTVLDmelASAKRFFSHRYHWLIYDRSMNFSVLESHFKEAQIFVDADVTYVTHDPFSKNFLLYDVY

NKGRQLGGELNITADREIFCNKTNCRVERYLSELYTRSALQHRKSFTGLTMRATAVVTALPLNVSIKEIF

DFMNSKYRIQLDTYARLGYQARQPLRDmelLDCKFKYIFRDRWSDGNATGGMIGDLILDKADLAIAPFIYSF

DRALFLQPITKFSVFREICMFRNPRSVSAgamLSATEFLQPFSGGVWLTFALLLLLAgamCLLWVTFILERRKQ

WKPSLLTSCLLSFGAgamCIQGAWLTPRSMGGRMAFFALMVTSYLMYNYYTSIVVSKLLGQPIKSNIRTLQQ

LADSNLDVGIEPTVYTRIYVETSEEPDVRDLYRKKVLGSKRSPDKIWIPTEAgamVLSVRDQEGFVYITGVA

TGYEFVRKHFLAHQICELNEIPLRDASHTHTVLAKRSPYAELIKLSELRMLETGVHFKHERSWMETKLHC

YQHNHTVAVGLEYAAPLFIILLGAIILCMGILGLEVIWHRHCTLH

>DmelIR75c

MTSWPLYRLIVFNLLEINLSNLMVFHCWSIKEAFPLVEMLNQNGIFSQYIDVQNPDNLANVHKEYLDSDL

VRLGVFLDLGCDKAELVTNQSSRARLYNQNLHWLLYDEAgamNFTKLTQLFEGANLSLNADVTYVSREDEER

FILHDVYNKGSHLGGKLNITVDQTLQCNRSHCQVKEYLSELHLRPRLQHRMDLSSVTFRLAALVSVLPIN

SSEEELLEFLNSDRDSHMDSISRIGNRLIMHTQEILGFKLHYIWCGTWSVQDAFGGAIGMLTNESAELCT

TPFVPSWNRLHYLHPMTEQAQFRAVCMFRTPHNAgamIKAAVFLEPFMPSVWFAFAgamLLIFAgamVLLWMIFHL

ERHWMQRCLDFIPSLLSSCLISFGAACIQGSYLMPKSAgamGRLAFIAVMLTSFLMYNYYTSIVVSTLLGSP

VRSNIRTIQQLADSSLDVGFDTVPFTKTYLVSSPRPDIRSLYKQKVESKRDPNSVWLSPEEGVIRVRDQP

GFVYTSEASFMYHFVEKHYLPREISDLNEIILRPESAVYGMVHLNSTYRQLLTQLQVRMLETGITSKQSR

FFSKTKLHTFSNSFVIQVGMEYAAPLFISLLVAYFLALLILILEICWARYAKKKFSTIIPQNQ

>DmelIR75d

MKVQVAHWLPLIFFLLVSGTPRVAgamSWRSEYSRQDPDPKTRWGNQLPDmelLVAYYRHHGVHSLMLVVCHTD

IADFRLWKLWQHFNLNNFYVQVSTESSLRDLQHVDALDEHKDAPPPKSFHANNSTHWETSFLLPALPYKM

GILLLEFSSECALNLLRWSAASEHNYFTTNRFWLLLTEDPGDIDLLEDPEIFIPPDSELRVLHYENVGNF

SCSLIDLYKVAAWKPLKRTLVGHNIRNSRHVIHALQHFGSAITYRQDLEGIVFNSAIVIAFPDLFTNIED

LSLRHIDTISKVNHRLMLELANRLNMSYNTYQTVNYGWRQPNGSFDGLMGRFQRYELDLAQLAIFMRLDR

IALVDFVAETYRVRAgamIMFRQPPLSAVANIFAMPFENDVWVSILMLLIITTVVLVLELFFSPHNHDmelSYM

DTLNFVWGAMCQQGFYVEVRNRSARIIVFTTFVAALFLFTSFSANIVALLQSPSDAIQSLSDLGQSPLEI

GVQDTQYNKIYFTESTDPVTKNLYHKKIASKGENIYMRPLLGMEKMRTGLFAYQVELQAgamYQIVSDTFSE

PEKCGLMELEPFQLPMLAIPTRKNFPYKELIRRQLRWQREVSLVNREERKWIPQKPKCEGGVGGFVSIGI

TECRYALGIFGCGAAVSFVLFLFEFIFRHFKQVYRIIKGYREVQR

>DmelIR76a

MFVYTKEFEDKKDSYLSGYIFQDQPNILVITSQYLNSSTFEIKTNRFVGPRNFNKNPEPVEFYILQRFDA

KGTKATWETQSAMSSKMRNLKGREVVIGIFDYKPFMLLDYEKPPLYYDRFMNTTDVTIDGTDIQLMLIFC

ELYNCTIQVDTSEPYDWGDIYLNASGYGLVGMILDRRNDYGVGGMYLWYEAYEYMDmelTHFLGRSGVTCLV

PAPNRLISWTLLLRPFQFVLWMCVMLCLLLESLALGITRRWEHSSVAAgamNSWISSLRFGCISTLKLFVNQ

STNYVTSSYALRTVLVASYMIDIILTTVYSGGLAAILTLPTLEEAADSRQRLFDHKLIWTGTSQAWITTI

DERSADPVLLGLMEHYRVYDANLISAFSHTEQMGFVVERLQFGHLGNTELIENDALKRLKLMVDDIYFAF

TVAFVPRLWPHLNAYNDFILAWHSSGFDKFWEWKIAAEYMNAHRQNRIVASEKTNLDIGPVKLGIDNFIG

LILLWCFGMICSLLTFLGELWRGQG

>DmelIR76b

MATGIELLVAAALCVACPPLNDSPPTNLIQMGENGTLSPVTELPMDVDASEAgamFDADAPVETLETINRKK

PKLREMLDWIGGKHLRIATLEDFPLSYTEVLENGTRVGHGVSFQIIDFLKKKFNFTYEVVVPQDNIIGSP

SDFDRSLIEMVNSSTVDLAAAFIPSLSDQRSFVYYSTTTLDEGEWIMVMQRPRESASGSGLLAPFEFWVW

ILILVSLLAVGPIIYALIILRNRLTGDGQQTPYSLGHCAWFVYGALMKQGSTLSPIADSTRLLFATWWIF

ITILTSFYTANLTAFLTLSKFTLPYNTVNDILTKNKHFVSMRGGGVEYAIRTTNESLSMLNRMIQNNYAV

FSDETNDTYNLQNYVEKNGYVFVRDRPAINIMLYRDYLYRKTVSFSDEKVHCPFAMAKEPFLKKKRTFAY

PIGSNLSQLFDPELLHLVESGIVKHLSKRNLPSAEICPQDLGGTERQLRNGDLMMTYYIMLAgamFATALAV

FSTELMFRYVNSRQEANKWARHGIGRTPNGQSVAPSRWLRGWRRLNSGHGQLLGASTHGQNVTPPPPYQS

IFNGGSHGDPLNRWRRPLANGNALGNGVLLGGDSEGGVRRLINGRDYMVFRNPNGQSQLVPVRSPSAALF

QYSYTE

>DmelIR7a

MFHHLWLLMGLRSLAMGALHPPQPEAMTPLVAAALEILAEQVSPSQSTLAVMDLTQDAEHRDERQEQLMT

IILRSVGSEMALRTFQKPPAEVPASFVVFLVNSAQAFNTLGFHFTDIHSTREFNFLILLTHRMSSRAERL

QVLRDISRTCVRFHTSNVILLTEKRDGVVLVYAYRLLNMDCDLSVNLELIDIYKNGLFRHGHEARSFNRV

LSLSGCPLQVSWYPLPPFVSFIGNSSDPEERAQIWRLTGIDGELIKLLASIFDFRILLEEPCNKCLSPDI

KDDCSGCFDQVIISNSSILIGAMSGSHQHRSHFSFTSSYHQSSLVFIMHMSSQFGAVAQLAVPFTVIVWL

ALVVSSLLLVLVLWMRNRLVCGRSDLASHALQVLTTLMGNPLEARSLPRSSRLRILYAgamWLLLVLVLRVV

YQGKLFDSFRLPYHKPLPTEISELIRSNYTLINQEYLDYYPRELTVLTRNGSKDRFDYIQGLGKEGKFTT

TSLIATMEYYNMMHWSTSRLTHIKEHIFLYQMVIYLRRHSLLKFAFDRKIKQLLSAgamIIGYFVREFDACQ

YRKPFEEDYEVTPIPLDSFCGLYYISLIWLSAAVVAFILELLSQRIVWLRRIFE

>DmelIR7b

MKYWLYILSCCSLVASTMESSSDWDLAEALAQVVANSEMGRFKTLYIYTHTNSQSTGGHLEELLDQVLMI

VPNNLQARRLLLQQSMEYKPYVHAVLALVDGLPSLSAIYARIRATQDLSHTLIYMSMPTDAYGEEMQATL

RFLWRLSVLNVGVVLRPPGDHILMVSYFPFSALHGCQVISANVVNRYQVGTKRWASQDYFPSKLGNFYGC

LLTCATWEDmelPYLVWRPDGSGSFVGIEGALLQFMAENLNFTVGLYWMNKEEVLATFDESGRIFDEIFGHH

ADFSLGGFHFKPSAgamSEIPYSQSTYYFMSHIMLVTNLQSAYSAYEKLSFPFTPLLWRAIGLVLILACLLL

MLLVRWRHHHELPRNPYYELLVLTMGGNLEDRWVPQRFPSRLVLLTWLFATLVLRSGYQSGMYQLLRQDT

QRNPPQTISEVLAQHFTIQLAEVNEARILASLPELRPEQLVYLEGSELQSFPALAQQSGSSARVAILTPY

EYFGYFRKVHPMSRRLHLVRERIYTQQLAFYVRRHSHLVGVLNKQIQHAHTHGFLEHWTRQYVSAVDEKD

ESVARIASTSYSTLDGIDGDPSLSESEEDQQVAPVRQNVLSMRELAALFWLILWANLGAVVVFVLELLLP

RIKLRKILRKMKSDIKKQISKLVRK

>DmelIR7c

MLHSAVHNVSLVYALVWAIDNYYGMATSTPLAVVQFPTSRESRRLHNDLIDAALGRSSGTGRIQFLLEDD

RVEMTETDTDPPPPSGLTGRPIAIWFLDSLRSYFRLEMYLNQLGSPYKRNGFFLVIYTGLEDQPMESLKI

MFRRLLNMYVLNVNVFLQRDGTVHLYTYYPYGPHHCQSSLPVYYTAFQDLAAPANGFGLTKPLFPRKLTN

MHGCEMVVATFEHRPYVIIEDDPKTPGGRSIHGIEGLIFRSLAERMNFTIKLVEQKDKNRGEILPDGNFT

GILKMMVDGEVNLTFVCFMYSKARSDLMLPSTSYTSFPIVLVVPSGGSISPMGRLTRPFRYIIWSCILVS

LIFGFVLICLLKITALPGLRNLVLGRRNRLPFMGMWASLLGGLALYNPQRNFARYILVMWLLQTLILRAA

YTGQLYLLLQDVEMRSPIKSLSEVLAKDYEFRILPALRTIFKDSMPTTNFHAVLSLEESLYRLRDEDDPG

ITVALLQPTVNQFDFRSGPNKRHLTVLPDPLMTAPLTFYMRPHSYFKRRIDRLIMAMMSSGIVARYRKMY

MDRIKRVSKRRNLEPKPLSIWRLSGIFVCCAgamLYLVALIVFILEILTTNHRRLRRAFNVINRYAA

>DmelIR7d

MDIRCVVALLLGLCKVQAVVWPHQHLLEEQLASQISATLQKIFINGLAVYNFGVFISTSYEEMDRDRVIL

VHQVLNRNLYPPNFPVAVVLASKMNRKITAQVFTQLLFVQNAEQAIAIAEGVNRNGLCVIVLLTSQPERP

IMTKIFTYFMQERYNINVVILVPRLHGVQAFNVRPYTPTSCSSLEPVEIDIKDGDLWDVFPRRLKNLHGC

PLSVIVWDIPPYMRINWKSSDPMDGLDGLDGLLLRIVARKMNFTLKLIPNEPNGLIGGSSFMNGTFTGAY

KMLRERRANITIGCAACTPERSTFLEATSPYSQMSYIIVLQARGGYSIYEVMLFPFEKYTWLLLSTILGL

HWIVGSRWRMPSPILAgamWMLWIFVIRASYEASVFNFIQNSPVKPSPRTLDQALSGGFRFITDHASYRMTL

KIPSFQGKTLISAgamQPVDVFDALLKAPWKTGAFTSRAFLADHLVRHRKHRNQLVILAEKIVDNMLCMYFP

HGSYFAWEINKLLFNMRSFGIFQHHSQILAWDNLPTTTDTDTPGKRIHSSTESVATGFAESMSFVVAALN

CLMGALCISIVVFGLELLSRRRHWTGLEWLFERV

>DmelIR7e

MNISALLNSYYDLSGEQMNHINEFVARAVLHVVHHYILSVTPSLVLTLCCRSNHTCNFYNKMMSTLFREW

GLAPLQIVNVLRGVPWHPVPGRRHFNVIFTDSFAAFEEIRMEYYSREYNYNEHYFIFLQARDRLLQGEMR

LIFDYCWRYRLIHCSIQVQKSNGDILFYSYYPFGEHGCSDmelEPQLINRYNGSMLVEPDLFPRKLRNFFGC

PLRCALWDVPPFLTLDEDQEEVLRVNGGYEGRLLLALAEKMNFTIAVRKVHVNMRDEALEMLRRDEVDLT

LGGIRQTVARGMVATSSHNYHQTREVFGVLASSYELSSFDILFYPYRLQIWMGILGVVALSALIQLIVGR

MLRERMGSRFWLNLELVFVGMPLLECPRSHTARLYCVMLMMYTLIIRTIYQGLLYHLIRTHQLNRWPQTI

ESLVQKNFTVVLTPIVQEVLDEIPSVQHMRFRLLEANSELDPLYFLEANHQLRQHVTASALDIFIHFNRL

SADKVHQRGEQGSGAHFEIVPEDIISMQLTMYLAKHSFLIDQLNEEIMWMRSVGLLSVWSRWELSESYLR

NEQSFQVLGTMELYAIFLMVLVGLIVGLLVFILELVSMRSIYLRKLFT

>DmelIR7f

MNTTSDSNAgamSSLSSGSGYSIYKSYLENSRIDmelQGEDANLYVARALRLVIENVLAQLSTTLVVTISTRHL

GTAHWFEYMMNILMDSWRMVAVQLLRIRPDLVVNPVPGRKRVSLLMVDSYQGLLDTNITASNANFDDPDY

YFIFLQARDHLIPKELQLILDHCLAHFWLHCNVMIQTAQVEVLVYTYYPYTADACQKAYPIPVNTFDGRK

WKASQMFPDKLSQMHGCPLTVLTWHQPPFVELVWDPKHNRSRGSGFEIQLVEHLARRMNFSLELVNIALL

RPNAYRLAEGSSEGPIEKLLQRNVNISMGYFRKTARRNQLLTTPMSYYSANLVAVLQLERYRIGSLALLV

FPFELSVWMLLLLALLIHLGIHLPSARRGNEEDGGGGLQVVALLLGAALARLPRSWRHRFIAAHWLWASI

PLRISYQSLLFHLIRLQLYNTPSFSLDQLLAEGFQGICTANTQRLLLEMPQLARDPDSIQSVDTPFDWDV

LNVLTRNRNRKIFAVANQDVTLSFLHSSAHPNAFHVVKQPVNVEYAgamMYMPKHSFLYEKMDDDIRRLDAS

GFIHAWRRASFASVHRKEQVHMTSRRYINHAKLSGIYMVMAgamLYLLAgamLLFAgamEVLLRQRN

>DmelIR7g

MNVTSLLNFESMKYIGAQTQAASINHHVAQALRVFIEDFYQRIAPAFIVVLSCRRPSPMNFYRNIMQLLY

ESVDTMIVQLVLVELGRPRRIAgamPRTHNLLLVDSLDALLDIEIHTYTAQSDTSEYYFIFLQQRDALIPHD

MQGVFAYCWRHQLINCNVMTQSSGGQVLLHTYFPYAPGQCNDSQPTRINMFLGESWKHRDYFPSKLHNLN

GCPLIVLARKVSPFLDLDEGQRELRGLEGRLLQELSRRMNFSIQFSGLQDQLKNRTTWTEKQLLQKLVQE

RIAHLAIGYVRKRIQYATNLTPVFPHYSNRVVGCLLLNAHNLTSLEIWSFPFQALTWICLLLFASWLIFG

LIVRSMYSALLFFILRYHLHQRLPGNLQDLTHGDYAAVMGRTTLQDLREVPSLQDLLGLKSVIVTSEREE

EVLRTLDRCTLREGAgamSHPLFFGLISQDALLHLTQRGHRAgamAYHIIPQDVLEQQLAIYLQKHSHLASHLD

HLVMSIRSVGLVHHWAgamQMASERYFRSRFLYREKRIRQPDLWAVYILTAgamLYLLSLVVFICELLASRRAgam

L

>DmelIR84a

MIKLQVKVISWPLIILTAFLRVLQIESINTNFLELAAFEDFLRSEHLSHVLVVRGDDADGDWKIECHQKL

LANYRVQFYRPEMSANFEDLMFYGSPRTAVLVLNSEHVLVRRQVFGVASEAgamYFNNSLAWFILGSGRESL

PVEQLIDQLLSGYRMGIDADITVALRGPDNASMLFYDVYRISRQANTPLIIEKKGLWTHSGGYQKFGNFK

NTWVIRRRNFLNVTLIGSTVLTEKPPGFGDmelEYLADDKQLQQLDPMQRKTYQLFQLVERMFNLSLAISLT

DKWGELLDNGSWSGVMGQVTSREADFAVCPIRFVLDRQPYVQYSAVLHTQNIHFLFRHPRRSHIKNIFFE

PLSNQVWWCVLALVTGSTILLLFHVRLERMLSNMENRFSFVWFTMLETYLQQGPANEIFRLFSTRLLISL

SCIFSFMLMQFYGAFIVGSLLSESARSIVNLQALYDSNLAIGMENISYNFPIFTNTSNQLVRDVYVKKIC

KSGEHNIMSLQQGAERIIQGRFAFHTAIDRMYRLLLELQMDEAEFCDLQEVMFNLPYDSGSVMPKGSPWR

EHLAHALLHFRATGLLQYNDKKWMVRRPDCSLFKTSQAEVDLEHFAPALFALALAMVASALVFLLELFLH

WLPDFRRRLGTMST

>DmelIR85a

MSIQWLKHILLLAILVNLAgamTRENHIPLDLKKSSIVMVKMSQILCKARIKVLFVYFENQTSHEHTGQILK

EVTKCDISNQNTPLEAVKDDGILMYMVMITTNISQPLELSLIRKKSAAKHRSHVFLLVRDADTVSDAWMR

ASFRQFWKIWLLNIVILYWRDGRLNAYRYNPFMDNYLIPVDNKPNEVPTLEQLFPKTIPNMQRKPLRMCI

YKDDVRAIFWRQGTILGTDGLLAAYVAERLNATMMITRPHSYNNHNLSSDICFLEVAKEYVDVAMNIRFL

VPDTFRKQAESTVSHTRDDLCVIVPKAKTAPTFWNIFRSFGSLVWALILVSVLVANVFCYILKSEVGRVP

MQLFAgamALTMPMTQIPPNHSIRLFLIFWLYFGLLICSAFKGNLTSMMVFQPYLPDINQLGALARSHYHII

IRPRHVKHIQHFLTLGHKHESRIREQMLEVSDTQMYEMMRNNDIRFAYLEKYHIARFQVNSRVHMHLGRP

LFHLMNSCLVPFHAVYIVPYGSPYLGFLDSLIRSSHEFGFERYWDRIMNSAFIKSGVKVVNRRRGSGNDE

PVVLKLQHFHAVFALWLVGIGMACIVLAWEHLTHNYNLAVTKRRD

>DmelIR87a

MSTPEQRFWLAALLFLLSQHSEVRGFGINLMKVQTEDKGQEACILALLRKYFDSGDGLSGSVLCINRNYQ

LPNIEEQLLRGVNNYENYPWSLLITNSREGPSPAKFLMNEKPQCYFLIVDNLEDEDLDEVFEHWKGMVNW

NPLAQFVVYLASLEETDEEMNDLMVELLLTFINKKIFNVNVIGQSEENQFYYGKTVFPYHPDNNCGNRVI

SVELLDACDYPSEETDSEDENDEDEGDGAQEEDDGPQEEGDGEQEEEDGPQEQEDGDQAKGDEGQENDDG

GLENKVENEFRIGASDDDELENDLSSNSSEPEAIIEEFFRAKFEDKFPRDLSGCPLTASFRPWEPYIFRN

SEEQPVDDYYYGLQGDEDDYNDTSPNYGESDDESYADPGEDGDGAIPDTETQSGGKLKLSGIEYEMVQTI

AERLHVSIEMQGENSNLYHLFQQLIDGEIEMIVGGIDEDPSISQFVSSSIPYHQDELTWCVARAKRRHGF

FNFVATFNADAgamFLIGIFVVTCSLVVWLAQRVSGFQLRNLNGYFPTCLRVLGILLNQAIPAQDFPITLRQ

LFALSFLMGFFFSNTYQSFLISTLTTPRSSYQIHTLQEIYSNKMTVMGTSEHVRHLNKDGEIFKYIREKF

QMCYNLVDCLNDAAQNEHIAVAVSRQHSFYNPRIQRDRLYCFDRRESLYVYLVTMLLPKKYHLLHQINPV

IQHIIESGHMQKWARDLDmelRRMIHEEITRVREDPFKALTFDQFRGAIAFSGGLLLVASCVFAFELCYVKY

VYRTEKRERKTKKITKKVHNIKIQHD

>DmelIR8a

MELPLLVLLLALRFAgamSEVLKITFWIEPVQRAEFDTDIAMVLKELDALRLDVKVDDTTLTLTRSEDGLDmel

QRFCEILSTVGASAVIDLTYSHWEEGYNLVRSLGIGYVRLERIMRPFLDmelFGDFMRQKRANNVAMVFMNA

RDAVEAMQQMLVGYPFRTLIMDASQTDPGQHFLERIRSLRPAPTYIALFARAAAMNGIFEKVQKADLFQR

PLEWHFVFLDTRDRVFKYRRQAELCTRFTLNPRAICRSMPMPDLYCGSGFTMQRAMLLNVLRSLINAAQV

SPGYPLAIYQDCNATASSSEVSDPLEKDDYNWLDmelVHWSNFLAYAPPLPHIQDQFQSPVPGLTFAVNISA

GYYSSEHEAKTDLAAWSSVGEMRLLNETISPARRFFRIGTAESIPWSYLRREEGTGELIRDRSGLPIWEG

YCIDFIIRLSQKLNFEFEIVAPEVGHMGELNELGEWDGVVGDLVRGETDFAIAALKMYSEREEVIDFLPP

YYEQTGISIAIRKPVRRTSLFKFMTVLRLEVWLSIVAALVGTAIMIWFMDKYSPYSSRNNRQAYPYACRE

FTLRESFWFALTSFTPQGGGEAPKAISGRMLVAAYWLFVVLMLATFTANLAAFLTVERMQTPVQSLEQLA

RQSRINYTVVKDSDTHQYFVNMKFAEDTLYRMWKELALNASKDFKKFRIWDYPIKEQYGHILLAINSSQP

VADAKEGFANVDAHENADYAFIHDSAEIKYEITRNCNLTEVGEVFAEQPYAVAVQQGSHLGDELSYAILE

LQKDRFFEELKAKYWNQSNLPNCPLSEDQEGITLESLGGVFIATLFGLVLAMMTLGMEVLYYKKKQNALE

ITQVRPVNDSSGSGGNSSTAPPTATSTTKQAWHIPVLEAEEKPAKVSPPPSFETATFRGKKLPARITLGD

GKFKPRHGLYARRNLGASDSHSGYME

>DmelIR92a

MLLQPLVMHLSQLLRIIVGQYFAEFPSILIVYNNSASTTPLQLEYLSALELVLRELSKPIRLQWINVAFL

KDLNDLEDQVMGALNSSVTEGFITILSQTHHFIHARYYATRNANVRLKDKRYLFLCEDESPAELLCMDIL

QFYPHHLMVRPGTETAPTGPTGPHPDPRRGGGASVSTKNKDDGEGGAgamNKTTSPYRDINFELWTQKFVGA

VGNLDALLLDAFLPNETFANRVELYPNKLLNLQRRSLLVGSITYVPYTITNYVPAgamQGDVDPIHPQWPNR

SLTFDGAEANVMKTFCQVHNCHLRVEAYGADNWGGIYDNESSDGMLGDIYEQRVEMAIGCIYNWYDGITE

TSHTIARSSVTILGPAPAPLPSWRTNIMPFNNRAWLVLISTLVICGTFLYFMKYVSYRLRYSGTQVKFHH

SRKLEKSMLDIFALFIQQPSAPLSFDRFAPRFFLATILCATITLENIYSGQLKSMLTFPFYSAPVDTIEK

WAQSGWKWSAPSIIWVHTVQSSDLETEQILARNFEVHDYSYLSNVSFMPNYGFGIERLSSGSLSVGDYVS

TEALENRIVLHDDLYFDYTRAVSIRGWILMPELNKHIRTCQETGLYFHWELEFIDKYMDKKKQEVLMDLA

NGHKVKGAPQALDVRNIAgamALFVLAFGVAFAgamCALVAELLIHRMDLSK

>DmelIR93a

MNPGEMRPSACLLLLAgamLQLSILVPTEANDFSSFLSANASLAVVVDHEYMTVHGENILAHFEKILSDVIR

ENLRNGGINVKYFSWNAVRLKKDFLAAITVTDCENTWNFYKNTQETSILLIAITDSDCPRLPLNRALMVP

IVENGDEFPQLILDAKVQQILNWKTAVVFVDQTILEENALLVKSIVHESITNHITPISLILYEINDSLRG

QQKRVALRQALSQFAPKKHEEMRQQFLVISAFHEDIIEIAETLNMFHVGNQWMIFVLDmelVARDFDAgamTVT

INLDEGANIAFALNETDPNCQDSLNCTISEISLALVNAISKITVEEESIYGEISDEEWEAIRFTKQEKQA

EILEYMKEFLKTNAKCSSCARWRVETAITWGKSQENRKFRSTPQRDAKNRNFEFINIGYWTPVLGFVCQE

LAFPHIEHHFRNITMDILTVHNPPWQILTKNSNGVIVEHKGIVMEIVKELSRALNFSYYLHEASAWKEED

SLSTSAgamGNESDELVGSMTFRIPYRVVEMVQGNQFFIAAVAATVEDPDQKPFNYTQPISVQKYSFITRKP

DEVSRIYLFTAPFTVETWFCLMGIILLTAPTLYAINRLAPLKEMRIVGLSTVKSCFWYIFGALLQQGGMY

LPTADSGRLVVGFWWIVVIVLVTTYCGNLVAFLTFPKFQPGVDYLNQLEDHKDIVQYGLRNGTFFERYVQ

STTREDFKHYLERAKIYGSAQEEDIEAVKRGERINIDWRINLQLIVQRHFEREKECHFALGRESFVDEQI

AMIVPAQSAYLHLVNRHIKSMFRMGFIERWHQMNLPSAgamKCNGKSAQRQVTNHKVNMDDmelQGCFLVLLLG

FTLALLIVCGEFWYRRFRASRKRRQFTN

>DmelIR94a

MALPKQLKFINIFLVLLIIYGSSDGTENQHEIFLNRLLQAVHNERSVETLFLLHHSNLANCSLQDWNPPR

IPTIRSNELTVFNVEKTFNHNALALVCLMKNSYREILNTLAKSFDCMRQERIILMIHRKSDSKFIEDITH

EVKNLQFLHLIVLIVQEKYNGQVFASTLRLQSFPEPHFKRIRNVFAIQRIFYRPINFHGKVLNAIPNDIP

ILFVALNEMFTEYARRYNSTLRIQNRTIKEDIEITEDNYDIDmelKIQLHNSQNFLHHMNIAMDIGSNSLII

LVPCATELRGLDIFKELGVRTLTWLALLFYIIFVLVEMLFVFISNRFNGRNFTMRYTNPLINLRAVRAIL

GQTSPISNRYSLSIQHFFVFMSLFGTLFGGFFDCKLRSFLTKRPYYSQIENFSELRKSGVTVVVDHTTRQ

FIEQEINANFFRDEVPNVRTTTIQELINHVYSYDRKFAFVANSIPWRTFREEMKSINQKILCDSKNLTIL

ENVPLTFSIRRNAIFSHHLRNFIINAADSGMITCWFKMAgamKVIRKHIKTTLRESEQQPSHLPLSFDHFKW

LWAVLCIAYVMSFMVFVMEILWSKYQRRTRSVSIV

>DmelIR94b

MSLIFNLLFILILSQAVSQETEFLQLKYLNNIVRSMIKLHKMETLVIVKHHLDNNCSLQNWNAHGMGIIR

TNDQGKLIMKDTFNSRTLAIICIGQNSHITLLRNVFETFGKVQQKKIILWTQMELKEKFFQEISKKSRDL

KLLNLLVLKAVTKDKLLIYRLNPFPSPHFKRIENIWTPNDTLFMDTKFNFHGMTAVVKHDYNWTIQMGNI

RKFPISRIEDKEVIEFALKYNLTLQFFNDVERFDIELRKRIILKSNSTQPIDSGIPMVFSSLLIVVPCGN

YLSIQDVIKVSGIEKWIFYIILVYVIFVLIEITFLGVTILISRQSRHQMIPNTLVNLCAFRAILGLPFPE

TRRTSLSLRQLFLAIALFGMIFSIFINCKLSSMLTNPCPRPQVNNFEELKTSGLTVVMDHDAENFIEKEI

GVDFFNQYMPRKVTLTFTERAKLLFSLKGNHAFTLFSESFAIIESYQRSKGLRAHCTSEDLIVAERVPRI

YILENNSILDRPLRRFIRQMQESGITNHWLKNIPSSLEKNLMQITIPYDRERVHPLSIEHLTWLWCILIL

GYSISMIVFFVEMSLKRRKKNLENRAPNICIC

>DmelIR94c

MSKVFKLLVLPLIYLSLTKGSKNPQLKFLRELINVIEEGREIRTIMVIKHSRDEYCHLDQWNPRGSPILR

TNEMGSIRISGYFNDQAVILACMGENSDYGLLKSLANAMDNMRQERIILWSEREPTKMLMDYISQQADRY

NFAQIIIVTMNEDVDAVPSLHQLNPYPTPRFRQITNISNIRRTSFFGCGLSFQGKTAILKESVVSNIRFK

VWSPSGPIPLSELKDYEIVQFAVKYNLSLKLYDQNESKSDHFDIQLGPLFITKDFPTQMAFVSPNTACSL

IVIVPCSPKWRFMDVLHKLGVLKLIGCLLIAYAVFVLIETLILWLTHRISGREVRLTSLNQLLNPRAFRG

ILGLPFPEFRRSSISLRQLFLVISVFGLVYSNFVSCTLSALLTKPAQNPQVRNFKELRDSGLITIMDKYT

HSFIEKHIDPEFFDHVLPHYLILQKKEALRMIWNFNDSYSYVMYTTTWKSLNTVQKSFDERVFCESESLT

IAWNLPRMYVLGNNSVLKWMLSRYITYMPQTGIPDSWTEQLPKVLKLLYNVTSPRRIKEGAVPLSIQHLS

WIWHLLFIGESIATLVFIVEILLQKSNQHTSNMRERSSEDDDFV

>DmelIR94d

MGQLHLLLVALVLLSPGGDSFYHSLIHHLNRELKIEYVLLLGNFDTTWLDILWQLPVSVLQIKEHSRETY

SLLENPSHNVLTIAFVNDSPEDILEILYRNLRMLNTQPVLLVIRKSTIRVNSLLEWCWHHQLLKVVAIAQ

DFMESLIVYSYNPFPVLQFIERRLDNSTVIFEKRLENLHGYEVPIALGGSSPRLIVYRDLEGKLIFSGPV

GNFMKSFEQRYNCRLVQPYPFDESAISPARDLIASVQNGSVQIALGAIYPQVPYTGYSYPIELMSWCLMM

PVPEEVPHSQLYSMVFSPMAFGITIVAMVLISLTLSMALRLHGYRVSFSEYFLHDSCLRGVLSQSFYEVL

RAPALIKAMYLVICLLGLLITSWYNSYFSTFVTSAPRFPQLTSYESIRHSNIKIVIWKPEYEMLLFFSEN

MEKYSSIFQLQEDYKEFLHLRDSFDTRYGYMMPMEKWSLMKEQQRVFSSPLFSLQDDLCVFHTVPIVFPM

VKNSIFKEPFDRLILDVTATGLLSRWRDmelSFTEMIKAgamQLGLEDRGHPKEFRAMKVGDLIQIWRFVGWML

GLATIVFLLELICFWRHKMWQNMKYMFCRNKNI

>DmelIR94f

MWQQVLLAETSNWFRSDVLQRFWTHLRVEIRFRTMLNYRLESCDCWFDNVLGSDNSTALLWNDQTYPHYL

RRRQDTDILVVSCLRFHQYQEVLLALSLMLDQMRSMPVVLQLCGDEDSMQELNSARLLLKHSQDLKMPNV

VLLSSTFFTSATLYSYEMFPEFNVQKLVYQAYLTLFPYKLGNLKGHPIRTVPDNSEPLTIVRKTLNGSIA

IDGLVWQFMIEFAKHINATLQLPIEPHPEKSIKLVQILDLVRNQTVDIAASLRPYSLNVQRSSTHIYGSP

MMVGNWCMMLPTERVIGSHEALTRLMKSPWTWLILLLFYSVHRFLAQKTRLRSSLIHLIKLLINLSLICF

LQAQLSAYFIGPQKVNHISNMQQVEESGLKIRGMRGEFMEYPIDmelRSRYASSFLLHDLFFDLAQYRNSLN

TSYGYTVTSVKWELYKEAQRHFRRPLFRYSEEICVQKLSLFSLIQQSNCIYCYRSRIFILRMHEAgamLIRL

WYRRSYYVMVTAgamRFPIGDLSTVHRAQPIRWTEWQNVVLLHGVGLLFSVVVFVIELTVHYANVCLNNL

>DmelIR94f

MWQQVLLAETSNWFRSDVLQRFWTHLRVEIRFRTMLNYRLESCDCWFDNVLGSDNSTALLWNDQTYPHYL

RRRQDTDILVVSCLRFHQYQEVLLALSLMLDQMRSMPVVLQLCGDEDSMQELNSARLLLKHSQDLKMPNV

VLLSSTFFTSATLYSYEMFPEFNVQKLVYQAYLTLFPYKLGNLKGHPIRTVPDNSEPLTIVRKTLNGSIA

IDGLVWQFMIEFAKHINATLQLPIEPHPEKSIKLVQILDLVRNQTVDIAASLRPYSLNVQRSSTHIYGSP

MMVGNWCMMLPTERVIGSHEALTRLMKSPWTWLILLLFYSVHRFLAQKTRLRSSLIHLIKLLINLSLICF

LQAQLSAYFIGPQKVNHISNMQQVEESGLKIRGMRGEFMEYPIDmelRSRYASSFLLHDLFFDLAQYRNSLN

TSYGYTVTSVKWELYKEAQRHFRRPLFRYSEEICVQKLSLFSLIQQSNCIYCYRSRIFILRMHEAgamLIRL

WYRRSYYVMVTAgamRFPIGDLSTVHRAQPIRWTEWQNVVLLHGVGLLFSVVVFVIELTVHYANVCLNNL

>DmelIR94g

MACLSSTSYDGQLQLLAESLTRYRSVRVLIEVQDKEGSFLASQILLLCQQHSMLNVVLYFSRWTRTLNVF

SYLAFPYFKLLKQRLSGSLRPKIFINQLKDLQGYKIRVQPDLSPPNSFSYRDRHGECQVGGFLWRIVENF

SKSLKGDTQVLYPTWAKAKVSAAEYMIQFTRNGSSDIGVTTTMITFKHEERYRDYSYPMYDISWCTMLPV

EKPLSVEILFSHVLSPGSALLLILAFILFFLIVPQLIKCLGITFRGRLIGMASRIFALVMLCSSSAQLLS

LLMSPPLHTRIKSFDDLLTSGLKIFGIRSELYFLDGGFRAKYASAFHLTENPNELYDNRNYFNTSWAYTI

TSVKWNVIEAQQRHFAHPVFRYSTDLCFSSETPWGLLIAPESFYREPLQHFTLKINQAgamLITQWMTQSFH

EMVRAgamRMTIKDYSRTNLMKPLRIQDLRKCWVIFAVGLGTSTVVFTIELLLIYTNVFLNSL

>DmelIR94h

MLSNISFSSAPELVDLYGLVLKFLVSSETTLFYFNPTGQKCSWETLPRTILSNHPQIIWFREETYPGLYK

RHSSNLFVMACLSSTSYDGQLQLLAESLTRYRSVRVLIEVQDKEGSFLASQILLLCQQHSMLNVVLYFSR

WTRTLNVFSYLAFPYFKLLKQRLSGSLRPKIFINQLKDLQGYKIRVQPDLSPPNSFSYRDRHGECQVGGF

LWRIVENFSKSLKGDTQVLYPTWAKAKVSAAEYMIQFTRNGSSDIGVTTTMITFKHEERYRDYSYPMYDI

SWCTMLPVEKPLSVEILFSHVLSPGSALLLILAFILFFLIVPQLIKCLGITFRGRLIGMASRIFALVMLC

SSSAQLLSLLMSPPLHTRIKSFDDLLTSGLKIFGIRSELYFLDGGFRAKYASAFHLTENPNELYDNRNYF

NTSWAYTITSVKWNVIEAQQRHFAHPVFRYSTDLCFSSETPWGLLIAPESFYREPLQHFTLKINQAgamLIT

QWMTQSFHEMVRAgamRMTIKDYSRTNLMKPLRIQDLRKCWVIFAVGLGTSTVVFTIELLLIYTNVFLNSL

>DmelGluRIIA

MRLCPVVIYAFIIIIGFLEGIIALGGDDRNEITVGAIFYENEKEIELSFDQAFREVNNMKFSELRFVTIK

RYMPTNDSFLLQQITCELISNGVAAIFGPSSKAASDIVAQIANATGIPHIEYDLKLEATRQEQLNHQMSI

NVAPSLSVLSRAYFEIIKSNYEWRTFTLIYETPEGLARLQDLMNIQALNSDYVKLRNLADYADDYRILWK

ETDETFHEQRIILDCEPKTLKELLKVSIDFKLQGPFRNWFLTHLDTHNSGLRDIYNEDFKANITSVRLKV

VDANPFERKKTRLTKVDQILGNQTMLPILIYDAVVLFASSARNVIAAMQPFHPPNRHCGSSSPWMLGAFI

VNEMKTISEDDVEPHFKTENMKLDEYGQRIHFNLEIYKPTVNEPMMVWTPDNGIKKRLLNLELESAgamTTQ

DFSEQRKVYTVVTHYEEPYFMMKEDHENFRGREKYEGYAVDLISKLSELMEFDYEFMIVNGNGKYNPETK

QWDGIIRKLIDHHAQIGVCDLTITQMRRSVVDFTVPFMQLGISILHYKSPPEPKNQFAFLEPFAVEVWIY

MIFAQLIMTLAFVFIARLSYREWLPPNPAIQDPDELENIWNVNNSTWLMVGSIMQQGCDILPRGPHMRIL

TGMWWFFALMMLSTYTANLAAFLTSNKWQSSIKSLQDLIEQDKVHFGSMRGGSTSLFFSESNDTDYQRAW

NQMKDFNPSAFTSTNKEGVARVRKEKGGYAFLMETTSLTYNIERNCDLTQIGEQIGEKHYGLAVPLGSDY

RTNLSVSILQLSERGELQKMKNKWWKNHNVTCDSYHEVDGDELSIIELGGVFLVLAgamGVLIGVILGIFEF

LWNVQNVAVEERVTPWQAFKAELIFALKFWVRKKPMRISSSSDKSSSRRSSGSRRSSKEKSRSKTVS

>DmelCG3822

MRSSGVLVLPLLLLQLILNCRKAQSLPDIIKIGGLFHPADDHQELAFRQAVDRINADRSILPRSKLVAQI

ERISPFDSFHAgamKRVCGLLNIGVAAIFGPQSSHTASHVQSICDNMEIPHLENRWDYRLRRESCLVNLYPH

PNTLSKAYVDIVRHWGWKTFTIIYENNDGIVRLQELLKAHGMTPFPITVRQLSDSGDYRPLLKQIKNSAE

AHIVLDCSTERIHEVLKQAQQIGMMSDYHSYLVTSLDLHTVNLDEFRYGGTNITGFRLINEKIVSDVVRQ

WSIDEKGLLRSANLTTVRSETALMYDAVHLFAKALHDLDTSQQIDIHPISCDGQSTWQHGFSLINYMKIV

EMKGLTNVIKFDHQGFRTDFMLDIVELTPAgamIRKIGTWNSTLPDGINFTRTFSQKQQEIEANLKNKTLVV

TTILSNPYCMRKESAIPLSGNDQFEGYAVDLIHEISKSLGFNYKIQLVPDGSYGSLNKLTGEWNGMIREL

LEQRADLAIADLTITFEREQAVDFTTPFMNLGVSILYRKPIKQPPNLFSFLSPLSLDVWIYMATAYLGVS

VLLFILAKFTPYEWPAYTDAHGEKVESQFTLLNCMWFAIGSLMQQGCDFLPKALSTRMVAgamIWWFFTLIM

ISSYTANLAAFLTVERMDSPIESAEDLAKQTRIKYGALKGGSTAAFFRDSKISTYQRMWSFMESARPSVF

TASNGEGVERVAKGKGSYAFLMESTSIEYVTERNCELTQVGGMLDTKSYGIATPPNSPYRTAINSVILKL

QEEGKLHILKTKWWKEKRGGGKCRVETSKSSSAANELGLANVGGVFVVLMGGMGVACVIAVCEFVWKSRK

VAVEERLSAILNE

>DmelCG5621

MWFVKMISTEASFPLGFILTSLLLAFPGCRGERTNVGLVYENTDPDLEKIFHLAISKANEENEDLQLHGV

SVSIEPGNSFETSKKLCKMLRQNLVAVFGPTSNLAARHAMSICDAKELPFLDTRWDFGAQLPTINLHPHP

ATLGVALRDmelVVALGWESFTIIYESGEYLPTVRELLQMYGTAgamPTVTVRRYELDLNGNYRNVLRRIRNAD

DFSFVVVGSMATLPEFFKQAQQVGLVTSDYRYIIGNLDWHTMDLEPYQHAgamTNITGLRLVSPDSEQVQEV

AKALYESEEPFQNVSCPLTNSMALVYDGVQLLAETYKHVNFRPVALSCNDDSAWDKGYTLVNYMKSLTLN

GLTGPIRFDYEGLRTDFKLEVIELAVSGMQKIGQWSGEDGFQENRPAPAHSLEPDmelRSLVNKSFVVITAI

SEPYGMLKETSEKLEGNDQFEGFGIELIDELSKKLGFSYTWRLQEDNKYGGIDPKTGEWNGMLREIIDSR

ADmelGITDLTMTSERESGVDFTIPFMSLGIGILFRKPMKEPPKLFSFMSPFSGEVWLWLGLAYMGVSISMF

VLGRLSPAEWDNPYPCIEEPTELENQFSFANCLWFSIGALLQQGSELAPKAYSTRAVAASWWFFTLILVS

SYTANLAAFLTVESLVTPINDADDLSKNKGGVNYGAKIGGATFNFFKESNYPTYQRMYEFMRDNPQYMTN

TNQEGVDRVENSNYAFLMESTTIEYITERRCTLTQVGALLDEKGYGIAMRKNWPYRDTLSQAVLEMQEQG

LLTKMKTKWWQEKRGGGACSVSDQPNLNLWQLVHVWVCAgamRGRGLWRRGPGD

>DmelCG9935

MLIASGFLLFQFLSYGLGVPPLVRIGAIFSNQPGMYNSELAFRYAIHRLNMDKSLLPETTVDYYVEYVNR

FDSFETVQKVCKLIRVGVQAVFSPTDSVLATHINSICDALDIPNIGRSAHDFSINVYPSKQLVNYAFNDV

IQYLNWTRFGILHEKENGIINLHQLSRSFHGEVHMRQVSRDSYVSALNEFKGKEIHNIIIDTNSNGISIL

LKNILQQQMNEYKYHYLFTSFDLETYDLEDFKYNFVNITSFRLVDTADVGVKQILKDIGLYSHHIFKKPY

LNLHIKKSTILESEPALMFDSVYVFAIGLQTLEQSHSLTLLNISCEEENSWDGGLSLINYLNAVEWKGLT

GPIQFKDGQRVQFKLDLIKLKQHSIVKVGEWTPHGHLNITEPSMFFDAgamSMNVTLVVITILETPYVMMHY

GKNFTGNERFYGFCVDILETISREVGFDYILDLVPDRKYGAKDPETGEWNGMVAQLMKYKADLAVGSMTI

TYARESVIDFTKPFMNLGISILFKVPTSEPTRLFSFMNPLAIEIWIYVLIAYFLVSLCIYIVGKLSPIEW

KCINACDLENISIGNQFSLTDSFWFTIGTFMQQSPDIYPRAMSTRIISSTWGFFSLIIVASYTANLAAFL

TTERMINPIENAEDLASQTEISYGTLDSGSTMTFFRVCN

>DmelCG11155

MVRKKREIVIKENIQGRSYLKKICCSYIILSILVISNALPPVIRVGAIFTEDERESSIESAFKYAIYRIN

KEKTLLPNTQLVYDIEYVPRDDSFRTTKKVCSQLEAgamVQAIFGPTDALLASHVQSICEAYDIPHIEGRID

LEYNSKEFSINLYPSHTLLTLAYRDIMVYLNWTKVAIIYEEDYGLFNLMHSSTETKAEMYIRQASPDSYR

QVLRAIRQKEIYKIIVDTNPSHIKSFFRSILQLQMNDHRYHYMFTTFDLETYDLEDFRYNSVNITAFRLV

DVDSKRYLEVINQMQKLQHNGLDTINGSPYIQTESALMFDSVYAFANGLHFLNLDNHQNFYIKNLSCTSD

QTWNDGISLYNQINAAITDGLTGTVQFVEGRRNIFKLDILKLKQEKIQKVGYWHPDDGVNISDPTAFYDS

NIANITLVVMTREERPYVMVKEDKNLTGNLRFEGFCIDLLKAIATQVGFQYKIELVPDNMYGVYIPETNS

WNGIVQELMERRADLAVASMTINYARESVIDFTKPFMNLGIGILFKVPTSQPTRLFSFMNPLAIEIWLYV

LAAYILVSFALFVMARFSPYEWKNPHPCYKETDIVENQFSISNSFWFITGTFLRQGSGLNPKATSTRIVG

GCWFFFCLIIISSYTANLAAFLTVERMISPIESASDLAEQTEISYGTLEGGSTMTFFRDSKIGIYQKMWR

YMENRKTAVFVKTYEDGIKRVMEGSYAFLMESTMLDYAVQRDCNLTQIGGLLDSKGYGIATPKGSPWRDK

ISLAILELQEKGIIQILYDKWWKNTGDVCNRDDKSKESKANALGVENIGGVFVVLLCGLALAVVVAIFEF

CWNSRKNLNTENQSLCSEMAEELRFAMHCHGSKSRHRPRKRSCLNCSSVPTYVPSNVSTSNVGVYYNYFN

>Dmelclumsy

MEKMVCNLISQGVIAIFGPSTGSSSDIIASICDTLDIPHIVYDWIPNESIPDREHSTMTLNVHPDNLLLS

QGLAEIVQSFAWRSFTVVYETDKELQQLQDILQVGEPISNPTTVKQLGPGDDHRPFLKEIKLSTDNCLIL

HCAPDNLLKILQQANELKMLGEYQSVFIPLLDTHSIDFGELSGVEANITTVRLMDPSDFHVKNVVHDWEE

REKREGRYFKVDPNRVKSQMILLNDAVWLFSKGLTELGIFEELTAPDLECRRKKPWPFGKRIIEFIKARS

EETSTGRIDFNENGQRSFFTLRFMELNSDGFLDLATWDPVNGLDVLNDDEESEKRVGQKLSNKTFIVSSR

LGAPFLTLREPQEGEILTGNSRYEGYSIDLINEIAKMLNFKFEFRMSPDGKYGALNKVTQTWDGIVRQLI

DGNADLGICDLTMTSSRRQAVDFTPPFMTLGISILFSKPPTPPTDLFSFLSPFSLDVWIYMGSAYLFISL

LLFALARMAPDDWENPHPCKEPEEVENIWSIMNTTWLSIGSLMGQGCDILPKAASTRLVTGMWWFFALMM

LNSYTANLAAFLTNSRQANSINSAEDLAAQSKIKYGAMAgamGSTMGFFRDSNFSTYQKMWTAMESASPSVF

TKTNDEGVERVQKGKNLYAFLMESTTLEYNVERKCDLVQIGGWLDYKSYGIAMPFTPLLARLHKSNGLMA

LCPF

>DmelGluR-1

MHSRLKFLAYLHFICASSIFWPEFSSAQQQQQTVSLTEKIPLGAIFEQGTDDVQSAFKYAMLNHNLNVSS

RRFELQAYVDVINTADAFKLSRLICNQFSRGVYSMLGAVSPDSFDTLHSYSNTFQMPFVTPWFPEKVLAP

SSGLLDFAISMRPDYHQAIIDTIQYYGWQSIIYLYDSHDGLLRLQQIYQELKPGNETFRVQMVKRIANVT

MAIEFLHTLEDLGRFSKKRIVLDCPAEMAKEIIVQHVRDIKLGRRTYHYLLSGLVMDNHWPSDVVEFGAI

NITGFRIVDSNRRAVRDFHDSRKRLEPSGQSQSQNAgamGPNSLPAISAQAALMYDAVFVLVEAFNRILRKK

PDQFRSNHLQRRSHGGSSSSSATGTNESSALLDCNTSKGWVTPWEQGEKISRVLRKVEIDGLSGEIRFDE

DGRRINYTLHVVEMSVNSTLQQVAEWRDDAgamLLPLHSHNYASSSRSASASTGDYDRNHTYIVSSLLEEPY

LSLKQYTYGESLVGNDRFEGYCKDLADmelLAAQLGIKYEIRLVQDGNYGAENQYAPGGWDGMVGELIRKEA

DIAISAMTITAERERVIDFSKPFMTLGISIMIKKPVKQTPGVFSFLNPLSQEIWISVILSYVGVSFVLYF

VTRFPPYEWRIVRRPQADSTAQQPPGIIGGATLSEPQAHVPPVPPNEFTMLNSFWYSLAAFMQQGCDITP

PSIAgamRIAAAVWWFFTIILISSYTANLAAFLTVERMVAPIKTPEDLTMQTDVNYGTLLYGSTWEFFRRSQ

IGLHNKMWEYMNANQHHSVHTYDEGIRRVRQSKGKYALLVESPKNEYVNARPPCDTMKVGRNIDTKGFGV

ATPIGSPLRKRLNEAVLTLKENGELLRIRNKWWFDKTECNLDQETSTPNELSLSNVAgamIYYILIGGLLLA

VIVAIMEFFCRNKTPQLKSPGSNGSAgamGVPGMLASSTYQRDSLSDAIMHSQAKLAMQASSEYDERLVGVE

LASNVRYQYSM

>DmelGluRIIB

MHGLQFLVLLALAIASGANEDTLVIKIGAIFFDTEMKLADAFSAALEEVNAINPALKLDAIKRYVTVDDS

IVLQDISCDLIGSGVAAIFGPSSKTNSDIVEVLCNMTGIPHLQFDWHPQQSNRERMNHQLTVNVAPMELF

LSAAFSDILASKTFDWKSFTIAYERSSHLIRLQHILAWKQLHKAgamIKMQEFERGDDYRILWKRINNAREK

FVLLDCPSDILVDVINASIGYNMTGSFNHLFLTNLDTHLSGIDGFYSRDFTVAVAAVRIRTYVPPPVHDE

IDVFDNSVDTRFSSLGSQLVYDSIVLFYNALLEISQRPGFYIPNFSCGRGFWQPGPRLVEQMKQITPKMV

KPPFKTQRLQINADGQREDFNLEVYNPIIDRVTHIWNKEFQLVDFEKLRENSTQALKQKRLQNKEDFSQK

PIRYTVATRVGKPYFSWREEPEGVHYEGNERFEGYAVDLIYMLAQECKFDFNFEPVRDNKYGSYDANTDE

WDGIIRQLIDNNAQIGICDLTITQARRSVVDFTVPFMQLGISILSYKEPPPKADIYAFLNPYNAEVWLFV

MIAMMITAFALIFTGRIDQYEWDQPVENVNREMERQNIWHLSNALWLVLGSMLNQGCDLLPRGLPMRLLT

AFWWIFALLISQTYIAKLAAFITSSKIAgamDIGSLHDLVDQNKVQFGTIRGGATSVYFSESNDTDNRMAWN

KMLSFKPDAFTKNNEEGVDRVKLSKGTYAFLMETTNLQYYVQRNCELTQIGESFGEKHYGIAVPLNADFR

SNLSVGILRLSERGELFKLRNKWFNSNESTCDSNVPTIDDGQFDmelDSVGGLFVVLIVGVVVGLVIGVAEF

LWHVQRISVKEKIPPMLALKAEFYFVIRFWLTRKPLHTYRQSRDSTSTGYSSLEQITSASSAKKKKKTRR

IEK

>AgosIR1-C

MENLLSAIRRSNLMSRNVVYVFLWLRSPVSRTFKTDILEAMRVCVITSPRPGFYQIYYSQASARPGYGSTLKMVNWWSAMDGLVRFPLLPPPKRVYKNFEGRYFNVPVLHKPPWTFVEYLNDSFRVEGGRDDKLINLLADKLHFHYQIYYSQASARPGYGSTLKMVNWWSAMDGLVRFPLLPPPKRVYKNFEGRYFNVPVLHKPPWTFVEYLNDSFRVEGGRDDKLINLLADKLHFQFKYIDPPDRTQGSGLDQGSSMQGVLGLIWQREADWFVGDLSITYERNLVVDFSFLTLVDNEAFLTHAPGRLNEAFSLIRPFHWSVWPLLLITVIFSGPILYILVDTTDGHPQGKSMLYWKCVWWSVTVFLQQGNVLH-

>AgosIR2-N

PEITATKFGHTIDSRVFIVTIGSKWQIQEFFKSQASQNIMNLLIASAgosPALQEKNKNEVADIKLYTHEMYIDGLGSSVQIILTTWKLNRFTRPEVNLYPVKLVNGFWGHRFIVSAIEKPPLVFRSIDKILTQEQSILWDGIEIRLIQLLAgosILNFTLEIHDATLSKSRDESDDRIIGDLVTSKAELGISGLYMTNARYSMVDFSPVIMQDCGTFMSLGSFALSKYRAIFGPFHWSIWVMVVITYMAAIFPIAFTNNRNVKTLCKSPKQLESMCCYMFGTYTNLFTFKDVKSWTNTKMGSTRLFIGTYWIFTIIITTAYTSSIIAFITLPAQPVIVDSSYQLVDQRYRVMTLDKGGWQYYLNITNDTMSKRLISNIKLMNNLEDAIDYIVRTRFILDYAFLGSKISLTYLYQNNYIQKYKNKKIFLHVASECYVPFNIGVAYKKHFLFRNIFNNFILRAQQSGLITKIIKDIEWEIIQKSGVRNPNLIIAPEDRQLALDDVQGMFVLLGGGILLATFTLIIEYVKRKREKRKITQIKIKNHQKKKKRSKSLTVAENTIAESIRPLTTF-

>AgosIR14-F

MVSIFIVSYCEDIWDLYYKAEFEGLNGLLYISITDTNCPRLPTDEAITIPLTTHDSELSQMILDLRMSNAFSWRSAILMHDNSIGDSIVQHIVTSLTKHYPSNILSPSVAIFEIYTQGSEWKRRKLFMEELEHFLKMSEINSNFICIVSIHYVPLILDVAKSLNLMTAENSWLIIIPDIESSRSNVSSFTSLLSEGENISFIYNSTKTDSKCVFRLNESGVCETCPMWHIDSGVTWGQEYFGHGCYILPVGYWNTKTGLKLTEPLFLAFGECYNVIVISNSDQTNATRTLFTQNNILDEHDAVVSKPIWDKMIDLIRSEKTWLCIAIVVGLMGPILNVFHVLSPYYEYHNITRKGGLNSPLNCFWYVYGALLQQGGAHLPDADSGRLVVGTWWLFVLVIVTTYSGNLVAYLTFPQMDTMVSNVADLMARKPQGYSWGIPKSSNLHSLLTSLPDDTMVKELIKNAEHHEGLSRPVIERVRSGKHAFIHRRTNLMYIMKNDFFKTNRCDFAIGNEDFAEEKLAMMLAKESPYLSRINKEIEKMHKVGLINKWLVDTLPKKDQCWTNTQLEVTNHKVNLDDmelQGSFIVLLLGVLSSLVSFLFEYMLDKYINRRQIVITPFIN-

>AgosIR9-F

MKRSAgosAVVAVVALLQAAAAVGGPTTRRRLRDLALDDDAHRIRLSRLAgosSVNKIIDEYLGTRSVVMFADEVDVSELGQRLLVDmelGHPRLLVQSSAATDARLANGLVVYLEPEARAQRRDGRSADYDAVLGRLPSSDHSRHMVLWEGSQRPGDRVDLHRIQMLFEAFWHHQLVDVAVLVPVYTGSIRVYSFNPYTGSRCNGAgosPPIMVNVWSSSTDAFVRPDRVFGLDNKLKDLHKCPLKCLGIHRPPVSTVVQTEKGFKLSGSGLRIINFIQEYMNFTGIVTMAVGHSGVHFVSETALSDNDSSPIGVKVKHKKVDLAFGRFTRIFDSESDVEFIKEDQMECFTWGLPSGIGHDPTLWINYVAEFSLVTWILIIMSIILAFGVVVTLSQLTSMLKQPAATISWSPLFILFYTYGTFIGAPIKVTPKSCALQVFLSNWLLYSLVVTSAYQAYLGSLITIPQTIPEINDQHTLLKTDLNLVGRQDmelYYLINSSAgosSSNDFKELVDRYQILPPEDFSHFIQRILLKRDTAVLASKRELIFYAQRYKTIFNDSRHLHVLPTCTIESYSSTFMLRRGSPFRHRISTIMSRLSETGIMQQWDRENLREEQVHNIVFTEDTILSMSQSFGAFVVLFFGLFLGFCAFIGEIIVYLINKFCIRHTKQIMFIN-

>AgosIR13-F

MYYGFRRFDEHGYTRAWKKVGTIGPSSTGGQLVKLDSVVWAgosGKMVPASDLGRRAVYRVVTAIGPPFVMHAPLQQDRQCLRGIQCYQMTTTNKDNITMILKDVKLDSKKNATLPNTYCCFGLSIDLLEKMSKDLEFDFHLYLVADGTFGSQKIHWNGVVGELVSGTAHMAFCPLSVTSTRSKWVDFSTPYFYSGVSMMVAPKRKTNVPLLAFLLPLSPSLWIAIFVSLHVTTVAVALYEWFSPFGLNPSGRQRSKNFGMPSALWAMWGLLCGALVNFKAPKSWPNKFLINMLLMKVGSAKSSSAEVYLKDKNPPLWQHVQKYSVPDTASGMRMLRNGSLDIFIGDKPILDYYSGTDHDCKLQTHGDPLYDDVYAVGMTKNFILKEKVSAAVSTYINNGFMDILQNKWFSDLPCVNRQLETSDIGQPTPLGVDAFLGVFLMLGFGILGGAIILCLEHAFYRYALPILRQKPPDSMWRNPNIMFVSQKLHRFINNVELISPQHTAKELVHTLRQGQIASLFQKSVRRVSLPFMHPQHAQKEFERRKRKGQFFEVIEEIRRIQREERDNKKQNVRLTLSNSPTAgosQSPKRTTSKFLSPARLSLGRRYSKQRSRSSGNLSIRRYSTDVASYSETVGRRLSQGASNSPPDFNTRKQLIRRSSGNPSPADSSRMSIFSASELIGAKLSNNNLYVENDLPRSPNLLSPGVFFRSSFSSDTSSSRPDLGIQSRKSSYSQGPPRVVINGEQSTRRKSDDEPPATLPRIMEVKRARRSSDGHRPTKNRKTVEVFRWLRQAPKTELEALSKMPEDEIKHCIIQALKDKDPT-

>AgosIR8-F

MRTYAgosVLTQNENDTNFIQLKSILTNYDNISIEPILNDQPNNITDKFCNVASNNTLAIIDLLNPSCTTCWKISNANAMAYFRTDFSYIQPAIQLIESYVTWLNITKEITFVFTNQEDADQAVTYLTSGKSSLRAIVLNKLTSNEIDQLKNTKIGIRHVALIGNNLDSYVKTINQEKLIKLDESWIIVTNDTSKYKLEAAVTLMKFTSWENGHTNKTTRARTLFNFIFYFLGRVSRDRLKLNCNMNSDmelLVLGKRKETEDILNSYEYKNEFHYDIDTKLMTYNEQATILKFSPDGVPNQLGTWTINGGLEMKYDALTVVSGRRFFRVGTAKSIPWTFMEDEWKGYCIDLIEKLSIEMNFKYELVIKDQFGELDPVTNKWNGLIGGLVDGELDIVIAALTMTSEREEVIDFIAPYFEQTGISIVIRKPSRKTSLFKFMTVLKPEVWLSIVAALALTAFMIWILDKYSPYSAQNNKPKYEQFRHFTLVESFWFALTSFTPQGGGETPKAISGRVLVAAYWVFVVLMLATFTANLAAFLTVERMQTPVQSLQQLARQSRINYSVIDGSDAHQFFRNMKMAEDILYKQCWVLEVSEKLKMPSSVWKEIALNQTNNRKDFRVWDYPIKEEYGQILAAIERTGTVPNRTVGYQMVTVWKEIALNQTNNRKDFRVWDYPIKEEYGQILAAIERTKSDIEYEVYNNCNLTEVGEIFAERPYSIAVQQGSLIQEEISRKILDLQKDRFFELLNAKYWNASKTSTCPNSDDSEGITLESLGGVFIATLVGLLIALITLAFEVVYFKHKRAKIAEVSVVNNTIHKDKLLYGHELFMTLGRNSNSDDQTRWANKIKLDSTTGRLNNALFFRRNKFQN-

>AgosIR7-F

MNCSYGEIKWNSEFENLAVDITYKWKDTATCLNLILDHYHDGILDKGFYTAIAgosIPLFKTLVDDSEDLMSPNFQTWQILNNVRKQGCNMNIIFILNADQTMRLLKFSDKKYRLKEMYELSTVPYPAPIKGTLVTLRLDIWNKRNFQKKTDLYIDKVSDLQGNLLKVVTFNYIPSAIKNPLMNENDENSGYKKGLEIEVLRTLGSAMNFIPVIYEPLNWRTEQWGKKQINGTISGLLGEVWSARADLALGNLHYTPYHLNILDLSIPYNTECLTFLTFESKTDNSWKTLILPFKLNMWVGVLITLLVGGFLFYAFATAHKHIEDNENLIKMIQCDIQKTKILENKPELLTENKTIIKNIDIIKHTVKYPKIIKEQKCINEQTKNNQFNNTDVTGLYLFENIENSILYTYGMLVAVSLPKVPSGWAIRILTGWWWIYCLLVVVAYKASMTAILANPDTRVTIDTLDALAESNINCGGWGEQSKEFFMTSLDKTGQKVGQKFQEVYEVDKAIDLVSKGQFAYYDNIHFLRYVKVMQNTKTYEQNVQLINGTRNDTSNGDFTLHIMSTCIINMPISLGLQKNSPLKPAVDRFLRRVIEAgosLVKKWLNDVMSDTVILEEPQQIEEVKALMDLKKLYGALVVLVAgosYILSILVLLIEIGYWYGVVKKDPLYDEYSLNCYYSQK-

>AgosIR3-C

MFCIQFSRGVYSMLGSVNPDSFDTLHSYSNTFQMPFVTPWFPEQVLSPSSGMLDYAVSLRPDYHRAILDTVRYYGWTNIIYMYDSHDGLLRLQQLYEALDLGPNSLKVDmelVKRIQNVSDALNFIHQVERVNRWGNKRIVLDCPTLMAKQIVVSHVKDIQLGKRTYHYLLSGLIMDDHWETEVIEYGAINITGFRLLDmelNQWSVKHFLSEWKNLDPNTSPGAgosKDTISAQAALMHDAVLVLVETFDKLLWKKPDmelFKMNAKRTLSNGNSSMSGISSSQILGLDCNNGRTSGNQWEHGEKISRFLRKVVMEGLTGHIMFNDDGKRYNYTLHVVQMTIDSTITKIAEWSDTDGFKTVVSKPERVHTSGHSHKGNSTLIVATVMEEPFIMFKKPKYGESLAgosNDRFEGYCKDLTTLLADKIGVK

>AgosIR25-F

MYKQWRNLDGEQQKYLIQISPPADLIPEIVRSIVVAQNITNAgosIMFDDTFVMDHKYKSLLQNIPTRHIIAAIDDTTSIKLHLTRFRDVDIVNFFVLGKLSIIKSVLDHANSNKLFGRKYAWHVITQDKGSLKCGCSNATILFVKPEPDAgosSREKLSNLRTTYGLTSTPELKAAFYFDFYYRSLLAIRSMMNSGEWPTNVTYTTCDEYNEENPLPRRNVDLRRYLKDmelTEPPSYAPFLIDTNGHSYEEFTMRLEKVTVLNSQSVSAENVGSWKASLNSPIVVKDAANMTHFSAVTVYRVVTVLQNPFMIQVDDEDGKGVKFKGYCIDLIEEIRKLIGFEYEIYIAPDNNFGNMDENGQWNGMVKELVEKRADIALGSLSVMAERENVVDFTVPYYDLVGITILMKKPQTPTSLFKFLTVLENDVWMCILGAYFFTSFLMWIFDRWSPYSYQNNRNKYKDDEEKREFNLKECLWFCMTSLTPQGGGEAPKNLSGRLVAATWWLFGFIIIASYTANLAAFLTVSRLDTPVESLDDLSKQYKIQYAPLNGSSAMTYFQRMADIETRFYELSINIFLNINRIWKDmelSLNDSLSEVERAKLAVWDYPVSDKYTKMWQAMKEAKLPNTLEEAIERVQSSKSSSEGFAYLGDATNDIRYQVMIDCHLQMVGDEFSRKPYAIAVQQGSPLKDQFNNAILLLLNKRKLEKLKETWWNLNPERIQCEKQDNQSDGISGVFIVIFVGIGLACFTLAFEYWWYKYKKSSKVANTMNPKQMAMNKGGEFTYPVVPTFNTTSGMRSRSIIQGFRRSVGQSSPKQK-

>AgosIR6-F

MDYINVVLLFLVTYTISTSQLDSNFDAQYKLMDQFLTIKNLDTAHLYTCWSQSDRIKAMKKFNEKKIAVTYRNKEDFYGNFNLNDLFTITVYSPLLGIIVDWSCNDDPLFKFSASWLWSASYHWLMMMSEKSNNIQSFLTSKNVNLTISSEILLAYPIMMDGVITNWHIFDIYRTAFEPRGKLMIDVVNKTLGMWGNRLWKFDKRSNLENLTLNVVTVVRTVMEPTKLSNRLIVLLMFVFSLVTYQFYSSSIVSGLLRPTVMNIDSVQKLEESGLDVGVEDFNVITKVIEVYGEMNPFLKKIIDSKIKPKSEYLLAKDGVKKIKKGHYAFFTDPATSYWLINDIFTEKEKCDLSELALHRPETTGYLVQKNSPYRKLINYANCILWETGLMQRELSIWHAEKPKCTAgosKTTTEESLVSVEIKDVASLIAFLIIGFIGSFAILVLEIIIHRHKTHTTAILVKNN-

>AgosIR5-F

MHQNYKPALLLNEFHVKLFNPNRYILPHAYLVAANNTTDLVDHLDTINVTDASWQPEVNFIIILEKFTELSQHILEPVFQRLWIKNMFKSVLLIPAIDGNAIEAFYWYPFHKRCGEYHTPTLEDLCTRGDNNNSQWTVFDVFEKIIPNTFYNCTVNIVGFNWAPMTLLSNQTPRTMLHGMDVEVVKLMSRIGKVQLEFHEVENNQRWGVKLENGTWNGGFGKLSGHRGDFLIGGGILTAERKEMFDSAPARQVIRFPIYTPLPRKLPYWQNMLNVFSGNFWLTLFVVFFLTSGLLWLSGIHLPSEKRAFSNCGYCLVISWAILCSVASGQQPTSVSSKMIFLSWVIYMLHISAVYTSMQLIYIYKPKYEPPMRTVNDVKESGLTICSVPTFIPIAHSMDKDNFNLTEYIPCMDNHFEALISGSIKKVNKVEEVVIVYNIGIFMQKGNPYKNILSKAQIIAYETGLHDKWRQDASPSRPIKKKGNIKVKKLSVDELQGAFIILICGLGMSSVIFVFEWLFGSK-

>AgosIR10-F

MRNSVAFWFYLVASSFKTSAEYDGLINALTRVFIQKHVLTVTAITCWPKDANSRLLLALSSADISVNFRPSMIPVHETWYRYGILVDVSCVQVTTTLFQKISEPNKFKGFYPFADTDLDIFAQMHYDLNMILQDQLNFKIDLGIVDSFGWNLGNGSFSGLTGLLQREEIDFGGIGSFIRNDRMNAIDYTVGTFYRQPAALFKQPPLSSVHNICILPFKFEVWMVTLFTFIGFTILIAFLSRTTRRFKKDEKESLNVLDSVTIVHGAICQQGYTMNLNAgosSIRVAIFVLFLTAVFLFTSYSASIVALLQSPSNSIKTINDLVESSMTFSAQVTPYSRVYFDETDDPLLRKLYDKKMKSHEKDmelYTEASTGIARIRTEFHGFLIEVVSAYKLISQLWREEEKCGISEIQLFKLPILALAVVKRSGYKDILKQKEESDFGAAgosSLMRLDRMTAVDFTVGTVSLESNILFKQPMLSSVTNIHIEPFKYEVWQVTLVMLMGFVLILLFLNKFKANRRTSLNVLEIIGLVHGAICQQGSTIVLVLNSIKIVVFVLFSTTFFLFNAYSASIVSLLQSTSSSINNVKDLLHEKSMTMSIQIATYAKPYFNVEKTSAYQIINRKWREEEKCGLYEIQLFKLPVLAIPVVKKSGYKDVFKQKLIQQHEVGIKKRLIQRWTPKKPTCDSAKKNRNYVSVSIKEILPTIILFGYGLLISLTVLVLELAYYYGMHYFMKRFKKIKK-

>AgosIR12-F

MDKFVIFIIFMVFKVANSADYNEMLNVLKIFFVRRHVQTVTAAITCWPFDVNKKLLDDLSTADISVSFNSHTLQYYSTWYRCAFIIDLSCENSTEILQQISNDRLFNTQNDWILFDQNSFANETNSAQFALRTFQLYLGNAYVLPDASVFLFLKTYDGVWEIWSGFRASKSDAIRVFEIGTASLNRMTIREMHDEKRNFRGVTLKSTSVIVEKDSFFGFDKKISSDLDVFAHMHYEMIVTLTNQLNFKTEITIVNDYGWYLGNGSFGGLIGLLQNEAIDFSATGVLIRTDRMSVIDFAVGIVELRTAAMFRQPSLSSVHNILLLPFTTDVWIGFNSTLTLISIRVVIAVLFFTSVFFYTSYSASIAALVQSNSNSIKSIKDIVESSMTFSTQISPYGKRYFEETEDSDLKKLYKTKMVPYGNKAFVKTAEGMERIRTEFHGFMVEIMSAYQTISKEWREEEKCALGEIQLFKLPLLSIALVKKSGHKDIFKQKLIQQMEIGLSKRISNQWIPPKPSCGSSSRTKQYVSVSVKETFLTLAVFGFGICISLASNRRLFSLQNDWVLFNRHGNPNKTDALEIALHSFKTHMNQAYVMPDSRVYLLIEVNRNLWEIWDGFRLSVAKEVQVSRVGVMTPERVEIKLSDRTNFKRVVLSASTVVSPM-

>AgosIR4-F

MALTNTLLLALCANWPPLNITETTLLPPWKWISEHKAKYINSSEYQCDLKNNFQIQYEILRGKRLKIATFPNSKPLSWVTKEINGSLIGHGIAFEIVETLRQRYGFTYDVVVPTRETLLNENGSIIYMLVNGEVDmelAAAFIPVLPGLDDIVKWGIELTQFQYVVLMKRPKESATGSGLLAPFEMEVWFLILMSLIAVGPIIYGIMMLRHKLCGHESGVYPIPTCIWFVYGALMKQGSSLNPDTDSARLIFATWWIFIMILTSFYTANLTAFLTLSKFTLPIKRIEDIASNEYRWISSEGSAVEYIVKVDNDLKPLRQSMFEGNGQFMVINIDEIDTILKYISSGTLLLHDKNWLNFLMLHIERSITDDKERCRYVLTTDPYLTRSMSFVYPKHSILPPLFNPIRMLSYMESGIVRHLQTKDLPEAVICPLNLGSKERQLRNSDLFTTYAVVVCGFSMAAAVFTLELLSRRTGWFATTDLSSRPHDPHSGVVRCEIAVFGKPSKTMYPFSHRHNVMNAASATHFVFHGSSSSSPSRSFVNARSKIASDSSSSSSSPFLWLEGKSSLRRFHY-

>BmorIR8a

AVPWTLPKLDPETGDPLYNEDGQPIYEGYCVDLIQKLSEAMNFDYEIVSPRSGGFGRRLPNGSWDGVVGDLTTGETDIAVAALTMTAEREEVIDFVAPYFEQTGILIAIRKPIRKTSLFKFMTVLRTEVWLSIVAALVLTGFMIWLLEKYSPYSAKNNPGAYPYPCRDFTLKESFWFALTSFTPQGGGEAPKALSGRTLVAAYWLFVVLMLATFTANLAAFLTVERMQTPVSSLEQLARQSRINYTVVEGSSTHQYFINMKFAEDTLYRVWKEITLNATSDQAQYRVWDYPIREQYGHILLAINASGPVADAETGFKQVNDHTDADFAFIHDSAEIKYEVTRNCNLTEVGELFAEQPYAIAVQQGSRLQEDISRALLELQKERFLEQLTSKYWNETLRQSCSDADESEGITLESLGGVFIATLFGLGLAMITLAWEVFYYKRKEKNKVQSTKENVERPPIKSAKLGGKMAVGVARLRKRATKIGKKKNVTIGDSFKPSVSYISVYPKGDYR

>BmorIR21a

MDRRSLYGLLFIFYIISSQEIISYHSESLLKNASRNLLWNKKITSIIKEHNDFAYHYESDLHFGNRIKNVKSKRAVDPVFHGHPKTREELWYERFLNRSSVFDQTPSLIKLIQNITLTYLNECTPVILYDSQIKLKESYLFQNLLRNFPVSFVHGYINEHSQLQEPKLLQPVRECLHFIIFLSDVKVSAKVLGKQSESKVVVVARSSQWAVHEFLSSSFSRGFINLVVIGQSFKEDDDSTIESPYILYTHKLYTDGLGASKPVVLNSWSHGKFSRNVNLFPPKMTGGYAgamHRVVVAAANQPPFVFRRIFYKKIYRIKSDLDGGNPRVVWDGIEIRLLHLLAEKNNFSIEIVEPQELHLGSGDAVAKEIAKGRADIGVAgamMYLTIDRTREMDVTFAHSQDCAVFITLMSTALPRYQAILGPFHWHVWVALTLTYLFGMFPLAFSDKHTLRHLINNSGEIENMFWYVFGTFTNCFTFLGRNSWSKTDKITTRLLIEIGKIFPCYYFRMVLDFTIIITSCYTGSIIAFVTLPMFPETVDTIHQLLAgamFYRVGTLDRGGWERWFLNSSDPNTNKLLKKLELVPNVEAgamIMNTTKAFFWPYAFLGSKAELEYIVQSNFTKTTSKRAVLHISNECFVPFGVTIGFPNNSLYTAKLNNDLRRMVQSGIVDKIVDEVRWEMQRSSNGKLLSAVGGSLKVSAAEEKGLTLEDTQGMFLLLAAgamFLIAATALISEWIGGFSKLCRFRKKKNTLVNSSTKEDSINMPPTDSKDFKTETESVLHFCSRSTSPGSNESLDGQIINVTEESIEIHKQFTSEWDSRRSSSVDLEKEVKEIFERDLRRRGAALXXXXSTASNNAFGDAVK

>BmorIR25a

MCRSSHIFQNSMPLFVVFLQFFIFRLIVSQTTQNINVLLINEENNALAEKSFEIAKEYVRRNPSLGLAIEPVIVVGNRSDAKTFLENVCRKYNDmelLSSKKTPHVVLDFTMTGVGSETIKSFTAALALPTISGSFGQTGDLRQWRSLNANQTKFLLQVMPPADILPESIRAIVTKQDITNAAIIFDELFVMDHKYKSLLQNIPTRHVITPVKSFNKEDIKTQLRSLRELDIVNFFIVGSLRTIKNVLDAADENQYFGRKTAWFAFSLDKGDITCGCKDATIVYMRPTPDAKSRDRLGKIKTTYSMNGEPEITSAFYFDLSLRTFLAVKSLLDSGKWPNNMKYITCDDYDGKNTPNRTLDLKLAFQEVKETPTYAPFYIPGDDPMNGRSYMEFSTDLSAVTVKDGASIGSKALGTWKAgamLNSPLSLTDSDNMSDYSAQLVYRVVTVEQQPFIIRDDNAPKGFKGYCIDLIEEIRQIVKFDYEVTLSPDGNFGTMDENGNWNGIIKELIEKRADIALTSLSVMAERENVVDFTVPYYDLVGITIMMKLPRTPTSLFKFLTVLENDVWLSILAAYFFTSFLMWVFDKWSPYSYQNNREKYKDDEEKREFTLKECLWFCMTSLTPQGGGEAPKNLSGRLLAATWWLFGFIIIASYTANLAAFLTVSRLDTPIESLDDLSKQYKIQYAPLNGSAAMTYFERMAAIEVRFYEIWKEMSLNDSLSDVERAKLAVWDYPVSDKYSKMWQAMKEAgamLPNSIEEAVQRVRDSKSSSEGFAWLGDATDVRYYVLTSCDLQMVGDEFSRKPYAIAVQQGSPLKDQFNNAILQLLNRRRLEKLKENWWNNNPKAMKCEKQDDQSDGISIQNIGGVFIVIFMGIGLACITLGVEYWWYKWRRRPIVGDVTQVEPAKSTRNNIGNFVKGEGFTFRSRNFGLSDLKQKF

>BmorIR40a

MTKLPKDFNVAIKDIAESLPSKEMTVVRGNSTNIRSQDVFELLRLLCQHNIQVVNLDIAAMENKEMYYGYLKKALDVSDERTNLILCEPYECENLLLELRENNLIHRTILYIFFWPYGSVSDRFLNTMVEAMRVAVITNPRESVFRIYYNQATPNRLNHLSLVNWWAFRLYKSPLLPSADKVYKNFRGRVFDVPVLHAPPWHFVKYNNDSSINVTGGRDDKLLKLIANKLNFRYRYYDPPDRSQGSGIIGNGTFKGTLGLIWKRQADFFLGDVTMTWERLQAVEFSFLTLADSGAFLTHAPAKLSETLAIIRPFRWEVWPLVCATLFITGPALWIVIAAPSLWQRKKRDQMGLLNNCCWFTVTLFLRQSSTKEPSSTHKARLVTVLISLGATYVIGDmelYSANLTSLLARPAKEPPIGTLPALEEAMREHGYELVVESHSSSLSILENGTGVYGRLAKLMKRQRVQRVHNVEAgamVRLVLNRRRVAVLGGRETLYYDTERFGSHNFHLSEKLYTRYSAIAFQIGSPYLETINNVVMTLFEAgamILGKMTTDEYKNLPEQSRRSEPVTESENLSTEKTGETAAVTQIQNETSKGLEPVSLTMLRGAFCLLGIGHLLAgamVTLLIEIQLYRRARKRALPPQTRNPTNTFKAKAKKCILRGWRRIKAAAILAIDRALAPDRGID

>BmorIR41a

IEILLQIIINKYLSESYCLVVISETPLSVKLPMSFTYLDPKKEHFSVETLLKLSEEGCSDYIIRMEDPRQFMNALEEIRPMSMVRRSDKKLVILPVTDDENSMEPILNLLTMKESSYYAHILLILPTQTERFECLAFNLITHRFVGSDSESKLPIILDRWYSCTNHFENNVYLFPNDLKNLNGKTMKISTFIYKPYVLLDVDTAVAPLGRDGIEIRMIDEFCRWINCTVQIIREDVDLWGEIYENETGIGVIGSVVEGRSDFGIAALYSWYEEWKAMDFSVSVVRSAVICLVPAPRVLESWELPFLPFGKSIWIAVVITFVYASIGLTIAQGCSSNKALLIVFGTIISQSQYIVSDSWRIRSVIGWLLVSSLILVSAYGAgamLASTFTVPQYEPSIDTVQDLLNSRMEWGANHEAWTFSLALSSEPVAKKLIKQFKIYSFEELQRRSFLRKMAFSLEELPAgamTFAIGEYLSKEAVQDmelQLMLEFFYFDHCVAMLHKNSPYTEKLSELIGRLHQSGLLLAWESQVSLKYLDYKIQLEIRLSRARSDVGDLKPLNFNHVEGIFLIFITGTILSTLFFALEIFIGKQARKK

>BmorIR64a

MNILGLNIISFLCSLDISSVIEVFKCKHVRDVIVFHCFKENQLILPQRMFHFNNFRTVFVFISNNISWELPNSYPKIGVLINTSCDGWEKFQEFQNSHTWVYYTDNLTSTITALSTFPIEINSDVTVVYKENSAYQVFDTYNTGRKNNGVFNVNYIGHINPGLQTNLKFTTRNLNGVTLKSTVVILKKVQYESFEEYLRKTEQTGLDSVHKHKFFQLLQYISEMYNITYDLIRTNTWGYAHDGRIDGMVGSLQRHEADVGGSPIFFKTDRAYVVDYVAETWPSKQSFIFRHPKHPTGVHTVYSRPLSNSVWYCVIAFLFVTASTVFFMLKFNIDEIERAETSQSLAFLFAWSAICQQGMSLRRNSLALKVVVFVTFVCSITLYQYYNATVVSTLLKESPITIRTLKDLLQSDLKVGVEDVAYVKDYFAHTKDPIAITMYEKKIVTGNNRNFFDPEYGMSLVKKGGYAFHVDTVYSYGIMKKTFTEREICEIHDVTMYPPQKMGAVLKKNSPYRNYFAIGIRRLWETGLMQRMKHIWDEPKPPCVRTQDSSIFSVSILEFSTPLFIVVFGVIASVVVLLCETLFDTLFNMR

>BmorIR68a

TIMPLTLFSRSPRSGKTRAVSKASPILEDIYEQKDLEFVLVDLLNHAgamRYHDFTCVAVICDAIYYNVFDGAFFKRIDTVPFVMIVVEEYDDLLSPNFDILEALREARRDGCNMYIILLANGLQAARLLKFGDRHRVLDTRAKYIILHDYRLFHSDLHYLWKRIVNVIFLKHHRKIGSVAKSQAWFDLSTVPFPNPIKGVFVPRRVDLWKSGKFHYNTVPFDDKTSNLNDEVLHVVYLDHVPSVVVVNSNETGQIGGVEIEIINTLSEKMNFRPKLYQPMNVELHKWGQKQPNGSFSGLLGEMVNGRADLALGNLQYTPYHLELIDLSIPYTSQCWTFLTPEALTDNSWKTLLLPFKLYMWIAVLLVLXITGTIFYGLARYQTYLHGLKRQEEMKKPVYSKPVGLYLFGEIINSILYTYGMLLVVSLPKLPTGWSIRFLTGWYWLYCILLVVSYRASMTAILANPAPRVTIDTLVELAASKLTCGGWGIETKNFFQDSLDEIGQKISDRFEISNDPNIAADKVAQGTFAYYDNKNFLKYITVRRQNGFIMETIDNTTNFTSISTKSNNERNLHIMSDCVVNIPISIGFHKNSPLKPLTDIYITRIVEVGLVEKWLNDAMYTIKTLETNEEEIKALMNLKKLYGAFIALAIGYFLSVMCLIGELAHWNCVVKKDPNYDKYALHKYYEKINKK

>BmorIR75d

VNIQNNFANAPELPAYDFRREGVVLDLNCPNSKLILEKASKNRAFIHRYTWLLIHNSTYKLETIQKILSDAAVLPDADVTWCAADDILDIHRLNEHQPYVVMDLGLSVNSTIEDLDAVWSTIPTAATRRRHLNNLTINAVVIVSQPQYFKGWSDLSNRQIDTFPKLTYPMLMLCAEDLRFRFNLKQVDEYGVELNGSFTGTVGLLQRGRAELGVASMFMRSDRWRVLHFSSATVALLNAFMLRAPAQSSVSNIFLLPLSRGVWCCAAALLCGSAVLLAVLSCRLVAADPTLQLLTLPEIFVFSIGTVCQQGFYIMPKLSSIRMIMFLTLLTSLFTFTAYSAKIVAILQTPSAAVRTVADLADSHMDVGIQETTYKKVYYAESTDPSILRLFHRKVAPLGERVYMSVVEGVERMRTGLFAFQVERSSGYEIISKTFTESEKCGLMEIEAFKLPMVAVPLRKHSGYRELFGTRLRWQREVGLMSRVRAIWLAARPRCEGRGVGFRAVRLLDmelLPALQMLAAgamGLVAVVLLILENVYHHYSRTGNVLRRTYRNVFKLVCYKCYTF

>BmorIR75p

MVDVRPHRELFKRRRDVMGRPLTMANVIQDSNNTRYHLPREDALELQYDVIPKICWMTAKLAFQMLNATPRYTFSYRWGYKVNGQWSGMINDLHTSKADLGTNCVVSDVERLSVVTYTDmelLAPFRVRFVFRQPPLPSVANIFYLPFTGRVWAAVAVCAMVYTAAIYWASKWEFNLEKRSASQFDGTVGDAMLLTMSALSQQGCFIEPKRAPGRIMLFVLFTALMALYAAYSANIVVLLQAPSNSITSLAQLAASKVTLAANDVDYNHFVFSLYKDPVRVMIHKRIDPETGNGQFYSLEDGVDmelIRQGFFAFHSIVEPVYRRIEETFLETEKCDLTEVDFLSSFDPFVPVKKDSPYLELLRVVKKKKIRCSFKQIRESGIQSALNRRYQVPKPRCSNKVAAFSSVGIVDLRPVLIMMIYGIISSCLILIMEMLVFKM

>BmorIR75q1

IFKIKNPVAITFGKIGNVEEVTPSNHILFLVDTTCNNSHIVLQEADAHQQFRRSYRWLVLETQGSGYNSKLLEIEPLNILIDSDVLLATKIENVTYVLKKIYKISTQSEWITEDYGNWTAEHGLIMSNIISSDASRRRNIRGHPVTTSIIVTENRTKSELDDLKNLLSDSLAKICFRHTKNLCQFMNASHKIGFASMWGYKTNGTWNGMMGDLAKGTVDFGGTIAFLTSQRLQVVDYLSSPVPINAKFVFREPPLSYQNNLFLLPYKANVWYCTAAFVVLLVIILYINAKWEIKKAEYEQAVTLQPSVSDVTILVISAISQQGSSNELKGTLGRAVLFLLFLTFLFLYISYSANIVALLQSNSKQIRTLQDLLNSNLNIXDGKPLFQTATEPIRKAIYETKVAPKGSKANFMSIEEGVKKLQKSPFAFNMNIGTGYKIIERYFEEHEKCGLQEINYIESSIPWMSCRKNSPFREIYKLGLFKLQEHGITDRENRLLFARKPVCIVRGGNVGSVNMVDVYPVILMFLYGLFLAFLILLVEIVVHRKL

>BmorIR75q2

MSGGFQKNTNINEVIAVRRRDLEGYEIKICYVLTDNDSIHHLSDEVNDHIDTITKVNFPSTNHLLDFLNAERKYVFVNTWGYRINGTWNGLTGFLVNGDVEIGGSPMFFTAERTAVVDFISSPTPTRSKFVFQQPKLSYENNLFLLSFRTAVWYSTLALISLIFTMLLSVTAWEWKKMSQIKTRDIDAgamVLRPSVTDVTMLVFGATCQQGSTVELKGSLGRVVMLILFLTLMFLYTSYSANIVALLQSSSSQIKTLEDLLHSRLKFGVHDTVFNRYYFSTADEPVRKAIYEKKIAPPGVAPQFMSMEEGVKKMRKGLFAFHMETGVGYKFVGKYFKESEKCGLKEIQYLQVIDPWLAVRKNTPYKEMFKIGMKRIQEHGLQNRENRLLYEKRPKCSGRESNFVSVSMVDCYPALLVLSYGIIIAIALVIMENLWQYRHLIKGKLEFFSSVNTIENFNQFEKHPSHNWKKFYVIDSAKIKKINPSTN

>BmorIR76b

MLFSNAQRSGQNFPLSWIERDENGTVQAYGVAFKIIDILQQKFNFTYEIVIPHRNFEIGGSKPEDSLIGLTNTSKVDmelIAAFIPRLVRFRKLVTFSRDLDEGVWMMMLRRPKESAAgamSGLLAPFNNFVWYVTLASVLCYGPCICFLTHVRSKLIKNEERPLRLSPSFWFVYSAFIKQSTNLAPEANTTRVLFATWWLFIILLSAFYTANLTAFLTLSKFTLDIETPEDLYKKNYRWVSVEGGSVQYTVKTQDEDLYYLNKMVTSGRAEFRTLSPDQEYLPIVKAgamAVLVKEMISLEHLMYGDYLTKTREGVEEAKRCTYVVAPKPFMKKPRAFVYPVGSKLKSLFDPTLAYILQSGIIDYLEHKDLPSTTICPLDLQSKDRQLTNSHLMMTYYIMCVGLASGLAVFVVEILVKRYINIKIKPIDKVKLKKFKRSKRSPRYDDSGPPPYESLFVKPKFKDSEKRWKMINGREYYVYEDARGGTRLVPVRTPSAFLYR

>BmorIR87a

MTTGNSDQIAKTAECVLKLSAKYFVERKALSGSIVIINVNSYSSTTQGLLLKTIHSSIKYSVMAKDSFYPHANASHFPEKAKNYMLILEERTELKRNIFQLNKLPSWNPLAKAVVFYQIKGNESAQRIAIEFINELREHKFFRSIIFINNGTESGVTSYTWRPYSENNCGGKCDSVYVLDRCKNNIVEQIEPQPEWFPSNMNGCPLTTYAIVSEPYVMPPIRKIPNAKFDDVYEFQKGGETNLVKTIAEFSNMTLIVRLSAIEENWGIIYANGTATGAYGVLRNDSVDIVFGNIEVTKQIRKWFHPTISYTQDEITWCLPKAgamQASAWDNLVIIFQWTIWVATFTSLILMGLLFHYMYYREKNKKITKWPTNSLLMTFSMLLGWGSHFEPKTATFRILIFGWLCFSINMGISYESFLRSFLMHPRFEKQIATESDLIQSGIRFGGREIYRTYFESNDASSSYLHTEYSSTTFSEGIRRAALNRDFAVVSSRRQAEYQDQKLGKGASLIYCFPESDNLYKYSVVLLARKWFPMLERFNGIIRSVSENGLINKWNDEMFIHRVSLEGASTIVPLSIQHLLGAFMFIGFMYGTSAFIFLVEVFVGFVQRRAFLSAFFCGKKKRFSAVFKVKV

>BmorIR93a

MKIWVLGVLCLAISVQGEDFPSLITANASIAVILDRQYLGDKYQTVLDELKDYIKELARVELKHGGVLVHYYSWTNISLNKGFLAVFSIASCEDTWELFSRTEEEDLLLFALTEVDCPRLPQRSAITVTYSEPGEELPQLLLDLRSSNAISWKSAVILHDDTLGRDmelVSRVVQSLTSQIDEESARPVSVTVFKMKHEMNEYLRRKEMHRVLSKLPVKYIGENFIAIVTSDVMTTMAEIARELLMSHTMAQWLYVISDTNAHASNLSGFINTLNEGENVAFIYNITENGPDCKNGLMCYSQEMMSAFISALDAAIQAEFDVAAQVSDEEWEAIRPSKVQRRDILLKHMQQYILAKSVCGNCTLWRALAADTWGVTYRQNDVPEQINEHANGSTGVIEHLELMNVGIWRPIDAMTFADLLFPHVHHGFRGKELPIITYHNPPWTFLQANESGAIVKYSGLMFDIVNQLAKNKNFQRLPHPSNRNALLLHGRNRQGGGTYPCGLTKGPITYNNIPLYFRAVFIAHQAgamVNLKNNYYRCINYTIPVSTQPHTFIVARPRELSRALLFLLPFTTDTWLCLGFAVILMGPMLYIVHRLSPYYEAMEITREGGLATIHNCLWYIYGALLQQGGMYLPRADSGRLVIGTWWLVVLVIVTTYSGNLVAFLTFPKLEAPVTTISELLKNSDAYTWSVTKGSYLEMELKNSEEPKYKRLIKEAELLKETGGIEGTIHAARGTLDRVRGQRHLIFDWRLRLTYLMSADHIATETCDFALAVEDFMEEQVAMIVPAgamSPYLPVINKEINRMHKAgamLISKWLSAYLPKPNRCLKISTVTQEVSNHTVNLSDmelQGSFFVLFLGNDKIYVYMYIAELI

>BmorIR143

MFSVKYPRFYLFIIQIASNFGDAVTMPQLNSSINSKSATDCLIKVCYADLSFRRTVILKHVSYESDEENAFYNEIIHAVNNNNIQLVVLEEIDNLNDTINIDDADWLVVVYFKNCKALTEFNVKIVFEKIKYFIIVSDDLNEDCTSKMKTIGNVINKYDVTFVFNENKEDNFKFMTFIPQIDEETCKEIVTLPKIVNICANGQIERKSIFPSKNPKDIKKCPINVGMGSLYPFGIINHKEKYKTFDPLNETEVRGLDVDLVKVLVNQFNGTLNLYFIYKKEENPFGQLDFIPLVLNGSLDVIAgamGFYRIYGNVVAYSGIYTSQAVTWMYVANRTTKSWQSLIVKIDGLYIFVIFHLIYSYVWYFVRKFDEQAVDFRNTILYSWGALVGTTSLQDALSLKQRILNLTYLIMCVHLSAYVSLHLYYFLTVLEPPELLKSNDDVMRSGRPAFLIPISKYFVLDEKYLSFANASEECTKFQDCSDLSLLRNGVTIILQGFFLNYQARTAINYEAKVLSAAENVLTVYYEMLLRKNSPYVERLQKLMTHLFEAgamIPDRFYRHAIGLTVIGKAHSACQNTVSNSYSCQSGCKITFDQFAgamVFYLWLFGCVLSCGAFIFELFSKFGRA

>NeriIR1

MDTMHQLLHNNYRVVAPDEDGWWELLSSATDDDASGLAGTASLVDSYPTGIKEIITSSKTDKPATFFASKEYLTYIINTNYTKSKVGKRQIFHISKQCFLTQMISIILPPGSLYLETFSDAVMRTAESGLIEKIRKDIEWDMYRGAARASLQDGLTLEPMERELTLDDTQGMFLLLGVGFGLACLSWAVEVGSWAQGRDVGALGRASLQAVSRRVSRALLQPI

>NeriIR2

MSAGAGFYLQMLGGGQLPLSIIVDYFTHVSVQSLLLVVCPPSGGNVELLLRLSASGFSVSFSPESLPSLPIFRTGALMDLTCPSAKDVIKKFTKSRNFGVNTEWLFLDYFNSTGDFQMTPGVEKVRDAYILPSSSATLLQTDGSKLSYYEVFRPGVTRPMEVHLRARQRSEDGFAPLPPLSPARLNFKKITLTAAAVLYHMEKFTGFDSYDHPEVDTIPKVNFPIVQTLSEQLNFSLTLLLIDDYGWQTNMTFSGLMGMLQREEIDMGATGIFMRPDRIVTVDFTGDTFQINNKLIFKQPALSSVSNIFVLPFTVMVWICCLALTTLTCGLLIFDNCVTTGRREAFIGYVNSFDIITTIIGCVCQQGATIVPKSGSAKLTIFLFSLCTLFFYTSYSANIVALLQSTSPTIRTISDLTNSPLAVGVQDVIYNQIYFEETTDGDIQKLYHKKVLPFGRKAFMQPEEGISKVRSGLFAYTVETHWGYKIISDTFLESEKCGIDEMRLFLLPKLSIPVIKKSGYRETFTVKNTLQRDVGLHSRVKRRWLLNKPVCDSRGRGYVGVGLTDFKPALLVMGYGYLVAAMVFALELLVHYRLKLIKNDETLQAWQEIESNFRKKKKF

>NeriIR3

MLPRPGRVGGWMVPFLPFNLQMWAAFFFSLALSTLSLYFVTRVSVKYTRFREGLLAKMQFTTIEDSVMRAIGLSVLQAPSSRLIGDSPNRYLFTSFEFLYLILSAMYSAELASYLTVPLFNPPIDLFHDLARSGIPWLATQDVWVYAISESPDPDARAISSLFKVLPMEELKAKAKEGKYGFPTERLPGGSITEQDHLTEDVIPLYHIMKEPIYGSPLASVMKKGGPYNEHYDRIINRVVQYGMTLYWEADVSRKFLNSKIQAEYKEAKSPHVDTEPSVIKLSHIEGILYLYFFGICT

>NeriIR4

MRLGIITVCIALYFKTSITLKLIVLKEENSTIWDDSGGRDGWQAEEVIELDREHASDSFQRVCKTLGEGGAWLVVDLTWSGWSEVAQLPGLRYIRANLGIAPFLRAVEAASVKLRNSTDAALIFQKNSHLEQSLWYLVSESVLRVTVVEGLDNETAANLLDMRPSPSSYVIIADTPNVNRILEAAVDNKLVGLDDRWVLLFLDMDYKQLNRKLLQKKMMILNLSARECCRKGEQQPCQCPQPFDIEKEGIDQFKDLLQNVITGSLKAGVPSTDVVYDCATGGSNNATDQQIILENLNSAMSESWALWLDSNRSHLVPVEVSLRVELEISVDGGPALVDLGKWDSKFGLRPAAVLPKVKRFFRVATGYNMPFAYDSKTKKKSDGTPEVEGYAVDLINELAKKMDFEYELVVSDDFGHRDENGTWHGLIGLLATGRVDMIIGALTMTSEREEVIDFVAPYFEQTGFSIIIRKPVKKTSLFKFMTVLRVEVWLSILGALGLTAVMIWLLDKYSPYSARNNKDKYPYPCRDFTLRESFWFAVTSFTPQGGGEAPKSLSARTLVAAYWLFVVLMLATFTANLAAFLTVERMQSPVQSLKHLARQSRINYTVVKDLDAHAHFRNMKNAEEILYRKWKEITLNSSTDQSQYRVWDYPIKEQYGHILMAIEKAGPVADLEEGLRKVQESEKAEYALIHDHLELKYHVYNNCNLTQIGEPFAEQPYAIAVQQGSHLNEEISRRILDLQRERFFETSSAKYWNSTEKAKCDSVDEDEGITLESLGGVFIATLVGLFLAMLTLGVEVWFHKKKLKGKINTKGEKKIPDFIKDEIFTSKEFSHHNIGNKKASLLNTKLKPKVNQIQVFPRGQLY

>NeriIR5

MMYSTLSKRASTKSMGHTCTSANFKVIEKSLRFQNAVTAFRQLTNKSPLYNVKHLPENYEAPELLISYHQPQRIGIFLDISCDRGVYLLQGSGDLFNASYSWLLWRDAGDAGAVTALFDDTRLSADSQVLLAMPGPLLYDLYRMHSSAETLLESLPPEGAAYRDPFNRTDFGGMVFNASLMMIDIKFNESNLIHPLLDKTYESGKDMVRRYGLAVFIHLAEMYNFSYSYVLTDAWGDPLPNGTVTGMVGQVMRGEAEFGLAPAKYIVPRYAIIDYVTSLHIVKVCFTFLQPKLFGSAKALILPLDDVVWYCLAGIGVLSVIAFRILGQFDHTGVSNDSWGGSFLLVIGAISQQGIPDTTERNSTRIVYILLLFVCFFTAIYYNTAILNGLLLQAPNAIQNIEQLLKSDTKLGMLDIPYLHNEMRQNDTLTVRVREKINKAKPKKLYFDAAEGVSMIKKGKFALYTEDEAIYTEILHQMTDAEVCSVSEVVKYNPFHVGAIAKKNSPYKEIFNRAFSLMRERGILNRQLEHWLEKKPECHWRQDALTLGVEPLALAYAVFFFGLAASGLVLGFELLLHRRQTRKKPIDNFK

>NeriIR6

MNNQGYRLLVEKHSGSHTFLENGTGLYSSIWQKMKSQATYLLNSTEEGILAVRDDKKVAVIGGRETFFYNTRRFGIHHFHLSEKLYTRYSAIAVQIGCPFLHNFNDILMALFEAGILTKITEDEYRKLGEKSSVSEEETVEGSPEKTTSNQREDDKELKSMSMKMLQGAFYVLLTGIGFAGLVLGVERLMHYCEKCKK

>NeriIR7

MTPRPTRVGGWMVPFLPFRLDMWLALLLSVFLSATSLYFITRASIKYTRFREMLLRKVQFTTLEDSGLRALGLVVLQQPSSRLIGDSPNRYLFTSFEFLYLVLSAMYSAELASFLTVPVYNPPIDLFHQFAQSGIIWYGTHEAWTYALRGSPDQDARTIVDHFRVLPMEQLKQVAKEGKYGFTAERLPGGSITEQDHITADVIPLYHIMKDYLFGSPLVVSIKKGGPYNKYYNRVINKVVEHGFSLYWEAEVARKYLDSRIQAEYNEAKTIHVDSTPAVIKVENIQGIIYLYAIGIILSFLSFIYEKFKPPVLDSKDSSVEIKSENGQ

>NeriIR8

MWLGVLLAFLVCSLVFYGLAKFQKDLTKETKQEKSKYSFWKDLFKLWHESKGIVKKPSLQLNSGEAPSGLHIFGDLESSIMNTYSMLLLVSLPKMPTGWSLRVFTGCWWLYCILVT

>NeriIR9

LHEVNRYELTTVPFPSSLTSRLRPVWVDSWKAGKFQKNNELFIDKTLNLYGEDIFVATFEYMPSTTKKFTQVILDDDEELMKTSVEYSGLEIQILRSLSDAMNFHAIIYEPSDCDTEAWGKKQLNGTYS

>NeriIR10

MFLLYILIVGEAIVTVIAQAGVQTINLLFITEDRNELANKSLEVALTYLRRNPRLGVRVDQVTRVSISGTDAKAILEELCQAYNVSVSSGKPPHLVLDATFSGLPSEAVKTMTAALALPTISASFGQEGDLRQWRSLDGEKLKYLIQVNPPADIVPEVIRSIIEHQNITNAGILFDNSFVMEYKYKALLINMPTRHIIVRIDDNKDLKQQLTRFRDLDIVNFFILGRLSTIKMALDTANANKYFGRKYAWHVITQDKGQLTCTCNNASILHVKPEPDPSCKERLDSLRTSYNLIDEPEITSAFYFDLFLHSILALKALLDANEWPKDFNYTLCEDYREDKEIVRKDLDLMKYLKEVSEPYSYAPFLLQKNGKSYQEFTMKLEKVAIVNSQSESAENIGNWKAGLKSPLILKDPEAMNNFSAVTMYRVVTVKQHPFVIEYQENGETKFKGYCIDLIEEIKKIVGFEYTITVAPDNQFGNMDDNGNWNGMIKELIEKRAEIALGSLSVMAERENVVDFTVPYYDLVGITILMKKQTTTTSLFKFLTVLESEVWLCILAAYFFTSFLMWVFDRWSPYSYQNNREKYKDDEEKREFNLKECLWFCMTSLTPQGGGEAPKNLSGRLVAATWWLFGFIIIASYTANLAAFLTVSRLDTPVESLDDLAKQYKIQYAPINGSSAMTYFQRMANIEARFYEIWKDMSLNDSLNEVERAKLAVWDYPVSDKYTKMWQAMKEAGLPNTLEEALNRVRESKSSSEGFAFLGDATDIRYQVLTDCEFQMVGEEFSRKPYAIAVQQGSPLKDQFNNAILQLLNKRKLEKLKEKWWNENPEKKTCEKQDDQSDGISIHNIGGVFIVIFVGIGLACITLGFEYWWYKYKRPGGAPTMVKPGGGGGGAHGRNMDKLSVTGLGDFSHHPNFRARNAYNSNLKGRNAFGQPPVSQW

>NeriIR11

MSIINFMKARQRGFGRCDNCTRWKIRSSDTWGSDFLKEKQSLLDIGEWAPRPGPLLFDELFPNVAHGFRGKTLPMFTFHNPPWQIIKYNNSGKIVDAKGVIFEVVNELARSLNFTYSLRPWSNTSNHNNSQSYKFDESVGDIVLEQTTEFLAWDENVRLVQNKKVFLGAAAFTVTEERKKSLNFTSTIKTENYAFLVSRPKELSRALLFILPFTSGTWLCIISAFLVMTPLLCFVHRVSPYYDHVSHRGKGGFTKMMNCFWYLYGAILQQGGGVMPEADSGRIVIGTWWLVVLVLVTTYSGNLVAFLTFPKMDKVISSVDQLMERRGFLTWGMPEISTLHTILKSTDNEKLNELSDGAILHKKFSNDIVDQIREGKHVYIDRKSALLYVMKQEFVASNHCGLTIGEEEFLTEELAMTILPESPYQQIINDQIYKMHKVGLINKWLEEYLPKKDKCWSKILSSESQTHTVNMDDMQGSFFLLFLGVAFGTMLIIGEFLFKKWKRTQQRQAIHPFVS

>NeriIR12

MESLIPIVIVINYYQSINIGSLSVILPCSSAYSKVKALRELSKSHIMTSFSLNSVREEMNMKNGAILDVTCSSSADILHEMSSRKLFSIDCEWLLLESSNEIGLFNKTYLSPINEDLYILPGSRVTLAQYSVNNGMIAFFDMYRIVPWTPTKFELMTLKQLNSTKNIVLRKERSNFEGVTLRTGTVILLRDEFKGFGSFENLEADFYAKALIFVARDIAFQYNFSMTLLVEELYGVMDKNGNFNGLVGKIQRNEIDLIASGVIMRQDRMSIADFTGSVYEIRSPIIFRQPSLSALSNIFLLPFSKSVWFAAVITSTLFAFVLGISYRIGNYLHKLSDRFDISIAETVTTILSCISQQGPLAIGDAPRARIALLLFELMIFFLFVAYSAKIVALLQSSVQKIKTLSDLIESPLEVGIQDIIYNRQFFQETNNPEHMLFYSKKMKPHKNDAYLEPEEGLKRLKSGMFAFKVETEWANRVIANTFEEKEKCGLGEINIFDIPKMSMPVPKRSGLREHITRSITRQREVGLLTRVTKPFTSSKPICNSHKLYTSVNLVDFKPALKTALFGAVLSVSLLLIEIFMSIRVILLKKIKESRKPEQLFRREI

**GR**

>CchiGR1

MELDNLTEFNTAHNRRISMLGIDDKKTLPPPGKRRRITFAEEDEIINPEDVDPSVGRFRNLVSSTVVPVKKPRLSVDLGTERVKFSSGEVHPNLLARRGGVKGGGGGLYDSLYTDMPGPDSSLSSSPSSLLLSSSMASKLGLSLPNPAPDVELEDPTMPPPSVPASKATEPTDTTSGGDQEKYQEPAKKKYAKEAWPGKKPVPSLLV

>CchiGR2

INPYIATLLLGVAEIFGAFICVLLIHYTGKRPLAIISTGGAGLCFFVVAVYAQFHQSHGWDAPVIPLVFLLGFGFVTYLCIRLLPWMLIGEVFPNNIRSTASGAAASSSYIFGFLINKLYFPMRDMLDLHGLLYFYASVNILGMALLYVFMPETESRTLSDIAQHFADKSKTFDTKIVRTDKIKVGGKDAAKVGVENLAL

>NeriGR1

LELDASEMYFGDKVGNGIRRNTRLASFKTRKRNGHIGNDLSPAQNILSYFQNEDKKLFERRAKVENIRRDNVQVELQIPLTIGQLISIFPMNGVRSSDPTDLDFRWSTFKFAYSAFIITFSVSLLVFTLMKGFQGGIKINNFGDALFYGTSALLSLYFLHIVRIWKDLIIKWAAVQEYMSDLKDVPVRKKINFITVWLTVSSLSEHVLSNSDSLLREVPPGVSKLRQFLESRHQQVF

>NeriGR2

MWAVTLYEIRVISRDLVRSLSFEGTSDLGRKRIIWLKLSCLASMLGDMFGHTGLTYLILLFTTFVPTSYGLLAFFFDHSVTSNAFWGLVITATICVSAKFMICDAAHRTTYEVGEVFSEELLQQDLSKLPQNTTNEVVLFLQTMAANPPHIQFHGFITVNRGLFVSFVSNTVTYLVVLLQFKASSMSVPVQSNITTTTHLYEM

>NeriGR3

MTLQLSELAVGLGSALGAVLVSFTVILLVSSVFALYALLTGRKENSPVIATAFAGAAFVAGAPLIILAETGHRATQVMRDDFTVKILKKSATSISESCRSELRLILEGLAAFPPVVHYSGFSSADRKTLTSIAGSVVTYLIVLGQFSGKAHETQLQTVAHNGTEGMQ

>NeriGR4

MVIFIQLAYKWKNIILMWTEITEDLKFDGRDTIKLLRNLTYSIFFLSVGEHIAANVNAFYKDLSKDSDNLKVFFLNKFFFIFNMTEYAIWKGVLLVAIHFQMTVVWNFIDIFIINVTIVLSKMFQFYNNNLIKDRSIIQVILSVHLLKIQSRLLKAKTLGI

>NeriGR5

MNNNISNLLILSFGSNLFFVCMQLYYTLTIGSSIAKNLYFITSFVFLLLRTCTVCLSAASVNDNSKKTLDILFSMSSTQYNLEIERLVDTVTADTVAITGYNFFYITRKLILGVAGTILTYELVLVQFNSFHEES

>NeriGR6

MITLLISTVFGIYTLLVGRKNNTICVSASLIFFSACAVMPILLLCEAGHRAANVIKDEFTLQVLKKDVTALSESCKAELMLILEGLKEVPPEVTYKGFASSNRNTLTRMLGSVVTYLIVLVQFSESDCGAWDHFLDNNATKETG

>NeriGR7

MRRNPSLCSDEQLRLYILMKPHITFSACGFFTLGYPLITSIIAAALTYLVILVQFSKDAF

>DpleGR1

TMKVNVYCEMHVCLRVLMRLCRWAGFFPVEGLDKLNMCEIRYNIRSFYAFYYIITLLSQTYFAGYTIYWFFTSNLGLETMTNLLFHATGVLSNILLLNLARKWFKILRRAADIEKRAFMICPKTINVKKDRITWSLMTLALLEHLLSVVYKYQGLKKWNKYSSSSYLVLLGKILFELTFFYYLQCLLLSLYLFVILSQVVVLQTTFLWNFTDVMTICLSTYLTGFFQNFNNIIEENQKEVITLTVKWGDIRILHTHLVALVMLVDKNMCYLILLSFFTNLFFICAQFFYLMSKVQSIEDFVYYFYSFSFLLTRSCVTSFLAANIHTVSKRPLMIIQHLPSAEYDIEIQRLIRQIRYTTTALSGVFFHITRGMILQVTSNL

>DpleGR2

SWKCFGLLLSMTGQILMTIMCIYNTVYSKASLNIETPVIFYGMTCITMAMFLRVATLWPKLVRHITAVEESDPNYDTSLNRKCNVTCVIILLLSSFEYVLSLLFAFVKASELKNKDSWCESFAEYLYPWVFNYLPFSPLLGVMVQLIHFQATFIVIFSDLFIICMSYYLTSRLRHINTKVLVARQKHLPEIFWGRTREDYTRAVKLVRKVDDVISGIIFISFAHNLFFVCVQLFHTLDLILESWAFLACIHFLHRRSSALGGYKTETYFVFSLIYLIIRSVAVSLITSQINTASMSPALVLYDVPTSLYCIEVQRFLDQVTGDKIALTGLQFFRVTRKLLLNIAGTIVTYELVMFQFD

>DpleGR3

DFIHTLTNVLSWSRWVGIVGLKRHIWKIYGACMMCLLLAIEFRAIYKVFKALAGWAVDTVGHRSVTARLAGTMFYSAAIFALIQCNYLSHSWKETSKYWSSLEWELSNKHVPIDKTLRRRMYRSALILIFCTAAIYLMNYLVVIDFKCKISYCLKTFVLRTHGFMLLEREYSDWIGVFILFVHVISNIIWTLQNLFIILMGMGLTSRYKRLNSYVATVVRGNDRKEKMSEKLRNQEVLLVYTWKKIREAYTKQAILVHRVNKSLGGLILVSSFFDFYFICLQLFYGIVQGISSDGIKHLYFLIHFSWVCGRTVYTILSAAYVNEYSKLALPHLYKCDSQFYNTEMKRLQRQISRENIALSGMGFFNLKRSIILQV

>DpleGR4

FQDFILTISKVFSGVRWIGIVGSSRIKWKLYALILFCLFLAIEGQASKKVMRALAGVAVDISGHRSVAARLAGLMFYTAVLLGFIQSNRISNSWNDISVYWASVEWSQNIPNMSRDKTLRKRMYRVTLVIIMSGSVVYFLDALQAINFQCELPFCLKQIILKTHGFLLRESEYSDWIGILTLFMSVIANIIWIFQDLLIILFSMGLSSRYRRLNECVEVMVAENNRNKNWNILQIYAWRKIREAYIKQAILVRRVDNTLGGLVLVYNFFNFYFICLQLFYGIIQGISNDGTKNYFFLIDLVWVAGRTIYTILSAADVNKYSTMALPYLFNCQNNGYNIEVQRLQKQLIKNYVALTGMGFYNINRSVVLQV

>DpleGR5

KNNLYPQIKNIPNGFAKQISDRPNNKIVFLDVPSPRTEQLNHITTNSIVPLRDNLISPQINSDIIYENIKPVFTLLRIMGVLPLTRTSPGHNQFKMLSPSMVYSIFCFLGLVSYVMYLSLHKVQILRTTEGKFEEAVIEYLFTVYLFPMMVVPIIWYETRKIANILNGWVDFEVCYKKLSSRVLPIKMYKKALTMAVVIPILSTSSVIITHVTMVDFKVMQILPYVFLEILTYLLGGYWYLLCETLSFCAGILAEDFHQALRHVGPAGKVAEYRALWLRLSKLAQDTGLANCYTFTFVNLYLFLIITLSIYGLLSQISEGFGIKDIGLFVTACCSIFLLFFICDEAHYASLNVRTNFQKKLLMVELSWMNTDAQTEVNMFLRATEMNPSQISLGGFFDVNRNLFKSLLATMVTYLVVLLQFQISIPDNTQDTYEDEKLITNDTTATDATKITTTLATTILTTLAKKK

>DpleGR6

FYKQISSFFGLAPLRFESRSHGFSVRMSNPMCIYSYILVTVLVICTVFGLVAEINVGVEMSVRMSSTMSQFVSTCDVLVVVITAGAGVYGAPKRMRNMLKFMESVASVNTSIGAHYSLVAERKLCAVLLAILIFFSVLIADDFCFYALQAKKFDRQWEVVTNYIGFYLLWYVVMILELQFAFTALSVRARFQAVNDALALTARHVSISEKVKEQTPMNMFAIQVGERREQPQSHPVIVRNTVSGEPKLIVPPCEAIRRLASLHGSLCNVVHRIDNSYGLPLIVILISTLLHLIVTPYFLIMEIIVSAHRIHFLVLQFLWCMTHLIRMFVVVEPCHYTITESRRTEVLVCRLMTSTPSTGMLPSRLELFSRQLMLQSISYTPMGMCTLDRPLVASVLGAVTTYLVILIQFQRYDT

>DpleGR7

IQLFFCLDFGFVEHKSFRMKFIWKFGIFLLSFCLGTFCMVSLFTCVPDYSDIWYVVHLAEFYFFVLIFIYTPRKNTFHSYYIDLKKFDKLVGVHSSSYNIEVKLIFFWLLTCSLDIILATPHYVLDGGYIRPKFLFYIILFSYHLPLLILSFSFYSTFLRLKTLKNLLHKREIEILRAHQLYKYMIDSLDNMMKIYRYVFLEDLVISIPAIIASIYLLLKDLTIDNFILQLNFEIFEPYIALSYIISIKIGPSLAATKLFHVAITIKELLSNRLIGEFDECQCQFIERFIRYIDTRPFKFAMLSVIPMDASLLIIVSDFSITYLIVPLQLTHIY

>DpleGR8

ISKPLRFFFIVNQIFASLDFGFKEYKTNKMRLLSKLGVIAVSLIQGTIIIVFLFIKMNFFYQKWVLKLLLEYYSYVLILIFTPDDRTFCFYLMELQYFDEQMGIQSSSYKIDFKIIAFWCVSSITDATLMWINYLTSDKFINLLTLNGLLYIVKRTCTLPLILLFFVFYSMLCRLLKLKLYINNKKISVLRALELYRFLIDAIEKVIGTFKYI

>DpleGR9

TVDTFSPLYWQLNIFSLQCQLNVNIYFQLLRMALAAIISCLITSFSLYYKIVNIYHHLLASIKYTDTVQAIYDYLQYVFDLFCVFKYGSDIFREYINQYAYIDCLLDMDYYRYIRRNLMKVMVFLFCMWFCSSVFDFVTWDIGFGFVSTSAFVMGYVYTLIKILSNLDLTAHIMQIEARLRVIGDTIQNSYQSFDNICPKFEDTIGKKNWFYNDCRTSTKTTDVRRNPCLCSNDVKWLSRCYVLLTEQVTFINNMFGRRILLNSLSFLLDLVRYINLSVRILIGTQVIRCIGSSKQPATSTVFKAVNCAIVIFSLVSHCERTYRQRDRIIKLIDHLTLFKKLDDQLLSSFMDMRNLIVSRSITFHTDFYTLNYSWLVSIASVVVTYSIILLQNFN

>DpleGR10

KEMKYFFNLIQIITFLDFGFNEHKSYKMKLFWKFYRMISFLSLGTYILVLLFTVIDEYFTTWGIKLLVECSFYFLILMFTPEDKTFSSYIIVLENFDKQIGVVSSSYRILLKIILYFSLGCLFDILIVCLLFLSSDGDMNMICATYALFFIHRRISGFPLKIVFFVFYSMLCRLQKLRLCIKKRKFSVFRERLMYKILIDSIENTLEPFKYIFLVCLILNIPGLMASMYLFLELTQLEEEMKFTVITYLFLIPIIITIILPTLAASSLSQEAQNIKMVLQNRFIELKYSNKTTERFIDFIDGRPFKFFIFSFIPMDATLLVVILKYCISYFIVLVQLTHIY

>DpleGR11

KRMKYFFITVQVITFLDFGFIECKFYKKLFWKLLTFMSFLFFGVMALVYIFVAQNTYFQIWSVKSLLECLFYVIILILTPKDKTFCSYVIALEYFDKEIGVSSSRYKILPKIIFYFALGTIFDLFFLVLLFFSSEADIITIRYTYILFNVPRRTASFPVMVFFFVFYSVFRRLRKLKLYIVKRRVSIFRARQYYKSLIDSTENALTYFRYIFIVSLLFNVPGLMASIYCLLKTTSHKDSINFTTLTYIDLIPIIVVNVLPTMAASVLSQEAAQIKMVLQNVLLRQKCISDENSITEIFIKYIDVRPFKFYMFLIVPMDARLLVIIVNYCMSYLVVIIQLTHLY

>DpleGR12

KELEKLSYDILDDDVVKIFEALHFQQKILGTLRVYIKNSFATAPSKIYTYYKIFFWSTIGLMYSYFINQCEIVDDVLIEKKYLKLAFLVDYAVIVAIVLRNTLQKNKYHLIYVNIQRLDRNLQIKYKKINGYMFMFNSCFSIFGSIFGILWGFLFHYNIIKGTCLADFAILLPGMASAVDVTLILVIITFIIIRVRYMNMKLKDITKKQSKINSCDPESLTDENIVWNNLLNGMETILAIIDDFNELFQCQIFFFTCQVVTWAIILIQCIILTLKNQDSVNSTGAMIMTSIVIVLMSFFMALCFLTQYFASHLLMVKTKCIKIMSSANNDTTRRNAKRMLLLINAKSELSVFGMFTLNIDLPVQVLAIITTYTIVLLQF

>DpleGR13

EYLSDDLLDEGYLKLFAPFRFVNYFFGSCRIDARDRFVTAPTWGQKAYTVIILILAAALHVYVISYNISKLYEYQIIYNICVLFSTLNFIMYALNIIHVRFMNNENNVKFYIQTQKADRKLKISGNNPCNMVLYYINLCSVGFLIVLTLIVLSVVYIYSKELFASFAGLLFLQLTSLMEWIFCSNIIIYFCLRLRFINAIMRNHLKGSPNAREVNSKFFLPTRKRMRCIVANTHELNSSATDEYLKDIFDVISRFQELFRFQVLLFCFKFVLTALLVFEFILLAMQNNVGPLRDKARKMIKLIDEKPHHSIYDMWNMDASTMLNMINVVTTLLVTLLQFALL

>DpleGR14

QYFFDVYFINKYGGQTFLHYITTYETIDKYLGITSYEEIKGNIRRMCFILLVIVPLTIVLDFFSCFLTVGWTASIIYTLQYVFYFFKIINTLDMISHVFQIKYRLKSIGNSLKHYYYNSDNVPGELKDLAHRNFFVIKTIHEQKRVPSDIMMFNKCYLLLSKQTYYINNLYGFRILTYNIIFITEMVMVLNLLIRLLFGSLSAEYSWLLIMAAATRFISISSFLFCGVNCCEKSYRQIDRILFWIENLIANKKLSSEMRVAITELKGVIQTKPICFHVLHFYKLEYGIILSIASAVVTYTVIFLQNVS

>DpleGR15

QYFFNVYFVNKYGGQSFLNYIETYETIDRYLGITSYKDLKRNIKKIYYLILAIVALTILLDFICSFIMLGWIASIIYTLQYVFYFFKILNTLDMISNICQIKYRLKSIGNSLKHYYYNSDNVPGELKDIAHHNFFVIKTVHEQKRVSSDVMVLNKCYLLLSKQTYYINNMYGFRVFIYNINFVIEMVMILNLLIRLLFGSMVLEYSWLLIMAAVTRLITICSFLLCGVHCCEQSYRQTERILFWVENLTANKKLSSEMRLALSELKGVIQTKPICFHVLNFFKLEYAMVLSIASAIVTYTVIFLQHIS

>DpleGR16

LILGFVQYIVDIHFVFKYGREFYLQYIDRFELFDRTIGIAVYEKIKRTINRICVFFILITFIGSFVEIIIWYLSYRSSSHMFYMLYYIYDFLNLLSELDFIANCTQVLFRLQSLEHKLKDFLCHVTNLPVPYNENNHSVVKSSNKNTNISAHTLTETVHRQNVVLLLNRCFLLLTDQFDYINIMFGLRVSKSDNKYVVLLFTISAFIMIYLNFRYCSPAGSRYLPISVTIVQIIVNSIVVFSIIHICERNYGQIERIIASIDGILHEKDVRPDILDSLKDLRNTIITRNMNFNAVKFFKLEYPILVSISSLLVTYGTILVQNV

>DpleGR17

YYMSIGMISDLGTSLSFVQMFHLLFNIQYFFDIYSVHEYGGHAFLRYIAIYQTIDKSLGVISYKEIKKNILEICLTMFAIVPLTAVFDFIYSVVTLGWLVSIVYTVQYMFYLFKILNTLEMVSHICQIKYRLKYIGDLLKYYHYYYDYMTEVHQHLSYRKRFFINKGNELNQIPRCYLLISKQTNYVNNMYGFRIFINNVMFVIEMVLLLNILIRIIFDSLVIDYSWLFMMAVATRLISICIFLLCSVHCCEQSYRQTERIFFFIENLIANKNLSPGMRKVVTELKEIIQTKPIYFHVLKFYKLEYGIILSIASSVVTYTVIFLQSVS

>DpleGR18

KNILVRYKSVLVLLLVCGFNYKVFRMSPKCKFILNIYCAILIIIIGISTLYCCEITSNTMVIWPLIEYILSATILVFYDSKLTKFAFELSRIDSFLRTDLKYYSRGKIGVFLLILTLWLVRLSFMIIFCFLYSCFSNVIFFVISVLSLLALDVNRIWRFLLLEIVRYRFLLLRQKINEMIENNSKISISEQIHFKKNKIKLCNKLYKSVLDVIDIISPELSASMFVSIICSLPKLISNVYHILLVFENHEPIQSIGFVLIHMCHTCFLLFSPCVVVELQCLEVDRIRLILVNQLLIEDDPKIRDDLQLFIQYTDVRKYQFKIWRCIPLNISLPIEIANICVSSVIVVINFTHLY

>DpleGR19

EYLSDDLLDEGYLKLFAPFRFCQLFFGSCRIDARDRFVTAPTWGQKAYTVIILILAAALHVYVISYNISKLYEYQIIYNICVLFSTLNFIMYALNIIHVRFMNNENNVKFYIKTQKADRKLKISGNNPCNMVLYYINLCSVGFLIVLTLIVLSVVYIYSKELFASFAGLLFLQLTSLMEWIFCSNIIIYFCLRLRFINAIMRNHLKGLPNGREVNSKFFLPTRKSMRCVVANMHELNSSATDEYLKDIFDVISRFQELFRFQVLLFCFKFVLTALLVFEFILLAMQNNVGINLSIKKFILLPFQTVIDLMMIVVFCVRCEAFRIEIQDIKRLSTTLLSIYQEGPIREKARKMLKLINEKPPHISVYDMWNMDASTMLNMINVVTTLLVTLLQFALL

>DpleGR20

EYLSDDLFDEGHLKLFAPFRFCQLIFGSCRIDARDRFVTAPTWGQKAYTVIILILVAALQLYIICHYVSQLYEYPIVYHINVLFTALNSILYALNIIHLRFTNNERNVKFYIKTQKIDRKLKISDNNPCIIFLYNVNLCSVVFVIVMSLIVFFVVYIYNTELFVSFSGLFFCQLTSLIELMFCSNIIIYFYMRFRFINAIIRNHLKGLPYKKGRNTKFFIPTRSNMRYLVASLHDFSTSQIDDYLKDIFDAHLQFQELYRWQVLLFCFKFVSMAILAFEYLLLLKKHNVGPLRDKARKMIKLIDEKPHHISIYDMWNMDASTMLNMINVVTTLLVTLLQFALL

>DpleGR21

LKIRIFFGFYHNVFENSIILRLLLKVILIVFGAAILYTGTQSRSKEFSKLNHFIKALEYITYFVLTLTTENSYLLRYFRHDPIIDTKRSNQAYQQVVKFTLSFFLLTIVYYVALYSVVTYAKYVSNSGYVLEYISLIFFRLSSELGRIPVLMVLLLFYYRTKLFSSFVQEAALNTTSTFNLKRYIVMYSRNLDSLKETDGCVKPMVIFIYV

>DpleGR22

LKLRMCFGFYHNVFGSNLSILKYLLKIFLVIIGCVILYTGLSCKVFKYKKISHVFRGIEYVTYFTLTILTENKYLVRYYQTVVIGDTDYWKKIYIVIFKIILSYFFIMILFYVFHVSFVASIKIIECSQSFRDTVLLCVLVFANELGKLPVLMILLLLYYRIKLLCSTIRRNVLNTTSIVCVKKYINMYDRSLDELKKTDGYIKPMVIFNLI

>DpleGR23

SDRYENAKISKTLKYFLILLLTFFMLDFGYKFKYKKINIAIRYIIFVVSSLGAFMLLVVTILDLYTFNHVFSYLYLAQYLLNIYILLYCDQKNSLYDILENLYFIDFQVYFQNAYKIERVLFSAIFFSFITNISVNVLFCKYFSFQTTVGECSLISTLAIGLNFPLLLNFFLFYSIYCRLKKLRDVVQNNCLNIVSCQFLYKFLIDSTEKGKKVYDFLSIYSLVFYLLTLELIKKTRIFQTQILYDAILCFYVLQYMILIFLPAFFSTMLSKEARKLKLILLEILVNKRDSVYKKDIQRFVEYIKARPFQLYVLHFIPLDYKFPFFVMNIYLTYTITIAQFS

>DpleGR24

INIIMLARLFFGLYTKFSSNKLITFASKIFCICTILTVAYYNFKFYIGIREYDFSAIFLSAIILYIIYGLVSLLSDGENVYKYLYRLNKIGSVLIVELKIEKCVSYFYLYLFIGVIIGHSVKFIIFFMSSDTFKICHYILTSFLFIARSLSYFERVIMFEMTWNRMRSLRKTIRNNVDFSISEDEESNEIKKVQQYLLIYNKLLNNYRCIGKATKLMV

>DpleGR25

FLSKYENSRVSKNLKIFFLILRTVFLLDYGYKFESNNVNIAVKYFILVVSSLCAFLLVFAIVFELYRFSELYCYLYTIEYLLNIYILLLCDQKKSFYAFIEQLYIIDFELNLQKCNKTEIALACYITLILFIHTTLNGIYYYLQRPKMLVEFGVVCVLAVGIFFSLVLSFYMFYLVYWRLRKINEVVKNNCSNIALYQHLYKFLIDSTEKSKEVFDYLFLLIIFIQLPEMMIELYGTIIQSKDITSVTIWYDVYFCIFLARYLTLTLIPAFFASMLTSESYRLKLTLLEILLCERDSGNTKEIQRFIEYIEARPFQLYIFNIIPLDYKFPLIGINIYITYTIVIIQFSRTHL

>DpleGR26

RIQYSRPESTKISKTLKIFFIILLVCLMLDFGFKTKSSKTNKALKRYILLVSSLCALLLLIIIIFGLHKFTLFYCYLYLIQYLLNVFILLFCDRKKTLYATMESLSFIDCNINFQKTHKIELTLIIYVVFILLINILITTISFIFLDNIIWANSMVEAIVFFIIAFSLWFSLFLNFFLVYSVYCRLKTLRINVNSNYSNIVSFQYIYKFLIDSTERCQEIFNYLLIMILIVQMPEMMQLLYNIVKKLKFTLVDIMNDILACIYALQYMIVIFLPALFATMLTNEAHKLKIVLHDMLLDERGSVHTHDIKRFIDYVDARPFRLRICNLIPLDYKYPLIIINVYVTYTIVIAQFS

>DpleGR27

KITFIVIVMLFIKLFSICYKMVETKSTSMKFGVNDSYELIAIFNLLRYITDIFLVRKFRNFYLKYVSRYKTIDNKLGRTYYYRIKQSIRRTFIFFSVIGSTVFISDIIAWGLKDELTYEFVYFCDYFYDFCKLLSDLDFIGNCVQVKYRLKMIRNILFAHYHHFENLPEAFINFKYNEQCSCRNINVKHLNNFINENGKQKMPLKKCYLLLVDQCSAINTMCGFRIILSSLYYLADVTLSILFTIRLVSNTKLGNMETSYFTIISAVMQAVVDTLVVFYAIYICENSYREVEKIVCYIEYFLLNKKMDPRDRHSLQELKGLILTRPIRFHAAYFFKMEYPTVVTVASTIVMCIVLLLQN

>DpleGR28

CKNYLRITLLVTFNFFINALCTYYRFPHTKDIALDIFDLFYLEYFYNYIRFNIDIIFVIKNTKKYIEYFETYEVIDSGLGRTFYPEIKKRIQRIYIIIVFWFFASIIDITTWALVYGWVKQIKYIIDYLHNLVKLLSGFDFIANCFQVKFRLQAIGDLLKEWNSCVNNLTGPHTQLKYKHRPTNAFFRHLKLLDLRMKFKQINNSINNNCLVTSSINILFGYRIFLGCMSYIAEVVVMLNFTLRLFLGCLEVSATTSYLSLITTITRMTGNISIVLYGLHICEQNYRQTERILRLIDYLLIDNTMNQEVQESLKQLRELIITRPIDFHAAKLFRMEYPTFLSLASVFITSTIILLQNI

>DpleGR29

IEVFSTFYPLNRILQLVGLSCVFSIDKQLKCYTSTLKVTCILIILLVFNCLSFYYKFPNFTHVSSAALINNFYLFIIILDYFQYFINIYFVYKYGKESHLKYLNNYVFIDNILHTYYIHGRVKSSINKTCVFFLVLCSIISIADFITWFIIYNSASVSYILYYVYAFLQLFSSLDFIANCTQVLYRLQTIGDELQYRGRDISNLSVLYGETVDDVLIKKNQLKTNSSRAVAKPIHRQNIVNIVTGSYRCYLLLIDQSSYINKVFGFRVSVPKLKVNYPLMELLKRNIFVRHGTEYLPIITTSILCVFNFLVIFYMIHICEQNYRQNERIIDCIDRVPGDVQMDPVIRDSLKELRNIIITRKIIFHANHFYKLEYPMLVSISSLLVTYGVILIQSI

>DpleGR30

EIPIPKRNNFFKNEYKTVDQIKKLILFEMCFGLNRQYLFSENKCVLFLSRAYSVMLIILSIICIVFIDLNSRLFNVLKDISGFEYILLSFSALVLNKKKLKKFFVDLNLFDKILGVNEDISKLSVKYWTSIQLSNIILYHFINFIVIAHYKISSYSYSFIYLPFLIHDCEIIFYTNFLIFLLRRVNILKAHAAKIFVDDVAVYNDSNNISNRNTFSGNTNVNINSFYKAYNLLYKSSLNLNSIMGFPLIVILAKSGLATIILLKHLITRIEEDNEDPSIIVIMSIVITVRCFMYTLLITFPCYVSMVTKCEVFDLCTIINDFLNTKKSDKAVRRKIKALHQLSCTNEFTYVLCGVINLEMTLPLSYTSLCTTYLIIILQVSKFID

>DpleGR31

IKRIQPFIIFQIMLGINRIYLCDCKQSVNIISNIYSITLVSIIMLISLLNPIINTSHFVMNSFTTFEYSALALCAVIIMKKNFIKFFNKLQNFDKTLNIHANASFTTPISWFYCGIITISIFNFIQYYHFIIFTNLDINLFVVPLFLGDFIHDMEMLFICILFVFILKRLHILKERTIRLIGNKNINCHSKINHVRSHLKTTDLNISFLHKAYDHLYKCSRQLNFAIGFPVFVYFYFYIVIILLIITVLTFSIGRFMKQSFLLITLCYCTFITKCLVLAIRGTLYDYLNTDNLSKIERRKLKAFLQMTQDNDFSYTLWGVIPLNMSLPLKYYAVCLTYLIIVYQFSKFI

>DpleGR32

SWKCVYLLLSLLGQIFITTMCIHKVLNSNASLNVTTPVIFYTTTCITMVVFFRIAISWPDLVHHIEKVEQLDPNYDMTLKFKCNFTCAVVFILASVEHILSLLSAIAASTACDTEDLTYERFVKYFYPWVFNFLPYSLILGAMTQFVHFQSTFIWNFSDLFVICMSYYLTSRLQNVNNKLLFAQGKYLPEVFWKTIREDYGRAVQLVRRVDDVINGITFISFANNLFFICLQLFNTLESTNIFGGYEAAAYFVFSLIYLISRSVAVSLIASQVNTASNAPAPVLYDVPSPVYCVEVQRFLDQVNGDKVALSGLEFFSVTRGLLLTVAGTIVTYELVMFQFKDTPSGENIT

>DpleGR33

IILDTWFDIQVNIFFNSHLDSTDYIDRLPMVVTVVQSSFALVVFLCNSKNNIKIINSFAYTDCLLKLHNNRGFHEKVRKTNRVAICIFFLILISSIVQNYVESPSAIIYNVIEALIDFKFHLETLFLYLYLKMISKRIEVLNKYLNHFVEKKDKIKPIVISKENLEVNKLGDYIGRINHQNTKITSLAFAYETLGESLLLLNNMCSINIFLYLASNFIYIIISLWSFVHFIRTKDHLSSLPGIIFESSVEISVVIIMCYVCEELTMKRRSTRILVNEIVMDYKLPNEMRIQAKSFFDLIEVWPLQVYASRMFCMNIQLLLGFVSVSSTYLIFIIQ

>DpleGR34

LNFIENVESFLCIYRSYSAFSSLKRFVVSLRILIEVIVIITTMYLRPIVFGYGEIYQLFYFYFYSIFSLLIISLALYKSNEFVKLIKNLEESLTYFRKDDKYLKHVVVKYKIIMSLTILYTLVFFVLMILLFRFQLEYIIKEPKLAICLVVFILCEIRYVIEYIAIYGILLVISEQVKSINRNIIKSTRFLSSGQFCKSDDKMFRVNNIDFSECSRVYIKIKESSNSLNRAFGVQVSSIVIIIFIFLLLYIFADFFEFIAYTMNRVLMHMITLYLLVFGAHSAQSSVDELRRNISKILIHSSPSGDFLRLISNRSIKTVAFGGINIDMALIPTFVMLFTSYAVILLQYNHVV

>DpleGR35

IKIFFILIQLLLCLDFGFIEYKTPKRRFLYKFGACLLSLFYGIFVMMVVVYYVQDDFYFTWTMYFFIEYYLYVFILILIPQNRTFCYYLMELQQFDKKNCVETSSYKLEYKIIFYFIFSCLYDILMMFLYYVYCEDYVKLINLKLLFYVPYRTYHFPLMVYFFIYLSMFYRMGQLKIFLVNRKSSVFQVNQLYRFLIDSIENIMKPFRYIYIIRLTFNIPTILASIYLMLENLQQDGINIATIAPYLSLVQSTIFWAIPTIVATLLTQEAFKIKLILHNRISKEYGNHIVDRNEILAMERFSSYIDARPFKFYMFSLIPMDLTLLVIVLDTCISYLVITVQLTHL

>DpleGR36

KNNVRQYNITVIPKKNKLFFIFIQIFFGLDFGYIVYKSKKNKFLVKLTTFLVSVCYGIVIISMLFEIGDNFYLTWGFKFFFEYYLYIFILIFTSDDRTFYFYQIQLQQFDNKVGVPTSSFKLANKMMFYIFFLFSFNVLVIFLYYGYSDDYELQMYLQLLFFIPLLTYDIPIMILFFVYLSMFYRLERLKILIENRKFSVHSLLQHYRFLVDSIEKVMKSFTYIYLLSLLFNIPGVMASIYYLLETIQKTGDKSFALIVPYIPLLLIITNRAAPAVAATWLGQEPMEMKLILYKRIFEEKDKRQILTMERFLRYIDARPFKFYMLHLVPMDVTLLAIILESCVTYIIIMVQMTHI

>DpleGR37

FVSVMEAIFNKAKWFGIPTYGGNLAVFWTILMLATLSITEVAAVWKLARLLTKSAGRNVSATLSGSIFYGNALLSLMISWKFVKSWKSSTSYWIRIESRASFLLPNSSIRRKVNFVSLFVAISATVEHILSMISATGADCPPEDYFERYILTSHGFLIHDYEYSLWLAIPIFIMSKSATILWNFQDLIIILMSMGLSSRYHRLNNFVKEVVESENEVLAVKKVGIENICFHLRVDVWRRIRQAFVQQSALVRKVDTELGALILLSNLNNLYFICLQLFLGIRSIEGTLINRIYYFYSLGWLLLRASSVVLAAAEINLHSQKALPFLSSFPTYAYNIEIKRLISQLNNDQISLNGMGFFALKRQKLLEVAAAIIKYELILIQYDK

>DpleGR38

CNILLIKLAKRWNDLKMNLASIDLREYIDPSIKFKCSLATIVLASLTIFNIFIADMSKLAAILDCHHSGNTTIFEQYALRSYIWLFKFGIPYSLPLGILLVAIDLISSISYSFIQIFVICVSLYLSSIIQQFNRKIHTLDCKYVATSYWLKLRQDYNQVSRLVRSFDDVINGIVFVSFACNLYLVCLQVYFLLRSDITIRQGFHACPENTSSGFLNGYERIISNVYFVYSSTYYIIRTFVVSFTAAKVHTASMEIAPVLYNVPPPTYCIEVQRFIEQIHGSTIALSGLNFFYCTKEVILSMISTVITYELVLLQFN

>DpleGR39

ILRLNFFIKFQAFFGINRLYLLHYRNSAIIFSSIYSTTLVMTVVSILLFKMSTSTSHFIVANMIAIEYFCLYINALFIRKGTLLKFFNNLQIFDEALNIRQNSSATNPITWFIFAIVTVLIYNLSVFYFYVYRLGFLGDIIHDVEILFFCALVIFISRRLRILKSHALKLYYDDEHLGCDSATLSRNISLNISSIHKFYDLLHKCSRQLNAAFSFPIIIMMLSSAVIDIFLLKNTIILILLLALKKEVKVIIGYSLGRSLKDAILLIVLCYCAFITKNEILIIRNNLYDVIRTEKQDKVERRKLRAFLQMTYDNDFTYTLCGIIPINMSLPLKFYSICLAYLLIAYQFSQFIN

>DpleGR40

RYNIRSFYAFYYIITLLSQTYFAGYTIYWFFTSNLGLETMTNLLFHATGVLSNILLLNLARNWFKILRRAADIEKRAFMICPKTINVKKYDRITWSLMTLALLEHLLSVVYKYQGLKKWNKYSSSSYMVLLGKILFEVFDFTTLQCLQLSLYPFVILSQVVVLQTTFLWNFTDVMTICLSTYLTGFFQNFNNIIEENQKEKIVKWGDIRILHTHLVALVMLVDKNMCYLILLSFFTNLFFICAQFFYLMSKVQSIEDFVYYFYSFSFLLTRSCVTSFLAANIHTVSKRPLMIIQHLPSAEYDIEIQRLIRQIRYTTTALSGVFFHITRGMILQV

>DpleGR41

ERDQRNLLSSQDGDTCEIHDQFYRDHKLLLVLFRALAVMPITRSRPGTITFSWKSRATTYAIFFYIVTTIIVLVVGYERLMILRSIKKFDDYIYSVLFVAFLVPHFWIPFVGWGVAHQVAIYKTNWGKFQVRYYRVTGENLKFPNLKTSIVIISVGCLLLAVCFLLSLCALLDGFLLRHTTAYYHIITMINMNCALWYINCKGIKIASQSLSNCFSRDVSIECTASLISSYRFLWLNLSELLQSLGNAYARTYSTYCLFMFFNITIAVYGALSEIVDHGFRFSFKEMGLIVDAAYCSTLLFIFADCSHKSTLKVAAGVQDCLLSIDVLSVDRPTQKEIDHFIQAIEMNPAVVSLKGYAHVNRELLTSAISMIAIYLIVLLQFKISLPKE

>DpleGR42

YLSFYGTTCLSSVLILRLASNWHVLMKQVEAAELNGYVDPYVKFNCNFATFTLLWFALLEHILSNTAKFLTVLNCLSGNETVYEQFIFKSYSWIFESGLSYSVTLGFLLPVLNAIITLNWSYCQVFIICISLYLTSIIRQINKKILSFDGQAIFVHIYWRILRQDYNRATRLVGSFDNVSNSIVFASFASNLFFVCLQLYNILRNDISTRHGVPLCPNNNATGPYYKYEQMVTNAYFIYSSLFVVVRTFFVSIIAAEVHSVSLEVAPVLYNVPSPTYCLEVHRFVEQIHGNKVALTGLKFFYVTKELVLSMVGTIVTYELVLLQFT

>DpleGR43

IKIFFILIQILLSLDFGFIEYKTLKQKFLLKLMTFLVSLCLGIFIVKMLYNMGDYYYINWGLKFLLEYYLYILILIFTPEHRTFRFYQMKLQQFDSKIQVQTSSFKLDNKIMFCFMLLFSLDMITIFLYYAYSDDYELQMYLQYIFYIPLRSYNLPLIIFYFVYLSMLYRLKQLNVYIESRKSSVLSTLRLYMFLSDSMDNVMKSFRYIYFVCLLFNIPGMMGSTYLLIELIQTKSNELNIGMLIPYFSLFQEAIIRAAPTITATCMSQEAMKIKILLYDQILQERDSREIRIMERFAKYIDARPFKFYIFFIIPMDATLLVIVLDACISYLIIIIQMTHI

>DpleGR44

DVYGPEITDKDGELLDQHDSFYINTKSLLVLFQIMGVMPIMRVPKGAQTTKRTTYNWISKATLWAYLVWSLESVIVIKVGRERYYNFQQNTNKRFDEVIYNIIFLSILIPHFLLPIASWRHGPEVAIFKNMWTHYQLKYLKITGTPIVFPKLYSLTWGLCVFSWALSFAVILSQNYLQDDFELWQSFAYYHIIAMLDGFCSLWYINCNAFGTASKGLATNLHKALEAEHPALKLAQYRHLWVDLSHMMQQLGRAYSNMYGIYCMVIFFTTTISLYGSLSEILDHGFSYKEMGLFVIVGYCMTLLFIICNEAYHATRKVGLEFQVRLLNVNLGAIDRSTQREVEMFLVAIEKNPPIMNLDGFTNINRELFAANISFMSTYLIVLMQFKLTLVRQGTKKVFKSIVDAIFNITTT

>DpleGR45

FYSSTTMITILFLHIAMKWPNLCRYISCTEAIDPLNDYALVRKCNVACLLVLSFAFVEHGLLELSGFVFATDCSPPGKVYETIIVNSFPWFNNIVNYNLNLGIITQLLNIQCTFNWNFSNLFVINISLYLTSRLEQINNRISAIKGKAMPSTFWRNVREDYNRITSLVRKVDKVIGGVIFISFANNLFFICFQLLHTLAPEVRLKAYYNISSQYITFYISLSEYGYEQATYFLYSLIFLIVRSLALSLIAARVHTVSRQPVYTLYELPSADYGIEVQRFIDQIHGDTVALTGLQFFKVTRGIALAIAGTIVTYELVLMQFT

>DpleGR46

IMMTRLFFGFYGKFSNNKLITIATKIYCIGTISIVASSNIIFYIRFRDLHFGKMWLSYIILYITYGLLSVLSNGENIYKYYDSLNKIGTILLVDLKIEKYVSIIYFYLFIGVIIGHSIRLSMLIAHYGHSNMTEHLLSSFLLMSVSLTFFNRAIMFELLWNRMRTLRQTMENHVDFTFNEDERKHKLVISKVQEYLLIYNKLLRNTRHIGGSSKLIVNTMSCFI

>DpleGR47

RATCIAIMLFTFNVSSAYYLLPNLQNGILDFKFSDSVHLVLIIGFVQYLVDINFVYKYGRESYLHYINRYGIFDESMGIVLYEEVKRYINKLCTIFITIVVTASISEIIIWLVTYNSSTQTFFFLYYLYMFLNLLTELDFIANCTQVLYRLKSIDDKLQDFYSHLNNLPGVYKRTVQNYIIKSKTMTINVSAQSVSKLVHRQNVILLLNRCFLLLTDQCDYINSMFGLRILLSCVNFIVDTLVKITLVIKLFSIDLQEMISGPVYLPVVTTIVQIILYSLVTFFIIYICEQNYGQIERIVASIDRILHEKDVRPDIRDSLKDLRKTIITRNMNFNAVKFFKLEYPIIVSISSVLVTYGVILVQSI

>HarmGR1

MNKEEHGFRVYNTNTVHKNETRKREMFQRIDEKDGIKEYDAKDLYGPEITDKDGALLDAHDSFYITTKSLLVLFQIMGVMPIMRVPKNAQTTKRTTFNWISKATLWAYLVWSLECIIVVKVGRERLANFQSSANKRFDEVIYNIIFLSILIPHFLLPIASWRHGPQVAIFKNMWTHYQLKYLKITGTPIVFPNLYSLTWGLCVFSWGLSFAVILSQHYLQDDFELWHSFAYYHIIAMLDGFCSLWYINCNAFGTASRGLAMNLHKALEAEHPALKVAQYRHLWVDLSHMMQQLGRAYSNMYGIYCMVIFFTTTISLYGALSEILEHGLSYKEMGLFVIVGYCMTLLFIICNEAYHASRKVGLEFQVRLLNVNLGAVDRSTQREVEMFLVAISKNPPIMNLDGFTNINRELFTANVSFMSTYLIVLMQFKLTLLRQSARKTLKTIVRAVFNTTTTILDDDFTDDVDEE

>HarmGR2

MTIPDHLFDEGINNTLLQHDMRHVQQNRIVYEKTQREYEQEQRDMLSSQDGDTCEIHDQFYRDHKLLLVLFRALAVMPITRSRPGTITFSWRSTATMYAVCFYIAATAVVMIVGYERIMILRSIRRFDEYIYAILFVIFLVPHFWIPFVGWGVAHQVAIYKTNWGKFQVRYYRVTGENLKFPNLKTTIVMISVGCLLLAVCFLLSLCILMDGFLLRHTTAYYHIITMINMNCALWYINCKGIKIASQSLSECFRRDVEAECSAKLISRYRYLWLNLSELLQSLGNAYARTYSTYCLFMFANITIAVYGALSEIVDHGFGFSFKEMGLFVDAAYCSTLLFIFVDCSHNSTLTVAAGVQETLLSIDVLSVDRPTQKEIDHFIQAIEMNPAVVSLKGYAHVNRELLTSAISMIAIYLIVLLQFKISLPRDPQIVAT

>HarmGR3

MTVPIPNGFPVQINSKPKNKIIFLDVTPVSTPIKPHSPNVVAPMRNNLVAPHISNDIIYENIKPVFTLLRIMGVLPITRPSACVNQFQIASSSMLYAILVFLSLVSYVLYLSLHKVQILRTAEGKFEEAVIEYLFTVYLFPMIAVPLLWYETRKIANVLNGWVDFEMVYKQLSGRTLPVKLYKKALAMAVIIPILSTTTVIVTHVTMVHFKPMQLVPYVFLEILTYMLGGYWYLLCETLSICANILAEDFQNALRHIGPAGKVAEYRALWLRLSKLSRDTGIANCYTFTFVNLYLFLIITLSIYGLLSQISDGFGIKDIGLALTAFCSISLLFFICDEAHYASHNVRTNFQKKLLMVELSWMNTDAQTEVNMFLRATEMNPSQISLGGFFNVNRTLFKSLLATMVTYLVVLLQFQISIPDESQNRDEEEEVPYNITSATTEAMTTSTTTIMTTVLTTLAKKKKKN

>HarmGR4

MLNLCVSEIENKSVRNISSMREVLIDKIVKQKLDGLIGRLSGAIFYGNALISLFLSSKFVHSWRSLSNYWLRMETSTALDFPPDVRIRKRTIYITAFVVSVAVVEHILSMISATGVGFPPEEFLYRYVTLSHGFILKAQDYTIWKAIPIFVLSKLATALWNFQDLIIILISMGLSSRYNRLNLYVRHIVSVEKQFESKQRFGTELYLQIQVWRRLREAYVRQSTLVRMVDRNLGSLVLLSNINNLYFICLQIYLGIHKSSGSTISRCYFLFSLGWLIFRACSVVLAASDVHLHSQRALKSLHACPSAAYNVEIKRLQYQLAHDFVALTGMGFFSLRRELLLEVAAAILKYELVLIQYDK-

>HarmGR5

STELRTLMQNGWNNVISNISVGSVNTVNYLFRTWERLAPNRNMDLYSLEKFKKYKNDWNYPVHVRYQDQVMAEKEKPRTTFQTAMKVTLTIGQCFGLNPVQGIREKDASKLRFKLLSGRCLFTFFSLIGQFIMAFVLFLSLFKETSSTVDTATALIFYSFGFTTTILFFRIATNWPKLCMHIAKVESVDPNTDNKLGKKFNIACISILFLALMEHLFSELHGISIALDCFPDTPVYESFMKLSFQWLFGFIPYSDFAGGMAHFSNLQCTFNWNFADVFVICMSMYLTARLEQVNQRIIAAKDKNSPSSFWRTMREDYNRSVHLVRQVDKIIGGVVFMSFASNLFFVCSQLLHTLAGGIKASQRCKPEIGADRRFFYGYEHSIYFVFSFSFLVIRSLAVSLTASKVHAASLEPAYSLYDVSSANYCVEVERFLDQIHGDTVALSGLQFFHVKRGLVLTIAGTIVTYELVLMQFTGITPTTSPESVSGVIK-

>HarmGR6

DCSLTRKCNITCAVVLILALCEHILSLLSAFAGASACYSGMDTYEGFVTHFYPWVFSYLPYSIVLGVITQFLHFQSTFIWNFSDLFVICMSYYLTSRLEQVNRKLLAAQGKYLPEIFWRATREDYCRATQIVRKVDEVISGVVFISFANNLFFICLQLFNTLEDGLKGTGECTPKLKKIVVSKSGPLGGHEAAAYFLFSLVYLLSRSVAVSLIASQVNSASSVPAPVLYDVPSPVYCVEVQRFLDQVNGDKVALSGLQFFSVTRGLLLTVAGTIVTYELVMFQFNSSTPTLNITSP

>HarmGR7

AILSLVLSSRLYRSWGQLSALWARVERIMAVKAPPDKTLKRRMYFFLGFMTVCSLLEHIMSVVSAIGLDCPPALIIKRYVLISHGFMILRHEYSDWYALPLIFMSTLASLLWNFQDVLIVLISMGLTSRYSRLNQCLAKICALERKQMDSDKKNETTKVYAWRKLREAYVKQAMLVRKVDDAIGGIIILSCFCNFYFICLQLFLGITQSKASEPIKTAYYFMSLGWICFRVICVVLAASDINVHSRLGLKYIYTHDSHSYNIEMGRLQDQLSKDYVALSGKGFFYLSKSILLQMAGAIITYELMLIQFDDQGTDDV

>HarmGR8

YNWRTNIAGLWGKVERSVGVKIPVDKTLKCRMSFVAGLMTFCSFFEHALSILASVGFDCP

PSLILKRYVLVSHGFIFMGQDYSEWFAMPLVIISTIATLLWNFQDQLIVLISMGLTSRYR

RLNECLAKFCELEKQHMDSDKKVEAVKVYTWRKIREAYVKQAMLVRKIDVALGGIIILSC

SCNFYFICLQMFLGITQGMSTDFLTGLYYMVSLAWLCIRVLSVVLAASGVNTHSKLALNH

LYTYETHCYNVEVERLQDQLTKDYIALSGMGFFYLNKTILLQMAGAIITYELVLIQFDDQ

GSDGIALNA

>HarmGR9

MGVESAKVEEVTAAPVPSESGARPSRPTHCVVGGAHAFILRISSFFGLAPLRFESRSNGFTVSISGAMCVYSYILVTVLVICTIFGLVAEINVGVELSVRMSSRMSQVVSTCDVLVVVATAGAGVYGAPRRMRNMLKFMENIASVDTSIGGQYSLVTERKLCGIILAILIFFSILIADDFTFYALQAKKLDREWDVVTNYLGFYLLWFVVLILELQFAFTALSVRARFSAVNDALALTARQVSIPVEKPKSSSPLNIYAIRVAPVDSQRSANVSLLVDTMTGREHVVIIKRTASGEPRLVVSPCDAVRRLAALHGTLCDVVNSIDDSYGLPLVVILISTLLHLIVTPYFLIMEIIVSTNRIHFLVLQFLWCVTHMLRMIVVVEPGHYTIAEGKRTEGLVCRLMTSAPSTGVLPSRLEIFSRQLMLQSVSYAPMGMCTLHRPLIASVIGAVTTYLVILIQFQRYDN

>HarmGRx

ISSFTDLGAWVITYGWMIPVVHSLSYLYLLIKILATLDLIANIIQVEVRLRIINNFIKNC

YKCASACPVGILADCIRNKNWLHGEDGSPDQSLKARSIDSHEIKRLSKCYLLLTEQVMFI

NKMYGFRILLNTTSLLFDMVKILNLAIRIIVGSQRTLYNSAGYNFLPGVSGFVRFLTCAA

ILITLVNRCEQAYRQRERILNVIDHLLINK

>BmorGR1

DIYGPEITDKDDGALLDKHDSFYLNTKSLLVLFQIMGVMPIMRVPKSAQTTRRTTYNWISKATLWAYLVWGLECIIVVKVGQERLANFQIGSNKRFDEVIYNIIFLSILIPHFLLPIASWRHGPQVAIFKNMWTHYQLKYLKITGKPIVFPNLYILTWGLCIFSWVLSFAVVLSQHYLQDDFELWHSFAYYHIIAMLDGFCSLWYINCNAFGTASRGLAINLHKALEAEHPALKLAQYRHLWVDLSHMMQQLGRAYSNMYGIYCMVIFFTTTISLYGALSEILEHGLSYKEMGLFVIVAYCMTLLFIICNEAYHASRKVGHEFQDRLLNVNLGAIDRSTQREVEMFLVAIAKNPPIMNLDGFTNINRELFTANISFMSTYLIVLMQFKLTLLRQGARKTVTAIVRAIFNTTITDNGAGGSDEDQE

>BmorGR2

KEQEQRDLLSSQDGDTCEIHDQFYRDHKLLLVLFRALAVMPITRSRPGTITFSWKSTATIYAVCFYIAATAVVLIVGYERIQILQSIKRFDDYIYAILFIVFLVPHFWIPFVGWGVAHQVAIYKTNWGKFQVRYYRVTGENLKFPNLKTLIVIISVGCLLLAVCFLLSLCALLDGFLLKHTSAYYHIITMINMNCALWYINCKAIKIASQSLSECFQRDVDIECSAQLIARYRYLWLNLSELLQSLGNAYARTYSTYCLFMFANITIAVYGALSEIVDHGFGFTFKEVGLFVDAAYCSTLLFVFADCSHKSTLKVAAGVQDTLLSIDVLAVDRPTQKEIDHFIQAIEMNPAFVSLKGYAHVNRELLTSAISMITIYLIVLLQFKISLPKEPHGTGQ

>BmorGR3

MSFEIKNNFFRTSVPIPNGFPVQTEAKSKNKPIFLDVSPAPTPKVNSPNAIIPMKNNLIDPFINKDIIYENIKPVFMVLRIMGVLPLTRTTSGVNEFHFISPAMVYSLTVFIILVSYISYLSLHKVQIVRNSEGKFEEAVIEYLFTVYLFPLTVVPILWYETRKIANVLNGWVQFEVTYKQLSNRILPVKLYKKSLLIAIIIPILSTTSVIVTHVTMVHFKTSQIIPYVFLEILTYMLGGYWYLLCEILSLCANVLADDFQQALRHVGPAGKVAKYRALWLRLSKLARNTGVANCYTFTFVNLYLFLIITLSIYGLLSKISEGFGTKDIGLALTALCSVFLLFFICDEAHYASHNVRTNFQKKLLMVELSWMNTDAQTEVNMFLRATEMNPSQISLGGFFDVNRTLFKSLLATMVTYLVVLLQFQISIPDATQPEIPTNIDDHVQNITDTTTEASSPISTLMSAFAKRKND

>BmorGR4

MDKDKFQEFLPTMSRIFSMTRYFGVSTCKPSIAFGWTVILLLMLLAIEVGAIWKIVRLLGGWAVHSTDSRGFTARLSGCIFYGNALLSLILSIKFVSSWEQLSERWSRTETDPGLRLPSDSRIKRRTVLVSAFVMTCACVEHMLSMMSATGFDCPPEEYTERYILSSHGFLVQNDEYNLWLAIPIFIMSKLATALWNFQDLIIILISMGFTSRYNRLNTYVHRVVMLERNLKEGAQVSSENYMRFQIWRRIRQAYVRQAALVRLVDDQLGALVLLSNVNNLYFICLQLFLGINSKDRGSF

INRLYYFISLGWLMFRACGVVLAAADVYIHSKKALISLYLCPELAYNLEIKRLKYQLKNDEVALTGMGLFSLNRELLLEVAAAVLKYELVLVQYDK

>BmorGR5

YFAFYLSTGCNTFIFLRVASKWPTLIKHVYETQLDSYIDVKVKNKCFAAYIIFFSMSMTEHMLSLLSKFVITMDCLPKGSDLFESYIIRNFPWLFEFDVPYYLPIGVILQFLTLVSTINWSYSDLFIVCMSIYLTSILKQINKKIEMAGNSNHLPIPFWRTLREDYTRATRLVRSFDDTISSVIFLSFASNLFFICLQLYNILSNGVTSKYNLLKEMCPNYPSGPLGGYEQIMYLLFSLSFLLGRSLVVSLVAAKVHSASMVPASALYNIPRNMYCSEIQRFLDQVHGDKVALSGLRFFYVTRSLVLSVAGTIVTYELVLLQFSNED

>BmorGR6

MLLRNYKQNLSFWTSAKKSKIHKIQSQETVTFQGSLKLVLFIGQLFSLFPVCGLLSNDANKVXFVPISWKCGYSMLSMIGQLFIIVMCILYVAHFETTLNGTTPIIFYGVTFISMIAFIRASRRWPELIQHISKSEELDPSFDFRLKKKCNITLLLVLVLAILEHIFSIRSAYSASQICYPHTGFYEGFVRYLYPWVFDFLPYSEELGMVTQFLNIQSHFIWNFTDLFVICMSYYLTSRLDLVNKKLLPAQGKYLPEIFWRTTRETYCRATKLVRKVDEIINGILFISFANNLFFVCVQLFNTFDDSVDMVGLCYNYSERRTKPVGREPVIYLLFSLGFLISRSITVSLIASQVNLASTVPAPILYDVPSAVYCVEVQRFLEQVNGDNVALTGLQFFSVTRGLLLSVAGTIVTYELVMVQFNQAPASDSFTEKLVENNISTIETFYNYS

>BmorGR7

MVLEAHTQIQYCTAKANYCEFHAGLRHLMRLARWAGFFPVQGLSQTNPDDVRFEFRSLYALYHAITVIGQTVMTFLAFYSFVDSNVSLSVVSNFLFYFTNYVTLVLLWRLSKNWSALISKTLEFEQSVTEIRTTRNLVSRTNTLTYVVLIFAMVEHALSKVFNIRSVMCCLGETSLNHTVINNYFKFKWKFVFDYFSTSTTYSYFVGFIAEFLCMQATFLWSFTDVLIMCFSIYLSSFFEDFNSTVSSFMKKASKTVPWSTLRVQYSQIVLIVKQMDEQLDYFVLISYFTNLFFICFQLYNSLNRIYDANDVCNENMDIIATASVTYLTYYVFSFLFLVTRALLLSIMAANVHSCAQVPQLALYEVPTADYSLDVQRFQLQLRYTTVGLSGVCFNVTRGMILRVIGTIVTYELVLIQLTKKNLDNDTSIRDYYLPKHLI

>BmorGR8

MAPRSVRSMVGTSKKDMLKGGFYETVRIPLYIYRLIGILPISGLWHRSSKYNRFSLKSFYTIIYAPTIVMQTFLLLVHIYDLFAFFFGHQRLGRLIYHMNFYTITILIFMGSRKWKNVIKEIETIELTLPRLRNSKKALALTKSFVFAFFVFSLAEVVLILQFTLRLTKQRHVLPGDSGLYLRSYFVYIFPYLYDHFPFSYVMGFIVQIIKVQGIITLNMVNCSVVILSIYLTNRLKHYNRIVFAKGSKTNNTRLKWVELNLLYTRISNLVKIIDKNLNPFVFISFTANLSYICAQLFYILNKLTSSRTVKITSFLEDKRCDWETVLYISISFALVVLKVLLVSIIAAEVHTTSREPLRLLYTLPTAEYTIETQRLMTQVYYSNLSLSGLNFFHITRGMLLGMVATLLTYEIVLLQI

>BmorGR9

MPPSPDLRADEPKTPCLVGGAHAFILKISSFCGLAPLRFEPRSQEYAVTISKGKCFYSYILVTFLVICTIYGLVAEIGVGVEKSVRMSSRMSQVVSACDILVVAVTAGVGVYGAPARMRTMLSYMENIVAVDRELGRHHSAATERKLCALLLLILLSFTILLVDDFCFYAMQAGKTGRQWEIVTNYAGFYFLWYIVMVLELQFAFTALSLRARLKLFNEALNVTASQVCKPVKKPKNSQLSVYATSVRPVSCKRENVIVETIRVRDKDDAFVMMKTADGVPCLQVPPCEAVGRLSRMRCTLCEVTRHIADGYGLPLVIILMSTLLHLIVTPYFLIMEIIVSTHRLHFLVLQFLWCTTHLIRMLVVVEPCHYTIREGKRTEDILCRLMTLAPHGGVLSSRLEVLSRLLMLQNISYSPLGMCTLDRPLMVTVLGAVTTYLVILIQFQRYDS

>BmorGR10

MTMSIKPRLQCMVPPSLALALRVSRLAGIAPLKFVAKQSNIMIRLSTSLCVYSYLLVTALNVCTLIAVMIDFSVPVKLSIRMQTETKRFVWIADVVIVGMLSGVGVYTAPIQMRRLIAYLHRIHKINSDLQTYSSSLTDKMLHRLTIGMLLITSVIIVTDFTFVMYLADLNHRQLLIAIMYWCYYCSYFIAHLLEMQFVLIAALALSSLKLVNNGLRTMLHQSGIESLTEIPNSNEQHAANAVLPQPPKKSVNNSIDTLAFVVTKRSVRFPTAGWTDQRTIRRLALSYGSICEVVRQIDNNNGIIVLLLLASFLLHLVVTPYYLIISFVTESPHTGFEKVLNPILQTVWCLYHTFGLVMIIEPCHRTHEEMETTRELVSRVMCSADPRDPISVELEMFFRQLVLNKASYAPLKVCTLTRSLVATILGSITTYLIVIVQLEIKNMQ

>BmorGR11

MKPFRFFLFVENVICVYRNYSFHKRYARAIILSRVMFEVSLIILTLHSCRNFGAVKYKTEIIFTYLATASSTILILLALYKTNRFTELFLNFKAFYRNRNLDVDHLEKWNRKQKMATVIIVLFCVIKFSTLIYTDLIGEYSTPCRGYFTEYLFYTNLFMCNARYLFEFSTACVVLHLVSEQLDYIAISMDCTMFLYIDISKKNIMSSAKKRKLKYFDIFKQFEKWTDAYMNVKRSANLCDTVFRAQLAIMITTITLYYIILLYGITSFNIERGKFSVVKSLSYLISLFGFLIALLLLSKAGQRIQKSAENLRRKLSKFLLHSLEDPEFHRAATNLLRLVCTHHIKMRCFGFIDIDMTLLPSCLMFVTSYTVIALQFNNVV

>BmorGR12

MKNLKLCRTTFYFKIIMCSRFISGLYFTATSKKWISYLYKVICVLYIICITRLFYAKEDTFKPLVFXQFIGNSIESLRTGEGHVLKCYSTIFSLKLIRNYLPDSNNHIPISSITNFLVIIWKVFDQVYIVLMHYFYTTDIIIHLRILSILTTIGVNLSLMPIIVIFELMWRAVKALRKSLGEHLKGPVLIEGRERLKAQQILRCLNVYKDLNATLKFNSTPMKTMILISTLATFIRLTLFLYQAILGHNEGLHLPRKILAIIYYALPVCLLGVLMELVARECDKLKTLMTKELLVCKDDSYCTVIVDAVSYIELNPLKFSILRAFNVNSTLILGLTNLCTTYLIAVIQFTYSCEDINGLSHSHSH

>BmorGR13

MEDSFNRLLSIRNMIIFQNVCGFYHMCTEKLYISRIIKMYCVALAIVLSVFCFQNPDITYLSWDVVWVTFGYTLNVIICLRYNGNYFFQYWNGLHEIDIKMNLTSIDKEKVPISRAVFTVFLILRSTAFAMTIFVFGYLETGILSNTIISIYSINLTEFYRNMSNIPMILMFETFYVRIKILKEQLCSELSTVLGCNNDARQLKLILKYLRNYRSLVRHLMDTTLPFKILILVILVGSFLRSLLIGYAFVYNSDQIILLSLPVMFSTKILSEVVEIKLICTKELLKNKNEGLVLLDLDSKKPTFLTSKACGEQLQDALSFLNNRSYSYTLLQVIEFDCSLAFVFTSFCITHLIVVVQFTHVLD

>BmGR14

MNLHKNIIPIRNNLFANKVTAIALPKTLSVLFKLIHIFFLLDLGVYEYKTFKIKCIVKFLTISGSLTISVVCFSFMVSNLSEHTFVGWYGFFISTYIFVVLFFNLSNRMTFVEFYKTLLRFDANYGIDSNEYKFNFKIIFVNILFIANRMVLSFVYCSYYPQNCIRPRYAQILFMLPWLTLDVLLTTNMFLFYATYCRIAKFPMLIKNSMNIVALRNSYKLIVDSLEKTQTSFDIVFIIALVFSVPEIMMSIYSTLLEVISKHFLEVASILSLNYVAIAQSLLLTLAPSLCAGVLPWKTNNIKIILHEKLFTEKDKASAREIELFIKYIESRPLKLRACNLVPLDFSLTIIVLNICVTYLIVIIQFTHLY

>BmorGR15

MISSSDINHKRNKVFAYNVPGIALSKTLTVLFKLLHYVLLLDVGIYEYKTFKNKCIVKFLTIATGVSVSIVYFCLIATVLRKNAFFYWFYVLFISQYMIIVFIFTLSNGMSFTDYYKMLLRFDAKYQINSNNYYFNIKIILVIIISILNRIGMAIIYCSYYTKNCYEMSFSQIIFVLPWLTRDVILIMNVFLFYVTYCRITKFPALLENTKNVGSLRNSYKLIVDSLEKTQKPFDFVFTISLVFNIPEIMLSIYFTLLQVIHSHFLEVAPTLSISYFSITHSVVLILAPSLCAGVLPWKTNTIKIVLHDKLFLEKDKNSARNIKLFIKYIEARPLKLRACNLVPLDFSLPVIVLNLCVTYLIVIVQFSHLS

>BmorGR16

MIMNLTTDRISKRNKVFAYNVPEVTLPTTLKVLFKLIQFTLSLDFGVYKYKTFKMKCVAKVLTLAGCLAASAACVSLIISNIFENQLFFGWYTLFVCQYTIVIFMFTFSNGMTFIDYKMMLLRFDAKYQIDSNVYHFNIKIVLVVVISVTSRLFLCAVYCIYSTENCIKPWYNQLLFFPWLSLDIVLIMNMFLFYATYCRLAKFPSLFENPKNVVPLRNSYKLIVDSLEKTKKSFDAVLIAALIFNIPEIMMSIYYTLFQVMNKHFQEVAPVLSLSYFTIILSVLLILAPSLCAGVLPWKTRHMRLILLEKLFAEKDKNSAREIELFIKYIEARPLQLRACNLVPLDFNLPVIVLNLCITYLIVIIQFTHLF

>BmorGR17

MGFSLGTTALSMFFFEKPVVFTIIQITMIIVKPAKYKLSDPFRPKDTSKLSESIIMYFKLFHIFLGIDLGGFRYQNRQVKYAVRLISLIQPLAIYGLCIYALLKIIANTEFLWYTISFTEYVAMSVAITLFSNEMTYCNFMINLKFIDTKLKIGDESFRIGVKLISSTILIGVTRCFTTTTYCLLGFCAKPTAAQILFQIPWLTIDLMLLQYMFIFYACYCRLVKILRILKKRNTDIEEMRRIYKTLVDVLDRARAPFDLAYLLGLLFSIPDVLYSIYESIIKVGEINTAKALSMSIIYITNIQSLALMFAPALTAGFLPSLTMKMRIILHDKLLEEQDKKTYRHIVLFIKYIETCPLKLKACQIIPLDFSFPIIILNIVVTYLIVAIQLTHFL

>BmorGR18

MRRSTKVISMVNQSDKGEIKTCSRFMKIYFFVIYILTGFNFGFYTGRGLNFLRVIQASVLLLRFIIASNCIYIAFHFRLLEAIWYSLTFSESLAIVVCFMLSRSALSCKNLFEYLYSVDQELKKSVGPSIEVKLALYTVVVSVLRLTVYVFCAIAYYETLHEGFCVELVYNTPCYCSDLYLVIHFTIFHSVYCRLKALRISMNEKFDVYKGTLIYKSLIDNLEEIKKSLDVPFFVILLNAVAIAMINILVTLEISYGQTMKFIRTAPRYLETVLLFSSAFAPVLAADMMASEAQKIKVTLNNILQRDDSLLEDDRRKVKQFAGYVSARPFRLRACRVLSLDCTLPVTVLSICVTYLIVVVQFTHLY

>BmorGR19

MRRSTKVISLVNQSDKGEIKTCSRFMKIYFFVIYILTGFNFGFYTGCGLNFLRVIQASVLLLRLSVASYSMYIARYSPLLEVIWCCLTASENLAVVVCFMLSRSALSCKNLFEYLYSVDQELKKSVGPSIEVKLALYTVVVSVLRLIIYVFCATAYYRKLFDGLRLELLYHTPCYSLDLYLVVHFTIFHSVYCRLKALRISLNEKFDVYKGTLIYKSLIDNLEEIKKSLDVPLFVILLNAVAIAMINILVTLHISYGKTMKLITAAPRYLETVLLFSAAFAPVLAADMMASEAQKIKVTLNNILQRDDSLLEDDRRKVKQFAGYVSARPFRLRACRVLSLDCTLPVTVLSICVTYLIVVVQFMHLY

>BmorGR20

MRRSTKVISLVKQSDKGEIKTCSRFMKIYFFVIYILTGFNFGFYTGCGLNFLRVIQASVLLLRSIIASYSIYVAIHFRVLEAIWYCLTFSESLMVVVCFMLSRSALSCKSLFEYLYSVDQELKKSVGPSIEVKLVLYTVVVSVLRLTVYVFCAIAYYESLHEGFSVELIYNTPCYCSDLYLVVHFTIFHSVYCRLKALRISMNEKFDVYKGTLIYKSLIDNLEEIKKSLDVPFFVILLNAVAIAMINILVTLEISYGQTMKFIRTASRYLETVLLFSSAFAPVLAADMMASEAQKIKVTLNNILQTDDSLLEDDRRKVKQFAGYVSARPFRLRACRVLSLDCTLPVTVLSICVTYLIVVVQFMHLY

>BmorGR21

MAHRTNSINLFRSRPPDIRAGVGEPRIFSKFICGTMFTQKSLVNFDLGKTPRGGDQEHSKFFKIYFLAVHSVTALDFGFDRNAKKLTKILISMFSISVRMGLAAVSFMSLWGRPNALALGWAPGTLLCENILVAVTYSASRSTFKCGDLFADLSTIDELFGSACDYRIESKMLLFTATMTVLRVVIYSTSRLVRADGFDFVDVLEVLNNLETMCMYLFLTVYFFVLFSIYCRFKKLRELMKNDFEIRRANLIYIALKDCTDKIKQSLDVPFLVVLVFTVLVVMVDVFITLEMIISNKYNMAVYVVRYLEITLDFLMLFAPVLLADMMAVQVDGLKITLHDRLCLNNGVDMKRYSSLAEFIGYVEARGCRLRACRVVPLDLTLPVTVFNVCVTYLIVMIQFADLY

>BmorGR22

MTRFEFSQRTKTKSVSKTLNILFNCIYVLTCMDFGFSLGVYKRMKVLENISLVLRVMVAIMCAAMVMKQDILDSAWADITLTESLLVIVSFKLSKPKLSYRELLENLSIVDETQGAPPAGYKVERKLITYIAGVTALRLTVLCLYCVAHTEQYSIDNFIEFLYNVPCYCLDLYLIVHFIIFHSIYCRLRTLRKALSNNFDVYRAHLIYKTLIDCTEEIKKCLDIPLVVILIATILVVMVNVLVTLRMLFKGESIISAFLLRYIEVILSLALLFVPVLVADMMALEAHKIHIFLHGRMQTANDAAEERREVRQFAHYVGTRPFRLRACHVLALDSSLPITVVSVCVTYLIVIVQFTHLY

>BmorGR23

MAQFQIPSMAGSGLNVAPFSRGRRHEGPKHSNFTKKYFLLVHLVTCLDFGFHRDNDTKTYKWFHAANIGVRLVLSAYVCSVSLSQDLSFASAAWTILNNSKHLLVVAIFTIFKPKSSCAEILKDLLMIDEALKIHRGCDVKGQITVCIVLVTAARLLIAAASSLSLHEAFSASVGAAEVLYSFQSYCLDFYILANFFIFYSVYCRLKNLRRVLQNNFNIYRGNMIYKVLVEHMDDIKKFLDIPFVTSLLVTVIMAMINVLKTLQLIHDGENDVLTIVLRYLEMFLSFSLIFAPVILSDLMSIEADNINVVLHNYIYETDAAEERERRHLSQFAWYVSARPFRLRACRVLSLDCTLPVTVLSICVTYLIVVVQFTHLY

>BmorGR24

MCINKKIQSIIKSLVSIRTIMLVQSILGFYHKMSNNFFVSFLFLTYTTILISVLSFYSVNDVMAHKFAYTLSMILEYDINTILSLITAGRQYFNFFEEMKKIDFSIGFGELNIEDLPLSRTLFVTIFVTNILLSIMTAALILFFSTPFLIISSGSTYAMAIVFFGLSLNVLPRIIIFELIYKRIKYINLSLKRKLKALALECDHTIARFEIINENLIIYNKLLQSLGNVNVSLKSSILLTTFTCFFRCSLICYYVITMNEDKVYIMQIIELTKQTLFLGVLIILAEYIKNEIENLKMTVSLQLFTCTDTQLYHQVSDXFEIHRITPIQLCGFKNMSVDTNLFLGLINVCSTYLIIITQFLNAYVN

>BmorGR25

MFVKCLKYVKKFKPMFSVMFIMNFRLICGLYYRIHSDAFVCFVFKVYCILCSMFLFFTSSDLAAPFSRSIPILATLFEYVANVLDCILTGQSYFFHLRMELMRIDPRLRGLDRPPASSIVFTAILSYKIFILAVYIHGKARTTYLQYEWFSSIGIHLLVLFSNLVHMNRMLIFEMVTFSLEAQKKTLGELLKSSLRRERVERKCEILNRFLKTYKRIIELFNNTMAATKLMTLISVVSCFIRILTYLYQVLTTQLSSSSAGSIFTTRHLTFIVTFVREHVLSVYFLKKVGICQRLQINFKIKCNVKTETLELISQDDEYTEKLEDALDFINSCSSKITILRAMTVDATLPLTFISLCTTYIIVVIQFSHIYD

>BmorGR26

MNKTKIYRKKLDKNERLVCSVQPAMFARLIVGLYYDIKVSNRVKWMIKSYCISLSSFICYLIIFRDDNFSLHPKLTSVMEYITYVTFSFLTCDKYLFRYLRFNPRTDGYPIFLYLCKKFEKFFKIIICLFVSFKILGVVLMMQSWPILSTPKYIWGTLALHFLWLASHMGRLVFILVYGILFCRMRTIRIIFENRGFQNTPQNRLTPKRYILMYEAVLNSIESVDFPVKFLIFTFICCFAPKLVVSLFEIMEEMKKGELSLTTFIWFLVELSPSYLFLLLSAIALDLVSEDVQELLSITIDRRLNCKNEKERSEIQEFFQYLRNNPFNYTLWQVVSLNLRTLLVATSFSIANVIAIMQIKNSKI

>BmorGR27

MVFKYKIMTKAPKSLPVLKILMLFRLVFGNYFRLSSNRYINFLVKSYCSTFTILLSVMCGKRLKNDSPYMLSLTEYILNKILNYATSEGYIFKYCNSIKTCDKIMGFKKLPIITIDVFIAIIITVITRTAITIYFGFLFPFDKYQVVLYVGCIVFSNDLNSLTIMNVFGLLNNRMNLLRKSLEAMTVPINIIGKNEVAPKVRLVRNAFRYYSNLLDNLDSVNHCVQYSLSVTLLLKFPKAVLLCYDSIKTYFVKIDNNFAMDIVDPTEIILSIVVMSFPAMLCEMITNEVEKIKAILTKHLIQCSDNSLRFELNITLLYICHRPFKYILWRAIPLDTSVPIGIVSLIITYVIVLIQLLHFST

>BmorGR28

MAHKIATVGPTNATATVKNNKRKLKISKRVTIFKVVRCLRFILGHYTELTSSKLKAFLIKCCSLLLAVIIIYAPLNYIKMAYVMGLIEYLLFVLLSLFTGDEYFYKFHNSIKSIDVLMGYKRGKIIDSNAIIFLLSVITIMRIVIIYCRSTVLAFRFTIIGVYLAIFSLRISYMLITVIFFAMYHRMKFLRKKFEIITIPVTIIGKQKVASKIRLIRKYLINYHHLLDCLRDINGGLQYFLAIMIACNLPKYIFFAYSAIKIQVLEHITIHSAVQNVKCZRILFVVVPAIFAELTTAEVERIIDVINRQLLRCTDEHMELELKVALEFIRRRPFDYVIWRTVPLNASLPIAIISLCITYVVIVIQLTQFHDNF

>BmorGR29

MYLRSKKSRFKLFSFERMIKILLMICGHYVQTDSSNVVSSIHRIFSIVITICLCPYFQFNPFFFHVIESVWYSILSQFTQYGFFFRYCSTIKTFDLLSGFKQIPLYTKRVCFFLLITLLVRLIIVLIHFSAHQTKLKTFCAFLIILSANTGHILMTIMFSILNTRMTLIQKLFANNPIPVNIVGKNQNASHIKRVRKGLICYNNLLDTLKVAEKEIQFTLTVTYLCHVPTIICYVYFVITVIYKSKFSGYNLIPMLDMILACMAVTAPALFAELTKNTVDKIKKILGSQLLRCSDESLRYELEITLEYVIQRPFSFSIWRAVSLDASLPVAMTSLCITYVIVILQLTQLRP

>BmGR30

MYLRSKKSRFKLFSFERMIKILLMICGHYVQTDSSNVVSSIHRIFSIVITICLCPYFQFNPFFFHVIESVLYSILSQFTQYGFFFRYCSTIKTFDLLSGFKQIPLYTKRVCFFLLITLLVRLIIVLIHFSAHQTKLKTFCAFLIILSANTGHILMTIMFSILNTRMTLIQKLFANNPIPVNIVGKNQNASHIKRVRKGLICYNNLLDTLKVAEKEIQFTLTVTYLCHVPKIICYVYFVITVIYKSKFSGYNLVPLFDMILACMAVTAPAVFAELTKNTVDKIKKILGSQLLRCSDESLRYELEITLEYVIQRPFSFSIWRAVSLDASLPVAMTSLCITYVIVILQLTQLRP

>BmorGR31

MYLRSKKSRFKLFSFERMIKILLMICGHYVQTDSSNVVSSIHRLFSIAITICLCPNFEFNPFYFHVIESVLYSILSQFTQYGFFFRFCSTIKTFDLLSGFKQIPLYTKRVCFFLLITLFMRLFTVLIHFLAYQSKFVTFCAFIIMLSANTGHILMTIMFSTLHTRMKSIQKLFANNPIPVNIVGKNENASHIKRVRKGLICYNNLLDTLKDAEKEIQFTLTVTCLCHVPKIICYVYFVITVIYKSESLRYELEITLEYVIQRPFSFSIWRAVSLDASLPVAMTSLCITYVIVILQLTQFRP

>BmorGR32

MCYTNFVSRQVSKCINFFSTIRYVIYLRMFCGLYYNCSSSFKIRCIARLYCFIIYCLNLHYNLYIFTSSVSLTNFLHTFITLAEVSIHILFSLYTGESNFMSFCIEMNKLTSGPIDFVATKCVATHFIAFFVIGLHILSSTLICGAEVSCFTFSVVLASMTFLTTLLSRFTTIIMFDLVWIRMRSLRKILVNALESDLSEDEKVKSIESFLKAYKQIIASIRITKLATRNLVTFNFVSLFGKIMTLIYFCINCPGYLNTYLISSWIFGILLAGFVTCAPPVLVEMNVNELDEIKYALADQLVDYTDDNYRTAIYNALDYVEVHSIRYTLWKNFPMDLTMFFGFAGFCATYIIGLLQFTY

>BmorGR33

MCYTNFVSRQVSKCIHFFSTIRYIIYLRMFCGLYYNCSSSFKIRCIARLYCFIIYCLNLHYNSYIFTTNVSLTNFFHTFIILAEVSVHILFSLYTGESNFISFCIEMNKLTSDPNEFIATKCVTTHFIAYLVIVSHILSSTLICGARASCFTFSVILTSMTFLTTLLSRFTTIIMFDVVWIRMRSLRKILVNALESDLAENEKAKSIESFLNAYKQIIASTRITKLATRNLVIFNFVSMFGRIMTLIYFCINNPGYLDTYHMSLWIFGILLAGFVTCAPPVLVEMNVNELDEIKYALADQLVDYTDDNYRTAIYNALDYVEVHSIRYTLWKNFPMDLTMFFGFAGFCATYIIGLLQFTY

>BmorGR34

MCYTNFVSRQVSKCIHFFSTIRYVIYLRMFCGLYYDCSSSFKIRCIARLYCFIIYCLNLHYNLYIFTSGVSLTNFFYSFITFAEVSIHILLPLYTGESSFMSFCIEMNKLTSGPNEFIATKCVATHFIALLVIVSHILSSTLMCGARASCFTFSVILASMMFLTTLLSRFTTIIMFDVVWIRMRSLRKILVNALESDLAEDEKAKSIENFLNAYKKVIASIRITKLATRNLVTFNFVFMFGKIMTLIYFCINNPGYLNTYLISSWIFGILLAGFVTCAPPVLVEMNVNELDEIKYALADQLVDYTDDNYRTAIYNALDYVEVHSIRYTLWKNFPMDLTMFFGFAGFCATYIIGLLQFTY

>BmorGR35

MYSSLKLKDYIVNMESVMCSDQSITISFKNVFKVFFDYISSLDFMMVCRLCFGYYYEFNCSNLCKIMFKCFSISVCIFCVSMHLVQLISPPYLNHCVIMLESTVSIITSLVTEDKYFFEFCLDMKDINSMMNQPRNIKSFKIIYVIISGAICHVIRHISICREKALSFCFSTEYLTASFTIISGYWNYLNITMMFDLLYQRLVAVKQMLTNGLNICDTDEYKIKSVQKFIDVYKALTVSLSKTSNLIKHTVSLGIFCALWRIIFFVYYCISMDFQVESAQFITWVSSMCLSVFLVYIPALIVELCSNEVDAIKWILASELLEYRDKRLRTSLRDALDYIDVCPIDFEIWHCFPMNLSLCLGFIDISSSYIISILQFKY

>BmorGR36

MTVPYDKIKSALSKFVQLLFSINVVLFVRFLFGFYMKIGSRKYFHIATKIWIVTLTIFRVYFQCRNFMNYPSYALILHDFTCTVELVILCIISSLGGEQHFYTYCSEMAELIDNRKNKRASYFTTSTLLIGFIILIVPTSISCKKVSNALSMLFLNIFNYVACFMHHLTIIYVFELLWREIRKCRISLERLEIMSVDDKIMKIENFLDSYKRSLDSLNKANGVMIPTMALAYFAIIAKIVFFTYNILSLRGFNILVHGDSWLLSTSIAIIFICAPALLVELAANEVNKIQNLLAVELLKMKDDKFZYRATLYDALDYIEVRSVKSMIWRNFPMDLNLCVGFINLIITYVIGLLQLLY

>BmorGR37

MSAKINYQEKLCSIKSIMYLGLFCGLYFRSSTSRMMLLMTKVYCIVLLFLGISFHLNMLSSDLPTETTLHSSIIMLEFFIHIITSLSTGQAKFLHFCTEMMKINGHNSKGSLDFQLVITNIALIIIIITQTTSSLLYCIIRSKCLSHSYVFTVITSLCVLFSSFTMIIKYELIWNTVRSLKNTLVSNLDSFDLSEQEKVNSVYNFLSTYRDIKANVDLTIKGTRTTDCLHASARVSAIVLSVAVTCIAPILVEINVYEFGRIKFALADQLLEYTDSKLRRSVYDVVEYITVAPPTVTIWPEFTVDLGLYMDFIGLTVCYCIMILQFEY

>BmorGR38

MKPKYVTNIKLVLFLRFLCGYYYEMEIPRRLKTVAKAYCIFFLFFYLVLHHLYCSFSNHTAKWSLYLEYSIYVFMSLYSKKMYLMDYYTSSRIIDFEPHSRIYKKLNIYLAILIPFLVVLKIVNMVTFCLSKSFNCWSWVSLLHNLLWNFTVLGRIPPVFVFALLFCRTRIIRRTLVSITVGPGSTVLKSLFKCTRYWLTVLKRPNTHLNSXLTVFLLCSTPKLILETFLMLNKIKESGPIVEKLAVYAIETFYTHLFFVVSSILFDLINVDLQRIKILIVEKRMKTKNTKHRIEVEKLFQFVKSQTIECTLWRVLSLNVRNILSFVSFAVTTIIAVLQIKNNNIY

>BmorGR39

MNVSPQYSVLKIFKPLFKVQTLFGSVRVKVGDNGITKTTKLQKFYSIFNILFATTGHFYTSFVYSVCVPCVGNSVAETSMALIQIYAGHLMNSFIVFSNTFLHNEKNVQMFKSLCSIDELMKIVRLEHRDLKLFIAIILLLSSTVIMNVYFLIYMVIILPISEKWVPFANIGIMNEDLEAITFVSVLYMLYDRVKYINQTALDPVNIAKLIKESDGQNDEETVSRILKAFKEISKAYKIVEKTFRVFNLSWGTFMSKNLIGVKFIARIMLISVCVSYLERELQKTKGLCNLAVRNCENDIVRCHLKNIYRIIDTEIEPMTVFGLFYINNVPLDLISLTATYTVVLLQFAFL

>BmorGR40

MLPICYIKLVYNISSIMFLRLIFGLYFELNVSNRMRWLQRMYCVFISIVIMYFCYFKTYRILMTVKVPFILEYGMHVILSLYLRENFEYTRYDPIIDSAADTKNFYKKFDIFIKIFLFVAILLKMZSMLVFCVLWSGLCYTGFIGICGMNFIWIATFMGRMVLPLIFGILLCRIRMFRLTLQKQGFDNLPYNRFSPRRYIMMYDSIVRGLEKTDFPAKNIMFVFTICIYSKILTGLFDLISVLKREGPKLMNVMLFTLEFLPSYVLLMIYSVTLDMVSTEMKEILKIVTEKRVFYEKQHANIQELCQYIKNNQLKYTIWRLVSLNMQSLLRATSFCIVSTIAILQIKDWNG

>BmorGR41

MSENSLEEYIHMSFSPIYKYQKFLGSNRISLKAKNKITVANNWEKLYAFLWMLAASYSIHHFISFFYSYYYERSNIIFLACSLGISMHYLTYILTITYDKFLTREADIDLFIDIQKIDRLLKLDRCTVLFKKFRLINIFLLILVTVPFISGFLIHVFDYIDKPYKTFFLGLGVTITYVDVLVTAFFITKLTLRLAYINDRIAMYNKINIPHKKYSGIRRSILWIFGWRIFKIMPKIKKNG

TREKKSTFIKYPSIIFNILKCYRSITEIYSLPVFLITATVSIWTFLVIGSLVAGSRSEIKIFPVVAMITVGLWNFYYIIQLTSLAFVNDLFLMEVKNTKQLCISVLLYTCDDSINKAANTILKNIECVPPIFSVYGIFVFDKSIILFLFGIITSNVMTVIQFSY

>BmorGR42

MNKTKKIERLSRDILDEDFIDVFKSIFIFQRIFGLLSVNITYKYITETSKLYKLFVMSLWTVNVLCLVDYILNYRTSFDVATDSMLKLVMSVNVTTNALIVWRNNFKLNTLKSQIYVKLQNLDRDLKTKDAVTMNKKLSALSIALMICGFIWCTIWLFVYNAIAMNTFCVPLTIILSANVGNWLEMVLLFIIFYFVNVRAEYVNKLLRRRLNQTECPDRVFLIQNAKPSDTVSREFICGMQSLLEIIGNIKDIYQFPIFLSTCQVMLCILVIVQNLIISVKEQTSTMVDSMLCMLPALLLMLTIFFSLCVIAEALTSKLDITKKLCAMGMHSFTDDISRRNSXQIVLLLKAKRPLSVFKIYTLGTRLPIHLLGVTASYTIVLLQFAVL

>BmorGR43

MLKKSAMITMKSPEYLSKDILDEDFVRVFRFPFLVQMVLGSCRVHLKARFITIPTLGQKLYTVMSIIICSLLYFNITKLYISLYYEQSIVYFLFLTVAGLDQLSFFANLIHVRFLNGETNTGFCIMMQRIDRKMKIDHNNIFNKTVIRANILTITLIILLYMSLVISTIILKKYSLVTLFGLVHGQLILLVEMAYCSNLIIFFFIRVRFVNAIIKNHVHPENQNQPPKLVRYFVTNRIMRYLAAQTHDFIVNDTDVYLKQIFEGFSMFIDIYRFQVCLFCIKLIVMSLLTFEFCLVGIQKNGPLRKKATRMLKIIEESTPQFSIYDMWQMDGYTFVKICSLVTNLIVTSLQFAYL

>BmorGR45

MITMKSPEYLSKDILDEDFVRVFRFPFLVQMALGSCRVHLKARFITIPTLGQKLYTVMSIIICSLLYFNMTKLYLPLYYEHSIVYYIFVTVTGLDQLSFFANLIHLRFLNGETNTAFYIMMQRIDRNMKIDHNNIFNKTVTLANILTITLIILHYVGLVISTIILKEYSLLSLFGLLYGQLMLMVEMALCSNLIIFFFMRVRFVNAILKNHVHPENQNQPPKLVRYFVTNRITRYLAAQTHDFIVNDTDVYLKQIFEGFSMFIDIYRFQVCLFCIKLVVLSLLNFEFCLVGIQRNLLETKNLTNYYIMTYSVIGFFTALYVSGRCELFFREIRETKRLAVAVLLQYQEGPLREKATRMLKIIEESTPQFSIYDMWNMDGYIFIRICSLVTYLIVTLLQFAYL

>BmorGR46

MITMKSPEYLSKDILDEDFVRVFRFPFLVQMALGSCRVHLKARFITIPTLGQKLYTVMSIIICSLLYFNITKLYLPLYYQHSIVYYLFLAVTGLDQLSFFANLIHVRFLNGETNTAFCIMMQRIDRNMKIDHNNILNKTVIRANIFTITFIILIYVVLVISTIMLNEYSLVTLFGLLYGQLIFMVEMAHCSNLILFFFTRVRFVNAIIKNHVHPENQNQPPKLVRYFVTNRITRYLAAQTHDFIVNDTDVYLKQIFEGFSMFTDIYRFQVCLFCIKIVVLSLLTFELCFVAVQRNLLETKNLTNYYIMTYSVIGFFTALYVSGRCELFFREIRETKRLAVAVLLQYQEGPLREKATRMLKIIEESTPQFSVYDMWNMDGYIFIKICSLVTNLIVTLLQFAYL

>BmorGR47

MKIRFLFGFYCDFPFNKRFQNILKFYCISVLVVLILGSWACSTGFRSDKKIVIYCEYIAYFLISLSTKDRYIFDYYKQQPLIDGSTTSKVLYKKLERLLKYFVTITIVLKMLNIFVFCGWNLTKCINELDGVLFINLLWIGLLLARLSLPVIYGLLYFRLRVLRMTLESKGFSNSPQNRFTPKKYITIYEKIMKDLLKMDYPLKYVFIIFLIGSVPKLLQNSWQFLNSLKNYGPEISKILEFTLECLHSYIVIILPIVVALDLSEDEIKKMKIITLNKRLACLNERQKMEIQQLFLLLKNNSLRYNLWRVVPVNLKSVLIFLSFGVTNAIAIMQAKNLN

>BmorGR48

MSGNYLEEYINMSFSPIYKYQKFLGSNRISLKAKNKITVANNWEKLYAFLWMLAASYSIHHFISFFYSYYYERSKILFLGCSLGTSSQYLTYILTITYDKFLTREADIDLFINLQKIDRLLKLDRCTVLFKKIRLIYIFLLILVTVPFISGFLIHVFDYIDQPYKTFFLGLGLTIIYVDVLVTAFFIANLTLRLAYINDRIAMYYKRSLPLRKDSGIRRSRSWICGSWIFQIMPKIKNNG

TRMKNYTFIKYQSLIFNILKCYRLITEIYSLPVFLITATVSIWTFLVIGSIVAGSRSEIKLFSIVAMITVGLWNLFFIIQLTSLAFVNDLFLMEVKNTKQLCIRVLSYTRDDSINKAVNTILKDIEYAPPIFSVYGIFVFDKSIILFLIGIITGNIMTVIQFSY

>BmorGR49

MAGIRTISFKVKPLELPDVSENNFADDGLKIVQRFKFFIYIQVITGINRLYLLKCNKFVMLFSYLYAIFLISFVASVYWTKEPMKNSHLVIRLFSFIEYILLICISVFLKKKKMMKFFENLSMFDQILKIDKNVNSTFCMKRVFFWVTGSIVYNLIEFYALEFYDNTSKGLMTIICTYTIAPAAIGVCVYLIVHCIKYTLLVVIPCYYSSITATQVSLIRITLHDAINTIPLGKLQRRKVKAFYLMTKEYSFVYTLAGVIKLNMSLPLSYISLCTTYLVIIIQFSKFLD

>BmorGR50

MAGIRTISSKVKPLELPDVSENNFADDGLKIVQPFKFFIYIQAITGINRLYLLKCNKFVLMFSYLYAIFLISFVALVYWTTEPKKNSHLVIRLFTFFEYTLLACISVFLKKKKMIKFFENLSLLDKMLKINKNVNSTCCMKQVFFWVTGSIVYNLIEFYAMEFYDNTNKGLKTIICTYAIALAHDCEQIFFFTLQRVVYLRLLVVKRHIQEYFKVDEDSSRKKPNKYEMLSNNVQLNLTALHEVYALLHNCAEKLNTVMSIPVLLMLFTSGLSTTILLKFFVRVIQLTDPSNPGSAIGVCMYLIVRCIKYTLLVVISCYYSSITATQVSLIRITIHDAINTVPLGKLQRRKVKAFYLMTKEYSFVYALAGVIKLNMSLPLSYISLCTTYLVIIIQFSKFLD

>BmorGR51

MAMGIRTILSKVKPLELPDVSENNFADDGLKIVQRFKFFIYIQVLTGINRLYLLKCNKFVMLFSYLYAIFLISFVASVYWTKEPMKNSHLVIRLFSFIEYILLICISVFLKKKKMMKFFENLSMFDQILKIDKNVNSTFCMKRVFFWVTGSIVYNLIEFYALEFYDNTSKGLMTIICTYTIALTHDCEQIFFFTLQRVVYLRLLVVKRHIQEHFKVDEDSNRKKPNKYEMLSKNVQLNLTALHEVYGLLHNCAEKLNKIMSIPVLLMLFTSGLTTTILLRILVRVIQLADPSNPGSAIGLCVYLIVRCIKYTLLVVISCYYSSITATQVSLIRITINDAINTIAFGKLQRRKVKAFYLMTKEYSFVYTLAGVIKLNMSLPLSYISLCTTYLVIIIQFSKFFD

>BmorGR52

MAGIRTISSKVKPLELPDVSENNFADDGLKIVQRFKFFIYIQVITGINRLYLLKCNKFVMLFSYLYAMFLISFVVLVYWTTEAMKNSNLVIRNFTCLEYILLICIAMFLKKKKMIKFFENLSCLDKMLKIDKNVNSTCCMKRVSFWVAGSIVYNLIEFYAIEFYDNTNKGLVTIICTYTFALAHDCEQIFFFTLQRVVYLRLLVVKRHIQEYFKVDEDSSRKKPNKYEMLSNNVQLNLTALHEVYALLHNCAEKLNTVMSIPVLLILFTSGLSTTILLKILVRVIQFTDPSNPGSTIGVCVYLIVRCIKYTLLVVIPCYYSSITATQVSLIRITLHDAINTIPLGKLQRRKVKAFYLMTKEYSFVYDLGGVIKLNMSLPLSYISLCISYLVIIIQFSKFLD

>BmorGR53

MAHIKDENQSKQQQKEHETLNKNKLKKVVYTLKPALMLENWFGLSDFLLVNEDELVLLMQTEKFGVILSIFFIVMFAVFVDFPDTETESIMELMDEVPSMVVLSQYFIASITTSSCLSAIAIRIFETFADLDSMLLITTTQDFYNKSRYQTNKYLIILGVSHIISSTLDLLTDDEIVWCKFFVLPIYFLQKLEVLTFCKLIVMIQCRLQIINKYLTNFIEEQEKNKALVFTLAESNPKKTDKFNWIGCPSPNNMKIRDLATMYDVIGTICSLINDLFNIQIFMTLVSTFTYIVIAIWSTLYFYRAPNFTFGTLTTIIIWCITIILSVVVMSFVCERLVSVRNNTKILVNKVIMNYDLPKTMRVQAKAFMELIESWPLKIMVYDMFSVDISLMLKFISVATTYLIVIIQLSHFV

>BmorGR54

MITIKNNFGFRQNNLSFYRPILIILQLCGYDFDYYNINLVLNVLTKAYCASLTCVVVYATIACCSSIQLSHIWSLIEYGTSVVIIACFRSQTKLFLKQLTTLDVYLRISNRRFVLEKCKIFTITSVIFLLRIVYTSIYCSTHHCFNVLIYFLLSQFALVCLDVNRIWRCIVFDAIRYRLKTLRLRMEENPDCNYYLYVKNNKSIRKNKISFCLFLYRTIADLVDLVSPELNVSLFLSVACSLPKIVSNAYHLLLIIEDREPLETGGYVLMHTLQVSLLLFTPFIIVECYTMEVEKIKLYLVHRLIDENGEDTTMRDNIRLFLEYMSVRTFRYRIFRIVPVNATLPLELVNLCVNYVIVLINFTHLYG

>BmorGR55

MERINLLKSFAFLENVMCIYRNFMFYNQRARFIIIGRIVAELVFYIFSAYNGFLLVYTDWFSQNFSVFFIEIISKSSFYVITFFTMVNGILKSREYKTFIFSINKIHDYILNDTDYLKRLKCTNIFCTATIIILFVVTLIRTAIDGSNYGQLSGINARSVIWMLTTILLECQYQTECVVYFGFILFIHAIMKYLNIRVTNTIIKIARSDMAVKRIPKYIIGRTELKDETDTGVDVNNVVDLEEVRYWVFIYRQLGLTTELLQKCFGMQTAFIFVTAVLNQIITVFRVIAVFIYGSLANRGAEHSIIANFLFTLLYRLPGLLMIIVGGQMVQNQTDMLRRSMARLNNIISNPHRETFSALSDFHRMIVKNPVKIYVLSVLPVGAYMLPLFMTLLINHIIILLQFNHVA

>BmorGR56

MKKIRLLRSIVFLENLLCIYRNFLFFNKKARAIILIHITIELVLYVLSIVNNSFIIYSYFHSDNRSMLIVFTTICCFYVVTFVSIVMGILRSEEFKDLVTSLELINKFFTNNKTYLKSLGRSNTMIIAITTILYCVTCIGIAVDKITLNDFYEFTSSDVIWTVSSTLLELRYQTECVVYFGIEYLFLIFTKHLNLLVKEAIKKVSLDNNGTVKDVPISSDAVTKNEVKRWATIYRQLMMSSKLLQACFSLQIICVFVSAVINFITTAFRMVKVSVLGSIATDMNEIIIVNLIFTLLYQNIGLVLIIVTGQRVWNQILLLNVLLARLYNGILIQPCRDTLRTLKNLQRMVVKNPVQIKMLSVLPVGSYMLPMFMTLSVSYIIVMLQFGHVV

>BmorGR57

MEEIKAIKLVTFIENCICVYRNYAMCTKRNKKIISLRIIVEIIIVFFVNINNILLLHKYYNGSGLLYIIYLFLVVYYINYMFCIFYGALQGKAYRQLIFCFNKINAIAKRDKSYKKSLARLKNMCIVISIALLIISALSVFVDRSNSWNIYEVSLRDSLLILSKIHMDFFYHFEYVVYFTHIKIFHLTLRYLNSRVKMAQFEMKMTRRDVHDEGERNIRILLTKELTTEWAVLYKCLVFGTKTMKSLFGLQMLIAMVMSFVNFTLSLYGIILICSIEQSQTASQHNLLLILTYYTATMLLIFIVAQSVYNEVEMLKRNLARMYNILAVDSDETQQKLVKDFLRMVYKNKVEIKMLSIFPVGMPMLTFFLSLSASYVVVMVQFSNVF

>BmorGR58

MSSRRVLYRAEVLLSNNVDAHVQDMLKPLNFFQFILFFPKYTIRDGYITPNSLIRNIWSATGAFVFISICVFRILTMNKIAVYDTFTTMLLISKYFDVALYCIGFIVNTYVNIAYSNVNVLLYLKLQTIKTFIPRNNEIMKNVKWYSVILIIVLFCGTLAMFSFFHLSFSYFNIFDLTTDLAVFSFDLNLVYACSVLNFLAQSLDELNKEIWRLGNAKVTVCKDGSKPDWNGINLTYINVLDAYNYFKEAFRLLIFFHTFKTLTHMFIYIQSIIELCKKFYPGDDYDAITVGAVVGVWFFRNITLQCLVGVSCQNFYSATSNTESICAVQVGSIVSDEHKLFLKAVRRLNNVVFYKWSMYGMFIVDATLPRRLIELIATYTVVFLQFAFK

>BmorGR59

MPYKKDSNRCEVLLYNNVDTDLQDMLRPLNFIQTIYLSPKYTIKDGYITPNSLFCNILSAAGAIVFFSICVYRILTASKIGTFEGFSTTLLITKYFDAILFSLGFVANAYVSIRLSHLNVLLYLKLQAIKTFVPCKKIMQKVKYYSIVLIIGLIIVRLIMYIHFHWSLGYLSYLDLITDLGVISFDLNLVYASSIVKFLGYNLEELNKEILRLDEIKATMDEEGSKPDWNGIRRTYLKFSEAYNYFKDAFRILILFHTLNTFAHVFIYVQSVIELCKAPADNYSAAFSVLIAVIVWLLRNIILQSLIGISCQSFYSATSNTQSICSILVRSVLSDDQKLFLKTVQRLNKANISKLDVYGLFIVDATLPLRLIQVIATYTIVLLQFAFQ

>BmorGR60

MLTPRSDLCNEKLSPSFPSGKTTAADKDDTEARCQVDSSLERLLLPFNLVQHVSFIPMYSIRRGLVSPDGPLAYLYSLLGFCLFTSVSVYRNAIMHGTRLSSLHLFTLYSDLVSFVINYSLSLICNVVNSKSNVEFVCRLQRLQTVLRRNQREQEQFARSNWAHLAVVTALYLAVVGLLNVVVLKQSLPDTLYLLLLFCIDVNVLYATRMLALLRCYLQLWTRKINEKAFNPVHHNMFTAYLDILQEYEVYTTLFKKIITYYVLETFLHGLLYVQVAIQICKSIRRSGRFSEQLMMIVSIFTWTIKNMIIMTLHNVECEKFYLAVEQAVAACQTQRASTTRCREEKRLYKNVCRVSRAAFSRERGWGLLAAGAALTLRFMDLATTYVTVLLQFAFVSRT

>BmorGR61

MSIRFEKDLLHNYVEIELQYFLRPFNVMQSLFFQSKYRIVDNFILPNTLFKNIMSFVVSVLCALSFIYTIISVWQNTHATSFHALVTSVYLSYNIYGILIGSVLIIWLSDRNIEFVLKIQDLIKILEFNKCFLIEYAFINSIIMAAIFILNFLLYGYFVVHLQKFALGLTFSAIVCILNQDLDIIYVIIFANILKKCASRWTVEARQKNNFNDQGKWVKLFNAFLNLTESYQLYQKIFEFYELLRRVGIVFLGLQLTVCRVCSNDIKSIQCTVMLHAFQLICVWIVKKFITLSILSFEMEIFYEKLREIETVCIILVSSDNPSERELKIWKNIIRVSSCSVRKTTACGLCEVGAALPQWLLQATTAYTIVLLQFHITTFSRARNDIYDLD

>BmorGR62

MNDLFLSKIVKWTKTTKYKLDDDFQSLFRVFNIAQAMNLCPKFLIYDKYITNNAWFIHILAISSFIVLVCLDSFFANFRLVLSEAMGPPFYGFSFYFISILYENIGVIIQITMNGYLTKNNVLIITKLQDTFKDFRTTDYITKSNRWTNWFIFFIYMNFIANYSYFNFYVNTFSFHKFCFAFIKMCFDLNIVYTIFIFKMIGDSLTMFKDTAFCSKNMKLYEVSNRVYWNKMLRLYSNILDVFELSKRTLNFFIFYFVSNILLRILSHVQLAILMNSINWLQHVAYSNIVMVLLTLAKEGIILIVLIAKCEKIYCVIGDVQTACQLALGNAACPEKRRFCKNVRRSSSAAFSKIYICNILAVDAKLAVSLMSVTTTYTIVMLQAILIK

>BmorGR63

MQIGNAVIHLKSTKLTTMNTISPTTKLLKIFALNSNIEEIDLKCSTKLRITMTAFVLCSLIFYSLYYKFIYVFDYVNISIKITDCVQMVYDFCQYIVDLYFVTNYGRNISSEYFQQYKIIDKILEVVCYEIIKHRIVKLLWVFMCIWFSSSCFDFIAWFLNYGWITPLVYSVAYIFLLIKILTTLDLSAHIMNVEIRLKMIADLIHHYYMSCEDNFQAEETLCHKNWLNSKERAKYYELQFRIHALKQLSCNNNEIKLLSRCYLMLTEQVEIINRMYGFRILLNSLSLLIDMVRFTNISVRIMIGSQNLAYNCGYFPAVSSIFRLLTCGAVIINLVSHCERVYYQRTRICNVIDHMIVNKNLSRESTEALQEFRNLVQNHPIEFNMANFFQLNYSLLVSIASVVVTYTIILLQSVN

>BmorGR64

MKISLRKIVSIRNMTLIQNMFGFYHKFTDNRAIGVLLKIFCGFYSLFLSFLCINCTPRFTNDFLTYDIFFFVIEYLTSVLVCLLYDGQYFLNYLYDLKLIDREAGIEESLEKLPISQPLFSLIFITRVIYLLSCLLMFDGIKDSLFLPAQSSVFGANFTEFARTIGYFPRVIMFEMFYKRVNYLKSQLRNDLAHANLYPIGFVCSKVIMKYINFYKLLLRNLQQNSLQFKILMSMSSLYIIIKALASAYAFIYREDGVHVFIFIEFATGVFLFFVMSSIIISIFNEIEDIRQIVLAQLRYCKQGANTKRVQDALTILNIRCFKYALCRIYTVDFTFILRILDVSVTYVIVLVQFTHILD

>BmorGR65

MKISLRKIVSIRNMTLIQNMFGFYHKFTDNRAIGVLLKIFCGFYSLFLSFLCINCTPRFTNDFLTYDIVVFVIEYLTSVLVCLLYDGQYFFNYLYDLKLMDREAGFEESCSQKLPISQPLFCFVFITRVIYLFLCLLMYDGINDTLFLHGQISVFGVHVFIFIEFAIGVFLFFVMSSIIISIFNEIEDIRQIVLAQLRYCKQGANTKRVQDALTILNIRSFKYALCRIYTVDFTFILRILDVSVTYVIVLVQFTHVLD

>BmorGR66

MKRKLKKFFPNKEYNNIVEATHLWKLIRKLTGLSVLTLESKEGNRIETRFSSLGFVFFLLWFTIYFYCTYKAHNEDQTILRNIYSTKLQRYGDDFERITSIIYVLYSMWKLPFQISGNRLLLQEIVDIDKAIESVGVTIDYKKNATFALFIYIGQIATYLFRLFCVWGCLGNLNSPVPVEKLYQDIFTDALSLLLTSQYCFSLVILRDRCRYINKILCGIENRESSRLRLFVYSSMPGAEKDITCRKIKDCSKIYGMIYKAVESTNITYGFALVLTMLLYLIFIILYMFYFMEATAAGLFLDTKKYIDFLICVLSELLHAMLIIFLNIYFSEETVKETRTTSFV

>BmorGR67

MRERKKKFNKLLNTRNYNNIVEALLPSDSIRKISGVSVVYLAVNSENRIVTKFSFIGTIFFLFWYILYFYCTYKAHSEDQTILRTIYNTKLKRYGDDFERIASIIYVTYSMWKVPFRMSGNQVFIQRIVDIDSAIENMGEAVDYNKNAKTALVISIAQLGDFLVRMFCIWLSLENLSVIVPTEKLYQVVYTDALSFVITSHYCFSLIVLRGRYKYINKVLSEIKTRSAWEYKVFVRNKVAPDLEKVQRLQDRIVCEKIKACARIYSMLYKATEAINRMYGTALVLTMLLYLVFIILYMFYFMEATASGLLYDIKKYVDFLICVFWQMSHALSIIYANVYFSESITREVCKF

>BmorGR68

MRFGLKAGAAVVTILRPYNLCLKNIFKPFYVMLSLLGLFPYSIRFLGGKQFLIKPKSIYTNAVCALSLMLSMTLFLIFHIDHIIYKSTEDNSLTEGFMTQVNYIIEMLNLEIFCVVYYFSSFLNRNKFVKVLNTVAVWSDRISISGIKTLSFLRLKIHFSIGILMFLLISQVCVNFTRVDSLWKKVLVMFTFNIPQMIQFTAILFYYILVNMVITLLVIIQENISISTRDTKTSSFIRVEHRMPLSLKQLELIYIKAFELKRDINKAFEAPILLTTMQCFHSIVSESHIIYHGAVMEPHMVLHSIMNCSVWILYQLFKLYILASTGHLLQEKIQHFSNLIHFHGKGLTVYGLFPLDGTLMFKVVASAAMYLIILVQFDKRN

>BmorGR69

MNCIVKLLTITGSLTISVVCSSFMISNLSEHAFVGCYFEPCLCNFNPRNGIRPRHAETMFVIPWLTLGVVLAINMFLFYATYCRIARYPSLLEKIKHVVPLRNSYKLIVDSLEKTQKSFNIVFIISLIFNVPETMLSMDSRAHSPPGVKIKTCLRACNLVPLDFSLTIIILNICLTYLIVIIQFTQLY
